# Supplementary material for: Designing chemical systems for precision deuteration of medicinal building blocks
Source: Nat Commun. 2024 Oct 1;15:8473. doi: 10.1038/s41467-024-52127-6 (PMC11442640; doi:10.1038/s41467-024-52127-6)
Supplement: Supplementary file 1 — Supplementary Information [file 41467_2024_52127_MOESM1_ESM.pdf]

Supplementary Information

Designing Chemical Systems for Precision Deuteration of Building Blocks for Medicinal Chemistry

Jonathan D. Dabbs, Caleb C. Taylor, Martin S. Holdren, Sarah E. Brewster, Brian T. Quillin, Alvin Q. Meng, Diane A. Dickie, Brooks H. Pate, and W. Dean Harman*

Department of Chemistry, University of Virginia, Charlottesville, Virginia, 22904

Supplementary Methods

S1-2 Supplementary Information Title Page and Table of Contents

S3 General methods

S4 Abbreviations

S6-32 Compound Synthetic Methodologies and Characterizations

S5 Synthesis and Characterization of d_5-pyridine borane (10)

S6 Synthesis and Characterization of [W] pyridine borane Complexes (2 & *d_5*-2)

S7 Synthesis and Characterization of [W] pyridinium Complexes (3 & *d_5*-3)

S8 Synthesis and Characterization of [W] (*N*-mesyl) pyridinium Complexes (4D & *d_5*-4D)

S9-10 Synthesis and Characterization of [W] 1,2-DHP Complexes (5, *d_1*-5, *d_5*-5, *d_6*-5)

S11-12 Synthesis and Characterization of [W] 1,2,6-allyl Complexes (6, *d_1*-6, *d_5*-6, *d_6*-6, *d_7*-6)

S13-16 Synthesis and Characterization of *Rel*-[W] 1,2,3,6-THP Complexes (7, *(3S)-d_1*-7, *(2S)-d_1*-7, *(2S,3S)-d_2*-7, *(2R,3R,6R)-d_5*-7, *(2R,6R)-d_6*-7, *(3R,6R)-d_6*-7, *(6R)-d_7*-7, *(3R)-d_7*-7, *d_8*-7)

S17-19 Synthesis and Characterization of *Rel*-1,2,3,6-THPs (14, *(3S)-d_1*-14, *(2S)-d_1*-14, *(2S,3S)-d_2*-14, *(2R,3R,6R)-d_5*-14, *(2R,6R)-d_6*-14, *(3R,6R)-d_6*-14, *(6R)-d_7*-14, *(3R)-d_7*-14, *d_8*-14)

S20-21 Synthesis and Characterization of [W] THP ring-opened Complexes (8 & 9)

S22 Synthesis and Characterization of [W] MPH 1,2-DHP Complexes (11 & *d_5*-11)

S23-24 Synthesis and Characterization of [W] MPH 1,2,6-allyl Complexes (12, *d_1*-12, *d_5*-12, *d_6*-12)

S25-28 Synthesis and Characterization of *Rel*-[W] MPH 1,2,5,6-THP Complexes (13, *(5'R)-d_1*-13, *(6'S)-d_1*-13, *(5'R,6'S)-d_2*-13, *(5'S,6'R)-d_5*-13, *(6'R)-d_6*-13, *(5'S)-d_6*-13, *d_7*-13)

S29-31 Synthesis and Characterization of *Rel*-MPH 1,2,5,6-THPs (15, *(5'R)-d_1*-15, *(6'S)-d_1*-15, *(5'R,6'S)-d_2*-15, *(5'S,6'R)-d_5*-15, *(6'R)-d_6*-15, *(5'S)-d_6*-15, *d_7*-15)

S32 Synthesis and Characterization of Hydrogenated Piperidines (16 & 17)

S33- 93 NMR Spectroscopy: ^{1}H & ^{13}C NMR and 2D data Supplementary Figures 1 – 121

S94-97 SC-XRD Data Supplementary Figure 122-125; Supplementary Tables 1 – 4.

S98-132 Molecular Rotational Resonance Spectroscopy Data

S133-143 Molecular Rotational Resonance Spectroscopy Results

S135-143 MRR Results & Proposed Mechanisms: Supplementary Figures 122-130

S144 Supplementary References

General Methods

NMR spectra were obtained on an 800 MHz spectrometer. Chemical shifts are referenced to tertramethylsilane (TMS) utilizing residual ^{1}H or ^{13}C signals of the deuterated solvents as internal standards. Chemical shifts are reported in ppm and coupling constants (*J*) are reported in hertz (Hz). Infrared Spectra (IR) were recorded on a spectrometer as a glaze on a diamond anvil ATR assembly, with peaks reported in cm^{-1}. Electrochemical experiments were performed under a nitrogen atmosphere. Most cyclic voltammetric (CV) data were recorded at ambient temperature at 100 mV/s, unless otherwise noted, with a standard three electrode cell from +1.25 V to -1.25 V with a platinum working electrode, *N*,*N*-dimethylacetamide (DMA) or acetonitrile solvent, and tetrabutylammonium hexafluorophosphate (TBAH) electrolyte (~1.0 M). All potentials are reported versus the normal hydrogen electrode (NHE) using cobaltocenium hexafluorophosphate ($E_{1/2}$ = -0.78 V, -1.75 V) or ferrocene ($E_{1/2}$ = 0.55 V) as an internal standard. Peak separation of all reversible couples was less than 100 mV. All synthetic reactions were performed in a glovebox under a dry nitrogen atmosphere unless otherwise noted. All solvents were purged with nitrogen prior to use. Deuterated solvents were used as received from Cambridge Isotopes.

NMR assignments of all compounds were determined using 2D NMR methods including NOESY, COESY, HMBC, and HSQC. When possible, pyrazole (Pz) protons of the (trispyrazolyl) borate (Tp) ligand were uniquely assigned (e.g., "PzB3") using two-dimensional NMR data (see Supplementary Figure 1). If unambiguous assignments were not possible, Tp protons were labeled as "Pz3/5 or Pz4". All *J* values for Pz protons are 2 (±0.4) Hz. BH peaks (around 4-5 ppm) in the ^{1}H NMR spectra are not assigned due to their quadrupole broadening; however, confirmation of the BH group is provided by IR data (around 2500 cm^{-1}).

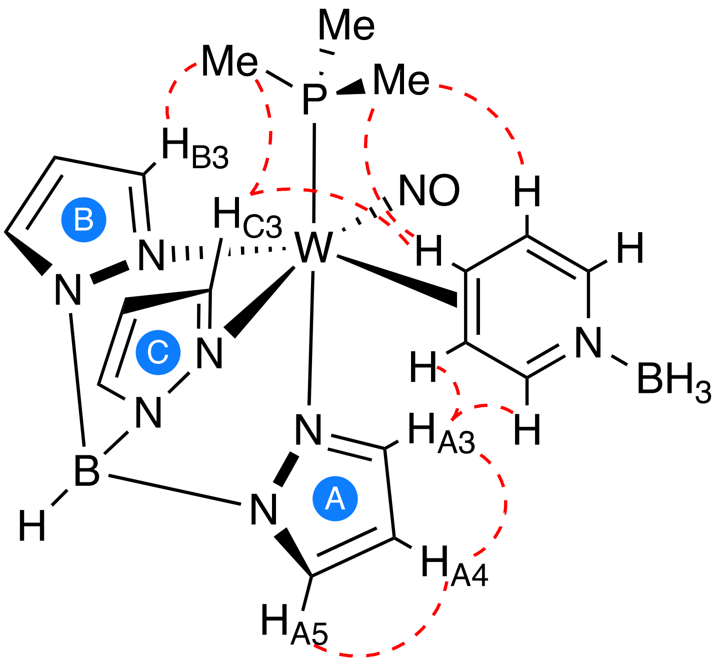

Abbreviations:

Acetonitrile	MeCN
2,3-dichloro-5,6-dicyanobenzoquinone	DDQ
Dichloromethane	DCM
Diethyl ether	Et_2O
Diphenylammonium Triflate	DPhAT
Ethyl Acetate	EtoAc
Magnesium Sulfate (Anhydrous)	$MgSO_4$
Methanesulfonic anhydride	Ms_2O
Methanol	MeOH
Propionitrile	EtCN
Aqueous Sodium Bicarbonate	$NaHCO_3$
Sodium Borohydride	$NaBH_4$
Sodium Borodeuteride	$NaBD_4$
Tetrahydrofuran	THF
Triethylamine	TEA
Triflic Acid	TfOH
Triflic Acid-d_1	TfOD

COMPOUND SYNTHETIC METHODOLOGIES AND CHARACTERIZATIONS

Synthesis and Characterization of *d_5*-pyridine borane (10)

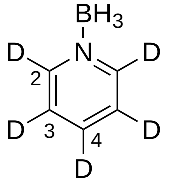

Conditions adapted from Ramachandran et al. [1] *on page S6*

A 1 L Erlenmeyer flask was set up on a hot plate in a fume hood with a 2" stir bar, and stirring was commenced. Finely ground $NaBH_4$ (33.7 g; 0.89 mol) was added to the flask followed by $NaHCO_3$ powder (150 g; 1.79 mol) and d_5-pyridine (50 g; 0.59 mol). This slurry was then diluted with 550 mL of dried THF. A 75 mL addition funnel was positioned over the top of the large flask and filled with dH_2O (32.5 g; 1.80 mol), which was then added dropwise to the slurry. Bubbling should occur quickly after the dH_2O begins adding. After all the dH_2O is added, the slurry was stirred for 23 hours. A 150 mL C frit was set up over a 1 L filter flask and filled with 1" of anhydrous Na_2SO_4. The slurry was the filtered through this plug, which was then washed an additional 100 mL of dry THF to rinse any residue off the plug. This solution was transferred to a 2L round-bottom flask and reduced to ~60 mL. This solution was added to a 1L separatory funnel, diluted with 300 mL of Et_2O, and washed 3x with 300 mL of brine. The combined aqueous layers were then back extracted with another 200 mL of Et_2O. The combined organic layers were then flushed through a silica plug. The silica plug was then washed with an additional 50 mL of Et_2O. These combined organic layers were dried with anhydrous Na_2SO_4 and then reduced *in vacuo*. The remaining liquid was then dried under 4Å molecular sieves in an inert N_2 atmosphere. (35.37 g; 60% yield).

^{1}H NMR (CD_2Cl_2, δ, 25 °C): 2.35-2.86 (q, BH_3).

^{13}C NMR (CD_2Cl_2, δ, 25 °C): 146.9 (*t*, J_{DC} = 28.1 Hz, 2C, C2), 139.1 (t, J_{DC} = 25.6 Hz, 1C, C4), 125.2 (t, J_{DC} = 25.8 Hz, 2C, C3).

Synthesis and Characterization of [W] pyridine borane Complexes

WTp(NO)(PMe$_3$)(η^2-(*N*-borane)-pyridine) (2)

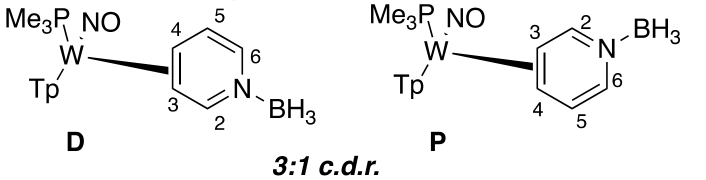

Previously described by Harrison et al. [2] *on page S4*

WTp(NO)(PMe$_3$)(η^2-(*N*-borane)-2,3,4,5,6-pentadeuteropyridine) (*d*$_5$-2)

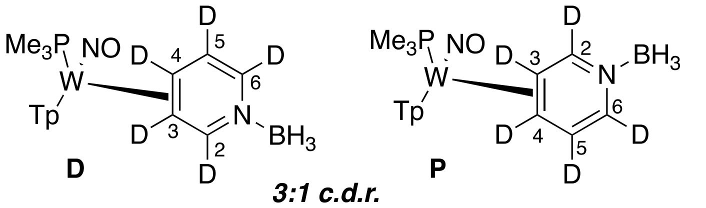

An oven-dried round 100 mL bottom flask with a 1” stir egg was charged with **1** (10.4 g, 17.02 mmol), d$_5$-pyridine borane (50 g, 0.51 mol), and TEA (3.1 g, 30.6 mmol). A yellow slurry was formed initially that gradually turned into a dark green/brown solution, which was stirred for 68 hours and monitored by ^{31}P NMR. After stirring, the solution was added to a 2L filter flask containing a 2” stir bar. To this filter flask was added 100 mL of dry THF, 400 mL of Et_2O, and 1 L of hexanes. This solution was stirred for 5 minutes and then allowed to settle for 15 minutes. Separately, a 2” celite plug was set up in a 150 mL C frit over another 2 L filter flask. After settling, the solution was then slowly poured through the celite plug taking care not to pour the lower thick oily layer. The eluent of the solution was discarded. This constituted 1x “dilution,” which was designed to remove excess pyridine borane ligand away from the coordinated complex. Dilutions ere repeated until a yellow powder is precipitated, which required 3x total dilutions. This yellow powder is then collected a 150 mL M frit. The oily residue on the celite plug was then dissolved off the celite plug with 100 mL of DCM. This solution was reduced to ~30 mL added to 200 mL of a 10:1 hexanes/Et_2O mixture, which precipitated a yellow powder that was isolated on the same frit as the previous fraction. The combined powders were then dried in a desiccator. (7.60 g; 74% yield)

^{1}H NMR (CD_2Cl_2, δ, 25 °C):
D: 7.99 (d, *J* = 1.7 Hz, 1H, Pz3/5), 7.90 (d, *J* = 1.6 Hz, 1H, Pz3/5), 7.85 (d, *J* = 2.3 Hz, 1H, Pz3/5), 7.83 (d, *J* = 2.1 Hz, 1H, Pz3/5), 7.71 (d, *J* = 2.2 Hz, 1H, Pz3/5), 7.16 (d, *J* = 1.8 Hz, 1H, Pz3/5), 6.35 (t, *J* = 2.3 Hz, 1H, Pz4), 6.32 (t, *J* = 2.2 Hz, 1H, Pz4), 6.28 (t, *J* = 2.2 Hz, 1H, Pz4), 1.25 (d, J_{PH} = 8.4 Hz, 9H, PMe_3).
P: 8.14 (s, 1H, Pz3/5), 7.91 (s, 1H, Pz3/5), 7.85 (s, 1H, Pz3/5), 7.84 (buried, 1H, Pz3/5), 7.73 (s, 1H, Pz3/5), 7.16 (s, 1H, Pz3/5), 6.35 (s, 1H, Pz4), 6.30 (s, 1H, Pz4), 6.27 (s, 1H, Pz4), 1.31 (d, J_{PH} = 8.2 Hz, 9H, PMe_3).

^{13}C NMR (CD_2Cl_2, δ, 25 °C):
D: 169.4 (t, J_{DC} = 26.5 Hz, C2), 144.5 (d, J_{PC} = 1.5 Hz, PzB3), 144.4 (Pz3/5), 140.6 (Pz3/5), 137.5 (Pz3/5), 137.1 (Pz3/5), 136.2 (Pz3/5), 126.5 (t, J_{DC} = 27.7 Hz, C6), 125.0 (t, J_{DC} = 24.0 Hz, C5), 107.1 (Pz4), 106.8 (Pz4), 106.7 (Pz4), 60.4 (t, J_{DC} = 20.6 Hz, C4), 57.4 (t, J_{DC} = 23.6 Hz, C3), 13.1 (d, J_{PC} = 28.7 Hz, 3C, PMe_3).
P: <Not reported due to peaks being too small in the baseline or ambiguous>

Synthesis and Characterization of [W] pyridinium Complexes

WTp(NO)(PMe$_3$)(η^2-(*N*-H)-pyridinium) (OTf) (3)

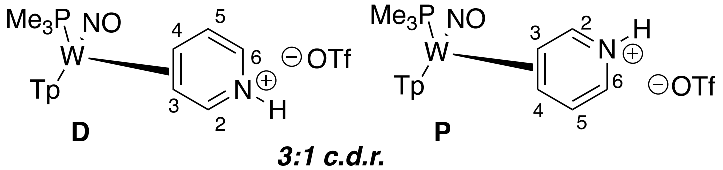

Previously synthesized by Harrison et al. [2] *on page S5 and characterized by Delafuente et al.* [3] *on page 415*

Synthesis and characterization of WTp(NO)(PMe$_3$)(η^2-(*N*-H)-2,3,4,5,6-pentadeuteropyridinium) (OTf) (*d*$_5$-3)

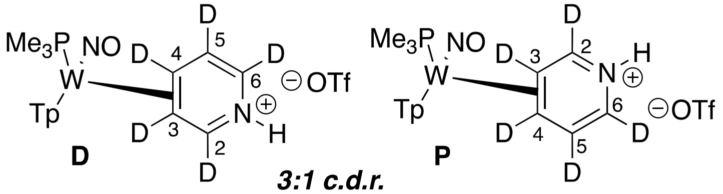

A 1 L Erlenmeyer flask containing a 2” stir bar was set up over a hot plate. 400 mL of Et_2O and **2** (7.6 g; 12.65 mmol) were added to the flask and stirred for 1 hour to loosen the clumps of tungsten in an evenly stirred slurry. DPhAT (4.24 g, 13.28 mmol) to a tared 4-dram vial along with acetone (22 g, 378.79 mmol). This solution was pipetted until homogenous and then slowly added to the slurry. Bubbling commenced immediately followed by a gradual color change from a yellow slurry to an orange slurry. This was stirred for 90 minutes. Upon completion, this powder was collected on a 150 mL M frit and dried in a desiccator. (9.035 g; 97% yield)

^{1}H NMR (CD_2Cl_2, δ, 25 °C):
D: 11.96 (br s, 1H, N-H), 8.21 (s, 1H, Pz3/5), 8.19 (s, 1H, Pz3/5), 8.08 (s, 1H, Pz3/5), 8.04 (s, 1H, Pz3/5), 8.02 (s, 1H, Pz3/5), 7.78 (s, 1H, Pz3/5), 6.48 (t, J = 2.0 Hz, 1H, Pz4), 6.45 (t. J = 2.0 Hz, 1H, Pz4), 6.44 (t, J = 2.1 Hz, 1H, Pz4), 1.18 (d, J_{PH} = 9.1 Hz, 9H, PMe$_3$).
P: 12.38 (br s, 1H, N-H), 8.21 (s, 1H, Pz3/5), 8.19 (s, 1H, Pz3/5), 8.08 (s, 1H, Pz3/5), 8.03 (s, 1H, Pz3/5), 7.97 (s, 1H, Pz3/5), 7.74 (s, 1H, Pz3/5), 6.47 (t, J = 2.0 Hz, 1H, Pz4), 6.43 (t, J = 2.1 Hz, 1H, Pz4), 6.40 (t, J = 2.1 Hz, 1H, Pz4), 1.23 (d, J = 8.9 Hz, 9H, PMe$_3$).

^{13}C NMR (CD_2Cl_2, δ, 25 °C):
D: 168.1 (t, J_{DC} = 25.9 Hz, C2), 145.3 (Pz3/5), 144.8 (Pz3/5), 141.9 (Pz3/5), 138.1 (Pz3/5), 137.6 (Pz3/5), 136.8 (Pz3/5), 125.9 (t, J_{DC} = 23.3 Hz, C5), 120.7 (q, J_{FC} = 322.1 Hz, -OTf), 115.7 (t, J_{DC} = 26.7 Hz, C6), 107.5 (Pz4), 107.1 (Pz4), 106.8 (Pz4), 64.9 (br s, C4), 57.0 (br s, C3), 11.8 (d, J_{PC} = 29.9 Hz, 3C, PMe$_3$).
P:163.2 (br s, C2), 144.5 (Pz3/5), 141.8 (Pz3/5), 140.7 (Pz3/5), 138.0 (Pz3/5), 137.4 (Pz3/5), 136.7 (Pz3/5), 128.5 (br s, C5), 113.5 (br s, C6), 107.3 (Pz4), 107.0 (Pz4), 106.4 (Pz4), 61.9 (br s, C4), 59.0 (br s, C3), 12.2 (d, J_{PC} = 29.4 Hz, 3C, PMe$_3$).

Synthesis and Characterization of [W] (*N*-mesyl) pyridinium Complexes

WTp(NO)(PMe$_3$)(η^2-(*N*-mesyl)-pyridinium) (OTf) (4D)

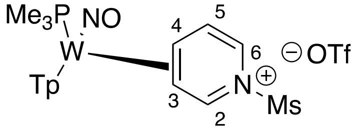

Previously described by Dabbs et al.[4] *on page S5*

WTp(NO)(PMe$_3$)(η^2-(*N*-mesyl)-2,3,4,5,6-pentadeuteropyridinium) (OTf) (*d$_5$*-4D)

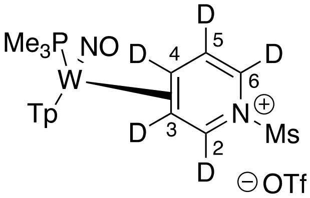

Ms_2O (5.36g, 30.77 mmol), **3** (9.035 g, 12.26 mmol), and EtCN (30 mL) were charged to a flame-dried 100 mL round-bottom flask with a 1 inch stir bar. Lutidine (3.36 g, 31.36 mmol) was added to initiate the reaction, and the flask was submerged in a pre-heated oil bath set to 55 °C. After stirring the solution for ~2 hours, the solution was diluted with 150 mL of DCM and added to a separatory funnel. This solution was washed 3x with 200 mL of saturated aqueous $NaHCO_3$. The organic layer was isolated and set aside. The combined aqueous layers were combined and back-extracted with 50 mL of DCM to prevent loss of product. The organic layers were combined in a single flask and dried with anhydrous $MgSO_4$. This powder was then filtered off into a 60 mL coarse porosity fritted funnel and washed with DCM. The dried organic layers were then reduced *in vacuo* down to dryness. The residue in the flask was redissolved in minimal DCM (approximately 10 mL). The solution was then slowly added to 500mL of stirring Et_2O. An orange precipitate formed immediately and was allowed to stir for ~10 minutes to ensure total precipitation. This powder was collected on a 60 mL medium porosity frit and washed 2x with 30 mL of ether. This powder was dried in a desiccator under vacuum for ~30 minutes. The dried powder was gently added to a stirring solution of 150 mL of HPLC grade EtoAc and triturated overnight. The final orange precipitate was collected on the F frit, washed 2x with 30 mL of ethyl acetate and 2x with 30 mL of Et_2O, and dried in the desiccator overnight under vacuum. (5.3 g, 53% yield)

^{1}H NMR (CD_2Cl_2, δ, 25 °C): 7.96 (d, *J* = 2.2 Hz, 1H, PzB3), 7.96 (d, *J* = 2.3 Hz, 1H, PzC5), 7.93 (d, *J* = 2.4 Hz, 1H, PzB5), 7.90 (d, *J* = 2.1 Hz, 1H, PzA3), 7.76 (d, *J* = 2.3 Hz, 1H, PzA5), 7.64 (d, *J* = 2.0 Hz, 1H, PzC3), 6.47 (t, *J* = 2.3 Hz, 1H, PzC4), 6.44 (t, *J* = 2.3 Hz, 1H, PzB4), 6.37 (t, *J* = 2.3 Hz, 1H, PzA4), 3.61 (s, 3H, Ms), 1.25 (d, J_{PH} = 9.2 Hz, 9H, PMe_3).

^{13}C NMR (CD_2Cl_2, δ, 25 °C): 167.2 (t, J_{CD} = 27.6 Hz, C2), 146.5 (PzA3), 145.6 (d, J_{PC} = 1.9 Hz, PzB3), 141.7 (PzC3), 138.7 (PzC5), 138.5 (PzB5), 137.6 (A5), 123.3 (t, J_{CD} = 25.1 Hz, C5), 121.3 (q, J_{CF} = 322.1 Hz, $^-$OTf), 114.7 (t, J_{CD} = 29.2 Hz, C6), 108.2 (PzC4), 108.1 (PzB4), 107.8 (PzA4), 66.1-66.5 (m, C4), 65.4 (t, J_{CD} = 26.3 Hz, C3), 43.9 (Ms), 12.9 (d, J_{PC} = 31.5 Hz, 3C, PMe_3).

HRMS (ESI) m/z: $[M]^+$ Calcd for $C_{18}H_{22}D_5BN_8O_3PSW^+$ 666.1575; Found 666.1581

Synthesis and Characterization of [W] 1,2-DHP Complexes

General Synthesis

Two separate solutions were prepared in two 30 mL glass screw-cap test tubes. Tube A was comprised of **4D/*d*$_5$-4D** (967 mg, 1.19 mmol) dissolved in EtCN and Tube B was a heterogeneous slurry of $NaBH_4$/$NaBD_4$ (4.49 mmol) in dry THF (2 mL). After both tubes were chilled in a 0 °C toluene bath for 15 minutes, Tube B slurry was added to Tube A, and the tube sat for 30 minutes. The solution was diluted with 150 mL of DCM and added to a separatory funnel. This solution was washed 3x with 200 mL of saturated aqueous $NaHCO_3$. The organic layer was isolated and set aside. The combined aqueous layers were combined and back-extracted with 50 mL of DCM to prevent loss of product. The organic layers were combined in a single flask and dried with anhydrous $MgSO_4$. This powder was then filtered off into a 60 mL coarse porosity fritted funnel and washed with DCM. The dried organic layers were then reduced *in vacuo* to dryness. The residue in the flask was redissolved in minimal DCM (approximately 10 mL). The solution was then slowly added to a stirring 200mL solution of 9:1 pentane/Et_2O. A tan precipitate formed immediately and was allowed to stir for ~10 minutes to ensure total precipitation. This powder was collected on a 60 mL medium porosity frit and washed 2x with 30 mL of pentane. This powder was dried in a desiccator under vacuum overnight. (625 mg, 79% yield)

WTp(NO)(PMe_3)(η^2-(*N*-mesyl)-1,2-dihydropyridine) (5)

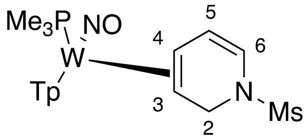

^{1}H NMR (CD_2Cl_2, δ, 25 °C): 8.09 (d, J = 1.8 Hz, 1H, PzA3), 8.03 (d, J = 1.8 Hz, 1H, PzB3), 7.77 (d, J = 2.3 Hz, 1H, PzB5), 7.75 (d, J = 2.2 Hz, 1H, PzC5), 7.67 (d, J = 2.3 Hz, 1H, PzA5), 7.31 (d, J = 2.0 Hz, 1H, PzC3), 6.34 (t, J = 2.2 Hz, 1H, PzB4), 6.27 (t, J = 2.2 Hz, 1H, PzA4), 6.23 (t, J = 2.2 Hz, 1H, PzC4), 6.01 (d, J = 7.8 Hz, 1H, H6), 5.78 (dd, J = 4.4, 7.8 Hz, 1H, H5), 4.60 (dd, J = 2.6, 11.6 Hz, 1H, H2), 4.58 (dd, J = 4.6, 11.6 Hz, 1H, H2'), 2.95 (s, 3H, Ms), 2.72 (ddd, J = 4.4, 10.4, 12.4 Hz, 1H, H4), 1.53-1.57 (m, 1H, H3), 1.24 (d, J_{PH} = 8.4 Hz, 9H, PMe_3).

^{13}C NMR (CD_2Cl_2, δ, 25 °C): 143.7 (d, J_{PC} = 2.2 Hz, PzB3), 143.1 (PzA3), 140.5 (PzC3), 137.0 (PzB5), 136.5 (PzC5), 136.0 (PzA5), 119.0 (C6), 114.6 (d, J_{PC} = 3.6 Hz, C5), 106.8 (PzB4), 106.3 (PzA4/C4), 106.2 (PzA4/C4), 52.3 (J_{WC} = 16.4 Hz, C3), 47.2, (C2), 46.5 (J_{PC} = 10.8 Hz, C4), 37.9 (Ms), 13.7 (d, J_{PC} = 28.1 Hz, 3C, PMe_3).

IR ν(NO) = 1567 cm^{-1}
CV (DMA; 100 mV/s) E_{pa} = +0.45 V (NHE)
SC-XRD Data on S95

WTp(NO)(PMe$_3$)(η^2-(*N*-mesyl)-2-*anti*-deutero-1,2-dihydropyridine) (d_1-5)

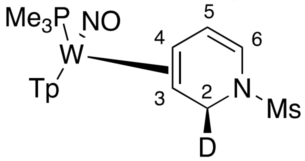

^{1}H NMR (CD_2Cl_2, δ, 25 °C): 8.09 (d, *J* = 2.0 Hz, 1H, PzA3), 8.03 (d, *J* = 2.0 Hz, 1H, PzB3), 7.77 (d, *J* = 2.3 Hz, 1H, PzB5), 7.75 (d, *J* = 2.3 Hz, 1H, PzC5), 7.67 (d, *J* = 2.4 Hz, 1H, PzA5), 7.31 (d, *J* = 2.2 Hz, 1H, PzC3), 6.34 (t, *J* = 2.2 Hz, 1H, PzB4), 6.27 (t, *J* = 2.2 Hz, 1H, PzA4), 6.23 (t, *J* = 2.2 Hz, 1H, PzC4), 6.01 (d, *J* = 7.8 Hz, 1H, H6), 5.78 (dd, *J* = 4.4, 7.8 Hz, 1H, H5), 4.58 (s, 1H, H2), 2.95 (s, 3H, Ms), 2.72 (ddd, *J* = 4.4, 10.3, 12.3 Hz, 1H, H4), 1.52-1.56 (m, 1H, H3), 1.24 (d, J_{PH} = 8.4 Hz, 9H, PMe$_3$).

^{13}C NMR (CD_2Cl_2, δ, 25 °C): 143.7 (d, J_{PC} = 2.2 Hz, PzB3), 143.2 (PzA3), 140.5 (PzC3), 137.0 (PzB5), 136.5 (PzC5), 136.0 (PzA5), 119.1 (C6), 114.7 (d, J_{PC} = 3.0 Hz, C5), 106.8 (PzB4), 106.3 (PzA4/C4), 106.2 (PzA4/C4), 52.0 (J_{WC} = 32.8 Hz, C3), 46.9 (t, J_{DC} = 21.0 Hz, C2), 46.6 (d, J_{PC} = 10.8 Hz, C4), 37.9 (Ms), 13.7 (d, J_{PC} = 28.0 Hz, 3C, PMe$_3$).

WTp(NO)(PMe$_3$)(η^2-(*N*-mesyl)-2-*syn*-3,4,5,6-pentadeutero-1,2-dihydropyridine) (d_5-5)

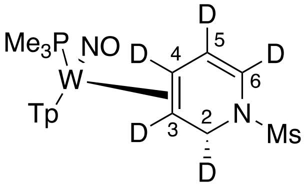

^{1}H NMR (CD_2Cl_2, δ, 25 °C): 8.10 (d, *J* = 1.7 Hz, 1H, PzA3), 8.04 (d, *J* = 1.9 Hz, 1H, PzB3), 7.78 (d, *J* = 2.2 Hz, 1H, PzB5), 7.76 (d, *J* = 2.1 Hz, 1H, PzC5), 7.68 (d, *J* = 2.3 Hz, 1H, PzA5), 7.31 (d, *J* = 2.0 Hz, 1H, PzC3), 6.34 (t, *J* = 2.1 Hz, 1H, PzB4), 6.26 (t, *J* = 2.2 Hz, 1H, PzA4), 6.24 (t, *J* = 2.1 Hz, 1H, PzC4), 4.56 (s, 1H, H2), 2.96 (s, 3H, Ms), 1.24 (d, J_{PH} = 8.4 Hz, 9H, PMe$_3$).

^{13}C NMR (CD_2Cl_2, δ, 25 °C): 143.7 (d, J_{PC} = 2.0 Hz, PzB3), 143.1 (PzA3), 140.5 (PzC3), 137.0 (PzB5), 136.5 (PzC5), 136.0 (PzA5), 118.5 (t, J_{DC} = 27.7 Hz, C6), 114.1 (t, J_{DC} = 24.0 Hz, C5), 106.7 (PzB4), 106.3 (PzA4/C4), 106.2 (PzA4/C4), 51.7 (t, J_{DC} = 21.9 Hz, C3), 46.7 (t, J_{DC} = 21.7 Hz, C2), 45.5-45.9 (m, C4), 37.8 (Ms), 13.7 (d, J_{PC} = 28.1 Hz, 9H, PMe$_3$).

WTp(NO)(PMe$_3$)(η^2-(*N*-mesyl)-2,2,3,4,5,6-hexadeutero-1,2-dihydropyridine) (d_6-5)

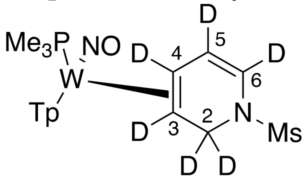

^{1}H NMR (CD_2Cl_2, δ, 25 °C): 8.10 (s, 3H, PzA3), 8.03 (s, 3H, PzB3), 7.78 (d, *J* = 1.9 Hz, 1H, PzB5), 7.76 (d, *J* = 1.8 Hz, 1H, PzC5), 7.68 (d, *J* = 2.0 Hz, 1H, PzA5), 7.32 (s, 1H, PzC3), 6.34 (t, *J* = 2.0 Hz, 1H, PzB4), 6.26 (t, *J* = 2.0 Hz, 1H, PzA4), 6.24 (t, *J* = 1.9 Hz, 1H, PzC4), 2.96 (s, 3H, Ms), 1.24 (d, J_{PH} = 8.5 Hz, 9H PMe$_3$).

^{13}C NMR (CD_2Cl_2, δ, 25 °C): 143.6 (d, J_{PC} = 2.2 Hz, 1C, PzB3), 143.1 (PzA3), 140.5 (PzC3), 137.0 (PzB5), 136.4 (PzC5), 135.9 (PzA5), 118.6 (t, J_{DC} = 27.4 Hz, 1C, C6), 114.1 (t, J_{DC} = 23.6 Hz, 1C, C5), 106.7 (PzB4), 106.3 (PzA4/C4), 106.2 (PzA4/C4), 51.2 (t, J_{DC} = 22.1 Hz, 1C, C3), 46.4 (t, J_{DC} = 21.2, C2), 45.7-46.0 (m, C4), 37.9 (Ms), 13.7 (d, J_{PC} = 27.9 Hz, 3C, PMe$_3$).

Synthesis and Characterization of [W] 1,2,6-allyl Complexes

General Synthesis
An oven-dried 4-dram vial containing a stir pea was tared, and ***d*$_x$-5** (275 mg, 0.41 mmol) was massed out and diluted with dried MeCN (2 mL). To a separate 4-dram vial was added dried MeCN (1 mL) and TfOH^x (63 mg, 0.42 mmol). The acid/MeCN solution was slowly added to the ***d*$_x$-5**/MeCN solution and stirred until solution was homogenous (about 2 minutes). The reaction was then slowly added to 100 mL of stirring Et_2O, which immediately formed a tan precipitate. This powder was triturated for 5 minutes and then collected on a 30 mL medium porosity frit. The collected powder was then washed 2x with 20 mL of Et_2O and dried overnight in a desiccator. (300 mg, 89% yield)

WTp(NO)(PMe$_3$)(η^3-(*N*-mesyl)-1,2,3,6-tetrahydropyridinium (OTf) (6)

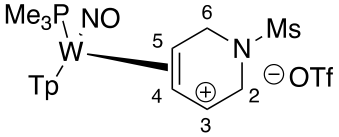

^{1}H NMR (CD_2Cl_2, δ, 25 °C): 8.35 (d, *J* = 2.1 Hz, 1H, PzB3), 8.13 (d, *J* = 2.2 Hz, 1H, PzC3), 7.98 (d, *J* = 2.2 Hz, 1H, PzA3), 7.94 (d, *J* = 2.3 Hz, 1H, PzC5), 7.87 (d, *J* = 2.4 Hz, 1H, PzB5), 7.71 (d, *J* = 2.4 Hz, 1H, PzA5), 6.58 (t, *J* = 2.3 Hz, 1H, PzC4), 6.52 (t, *J* = 2.3 Hz, 1H, PzB4), 6.44 (d, *J* = 7.2 Hz, 1H, H3), 6.31 (t, *J* = 2.3 Hz, 1H, PzA4), 5.10 (t, *J* = 7.7 Hz, 2H, H4), 4.90 (d, *J* = 18.1 Hz, 1H, H2), 4.81 (dd, *J* = 3.0, 12.6 Hz, 1H, H6), 4.52 (d, *J* = 18.1 Hz, 1H, H2), 4.43-4.47 (m, 1H, H5), 4.00 (dd, *J* = 2.0, 12.7 Hz, 1H, H6), 2.90 (s, 3H, Ms), 1.27 (d, J_{PH} = 9.7 Hz, 9H, PMe_3).

^{13}C NMR (CD_2Cl_2, δ, 25 °C): 147.9 (PzA3), 144.9 (d, J_{PC} = 2.2 Hz, PzB3), 142.8 (PzC3), 139.0 (PzC5), 138.8 (PzA5/B5), 138.7 (PzA5/B5), 127.4 (C3), 121.2 (q, J_{FC} = 321.8 Hz, -OTf), 109.0 (PzB4/C4), 108.9 (PzB4/C4), 107.7 (PzA4), 95.7 (d, J_{PC} = 2.8 Hz, C4), 65.7 (d, J_{PC} = 15.3 Hz, C5), 44.7 (C2), 44.1 (d, J_{PC} = 2.8 Hz, C6), 36.0 (Ms), 13.4 (d, J_{PC} = 32.4 Hz, 3C, PMe_3).

IR: ν(NO) = 1651 cm^{-1}
CV: (MeCN; 100 mV/s) $E_{1/2}$ = -0.78 V (NHE)
Unable to obtain EA, SC-XRD, or HRMS data for compound. Full 2D NMR provided below.

WTp(NO)(PMe$_3$)(η^2-(*N*-mesyl)-2-*anti*-deutero-1,2,3,6-tetrahydropyridinium (OTf) (*d*$_1$-6)

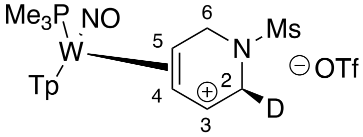

^{1}H NMR (d_6-acetone, δ, 25 °C): 8.61 (d, *J* = 2.2 Hz, 1H, Pz3/5), 8.41-8.42 (m, 2H, Pz3/5), 8.22 (d, *J* = 2.5 Hz, 1H, Pz3/5), 8.16 (d, *J* = 2.4 Hz, 1H, Pz3/5), 7.97 (d, *J* = 2.4 Hz, 1H, Pz3/5), 6.8 (d, *J* = 7.8 Hz, 1H, H3), 6.65 (t, *J* = 2.3 Hz, 1H, Pz4), 6.64 (t, *J* = 2.3 Hz, 1H, Pz4), 6.39 (t, *J* = 2.4 Hz, 1H, Pz4), 5.32 (t, *J* = 7.6 Hz, 1H, H4), 4.92 (s, 1H, H2), 4.88 (dd, *J* = 3.6, 12.8 Hz, 1H, C6), 4.65-4.69 (m, 1H, H5), 4.06 (dd, *J* = 1.9, 12.8 Hz, 1H, H6), 2.96 (s, 3H, Ms), 1.38 (d, J_{PH} = 9.9 Hz, 9H, PMe_3).

^{13}C NMR (d_6-acetone, δ, 25 °C): 149.1 (Pz3/5), 146.1 (Pz3/5), 143.6 (Pz3/5), 139.7 (Pz3/5), 139.5 (Pz3/5), 139.4 (Pz3/5), 129.6 (C3), 122.3 (q, J_{FC} = 322.4 Hz, -OTf), 109.5 (Pz4), 109.1 (Pz4), 108.1 (Pz4), 96.2 (C4), 66.2 (d, J_{PC} = 14.8 Hz, C5), 44.9 (d, J_{DC} = 21.1 Hz, C2), 44.7 (d, J_{PC} = 2.9 Hz, C6), 35.6 (Ms), 13.05 (d, J_{PC} = 33.1 Hz, 3C, PMe_3).

WTp(NO)(PMe$_3$)(η^2-(*N*-mesyl)-2-*syn*-6-*anti*-3,4,5-pentadeutero-1,2,3,6-tetrahydropyridinium (OTf) (*d$_5$*-6)

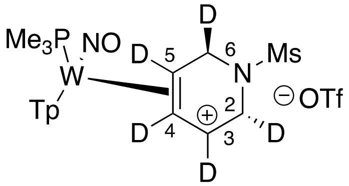

^{1}H NMR (CD_2Cl_2, δ, 25 °C): 8.35 (d, *J* = 2.1 Hz, 1H, PzB3), 8.12 (d, *J* = 2.2 Hz, 1H, PzC3), 7.99 (d, *J* = 2.2 Hz, 1H, PzA3), 7.94 (d, *J* = 2.3 Hz, 1H, PzC5), 7.87 (d, *J* = 2.4 Hz, 1H, PzB5), 7.71 (d, *J* = 2.4 Hz, 1H, PzA5), 6.57 (t, *J* = 2.3 Hz, 1H, PzC4), 6.52 (t, *J* = 2.3 Hz, 1H, PzB4), 6.31 (t, *J* = 2.3 Hz, 1H, PzA4), 4.52 (s, 1H, H2), 3.97 (s, 1H, H6), 2.90 (s, 3H, Ms), 1.26 (d, J_{PH} = 9.6 Hz, 9H, PMe_3).

^{13}C NMR (CD_2Cl_2, δ, 25 °C): 148.0 (PzA3), 144.9 (PzB3), 142.8 (PzC3), 139.0 (PzC5), 138.8 (PzA5/B5), 138.7 (PzA5/B5), 126.5 (t, J_{DC} = 25.4 Hz, C3), 121.3 (q, J_{FC} = 320.7 Hz, -OTf), 108.9 (PzB4/C4), 107.7 (PzA4), 95.4 (t, J_{DC} = 24.8 Hz, C4), 64.9-65.3 (m, C5), 44.3 (t, J_{DC} = 22.1 Hz, C2), 43.7 (t, J_{DC} = 20.8 Hz, C6), 36.0 (Ms), 13.4 (d, J_{PH} = 33.0 Hz, 3C, PMe_3).

WTp(NO)(PMe$_3$)(η^2-(*N*-mesyl)-6-*anti*-2,2,3,4,5-hexadeutero-1,2,3,6-tetrahydropyridinium (OTf) (*d$_6$*-6)

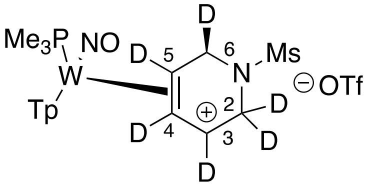

^{1}H NMR (CD_2Cl_2, δ, 25 °C): 8.36 (d, *J* = 2.1 Hz, 1H, PzB3), 8.14 (d, *J* = 2.1 Hz, 1H, PzC3), 7.96 (d, *J* = 2.2 Hz, 1H, PzA3), 7.94 (d, *J* = 2.2 Hz, 1H, PzC5), 7.86 (d, *J* = 2.4 Hz, 1H, PzB5), 7.71 (d, *J* = 2.4 Hz, 1H, PzA5), 6.58 (t, *J* = 2.3 Hz, 1H, PzC4), 6.52 (t, *J* = 2.3 Hz, 1H, PzB4), 6.32 (t, *J* = 2.4 Hz, 1H, PzA4), 3.98 (s, 1H, H6), 2.90 (s, 3H, Ms), 1.27 (d, J_{PH} = 9.6 Hz, 9H, PMe_3).

^{13}C NMR (CD_2Cl_2, δ, 25 °C): 147.9 (PzA3), 144.9 (d, J_{PC} = 2.5 Hz, PzB3), 142.8 (PzC3), 139.0 (PzC5), 138.8 (PzB5), 138.7 (PzA5), 121.3 (q, J_{FC} = 320.2 Hz, TfO-), 125.9-126.5 (m, C3), 108.9 (2C, PzB4/C4), 107.7 (PzA4), 95.2-95.9 (m, C4), 64.9-65.4 (m, C5), 43.9-44.3 (m, C2), 43.5-43.9 (m, C6), 36.1 (Ms), 13.4 (d, J_{PC} = 33.0 Hz, 3C, PMe_3).

WTp(NO)(PMe$_3$)(η^2-(*N*-mesyl)-2,2,3,4,5,6,6-heptadeutero-1,2,3,6-tetrahydropyridinium (OTf) (*d$_7$*-6)

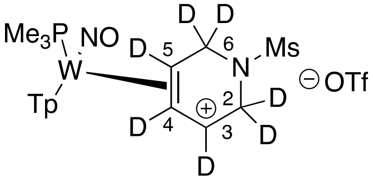

^{1}H NMR (CD_2Cl_2, δ, 25 °C): 8.35 (d, *J* -= 2.0 Hz, 1H, PzB3), 8.11 (d, *J* = 2.0 Hz, 1H, PzC3), 8.00 (d, *J* = 1.9 Hz, 1H, PzA3), 7.94 (d, *J* = 2.2 Hz, 1H, PzC5), 7.87 (d, *J* = 2.2 Hz, 1H, PzB5), 7.71 (d, *J* = 2.1 Hz, 1H, PzA5), 6.57 (t, *J* = 2.3 Hz, 1H, PzC4), 6.52 (t, *J* = 2.3 Hz, 1H, PzB4), 6.31 (t, *J* = 2.3, 1H, PzA4), 2.90 (s, 3H, Ms), 1.26 (d, J_{PH} = 9.7 Hz, 9H, PMe_3).

^{13}C NMR (CD_2Cl_2, δ, 25 °C): 148.0 (PzA3), 144.9 (PzB3), 142.7 (PzC3), 139.0 (PzC5), 138.8 (PzA5/B5), 138.7 (PzA5/B5), 126.2-126.7 (m, C3), 121.2 (q, J_{FC} = 320.8 Hz, -OTf), 109.0 (Pz4), 108.9 (Pz4), 107.7 (Pz4), 95.3-95.8 (m, C4), 64.8-65.3 (m, C5), 43.9-44.4 (m, C2), 43.5-43.8 (m, C6), 36.0 (Ms), 13.3 (d, J_{PC} = 32.7 Hz, 3C, PMe_3).

Synthesis and Characterization of [W] 1,2,3,6-THP Complexes

General Synthesis

Two separate solutions were prepared in two 30 mL glass screw-cap test tubes. Tube A was comprised of ***d_x*-6** (444 mg, 0.55 mmol) dissolved in EtCN (3 mL) and Tube B was a heterogeneous slurry of $NaBH^x_4$ (207 mg, 5.47 mmol) in dry THF (2 mL). After both tubes were chilled in a -50 °C toluene bath for 20 minutes, Tube B slurry was added to Tube A, and the tube sat for 16 hours. The solution was diluted with 150 mL of DCM and added to a separatory funnel. This solution was washed 3x with 200 mL of saturated aqueous $NaHCO_3$. The organic layer was isolated and set aside. The combined aqueous layers were combined and back-extracted with 50 mL of DCM to prevent loss of product. The organic layers were combined in a single flask and dried with anhydrous $MgSO_4$. This powder was then filtered off into a 60 mL coarse porosity fritted funnel and washed with DCM. The dried organic layers were then reduced *in vacuo* to dryness. The residue in the flask was redissolved in minimal DCM (approximately 10 mL). The solution was then slowly added to a stirring 200mL solution of 3:1 pentane/Et_2O. A tan precipitate formed immediately and was allowed to stir for ~10 minutes to ensure total precipitation. This powder was collected on a 60 mL medium porosity frit and washed 2x with 30 mL of pentane. This powder was dried in a desiccator under vacuum overnight.

Highly purified material can be obtained by performing the following extra steps. The complex was eluted off of a basic alumina column with 100% ethyl acetate. The fraction was reduced to dryness and redissolved in minimal DCM, which was then slowly added to a 50 mL solution of stirring pentane. A white precipitate was then formed and collected on a 15 mL F frit. This powder, after drying in a desiccator for 1 hour, was then added to a 4 dram vial containing 1 mL of d_6-acetone and was then stirred for 1 hour. The white powder was then collected in a 2 mL F frit and then dried in a desiccator overnight. (317 mg, 87% yield)

WTp(NO)(PMe_3)(η^2-(*N*-mesyl)-1,2,3,6-tetrahydropyridine) (7)

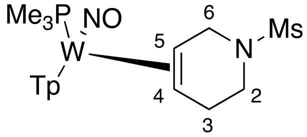

^{1}H NMR (CD_2Cl_2, δ, 25 °C): 8.09 (d, *J* = 1.8 Hz, 1H, PzA3), 8.03 (d, *J* = 1.9 Hz, 1H, PzB3), 7.79 (d, *J* = 2.4 Hz, 1H, PzB5), 7.72 (d, *J* = 2.2 Hz, 1H, PzC5), 7.67 (d, *J* = 2.4 Hz, 1H, PzA5), 7.21 (d, *J* = 2.0 Hz, 1H, PzC3), 6.36 (t, *J* = 2.2 Hz, 1H, PzB4), 6.27 (t, *J* = 2.2 Hz, 1H, PzA4), 6.19 (t, *J* = 2.2 Hz, 1H, PzC4), 4.31 (dd, *J* = 7.4, 12.6 Hz, 1H, H6), 3.77 (dd. *J* = 8.9, 12.6 Hz, 1H, H6), 3.49 (dt, *J* = 4.6, 10.7 Hz, 1H, H2), 3.25 (td, *J* = 4.9, 10.7 Hz, 1H, H^i), 2.85-2.91 (m, 1H, H5), 2.84 (s, 3H, Ms), 2.78-2.83 (m, 2H, H3), 1.27-1.30 (m, 1H, H4), 1.23 (d, J_{PH} = 8.2 Hz, 9H, PMe_3).

^{13}C NMR (CD_2Cl_2, δ, 25 °C): 144.0 (d, J_{PC} = 2.1 Hz, PzB3), 143.2 (PzA3), 140.8 (PzC3), 136.9 (PzC5), 136.5 (PzB5), 135.8 (PzA5), 106.8 (PzB4), 106.2 (PzC4), 106.0 (PzA4), 51.0 (2C, C4/C5), 48.9 (d, J_{PC} = 2.1 Hz, C6), 47.1 (C2), 35.7 (Ms), 28.7 (C3), 14.3 (d, J_{PC} = 28.1, 3C, PMe_3).

IR: ν(NO) = 1537 cm^{-1}
CV (DMA; 50 mV/s): E_{pa} = +0.49 V (NHE)
SC-XRD Data on S96

WTp(NO)(PMe$_3$)(η^2-(*N*-mesyl)-2-*anti*-deutero-1,2,3,6-tetrahydropyridine) (*Rel-(2S)-d$_1$*-7)

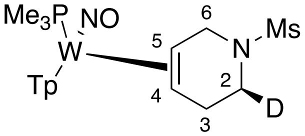

^{1}H NMR (CD_2Cl_2, δ, 25 °C): 8.09 (d, *J* = 1.8 Hz, 1H, PzA3), 8.03 (d, *J* = 1.8 Hz, 1H, PzB3), 7.79 (d, *J* = 2.2 Hz, 1H, PzB5), 7.72 (d, *J* = 2.2 Hz, 1H, PzC5), 7.68 (d, *J* = 2.2 Hz, PzA5), 7.21 (d, *J* = 1.8 Hz, 1H, PzC3), 6.36 (t, *J* = 2.1 Hz, 1H, PzB4), 6.26 (t, *J* = 2.1 Hz, 1H, PzA4), 6.19 (t, *J* = 2.2 Hz, 1H, PzC4), 4.32 (dd, *J* = 7.6, 12.5 Hz, 1H, H6), 3.78 (dd, *J* = 8.8, 12.5 Hz, 1H, H6), 3.46 (t, *J* = 4.4 Hz, 1H, H2), 2.86-2.91 (m, 1H, H5), 2.84 (s, 3H, Ms), 2.77-2.83 (m, 2H, H3), 1.26-1.30 (m, 1H, H4), 1.24 (d, J_{PH} = 8.3 Hz, 9H, PMe$_3$).

^{13}C NMR (CD_2Cl_2, δ, 25 °C): 144.0 (d, J_{PC} = 1.9 Hz, PzB3), 143.2 (PzA3), 140.8 (PzC3), 136.9 (PzC5), 136.5 (PzB5), 135.8 (PzA5), 106.8 (PzB4), 106.2 (PzC4), 106.0 (PzA4), 50.9 (d, J_{PC} = 12.7 Hz, C5), 50.9 (C4), 48.9 (d, J_{PC} = 2.2 Hz, C6), 46.7 (t, J_{CD} = 21.3 Hz, C2), 35.7 (Ms), 28.6 (C3), 14.3 (d, J_{PC} = 27.7 Hz, 9H, PMe$_3$).

WTp(NO)(PMe$_3$)(η^2-(*N*-mesyl)-3-*anti*-deutero-1,2,3,6-tetrahydropyridine) (*Rel-(3S)-d$_1$*-7)

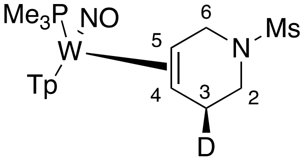

^{1}H NMR (CD_2Cl_2, δ, 25 °C): 8.09 (d, *J* = 1.9 Hz, 1H, PzA3), 8.03 (d, *J* = 1.8 Hz, 1H, PzB3), 7.79 (d, *J* = 2.2 Hz, 1H, PzB5), 7.72 (d, *J* = 2.2 Hz, 1H, PzC5), 7.67 (d, *J* = 2.2 Hz, 1H, PzA5), 7.21 (d, *J* = 1.8 Hz, 1H, PzC3), 6.36 (t, *J* = 2.1 Hz, 1H, PzB4), 6.26 (t, *J* = 2.1 Hz, 1H, PzA4),6.19 (t, *J* = 2.2 Hz, 1H, PzC4), 4.31 (dd, *J* = 7.5, 12.6 Hz, 1H, H6), 3.77 (dd, *J* = 8.9, 12.6 Hz, 1H, H6), 3.48 (dd, *J* = 4.8, 10.8 Hz, 1H, H2), 3.24 (t, *J* = 10.8 Hz, 1H, H2), 2.85-2.91 (m, 1H, H5), 2.84 (s, 3H, Ms), 2.75-2.80 (m, 1H, H3), 1.26-1.29 (m, 1H, H4), 1.24 (J_{PH} = 8.3 Hz, 9H, PMe$_3$).

^{13}C NMR (CD_2Cl_2, δ, 25 °C): 144.0 (d, J_{PC} = 2.0 Hz, PzB3), 143.2 (PzA3), 140.8 (PzC3), 136.9 (PzC5), 136.5 (PzB5), 135.8 (PzA5), 106.8 (PzB4), 106.2 (PzC4), 106.0 (PzA4), 51.0 (d, J_{PC} = 12.7 Hz, C5), 50.9 (C4), 48.9 (d, J_{PC} = 1.8 Hz, C6), 47.0 (C2), 35.7 (Ms), 28.4 (t, J_{DC} = 19.5 Hz, C3), 14.3 (d, J_{PC} = 28.2 Hz, 9H, PMe$_3$).

WTp(NO)(PMe$_3$)(η^2-(*N*-mesyl)-2,3-*anti*-dideutero-1,2,3,6-tetrahydropyridine) (*Rel-(2S,3S)-d$_2$*-7)

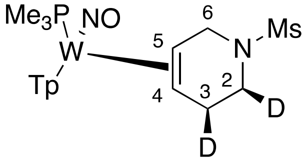

^{1}H NMR (CD_2Cl_2, δ, 25 °C): 8.09 (d, *J* = 2.0 Hz, 1H, PzA3), 8.03 (d, *J* = 2.0 Hz, 1H, PzB3), 7.79 (d, *J* = 2.3 Hz, 1H, PzB5), 7.72 (d, *J* = 2.2 Hz, 1H, PzC5), 7.67 (d, *J* = 2.3 Hz, 1H, PzA5), 7.21 (d, *J* = 2.0 Hz, 1H, PzC3), 6.36 (t, *J* = 2.2 Hz, 1H, PzB4), 6.26 (t, *J* = 2.2 Hz, 1H, PzA4), 6.19 (t, *J* = 2.2 Hz, 1H, PzC4), 4.31 (dd, *J* = 7.5, 12.6 Hz, 1H, H6), 3.77 (dd, *J* = 9.0, 12.6 Hz, 1H, H6), 3.46 (d, *J* = 4.5 Hz, 1H, H2), 2.85-2.90 (m, 1H, H5), 2.84 (s, 3H, Ms), 2.75-2.79 (m, 1H, H3), 1.27 (ddd, *J* = 1.1, 7.6, 9.9 Hz, 1H, H4), 1.24 (d, J_{PH} = 8.2 Hz, 9H, PMe$_3$).

^{13}C NMR (CD_2Cl_2, δ, 25 °C): 144.0 (d, J_{PC} = 2.1 Hz, PzB3), 143.2 (PzA3), 140.8 (PzC3), 136.9 (PzC5), 136.5 (PzB5), 135.8 (PzA5), 106.8 (PzB4), 106.2 (PzC4), 106.0 (PzA4), 51.0 (d, J_{PC} = 12.6 Hz, C5), 50.8 (C4), 48.9 (d, J_{PC} = 2.1 Hz, C6), 46.7 (t, J_{DC} = 21.6 Hz, C2), 35.7 (Ms), 28.4 (t, J_{DC} = 19.5 Hz, 1C, C3), 14.3 (d, J_{PC} = 28.2 Hz, 3C, PMe$_3$).

WTp(NO)(PMe$_3$)(η^2-(*N*-mesyl)-6-*syn*-2,3-*anti*-4,5-pentadeutero-1,2,3,6-tetrahydropyridine) (*Rel-(2R,3R,6R)-d$_5$*-7)

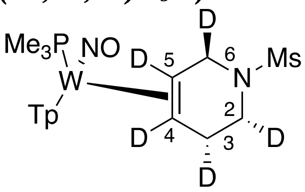

^{1}H NMR (CD$_2$Cl$_2$, δ, 25 °C): 8.09 (d, *J* = 1.9 Hz, 1H, PzA3), 8.03 (d, *J* = 2.0 Hz, 1H, PzB3), 7.79 (d, *J* = 2.2 Hz, 1H, PzB5), 7.72 (d, *J* = 2.2 Hz, 1H, PzC5), 7.68 (d, *J* = 2.2 Hz, 1H, PzA5), 7.21 (d, *J* = 1.8 Hz, 1H, PzC3), 6.36 (t, *J* = 2.1 Hz, 1H, PzB4), 6.26 (t, *J* = 2.1 Hz, 1H, PzA4), 6.20 (t, *J* = 2.1 Hz, 1H, PzC4), 3.76 (s, 1H, H6), 3.23 (d, *J* = 4.1 Hz, 1H, H2), 2.84 (s, 3H, Ms), 2.80 (d, *J* = 4.5 Hz, 1H, H3), 1.23 (d, J_{PH} = 8.2 Hz, 9H, PMe$_3$).

^{13}C NMR (CD$_2$Cl$_2$, δ, 25 °C): 144.0 (d, J_{PC} = 1.9 Hz, PzB3), 143.2 (PzA3), 140.7 (PzC3), 136.9 (PzC5), 136.5 (PzB5), 135.8 (PzA5), 106.8 (PzB4), 106.2 (PzC4), 106.0 (PzA4), 50.0-50.4 (m, 2C, C4/C5), 48.5 (t, J_{DC} = 20.8 Hz, C6), 46.4-46.7 (m, C2), 35.7 (Ms), 28.1 (t, J_{PC} = 19.5 Hz, C3), 14.3 (d, J_{PC} = 28.6 Hz, 3C, PMe$_3$).

WTp(NO)(PMe$_3$)(η^2-(*N*-mesyl)-3-*syn*-6-*anti*-2,2,4,5-hexadeutero-1,2,3,6-tetrahydropyridine) (*Rel-(3R,6R)-d$_6$*-7)

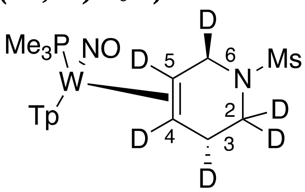

^{1}H NMR (CD$_2$Cl$_2$, δ, 25 °C): 8.09 (d, *J* = 1.8 Hz, 1H, PzA3), 8.03 (d, *J* = 1.9 Hz, 1H, PzB3), 7.79 (d, *J* = 2.2 Hz, 1H, PzB5), 7.72 (d, *J* = 2.2 Hz, 1H, PzC5), 7.67 (d, *J* = 2.2 Hz, 1H, PzA5), 7.20 (d, *J* = 1.9 Hz, 1H, PzC3), 6.36 (t, *J* = 2.1 Hz, 1H, PzB4), 6.26 (t, *J* = 2.1 Hz, 1H, PzA4), 6.19 (t, *J* = 2.1 Hz, 1H, PzC4), 3.75 (s, 1H, H6), 2.84 (s, 3H, Ms), 2.79 (s, 1H, H3), 1.23 (d, J_{PH} = 8.2 Hz, 9H, PMe$_3$).

^{13}C NMR (CD$_2$Cl$_2$, δ, 25 °C): 144.0 (d, J_{PC} = 1.5 Hz, PzB3), 143.2 (PzA3), 140.7 (PzC3), 136.9 (PzC5), 136.5 (PzB5), 135.8 (PzA5), 106.8 (PzB4), 106.2 (PzC4), 106.0 (PzA4), 50.0-50.5 (m, 2C, C4/C5), 48.5 (t, J_{DC} = 20.8 Hz, C6), 46.4-46.7 (m, C2), 35.7 (Ms), 27.8-28.2 (m, C3), 14.3 (d, J_{PC} = 27.9 Hz, 3C, PMe$_3$).

WTp(NO)(PMe$_3$)(η^2-(*N*-mesyl)-2-*syn*-6-*anti*-3,3,4,5-hexadeutero-1,2,3,6-tetrahydropyridine) (*Rel-(2R,6R)-d$_6$*-7)

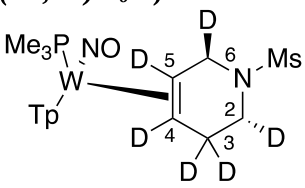

^{1}H NMR (CD$_2$Cl$_2$, δ, 25 °C): 8.09 (d, *J* = 1.9 Hz, 1H, PzA3), 8.03 (d, *J* = 2.0 Hz, 1H, PzB3), 7.79 (d, *J* = 2.3 Hz, 1H, PzB5), 7.72 (d, *J* = 2.2 Hz, 1H, PzC5), 7.68 (d, *J* = 2.2 Hz, 1H, PzA5), 7.20 (d, *J* = 1.9 Hz, 1H, PzC3), 6.36 (t, *J* = 2.1 Hz, 1H, PzB4), 6.26 (t, *J* = 2.1 Hz, 1H, PzA4), 6.19 (t, *J* = 2.2 Hz, 1H, PzC4), 3.75 (s, 1H, H6), 3.22 (s, 1H, H2), 2.84 (s, 3H, Ms), 1.24 (d, J_{PH} = 8.2 Hz, 9H, PMe$_3$).

^{13}C NMR (CD$_2$Cl$_2$, δ, 25 °C): 144.0 (d, J_{PC} = 1.5 Hz, PzB3), 143.2 (PzA3), 140.7 (PzC3), 136.9 (PzC5), 136.5 (PzB5), 135.8 (PzA5), 106.8 (PzB4), 106.2 (PzC4), 106.0 (PzA4), 49.9-50.4 (m, 2C, C4/C5), 48.5 (t, J_{DC} = 20.7 Hz, C6), 46.3-46.7 (m, C2), 35.7 (Ms), 27.5-28.2 (m, C3), 14.3 (d, J_{PC} = 28.5 Hz, 3C, PMe$_3$).

WTp(NO)(PMe$_3$)(η^2-(*N*-mesyl)-6-*anti*-2,2,3,3,4,5-heptadeutero-1,2,3,6-tetrahydropyridine) (*Rel-(6R)-d$_7$*-7)

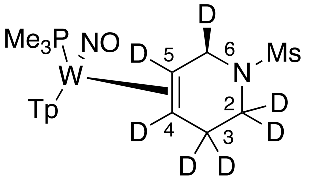

^{1}H NMR (CD_2Cl_2, δ, 25 °C): 8.09 (d, *J* = 1.9 Hz, 1H, PzA3), 8.03 (d, *J* = 1.8 Hz, 1H, PzB3), 7.80 (d, *J* = 2.2 Hz, 1H, PzB5), 7.72 (d, *J* = 2.2 Hz, 1H, PzPzC5), 7.68 (d, *J* = 2.2 Hz, 1H, PzA5), 7.21 (d, *J* = 1.9 Hz, 1H, PzC3), 6.36 (t, *J* = 2.1 Hz, 1H, PzB4), 6.26 (t, *J* = 2.2 Hz, 1H, PzA4), 6.20 (t, *J* = 2.2 Hz, PzC4), 3.76 (s, 1H, H6), 2.84 (s, 3H, Ms), 1.23 (d, J_{PH} = 8.2 Hz, 9H, PMe$_3$).

^{13}C NMR (CD_2Cl_2, δ, 25 °C): 144.0 (d, J_{PC} = 1.7 Hz, PzB3), 143.1 (PzA3), 140.7 (PzC3), 136.9 (PzC5), 136.5 (PzB5), 135.8 (PzA5), 106.8 (PzB4), 106.2 (PzC4), 106.0 (PzA4), 49.9-50.4 (m, 2C, C4/C5), 48.5 (t, J_{CD} = 20.6 Hz, C6), 45.9-46.7 (m, C2), 35.7 (Ms), 27.4-28.1 (m, C3), 14.2 (d, J_{PC} = 28.2 Hz, 3C, PMe$_3$).

WTp(NO)(PMe$_3$)(η^2-(*N*-mesyl)-3-*syn*-2,2,4,5,6,6-heptadeutero-1,2,3,6-tetrahydropyridine) (*Rel-(3R)-d$_7$*-7)

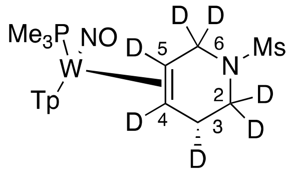

^{1}H NMR (CD_2Cl_2, δ, 25 °C): 8.10 (d, *J* = 1.9 Hz, 1H, PzA3), 8.04 (d, *J* = 1.9 Hz, 1H, PzB3), 7.80 (d, *J* = 2.3 Hz, 1H, PzB5), 7.73 (d, *J* = 2.3 Hz, 1H, PzC5), 7.68 (d, *J* = 2.3 HZ, 1H, PzA5), 7.21 (d, *J* = 1.9 Hz, 1H, PzC3), 6.36 (t, *J* = 2.2 Hz, 1H, PzB4), 6.26 (t, *J* = 2.2 Hz, 1H, PzA4), 6.20 (t, *J* = 2.2 Hz, 1H, PzC4), 2.85 (s, 3H, Ms), 2.80 (s, 1H, H3), 1.24 (d, J_{PH} = 8.3 Hz, 9H, PMe$_3$).

^{13}C NMR (CD_2Cl_2, δ, 25 °C): 144.0 (d, J_{PC} = 1.9 Hz, PzB3), 143.1 (PzA3), 140.7 (PzC3), 136.9 (PzC5), 136.5 (PzB5), 135.8 (PzA5), 106.8 (PzB4), 106.2 (PzC4), 106.0 (PzA4), 49.8-50.4 (m, 2C, C4/C5), 47.9-48.6 (C6), 45.9-46.4 (m, C2), 35.7 (s, 3H, Ms), 28.0 (t, J_{DC} = 19.5 Hz, C3), 14.2 (d, J_{PC} = 28.0 Hz, 3C, PMe$_3$).

WTp(NO)(PMe$_3$)(η^2-(*N*-mesyl)-2,2,3,3,4,5,6,6-octadeutero-1,2,3,6-tetrahydropyridine) (*d$_8$*-7)

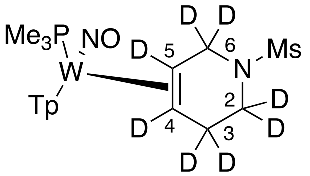

^{1}H NMR (CD_2Cl_2, δ, 25 °C): 8.09 (d, *J* = 1.6 Hz, PzA3), 8.03 (d, *J* = 1.7 Hz, PzB3), 7.80 (d, *J* = 2.2 Hz, PzB5), 7.73 (d, *J* = 2.1 Hz, PzC5), 7.68 (d, *J* = 2.3 Hz, PzA5), 6.36 (t, *J* = 2.2 Hz, PzB4), 6.26 (t, *J* = 2.2 Hz, PzA4), 6.20 (t, *J* = 2.2 Hz, PzC4), 2.84 (s, 3H, Ms), 1.24 (d, J_{PH} = 8.3 Hz, 9H, PMe$_3$).

^{13}C NMR (CD_2Cl_2, δ, 25 °C): 143.6 (d, J_{PC} = 1.7 Hz, PzB3), 142.8 (PzA3), 140.4 (PzC3), 136.6 (PzB5), 136.1 (PzC5), 135.4 (PzA5), 106.5 (PzB4), 105.9 (PzC4), 105.7 (PzA4), 49.5-50.0 (m, 2C, C4/C5), 47.6-48.3 (m,C6), 45.5-46.1 (m, C2), 35.4 (Ms), 27.0-27.5 (m, C3), 13.88 (d, J_{PC} = 27.9 Hz, 3C, PMe$_3$).

Synthesis and Characterization of *Rel*-1,2,3,6-THPs

General Synthesis

Acetone (3 mL), DDQ (200 mg, 0.88 mmol), and **d_7-7** (130 mg, 0.20 mmol) were added to a 4-dram vial containing a stir pea. The reaction was stirred in a fume hood for 4 hours. This solution was diluted with 30 mL of DCM and was washed 3x with 50 mL of saturated aqueous $NaHCO_3$. The organic layer was isolated and set aside. The combined aqueous layers were combined and back-extracted with 30 mL of DCM to prevent loss of product. The organic layers were combined in a single round-bottom flask to which 5 grams of basic alumina powder were added. The solution was then reduced off *in vacuo*, bringing the eluted organic into the basic alumina powder. This powder was then dry loaded onto an 8 gram Teledyne basic alumina column, upon which clean product was eluted off of with 40% EtoAc in hexanes. The tubes containing organic were combined and reduced *in vacuo*, yielding a colorless residue. (14 mg, 44% isolated yield)

(*N*-mesyl)-1,2,3,6-tetrahydropyridine (14)

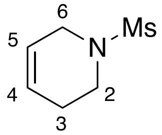

^{1}H NMR (CD_2Cl_2, δ, 25 °C): 5.84-5.88 (m, 1H, H4), 5.69-5.73 (m, 1H, H5), 3.71-3.74 (m, 2H, H6), 3.33 (dt, J = 1.2, 5.7 Hz, 2H, H2), 2.77 (s, 3H, Ms), 2.23-2.27 (m, 2H, H3).

^{13}C NMR (CD_2Cl_2, δ, 25 °C): 125.7 (C4), 123.4 (C5), 45.1 (C6), 42.8 (C2), 35.3 (Ms), 25.6 (C3).

SC-XRD Data on S97

(*N*-mesyl)-2-deutero-1,2,3,6-tetrahydropyridine (*Rel-(2S)-d_1*-14)

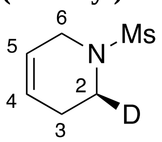

^{1}H NMR (CD_2Cl_2, δ, 25 °C): 5.85-5.89 (m, 1H, H4), 5.70-5.73 (m, 1H, H5), 3.72-3.74 (m, 2H, H6), 3.31 (tt, J = 1.7, 5.8 Hz, 1H, H2), 2.77 (s, 3H, Ms), 2.23-2.26 (m, 2H, H3).

^{13}C NMR (CD_2Cl_2, δ, 25 °C): 125.8 (C4), 123.4 (C5), 45.0 (C6), 42.5 (t, J_{DC} = 21.3 Hz, C2), 35.4 (Ms), 25.5 (C3).

(*N*-mesyl)-3-deutero-1,2,3,6-tetrahydropyridine (*Rel-(3S)-d_1*-14)

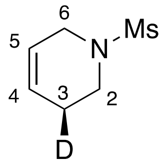

^{1}H NMR (CD_2Cl_2, δ, 25 °C): 5.85-5.89 (m, 1H, H4), 5.72 (dq, J = 3.2, 10.2 Hz, 1H, H5), 3.73 (q, J = 2.8 Hz, 2H, H6), 3.31-3.35 (m, 2H, H2), 2.78 (s, 3H, Ms), 2.22-2.26 (m, 1H, H3).

^{13}C NMR (CD_2Cl_2, δ, 25 °C): 125.7 (C4), 123.5 (C5), 45.1 (C6), 42.7 (C2), 35.3 (Ms), 25.3 (t, J_{DC} = 19.8 Hz, C3).

(*N*-mesyl)-2,3-*cis*-dideutero-1,2,3,6-tetrahydropyridine (*Rel-(2S,3S)-d*$_2$-14)

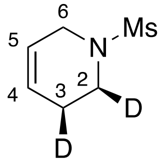

^{1}H NMR (CD_2Cl_2, δ, 25 °C): 5.85-5.88 (m, 1H, H5), 5.72 (dq, J = 3.0, 10.2 Hz, 1H, H4), 3.73 (s, 2H, H6), 3.30 (s, 1H, H2), 2.77 (s, 3H, Ms), 2.23 (s, 1H, H3).

^{13}C NMR (CD_2Cl_2, δ, 25 °C): 125.7 (C4), 123.5 (C5), 45.0 (C6), 42.4 (t, J_{DC} = 21.4 Hz, C2), 35.4 (Ms), 25.1 (t, J_{DC} = 19.8 Hz, C3).

(*N*-mesyl)-2,3-*cis*-6-*trans*-4,5-pentadeutero-1,2,3,6-tetrahydropyridine (*Rel-(2R,3R,6R)-d*$_5$-14)

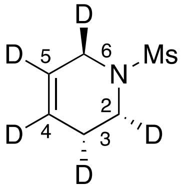

^{1}H NMR (CD_2Cl_2, δ, 25 °C): 3.69-3.71 (m, 1H, H6), 3.27-3.33 (m, 1H, H2), 2.77 (s, 3H, Ms), 2.22 (br s, 1H, H3).

^{13}C NMR (CD_2Cl_2, δ, 25 °C): 125.3 (t, J_{DC} = 24.6 Hz, C4), 123.0 (t, J_{DC} = 24.6 Hz, C5), 44.6 (t, J_{DC} = 21.4 Hz, C6), 42.4 (t, J_{DC} = 21.3 Hz, C2), 35.4 (Ms), 25.0 (t, J_{DC} = 19.9 Hz, C3).

(*N*-mesyl)-3,6-*trans*-2,2,4,5-hexadeutero-1,2,3,6-tetrahydropyridine (*Rel-(3R,6R)-d*$_6$-14)

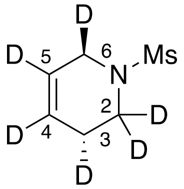

^{1}H NMR (CD_2Cl_2, δ, 25 °C): 3.69 (s, 1H, H3), 2.77 (s, 3H, Ms), 2.22 (s, 1H, H6).

^{13}C NMR (CD_2Cl_2, δ, 25 °C): 125.3 (t, J_{DC}= 24.6 Hz, C4), 123.0 (t, J_{DC} = 24.9 Hz, C5), 44.6 (t, J_{DC} = 21.3 Hz, C6), 41.8-42.5 (m, C2), 35.4 (Ms), 24.9 (t, J_{DC} = 19.9 Hz, C3).

(*N*-mesyl)-2,6-*trans*-3,3,4,5-hexadeutero-1,2,3,6-tetrahydropyridine (*Rel-(2R,6R)-d*$_6$-14)

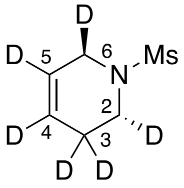

^{1}H NMR (CD_2Cl_2, δ, 25 °C): 3.70 (t, J = 2.4 Hz, 1H, H6), 3.31 (s, 1H, H2), 2.77 (s, 3H, Ms).

^{13}C NMR (CD_2Cl_2, δ, 25 °C): 125.3 (t, J_{DC} = 24.6 Hz, C4), 123.1 (t, J_{DC} = 24.6 Hz, C5), 44.6 (t, J_{DC} =21.3 Hz, C6), 42.3 (t, J_{DC} = 21.6 Hz, C2), 35.4 (Ms), 24.6 (q, J_{DC} = 19.9 Hz, C3).

(*N*-mesyl)-2,2,3,3,4,5,6-heptadeutero-1,2,3,6-tetrahydropyridine (*Rel-(6R)-d_7*-14)

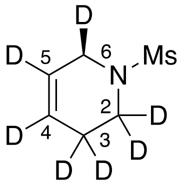

^{1}H NMR (CD_2Cl_2, δ, 25 °C): 3.70 (s, 1H, H6), 2.78 (s, 3H, Ms).

^{13}C NMR (CD_2Cl_2, δ, 25 °C): 125.2 (t, J_{DC} = 24.7 Hz, C4), 123.1 (t, J_{DC} = 24.8 Hz, C5), 44.6 (t, J_{DC} = 21.4 Hz, C6), 41.7-42.4 (m, C2), 35.4 (Ms), 24.3-25.0 (m, C3).

(*N*-mesyl)-2,2,3,4,5,6,6-heptadeutero-1,2,3,6-tetrahydropyridine (*Rel-(3R)-d_7*-14)

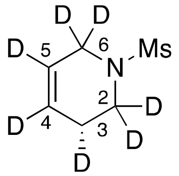

^{1}H NMR (CD_2Cl_2, δ, 25 °C): 2.77 (s, 3H, Ms), 2.21 (s, 1H, H3).

^{13}C NMR (CD_2Cl_2, δ, 25 °C): 125.4 (t, J_{DC} = 24.7 Hz, C4), 122.9 (t, J_{DC} = 24.7 Hz, C5), 44.1-44.7 (m, C6), 41.8-42.5 (m, C2), 35.4 (Ms), 24.9 (t, J_{DC} = 19.9 Hz, C3).

(*N*-mesyl)-2,2,3,3,4,5,6,6-octadeutero-1,2,3,6-tetrahydropyridine (*d_8*-14)

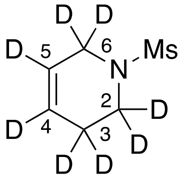

^{1}H NMR (CD_2Cl_2, δ, 25 °C): 2.77 (s, 3H, Ms).

^{13}C NMR (CD_2Cl_2, δ, 25 °C): 125.3 (t, J_{DC} = 24.8 Hz, C4), 123.0 (t, J_{DC} = 24.3 Hz, C5), 43.9-44.7 (m, C6), 41.7-42.4 (m, C2), 35.4 (Ms), 24.4-24.8 (m, C3).

Synthesis and Characterization of [W] THP ring-opened Complexes

WTp(NO)(PMe$_3$)(η^2-(*N*-mesyl)-1-amino-5-pentenylium)(OTf) (8)

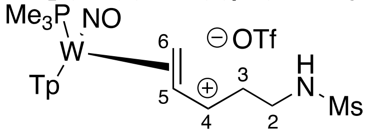

An oven-dried 4-dram vial and stir pea were set up on a stir plate. EtCN and **7** (60 mg, 0.09 mmol) were charged into the vial followed by DPhAT (34 mg, 0.11 mmol). The homogenous solution was stirred for 3 minutes, and then the reaction mixture was added to 50 mL of a stirring 4:1 Et_2O/hexanes solution. A tan precipitate was formed and collected on a 15 mL F frit. This was washed 2x with 10 mL of hexanes and dried in a desiccator. (60 mg, 82% yield)

^{1}H NMR (CD_2Cl_2, δ, 25 °C): 8.33 (d, *J* = 2.1 Hz, 1H, PzB3), 8.09 (d, *J* = 2.0 Hz, 1H, PzA3), 7.91 (d, *J* = 2.2 Hz, 1H, PzC5), 7.89 (d, *J* = 2.1 Hz, 1H, PzC3), 7.86 (d, *J* = 2.1 Hz, 1H, PzB5), 7.68 (d, *J* = 2.1 Hz, 1H, PzA5), 6.52 (t, *J* = 2.3 Hz, 1H, PzC4), 6.50 (t, *J* = 2.3 Hz, 1H, PzA4), 6.31 (t, *J* = 2.3 Hz, 1H, PzB4), 6.06-6.10 (m, 1H, H3), 5.87 (t, *J* = 6.0 Hz, 1H, *N*-H), 4.99 (dt, *J* = 8.4, 13.7 Hz, 1H, H4), 3.41-3.47 (m, 2H, H5/H1), 3.32-3.37 (m, 1H, H1), 3.11-3.16 (m, 1H, H2), 2.96 (s, 3H, Ms), 2.71 (ddd, *J* = 4.7, 8.9, 13.8 Hz, 1H, H5), 2.20-2.26 (m, 1H, H2), 1.26 (d, J_{PH} = 9.9 Hz, 9H, PMe_3).

^{13}C NMR (CD_2Cl_2, δ, 25 °C): 148.2 (PzA3), 145.6 (d, J_{PC} = 2.2 Hz, PzB3), 142.0 (PzC3), 138.9 (PzB5), 138.8 (PzC5), 138.5 (PzA5), 132.5 (C3), 121.2 (q, J_{FC} = 320.2 Hz, -OTf), 108.7 (PzB4), 108.4 (PzC4), 107.6 (PzA4), 105.1 (d, J_{PC} = 5.6 Hz, C4), 52.9 (d, J_{PC} = 12.9 Hz, C5), 45.0 (C2), 40.0 (Ms), 31.9 (C1), 13.6 (d, J_{PC} = 33.1 Hz, 3C, PMe_3).

IR: ν(NO) = 1634 cm^{-1}
CV (MeCN; 100 mV/s): $E_{1/2}$ = -801 mV, $E_{1/2}$ = -1507 mV, (NHE)
Unable to obtain EA, SC-XRD, or HRMS data for compound. Full 2D NMR provided below.

WTp(NO)(PMe$_3$)(η^2-(*N*-mesyl)-1-amino-5-pentene) (9)

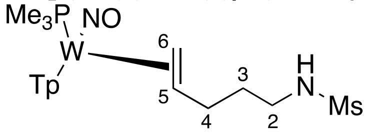

A test tube containing **8** (167 mg, 0.21 mmol) with EtCN (3 mL) was cooled in a toluene bath set to -50 °C. A separate tube was set up in the cold bath containing $NaBH_4$ (143 mg, 3.78 mmol) and dried THF (2 mL). Both tubes were chilled for 15 minutes, after which the $NaBH_4$ slurry was added to the solution of **8**. This solution was washed 3x with 200 mL of saturated aqueous $NaHCO_3$. The organic layer was isolated and set aside. The combined aqueous layers were combined and back-extracted with 50 mL of DCM to prevent loss of product. The organic layers were combined in a single flask and dried with anhydrous $MgSO_4$. This powder was then filtered off into a 60 mL coarse porosity fritted funnel and washed with DCM. The dried organic layers were then reduced *in vacuo* to dryness. The residue in the flask was redissolved in minimal DCM (approximately 10 mL). The solution was then slowly added to a stirring 50 mL solution of pentane. A tan precipitate formed immediately and was allowed to stir for ~10 minutes to ensure total precipitation. This powder was collected on a 60 mL medium porosity frit and washed 2x with 30 mL of pentane. This powder was dried in a desiccator under vacuum overnight.

^{1}H NMR (CD_2Cl_2, δ, 25 °C): 8.19 (d, *J* = 1.2 Hz, 1H, PzA3), 8.04 (d, *J* = 1.2 Hz, 1H, PzB3), 7.76 (d, *J* = 2.0 Hz, 1H, PzB5), 7.70 (d, *J* = 1.6 Hz, 1H, PzC5), 7.67 (d, *J* = 2.1 Hz, 1H, PzA5), 7.33 (d, *J* = 1.6 Hz, 1H, PzC3), 6.32 (t, *J* = 2.1 Hz, 1H, PzB4), 6.25 (t, *J* = 2.1 Hz, 1H, PzA4), 6.18 (t, *J* = 2.1 Hz, 1H, PzC4), 4.46-4.51 (b, 1H, NH), 3.20-3.27 (m, 2H, H2), 2.91 (s, 3H, Ms), 2.52-2.59 (m, 1H, H4), 1.78-1.84 (m, 2H, H3), 1.71-1.78 (m, 4H, H4/H6), 1.53-1.57 (m, 1H, H6), 1.28 (d, J_{PH} = 8.16 Hz, 9H, PMe_3), 1.05-1.10 (m, 1H, H5).

^{13}C NMR (CD_2Cl_2, δ, 25 °C): 144.4 (d, J_{PC} = 1.9 Hz, PzB3), 142.9 (PzA3), 141.1 (PzC3), 136.7 (PzC5), 136.3 (PzB5), 135.8 (PzA5), 106.3 (PzB4), 105.9 (PzA4), 105.8 (PzC4), 52.8 (C5), 44.1 (C2), 43.9 (d, J_{PC} = 10.7 Hz, C6), 40.3 (Ms), 37.4 (C3), 34.9 (C4), 14.2 (d, J_{PC} = 28.6 Hz, 3C, PMe_3).

IR: ν(NO) = 1532 cm^{-1}
CV, (MeCN; 100 mV/s): $E_{p,a}$ = +0.65 V
Unable to obtain EA, SC-XRD, or HRMS data for compound. Full 2D NMR provided below.

Synthesis and Characterization of [W] MPH 1,2-DHP Complexes

WTp(NO)(PMe$_3$)(η^2-(*N*-mesyl)-2-(methyl-α-phenylacetate)-1,2-dihydropyridine (11)

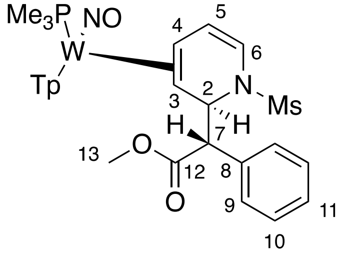

Previously described by Dabbs et al.[4] *(Ref) on page S7*

WTp(NO)(PMe$_3$)(η^2-(*N*-mesyl)-2-(methyl-α-phenylacetate)-2-*syn*-3,4,5,6-pentadeutero-1,2-dihydropyridine (d_5-11)

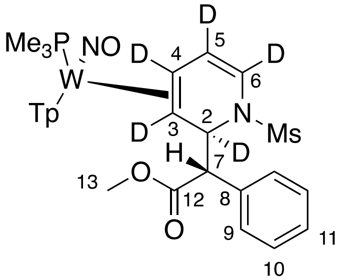

Methyl α-phenylbromoacetate (1.35 g, 5.89 mmol), ***d*₅-4D** (1.95 g, 2.39 mmol), and THF (10 mL) were charged to a flame-dried 50 mL round-bottom flask with a 1 inch stir bar. Zinc powder (684 mg, 10.46 mmol) was added to initiate the reaction, and the heterogenous solution was stirred for 1 hour. The zinc was removed by filtering the solution through a celite plug set up in a 60 mL coarse porosity frit with 1 inch of celite, which was then washed with residual DCM to prevent loss of product. The solution was diluted with 150 mL of DCM and added to a separatory funnel. This solution was washed 3x with 200 mL of saturated aqueous $NaHCO_3$. The organic layer was isolated and set aside. The combined aqueous layers were combined and back-extracted with 50 mL of DCM to prevent loss of product. The organic layers were combined in a single flask and dried with anhydrous $MgSO_4$. This powder was then filtered off into a 60 mL coarse porosity fritted funnel and washed with DCM. The dried organic layers were then reduced *in vacuo* to dryness. The residue in the flask was redissolved in minimal DCM (approximately 10 mL). The solution was then slowly added to 200mL of stirring pentane. A tan precipitate formed immediately and was allowed to stir for ~10 minutes to ensure total precipitation. This powder was collected on a 60 mL medium porosity frit and washed 2x with 30 mL of pentane. This powder was dried in a desiccator under vacuum for ~30 minutes. The dried powder was gently added to a 4-dram vial and stir pea filled with 15 mL of stirring MeOH and triturated overnight. The tan precipitate was collected on a 15 mL fine porosity frit and washed 2x with 10 mL of MeOH. The highly pure tan precipitate, after drying in a desiccator for 2 hours, was added to a 4-dram vial and stir pea filled with 15 mL of DME and triturated overnight. The highly enriched tan powder was collected on a 15 mL fine porosity frit and washed 1x with 10 mL of DME and 2x with 10 mL of diethyl ether. This powder was then dried in the desiccator. (700 mg, 36% yield)

^{1}H NMR (CD_2Cl_2, δ, 25 °C): 8.16 (d, J = 1.8 Hz, 1H, PzA3), 8.10 (d, J = 1.9 Hz, 1H, PzB3), 7.79 (d, J = 2.4 Hz, 1H, PzB5), 7.75 (d, J = 2.2 Hz, 1H, PzC5), 7.67 (d, J = 2.3 Hz, 1H, PzA5), 7.41 (d, J = 7.1 Hz, 2H, H9), 7.25 (t, J = 7.1 Hz, 2H, H10), 7.24 (d, J = 2.2 Hz, 1H, PzC3), 7.21 (tt, J = 1.3, 7.1 Hz, 1H, H11), 6.35 (t, J = 2.2 Hz, 1H, PzB4), 6.29 (t, J = 2.2 Hz, 1H, PzA4), 6.23 (t, J = 2.2 Hz, 1H, PzC4), 4.33 (s, 1H, H7), 3.42 (s, 3H, H13), 2.59 (Ms), 1.19 (d, J_{PC} = 8.5 Hz, 9H, PMe_3).

^{13}C NMR (CD_2Cl_2, δ, 25 °C): 173.5 (C12), 143.9 (PzA3), 142.8 (d, J_{PC} = 2.0 Hz, PzB3), 139.9 (PzC3), 137.6 (C8), 137.0 (PzC5), 136.4 (PzB5), 135.9 (PzA5), 130.1 (2C, C9), 128.3 (2C, C10), 127.7 (C11), 115.4 (t, J_{DC} = 26.4 Hz, C6), 114.2 (t, J_{DC} = 22.5 Hz, C5), 106.8 (PzB4), 106.4 (PzC4), 106.2 (PzA4), 63.3 (t, J_{DC} = 21.2 Hz, C3), 59.8 (C7), 58.7 (t, J_{DC} = 22.3 Hz, C2), 51.8 (C13), 43.5-43.8 (m, C4), 41.9 (Ms), 13.0 (d, J_{PC} = 28.3, 3C, PMe_3).

Synthesis and Characterization of [W] MPH 1,2,6-allyl Complexes

General Synthesis

An oven-dried 4-dram vial containing a stir pea was set up on a hot plate. To this vial, ***d_x*-11** (643 mg, 0.79 mmol) was added followed by 3 mL of EtCN. A separate oven-dried 4-dram vial was tared and filled with 1 mL of EtCN. To this was then added ^{x}HOTf (153 mg, 1.02 mmol), and the combined solution was then added to the ***d_x*-11**/EtCN solution, which was stirred until homogenous (about 1 minute). This solution was then added to a 150 mL stirring solution of Et_2O in a 300 mL Erlenmeyer flask with a 1" stir bar. A tan precipitate immediately formed, and the slurry was triturated for 10 minutes. The powder was then collected in a 30 mL F frit, washed 2x with 20 mL of Et_2O, and dried in a desiccator. (768 mg, 95% yield)

WTp(NO)(PMe_3)(η^2-(*N*-mesyl)-2-(methyl-α-phenylacetate)-1,2,3,6-tetrahydropyridinium (OTf) (12)

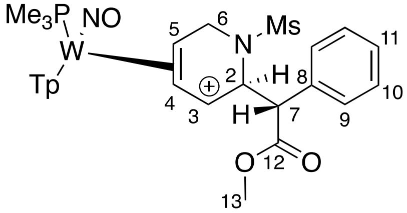

Previously described by Dabbs et al.[4] *on page S8*

WTp(NO)(PMe_3)(η^2-(*N*-mesyl)-2-(methyl-α-phenylacetate)-6-*syn*-deutero-1,2,3,6-tetrahydropyridinium (OTf) (d_1-12)

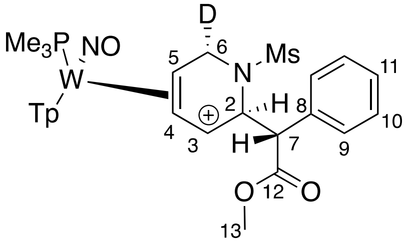

^1H NMR (CD_2Cl_2, δ, 25 °C): 8.31 (d, J = 2.2 Hz, 1H, PzB3), 8.11 (d, J = 2.2 Hz, 1H, PzC3), 7.90 (d, J = 2.3 Hz, 1H, PzC5), 7.80 (d, J = 2.3 Hz, 1H, PzB5), 7.59 (d, J = 2.3 Hz, 1H, PzA5), 7.53 (d, J = 7.4 H, 2H, H9), 7.46 (t, J = 7.8 Hz, 2H, H10), 7.42 (t, J = 7.4 Hz, 1H, H11), 6.86 (d, J = 2.2 Hz, 1H, PzA3), 6.58 (t, J = 2.4 Hz, 1H, PzC4), 6.47 (t, J = 2.4 Hz, 1H, PzB4), 5.98 (t, J = 2.4 Hz, 1H, PzA4), 5.89 (d, J = 7.9 Hz, 1H, H3), 5.46-5.48 (m, 1H, H2), 5.23 (t, J = 8.0 Hz, 1H, H4), 4.77-4.79 (m, 2H, H7, H6), 4.49-4.54 (m, 1H, H5), 3.84 (s, 3H, H13), 2.95 (s, 3H, Ms), 1.26 (d, J_{PH} = 9.7 Hz, 9H, PMe_3).

^{13}C NMR (CD_2Cl_2, δ, 25 °C): 171.4 (C12), 146.4 (PzA3), 144.5 (d, J_{PC} = 2.1 Hz, PzB3), 142.7 (PzC3), 138.9 (PzC5), 138.7 (PzA5/B5), 138.6 (PzA5/B5), 134.6 (C8), 129.7 (2C, C10), 129.0 (2C, C9), 128.9 (C11), 121.4 (q, J_{FC} = 321.7 Hz, TfO^-), 120.2 (C3), 109.1 (PzB4/C4), 108.9 (PzB4/C4), 107.3 (PzA4), 98.3 (d, J_{PC} = 3.0 Hz, C4), 65.1 (d, J_{PC} = 14.6 Hz, C5), 58.6 (C2), 55.9 (C7), 53.0 (C13), 42.7 (d, J_{DC} = 20.1 Hz, C6), 41.2 (Ms), 13.5 (d, J_{PC} = 32.9 Hz, 3C, PMe_3).

WTp(NO)(PMe$_3$)(η^2-(*N*-mesyl)-2-(methyl-α-phenylacetate)-2-*syn*-6-*anti*-3,4,5-pentadeutero-1,2,3,6-tetrahydropyridinium (OTf) (*d$_5$*-12)

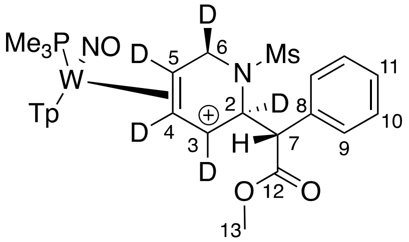

^{1}H NMR (CD_2Cl_2, δ, 25 °C): 8.30 (d, *J* = 2.1 Hz, 1H, PzB3), 8.13 (d, *J* = 2.1 Hz, 1H, PzC3), 7.91 (d, *J* = 2.3 Hz, 1H, PzC5), 7.83 (d, *J* = 2.2 Hz, 1H, PzB5), 7.61 (d, *J* = 2.3 Hz, 1H, PzA5), 7.54 (d, *J* = 7.6 Hz, 2H, H9), 7.46 (t, *J* = 7.6 Hz, 2H, H10), 7.42 (d, *J* = 7.4 Hz, 1H, H11), 6.84 (d, *J* = 2.1 Hz, 1H, PzA3), 6.57 (t, *J* = 2.3 Hz, 1H, PzC4), 6.48 (t, *J* = 2.3 Hz, 1H, PzB4), 5.98 (t, *J* = 2.3 Hz, 1H, PzA4), 4.78 (s, 1H, H7), 4.10 (s, 1H, H6^{syn}), 3.84 (s, 3H, H13), 2.96 (s, 3H, Ms), 1.27 (t, J_{PH} = 9.7 Hz, 9H, PMe$_3$).

^{13}C NMR (CD_2Cl_2, δ, 25 °C): 171.3 (C12), 146.3 (PzA3), 144.4 (d, J_{PC} = 2.2 Hz, PzB3), 142.7 (PzC3), 138.9 (PzC5), 138.7 (PzA5/B5), 138.6 (PzA5/B5), 134.6 (C8), 129.7 (2C, C10), 129.0 (2C, C9), 128.8 (C11), 121.4 (q, J_{FC} = 320 Hz, TfO$^-$), 118.9 (t, J_{DC} 23.5 Hz, C3), 109.0 (PzB4/C4), 108.9 (PzB4/C4), 107.3 (PzA4), 98.1 (t, J_{DC} = 25.7 Hz, C4), 64.6-65.0 (m, C5), 58.3 (t, J_{DC} = 22.4 Hz, C2), 55.8 (C7), 53.0 (C13), 42.6 (t, J_{DC} = 21.5 Hz, C6), 41.1 (Ms), 13.50 (d, J_{PC} = 32.9 Hz, 3C, PMe$_3$).

WTp(NO)(PMe$_3$)(η^2-(*N*-mesyl)-2-(methyl-α-phenylacetate)-2-*syn*-3,4,5,6,6-hexadeutero-1,2,3,6-tetrahydropyridinium (OTf) (*d$_6$*-12)

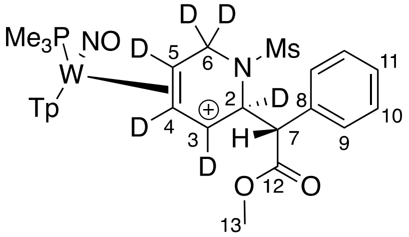

^{1}H NMR (CD_2Cl_2, δ, 25 °C): 8.30 (d, *J* = 2.1 Hz, 1H, PzB3), 8.13 (d, *J* = 2.2 Hz, 1H, PzC3), 7.91 (d, *J* = 2.2 Hz, 1H, PzC5), 7.82 (d, *J* = 2.3 Hz, 1H, PzB5), 7.60 (d, *J* = 2.3 Hz, 1H, PzA5), 7.54 (d, *J* = 7.4 Hz, 2H, H9), 7.46 (t, *J* = 7.6 Hz, 2H, H10), 7.42 (t, *J* = 7.3 Hz, 1H, H11), 6.84 (t, *J* = 2.1 Hz, 1H, PzA3), 6.57 (t, *J* = 2.3 Hz, 1H, PzC4), 6.47 (t, *J* = 2.4 Hz, 1H, PzB4), 5.98 (t, *J* = 2.4 Hz, 1H, PzA4), 4.77 (s, 1H, H7), 3.84 (s, 3H, H13), 2.96 (s, 3H, Ms), 1.27 (d, J_{PC} = 9.7 Hz, 9H, PMe$_3$).

^{13}C NMR (CD_2Cl_2, δ, 25 °C): 171.3 (C12), 146.4 (PzA3), 144.4 (d, J_{PC} = 2.4 Hz, PzB3), 142.8 (PzC3), 138.9 (PzC5), 138.7 (PzA5/B5), 138.6 (PzA5/B5), 134.6 (C8), 129.7 (2C, C10), 129.0 (2C C9), 128.8 (C11), 121.4 (q, J_{FC} = 320.8 Hz, TfO$^-$), 119.0 (t, J_{DC} = 26.3 Hz, C3), 109.1 (PzB4/C4), 108.9 (PzB4/C4), 107.3 (PzA4), 98.1 (t, J_{DC} = 25.7, C4), 64.5-64.9 (m, C5), 58.2 (t, J_{DC} = 22.2 Hz, C2), 55.9 (C7), 53.0 (C13), 42.1-42.5 (m, C6), 41.2 (Ms), 13.5 (d, J_{PC} = 32.9 Hz, 3C, PMe$_3$).

Synthesis and Characterization of [W] MPH 1,2,5,6-THP Complexes

General Synthesis

$NaCNB^{X}H_3$ (340 mg, 5.4 mmol) and dry THF (2.0 mL) were added to a screw-cap test tube that was placed in a cold bath set at -50 °C. To another tube was added ***d***$\boldsymbol{d_x}$**-12** (518 mg, 0.54 mmol) and propionitrile (3 mL). Both tubes were chilled for 15 minutes before the $NaCNB^{X}H_3$/THF solution was added to the solution of $\boldsymbol{d_x}$**-12**, which sat in the cold bath for 16 hours. This solution was diluted with 50 mL of DCM and was washed 3x with 50 mL of saturated aqueous $NaHCO_3$. The organic layer was isolated and set aside. The combined aqueous layers were combined and back-extracted with 50 mL of DCM to prevent loss of product. The organic layers were combined in a single flask and dried with anhydrous $MgSO_4$. This powder was then filtered off into a 60 mL coarse porosity fritted funnel and washed with DCM. The dried organic layers were then reduced *in vacuo* down to dryness. The residue in the flask was redissolved in minimal DCM (approximately 10 mL). The solution was then slowly added to 100 mL of stirring pentane. A light tan precipitate formed immediately and was allowed to stir for ~10 minutes to ensure total precipitation. This powder was collected on a 15 mL fine porosity frit and washed 2x with 10 mL of pentane. This powder was dried in a desiccator under vacuum overnight. (202 mg, 46% yield)

WTp(NO)(PMe$_3$)(η^2-(*N*-mesyl)-2-(methyl-α-phenylacetate)-1,2,5,6-tetrahydropyridine) (13)

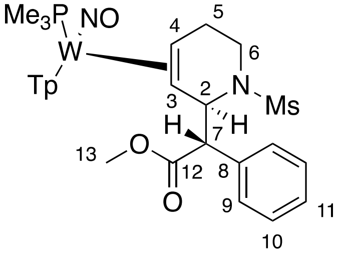

Previously described by Dabbs et al.[4] *on page S9*

WTp(NO)(PMe$_3$)(η^2-(*N*-mesyl)-2-(methyl-α-phenylacetate)-5-*anti*-deutero-1,2,5,6-tetrahydropyridine) (*Rel-(5R)-d$_1$*-13)

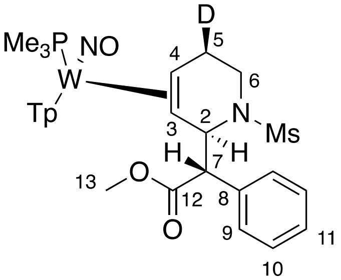

^{1}H NMR (CD_2Cl_2, δ, 25 °C): 8.31 (d, *J* = 1.8 Hz, 1H, PzA3), 8.04 (d, *J* = 1.8 Hz, 1H, PzB3), 7.75 (d, *J* = 2.3 Hz, 1H, PzC5), 7.73 (d, *J* = 2.3 Hz, 1H, PzB5), 7.69 (d, *J* = 2.3 Hz, 1H, PzA5), 7.38 (d, *J* = 7.1 Hz, 2H, H9), 7.24 (tt, *J* = 1.5, 7.1 Hz, 2H, H10), 7.21 (tt, *J* = 1.5, 7.1 Hz, 1H, H11), 7.20 (d, *J* = 1.6 Hz, 1H, PzC3), 6.32 (t, *J* = 2.2 Hz, 1H, PzB4), 6.31 (t, *J* = 2.2 Hz, 1H, PzA4), 6.24 (t, *J* = 2.2 Hz, 1H, PzC4), 5.54 (dd, *J* = 1.8, 8.3 Hz, 1H, H2), 4.07 (d, *J* = 8.3 Hz, H7), 3.39 (dd, *J* = 4.9, 13.1 Hz, 1H, H6), 3.06 (s, 3H, H13), 2.98 (dd, *J* = 11.8, 12.9 Hz, 1H, H6), 2.84 (ddd, *J* = 4.9, 10.4, 20.7 Hz, 1H, H4), 2.66 (dt, *J* = 5.0, 11.5 Hz, 1H, H5), 2.19 (s, 3H, Ms), 1.22 (d, J_{PH} = 8.3 Hz, 9H, PMe$_3$), 0.73 (d, *J* = 11.7 Hz, 1H, H3).

^{13}C NMR (CD_2Cl_2, δ, 25 °C): l72.7 (C12), 144.0 (PzA3), 143.2 (d, J_{PC} = 2.1 Hz, PzB3), 140.0 (PzC3), 137.7 (C8), 136.6 (PzB5/C5), 136.4 (PzB5/C5), 136.0 (PzA5), 130.1 (2C, C9), 128.6 (2C, C10), 127.9 (C11), 106.6 (PzA4/B4/C4), 106.4 (PzA4/B4/C4), 106.3 (PzA4/B4/C4), 62.8 (C7), 59.7 (C2), 54.3 (J_{WC} = 32.9 Hz, C3), 51.4 (C13), 46.0 (d, J_{PC} = 12.6 Hz, C4), 41.2 (C6), 40.6 (Ms), 27.9 (t, J_{DC} =18.6 Hz, C5), 13.8 (d, J_{PC} = 27.9 Hz, 3C, PMe$_3$).

WTp(NO)(PMe$_3$)(η^2-(*N*-mesyl)-2-(methyl-α-phenylacetate)-6-*syn*-deutero-1,2,5,6-tetrahydropyridine) (*Rel-(6S)-d$_1$*-13)

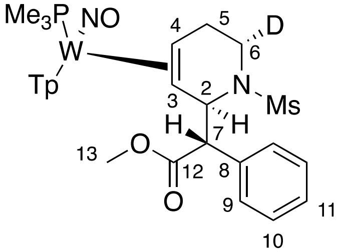

^{1}H NMR (CD_2Cl_2, δ, 25 °C): 8.33 (s, 1H, PzA3), 8.05 (d, *J* = 1.7 Hz, 1H, PzB3), 7.76 (d, *J* = 2.2 Hz, 1H, PzB5), 7.75 (d, *J* = 2.2 Hz, 1H, PzC5), 7.70 (d, *J* = 2.2 Hz, 1H, PzA5), 7.40 (d, *J* = 7.3 Hz, 2H, H9), 7.25 (t, *J* = 7.0 Hz, 2H, H10), 7.20-7.23 (m, 2H, H11 & PzC3), 6.33 (t, *J* = 2.1 Hz, 1H, PzB4), 6.31 (t, *J* = 2.1 Hz, 1H, PzA4), 6.25 (t, *J* = 2.1 Hz, 1H, PzC4), 5.56 (dd, *J* = 1.7, 8.3 Hz, 1H, H2), 4.09 (d, *J* = 8.2 Hz, 1H, H7), 3.08 (s, 3H, H13), 2.96-3.00 (m, 1H, H6), 2.82-2.89 (m, 1H, H4), 2.67-2.72 (m, 1H, H5), 2.51-2.55 (m, 1H, H5), 2.22 (s, 3H, Ms), 1.22 (d, J_{PH} = 8.3 Hz, 9H, PMe_3), 0.76 (d, *J* = 11.7 Hz, 1H, H3).

^{13}C NMR (CD_2Cl_2, δ, 25 °C): 172.6 (C12), 143.9 (PzA3), 143.1 (d, J_{PC} = 1.8 Hz, PzB3), 140.0 (PzC3), 137.6 (C8), 136.6 (PzB5/C5), 136.4 (PzB5/C5), 136.0 (PzA5), 130.1 (2C, C9), 128.5 (2C, C10), 127.8 (C11), 106.6 (PzA4/B4/C4), 106.4 (PzA4/B4/C4), 106.3 (PzA4/B4/C4), 62.7 (C7), 59.7 (C2), 54.3 (C3), 51.4 (C13), 46.0 (d, J_{PC} = 12.5 Hz, C4), 40.9 (t, J_{PC} = 20.5 Hz, C6), 40.5 (Ms), 28.2 (d, J_{PC} = 1.9 Hz, C5), 13.7 (d, J_{PC} = 27.8 Hz, 3C, PMe_3).

WTp(NO)(PMe$_3$)(η^2-(N-Mesyl)-2-(methyl-α-phenylacetate)-6-*syn*-5-*anti*-dideutero-1,2,5,6-tetrahydropyridine) (*Rel-(5R,6S)-d$_2$*-13)

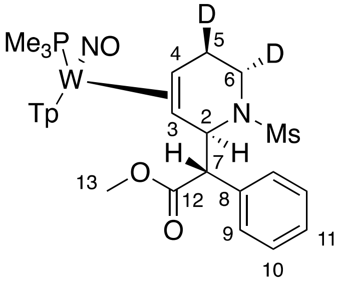

^{1}H NMR (CD_2Cl_2, δ, 25 °C): 8.32 (s, 1H, PzA3), 8.05 (s, 1H, PzB3), 7.76 (d, *J* = 2.2 Hz, 1H, PzB5), 7.74 (d, *J* = 2.2 Hz, 1H, PzC5), 7.69 (d, *J* = 2.2 Hz, 1H, PzA5), 7.40 (d, *J* = 7.2 Hz, 2H, C9), 7.25 (t, *J* = 7.6 Hz, 2H, C10), 7.20-7.23 (m, 2H, C11 & PzC3), 6.32 (t, *J* = 2.1 Hz, 1H, PzB4), 6.31 (t, *J* = 2.1 Hz, 1H, PzA4), 6.24 (t, *J* = 2.0 Hz, 1H, PzC4), 5.55 (dd, *J* = 1.5, 8.2 Hz, 1H, H2), 4.08 (d, *J* = 8.2 Hz, 1H, H7), 3.08 (H13), 2.95-3.00 (m, 1H, H6), 2.82-2.88 (m, 1H, H4), 2.65-2.69 (m, 1H, H5), 2.21 (s, 3H, Ms), 1.22 (d, J_{PH} =8.3 Hz, 9H, PMe_3), 0.75 (d, *J* = 11.7 Hz, 1H, H3).

^{13}C NMR (CD_2Cl_2, δ, 25 °C): 172.6 (C12), 143.9 (PzA3), 143.1 (d, J_{PC} = 2.2 Hz, PzB3), 140.0 (PzC3), 137.7 (C8), 136.6 (PzB5/C5), 136.4 (PzB5/C5), 136.0 (PzA5), 130.1 (2C, C9), 128.5 (2C, C10), 127.8 (C11), 106.6 (PzA4/B4/C4), 106.3 (PzA4/B4/C4), 106.2 (PzA4/B4/C4), 62.8 (C7), 59.7 (C2), 54.3 (C3), 51.4 (C13), 46.0 (d, J_{PC} = 12.5 Hz, C4), 40.9 (t, J_{DC} = 20.9 Hz, C6), 40.6 (Ms), 27.8 (t, J_{DC} = 18.3 Hz, C5), 13.7 (d, J_{PC} = 27.8 Hz, 3C, PMe_3).

WTp(NO)(PMe$_3$)(η^2-(*N*-mesyl)-2-(methyl-α-phenylacetate)-2,5-*syn*-6-*anti*-3,4-pentadeutero-1,2,5,6-tetrahydropyridine) (*Rel-(5S,6R)-d$_5$*-13)

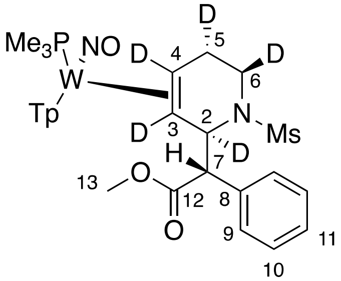

^{1}H NMR (CD$_2$Cl$_2$, δ, 25 °C): 8.31 (s, 1H, PzA3), 8.04 (s, 1H, PzB3), 7.75 (d, *J* = 1.9 Hz, 1H, PzB5), 7.74 (d, *J* = 1.9 Hz, 1H, PzC5), 7.69 (d, *J* = 1.9 Hz, 1H, PzA5), 7.38 (d, *J* = 7.4 Hz, 2H, H9), 7.24 (t, *J* = 7.4 Hz, 2H, H10), 7.21 (d, *J* = 7.4 Hz, 1H, H11), 7.19 (s, 1H, PzC3), 6.32 (t, *J* = 2.0 Hz, 1H, PzB4), 6.31 (t, *J* = 2.0 Hz, 1H, PzA4), 6.24 (t, *J* = 2.0 Hz, 1H, PzC4), 4.06 (s, 1H, H7), 3.37 (d, *J* = 2.2 Hz, 1H, H6), 3.05 (s, 3H, H13), 2.48 (d, *J* = 2.2 Hz, 1H, H5), 2.18 (s, 3H, Ms), 1.22 (d, J_{PH} = 8.3 Hz, 9H, PMe$_3$).

^{13}C NMR (CD$_2$Cl$_2$, δ, 25 °C): 172.7 (C12), 144.0 (PzA3), 143.2 (d, J_{PC} = 2.0 Hz, PzB3), 140.0 (PzC3), 137.7 (C8), 136.6 (PzB5/C5), 136.4 (PzB5/C5), 136.0 (PzA5), 130.1 (2C, C9), 128.6 (2C, C10), 127.9 (C11), 106.6 (PzA4/B4/C4), 106.4 (PzA4/B4/C4), 106.3 (PzA4/B4/C4), 62.7 (C7), 59.3 (t, J_{DC} = 21.9 Hz, C2), C3 buried under solvent peak, 51.4 (C13), 45.1-45.5 (m, C4), 40.8 (t, J_{DC} = 21.2 Hz, C6), 40.6 (Ms), 27.7 (t, J_{DC} = 19.6 Hz, C5), 13.8 (d, J_{PC} = 27.9 Hz, 3C, PMe$_3$).

WTp(NO)(PMe$_3$)(η^2-(*N*-mesyl)-2-(methyl-α-phenylacetate)-2-*syn*-6-*anti*-3,4,5,5-hexadeutero-1,2,5,6-tetrahydropyridine) (*Rel-(6R)-d$_6$*-13)

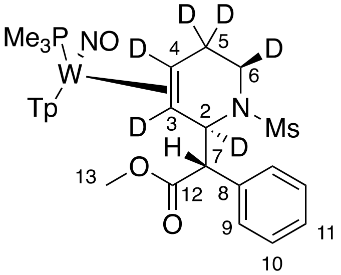

^{1}H NMR (CD$_2$Cl$_2$, δ, 25 °C): 8.31 (s, 1H, PzA3), 8.04 (s, 1H, PzB3), 7.75 (d, *J* = 1.9 Hz, 1H, PzB5), 7.73 (d, *J* = 1.9 Hz, 1H, PzC5), 7.69 (d, *J* = 1.9 Hz, 1H, PzA5), 7.38 (d, *J* = 7.4 Hz ,2H, H9), 7.24 (t, *J* = 7.4 Hz, 2H, H10), 7.21 (d, *J* = 7.3 Hz, 1H, H11), 7.19 (d, *J* = 1.6 Hz, 1H, PzC3), 6.32 (t, *J* = 2.1 Hz, 1H, PzB4), 6.31 (t, *J* = 2.0 Hz, 1H, PzA4), 6.24 (t, *J* = 2.0 Hz, 1H, PzC4), 4.05 (s, 1H, H7), 3.37 (s, 1H, H6), 3.05 (s, 3H, H13), 2.18 (s, 3H, Ms), 1.22 (d, J_{PH} = 8.2 Hz, 9H, PMe$_3$).

^{13}C NMR (CD$_2$Cl$_2$, δ, 25 °C): 172.7 (C12), 144.0 (PzA3), 143.2 (d, J_{PC} = 2.2 Hz, PzB3), 140.0 (PzC3), 137.7 (C8), 136.6 (PzB5/C5), 136.4 (PzB5/C5), 136.0 (PzA5), 130.1 (2C, C9), 128.6 (2C, C10), 127.9 (C11), 106.6 (PzA4/B4/C4), 106.4 (PzA4/B4/C4), 106.3 (PzA4/B4/C4), 62.7 (C7), 59.3 (t, J_{DC} = 21.4 Hz, C2), C3 buried under solvent peak, 51.4 (C13), 45.1-45.5 (m, C4), 40.7 (t, J_{DC} = 20.5 Hz, C6), 40.6 (Ms), 27.1-27.4 (m, C5), 13.8 (d, J_{PC} = 27.9 Hz, 3C, PMe$_3$).

WTp(NO)(PMe$_3$)(η^2-(*N*-mesyl)-2-(methyl-α-phenylacetate)-2,5-*syn*-3,4,6,6-hexadeutero-1,2,5,6-tetrahydropyridine) (*Rel-(5S)-d$_6$*-13)

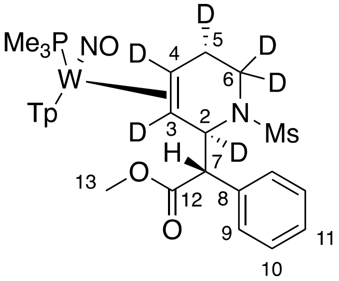

^{1}H NMR (CD_2Cl_2, δ, 25 °C): 8.41 (s, 1H, PzA3), 8.07 (s, 1H, PzB3), 7.79 (s, 1H, PzB5), 7.76 (s, 1H, PzC5), 7.75 (s, 1H, PzA5), 7.47 (d, *J* = 7.4 Hz, 2H, H9), 7.38 (t, *J* = 7.4, 2H, H10), 7.32 (t, *J* = 7.2 Hz, 1H, H11), 7.21 (s, 1H, PzC3), 6.42 (s, 1H, PzA4/B4/C4), 6.37 (s, 1H, PzA4/B4/C4), 6.27 (s, 1H, PzA4/B4/C4), 4.12 (s, 1H, H7), 2.99 (s, 3H, H13), 2.76 (s, 1H, H5), 1.64 (s, 3H, Ms), 1.26 (d, J_{PH} = 8.6 Hz, 9H, PMe$_3$).

^{13}C NMR (CD_2Cl_2, δ, 25 °C): 172.1 (C12), 144.5 (PzA3), 143.3 (PzB3), 140.3 (PzC3), 137.9 (C8), 137.2 (PzB5/C5), 136.9 (PzB5/C5), 136.7 (PzA5), 129.5 (2C, C9), 129.4 (2C, C10), 128.8 (C11), 107.4 (PzA4/B4/C4), 107.0 (PzA4/B4/C4), 106.7 (PzA4/B4/C4), 61.6 (C7), 58.5-58.8 (C2), 56.5-56.8 (m, C3), 51.8 (C13), 45.9-46.2 (m, C4), 39.5 (Ms), 38.8-39.1 (m, C6), 26.8-27.1 (m, C5), 13.6 (d, J_{PC} = 29.3 Hz, 3C, PMe$_3$).

WTp(NO)(PMe$_3$)(η^2-(*N*-mesyl)-2-(methyl-α-phenylacetate)-2-*syn*-3,4,5,5,6,6-heptadeutero-1,2,5,6-tetrahydropyridine) (*d$_7$*-13)

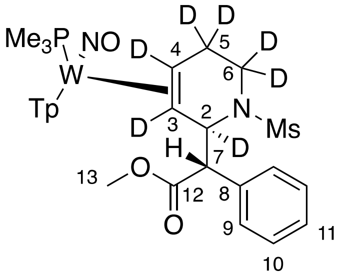

^{1}H NMR (CD_2Cl_2, δ, 25 °C): 8.35 (s, 1H, PzA3), 8.03 (s, 1H, PzB3), 7.75 (d, *J* = 2.1 Hz, 1H, PzB5), 7.73 (d, *J* = 1.8 Hz, 1H, PzC5), 7.70 (d, *J* = 1.8 Hz, 1H, PzA5), 7.40 (d, *J* = 7.5 Hz, 2H, H9), 7.27 (t, *J* = 7.5 Hz, 2H, H10), 7.22 (t, *J* = 7.2 Hz, 1H, H11), 7.16 (s, 1H, PzC3), 6.35 (s, 1H, PzB4), 6.32 (s, 1H, PzA4), 6.22 (s, 1H, PzC4), 4.01 (s, 1H, H7), 2.97 (s, 3H, H13), 2.02 (s, 3H, Ms), 1.20 (d, J_{PH} = 8.2 Hz, 9H, PMe$_3$).

^{13}C NMR (CD_2Cl_2, δ, 25 °C): 172.5 (C12), 143.9 (PzA3), 143.0 (d, J_{PC} = 1.6 Hz, PzB3), 139.7 (PzC3), 137.5 (C8), 136.4 (PzB5/C5), 136.3 (PzB5/C5), 135.9 (PzA5), 129.9 (2C, C9), 128.6 (2C, C10), 127.9 (C11), 106.5 (PzA4/B4/C4), 106.2 (2C, PzA4/B4/C4), 62.4 (C7), 58.9-59.1 (m, C2), C3 buried under solvent peak, 51.3 (C13), 44.6-44.9 (m, C4), 40.3 (Ms), 40.0-40.3 (m, C6), 26.7-27.3 (m, C5), 13.6 (d, J_{PC} = 27.9 Hz, 3C, PMe$_3$).

Synthesis and Characterization of MPH 1,2,5,6-THPs

General Synthesis

Acetone (3 mL), DDQ (170 mg, 0.75 mmol), and ***d$_x$*-13** (200 mg, 0.25 mmol) were added to a 4-dram vial containing a stir pea. The reaction was stirred in a fume hood for 4 hours. The solution was flushed through a 1 cm basic alumina plug set up in a 30 mL coarse porosity frit. This solution was diluted with 30 mL of DCM and was washed 3x with 50 mL of saturated aqueous $NaHCO_3$. The organic layer was isolated and set aside. The combined aqueous layers were combined and back-extracted with 30 mL of DCM to prevent loss of product. The organic layers were combined in a single round-bottom flask to which 5 grams of basic alumina powder were added. The solution was then reduced off *in vacuo* and then dry loaded onto a 8 gram Teledyne basic alumina column, upon which clean product was eluted off of with 40% ethyl acetate in hexanes. The tubes containing organic were combined and reduced *in vacuo*, yielding a white residue. (949 mg, 64% isolated yield)

***Erythro*-(*N*-mesyl)-2-(methyl-*α*-phenylacetate)-1,2,5,6-tetrahydropyridine (15)**

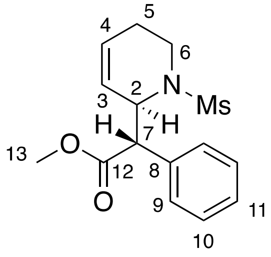

Previously described by Dabbs et al.[4] *on page S21*

***Erythro*-(*N*-mesyl)-2-(methyl-*α*-phenylacetate)-5-*cis*-deutero-1,2,5,6-tetrahydropyridine (*Rel-(5R)-d$_1$*-15)**

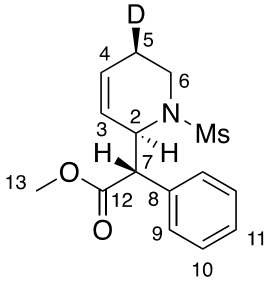

^{1}H NMR (d$_6$-acetone, δ, 25 °C): 7.45 (d, *J* = 7.6 Hz, 2H, H9), 7.36 (t, *J* = 7.6 Hz, 1H, H10), 7.31 (t, *J* = 7.6 Hz, 1H, H11), 5.96 (d, *J* = 10.5 Hz, 1H, H4), 5.87 (ddd, *J* = 2.9, 4.0, 10.5 Hz, 1H, H3), 4.90 (d, *J* = 10.5 Hz, 1H, H2), 3.89 (d, *J* = 10.7, Hz, 1H, H7), 3.68 (s, 3H, H13), 3.60 (dd, *J* = 6.5, 14.4 Hz, 1H, H6), 3.26 (dd, *J* = 12.0, 14.4 Hz, 1H, H6), 2.29-2.34 (m, 1H, H5), 2.06 (s, 3H, Ms).

^{13}C NMR (d$_6$-acetone, δ, 25 °C): 172.8 (C12), 137.6 (C8), 129.8. (2C, C9), 129.4 (2C, C10), 128.7 (C11), 128.4 (C4), 127.3 (C3), 56.5 (C7), 56.1 (C2), 52.5 (C13), 39.8 (Ms), 39.0 (C6), 24.4 (t, J_{DC} = 19.6 Hz, C5).

***Erythro*-(*N*-mesyl)-2-(methyl-*α*-phenylacetate)-6-*trans*-deutero-1,2,5,6-tetrahydropyridine (*Rel-(6S)-d₁*-15)**

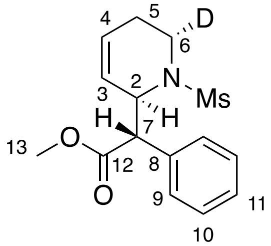

^{1}H NMR (d_6-acetone, δ, 25 °C): 7.45 (d, *J* = 7.2Hz, 2H, H9), 7.36 (tt, *J* = 1.4, 7.8 Hz, 2H, H10), 7.31 (tt, *J* = 1.4, 7.4 Hz, 1H, H11), 5.97 (ddt, *J* = 1.9, 5.5, 10.4 Hz, 1H, H4), 5.85-5.88 (m, 1H, H3), 4.90 (d, *J* = 10.7 Hz, 1H, H2), 3.89 (d, *J* = 10.7 Hz, 1H, H7), 3.68 (s, 3H, H13), 3.25 (d, *J* = 11.8 Hz, 1H, H6), 2.33 (ddq, *J* = 2.4, 11.8, 18.0 Hz, 1H, H5), 2.06 (s, 3H, Ms), 1.99 (dt, *J* = 4.6, 18.0 Hz, 1H, H5).

^{13}C NMR (d_6-acetone, δ, 25 °C): 172.8 (C12), 137.6 (C8), 129.8 (2C, C9), 129.4 (2C, C10), 128.7 (C11), 128.4 (C4), 127.3 (C3), 56.5 (C7), 56.1 (C2), 52.5 (C13), 39.8 (Ms), 38.7 (d, J_{DC} = 21.5 Hz, C6), 24.6 (C5).

***Erythro*-(*N*-mesyl)-2-(methyl-*α*-phenylacetate)-5-*cis*-6-*trans*-dideutero-1,2,5,6-tetrahydropyridine (*Rel-(5R,6S)-d₂*-15)**

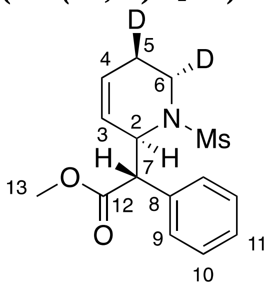

^{1}H NMR (d_6-acetone, δ, 25 °C): 7.45 (d, *J* = 7.3 Hz, 2H, H9), 7.36 (tt, *J* = 1.7, 7.8 Hz, 2H, H10), 7.31 (tt, *J* = 1.7, 7.4 Hz, 1H, H11), 5.96 (d, *J* = 10.4 Hz, 1H, H4), 5.87 (ddd, *J* = 2.7, 4.1, 10.4 Hz, 1H, H3), 4.90 (d, *J* = 10.7 Hz, 1H, H2), 3.88 (d, *J* = 10.7 Hz, 1H, H7), 3.67 (s, 3H, H13), 3.24 (d, *J* = 11.9 Hz, 1H, H6), 2.31 (d, *J* = 11.9 Hz, 1H, H5), 2.06 (s, 3H, Ms).

^{13}C NMR (d_6-acetone, δ, 25 °C): 172.8 (C12), 137.6 (C8), 129.8 (2C, C9), 129.4 (2C, C10), 128.7 (C11), 128.4 (C4), 127.3 (C3), 56.5 (C7), 56.0 (C2), 52.5 (C13), 39.8 (Ms), 38.7 (t, J_{DC} = 21.7, C6), 24.3 (t, J_{DC} = 19.6 Hz, C5).

***Erythro*-(*N*-mesyl)-2-(methyl-*α*-phenylacetate)-2,5-*trans*-6-*cis*-3,4-pentadeutero-1,2,5,6-tetrahydropyridine (*Rel-(5S,6R)-d₅*-15)**

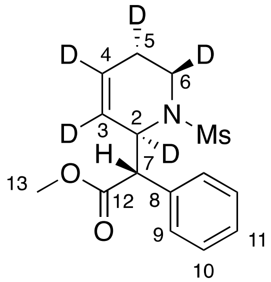

^{1}H NMR (d_6-acetone, δ, 25 °C): 7.45 (d, *J* = 7.5 Hz, 2H, H9), 7.36 (t, *J* = 7.3 Hz, 2H, H10), 7.31 (tt, *J* = 1.2, 7.3 Hz, 1H, H11), 3.88 (s, 1H, H7), 3.67 (s, 3H, H13), 3.58 (s, 1H, H6), 2.06 (s, 3H, Ms), 1.96 (s, 1H, H5).

^{13}C NMR (d_6-acetone, δ, 25 °C): 172.8 (C12), 137.6 (C8), 129.8 (2C, C9), 129.4 (2C, C10), 128.7 (C11), 128.0 (t, J_{DC} = 24.5 Hz, C4), 126.8 (t, J_{DC} = 24.6 Hz, C3), 56.4 (C7), 55.6 (t, J_{DC} = 22.0 Hz, C2), 52.5 (C13), 39.8 (Ms), 38.6 (t, J_{DC} = 21.4 Hz, C6), 24.0 (t, J_{DC} = 19.7 Hz, C5).

***Erythro*-(*N*-mesyl)-2-(methyl-*α*-phenylacetate)-6-*cis*-2-*trans*-3,4,5,5-hexadeutero-1,2,5,6-tetrahydropyridine (*Rel-(6R)-d*$_6$-15)**

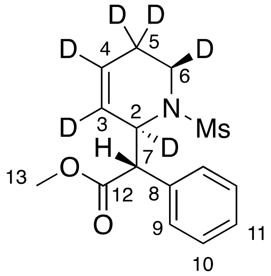

^{1}H NMR (d_6-acetone, δ, 25 °C): 7.45 (d, *J* = 7.5 Hz, 2H, H9), 7.36 (t, *J* = 7.8 Hz, 2H, H10), 7.31 (tt, *J* = 1.9, 7.3 Hz, 1H, H11), 3.88 (s, 1H, H7), 3.67 (s, 3H, H13), 3.57 (s, 1H, H6), 2.06 (s, 3H, Ms).
^{13}C NMR (d_6-acetone, δ, 25 °C): 172.8 (C12), 137.5 (C8), 129.7 (2C, C9), 129.4 (2C, C10), 128.7 (C11), 127.9 (t, J_{DC} = 24.6 Hz, C4), 126.9 (t, J_{DC} = 24.2 Hz, C3), 56.4 (C7), 55.6 (t, J_{DC} = 22.1 Hz, C2), 52.5 (C13), 39.8 (Ms), 38.5 (t, J_{DC} = 21.2 Hz, C6), 23.5-24.2 (m, C5).

***Erythro*-(*N*-mesyl)-2-(methyl-*α*-phenylacetate)-2,5-*trans*-3,4,6,6-hexadeutero-1,2,5,6-tetrahydropyridine (*Rel-(5S)-d*$_6$-15)**

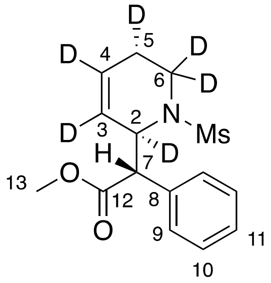

^{1}H NMR (d_6-acetone, δ, 25 °C): 7.45 (d, *J* = 7.2 Hz, 2H, H9), 7.36 (tt, *J* = 1.5 Hz, 2H, H10), 7.31 (tt, *J* = 1.3 Hz, 1H, H11), 3.88 (s, 1H, H7), 3.67 (s, 3H, H13), 2.06 (s, 3H, Ms), 1.96 (s, 1H, H5).

^{13}C NMR (d_6-acetone, δ, 25 °C): 172.8 (C12), 137.6 (C8), 129.8 (2C, C9), 129.4 (2C, C10), 128.7 (C11), 128.0 (t, J_{DC} = 24.7 Hz, C4), 126.9 (t, J_{DC} = 24.8 Hz, C3), 56.4 (C7), 55.6 (t, J_{DC} = 21.9 Hz, C2), 52.5 (C13), 39.8 (Ms), 38.1-38.7 (m, C6), 23.9 (t, J_{DC} = 19.8 Hz, C5).

***Erythro*-(*N*-mesyl)-2-(methyl-*α*-phenylacetate)-2,3,4,5,5,6,6-heptadeutero-1,2,5,6-tetrahydropyridine (*d*$_7$-15)**

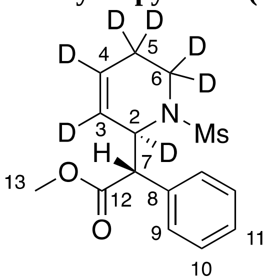

^{1}H NMR (d_6-acetone, δ, 25 °C): 7.45 (d, *J* = 7.4 Hz, 2H, H9), 7.36 (t, *J* = 7.3 Hz, 2H, H10), 7.31 (t, *J* = 7.3 Hz, 1H, H11), 3.88 (s, 1H, H7), 3.67 (s, 3H, H13), 2.06 (s, 3H, Ms).

^{13}C NMR (d_6-acetone, δ, 25 °C): 172.8 (C12), 137.6 (C8), 129.8 (2C, C9), 129.4 (2C, C10), 128.7 (C11), 127.9 (t, J_{DC} = 24.3 Hz, C4), 126.9 (t, J_{DC} = 24.6 Hz, C3), 56.5 (C7), 55.61 (t, J_{DC} = 22.2 Hz, C2), 52.5 (C13), 39.8 (Ms), 38.1-38.7 (m, C6), 23.4-24.0 (m, C5).

Synthesis and Characterization of Hydrogenated Piperidines

(*N*-mesyl)-piperidine (16)

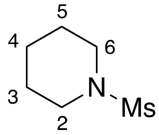

THP **14** (57 mg, 35 mmol) was dissolved in methanol (3 mL) and added to a 4-dram vial. This solution was then circulated through a ThalesNano H-Cube flow hydrogenator for 5 hours. The temperature was set to 50 °C, the H_2 pressure to 25 bars, and the catalyst cartridge used contained 5% Pd on carbon. After circulation, the solution was collected and reduced to dryness (38 mg 66% yield).

^{1}H NMR (d_6-CD_2Cl_2, δ, 25 °C): 3.14 (t, *J* = 5.4 Hz, 4H, H2), 2.71 (s, 3H, Ms), 1.66 (q , *J* = 5.6 Hz, 4H, H3), 1.53-1.57 (m, 2H, H4).

^{13}C NMR (d_6-CD_2Cl_2, δ, 25 °C): 46.8 (2C, C2), 34.0 (Ms), 25.4 (2C, C3), 23.7 (C4).

SC-XRD Data on S98

***erythro*-(*N*-mesyl)-methylphenidate (17)**

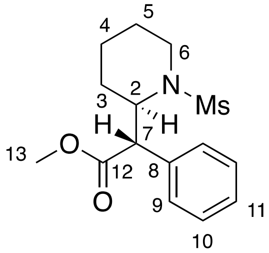

Previously described by Dabbs et al.[4] *on page S28*

NMR SPECTROSCOPY

Supplementary Figure 1: ^{1}H NMR, 800 MHz, CD_2Cl_2, 25 °C, *d*$_5$-pyridine borane (**10**)

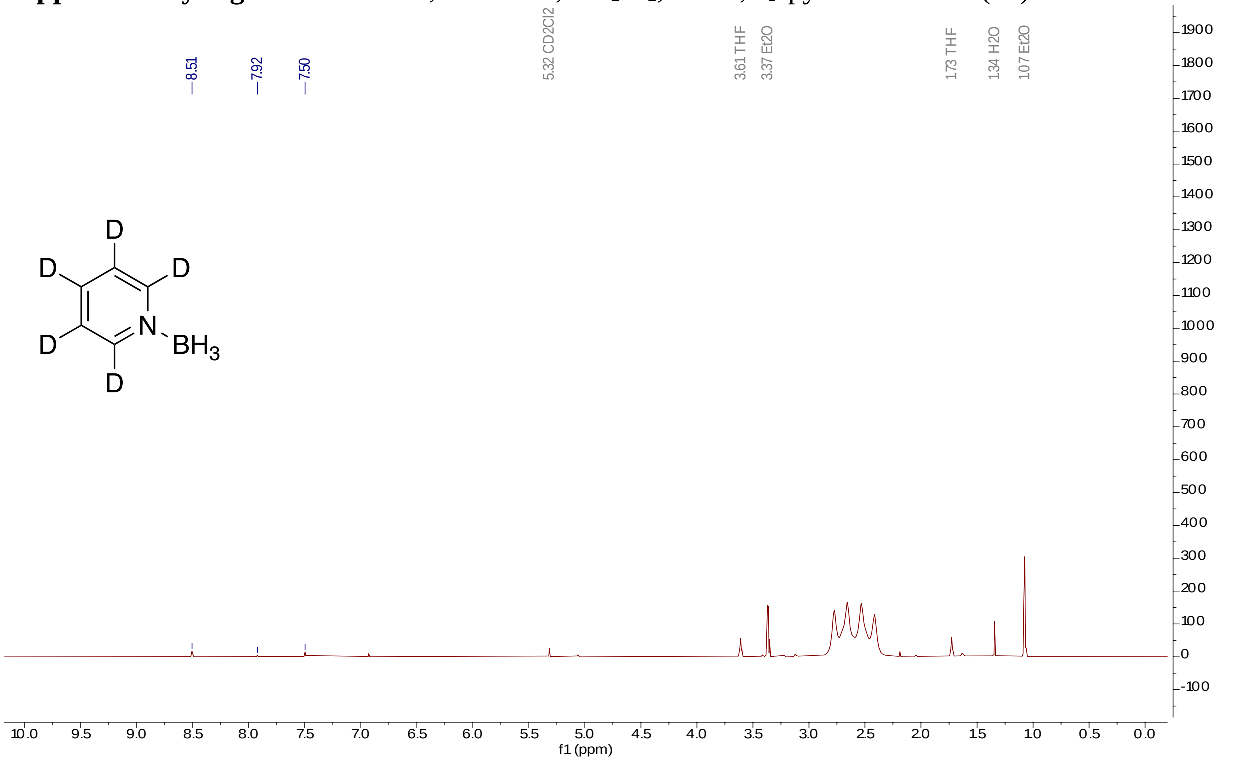

Supplementary Figure 2: ^{13}C NMR, 200 MHz, CD_2Cl_2, 25 °C, *d*$_5$-pyridine borane (**10**)

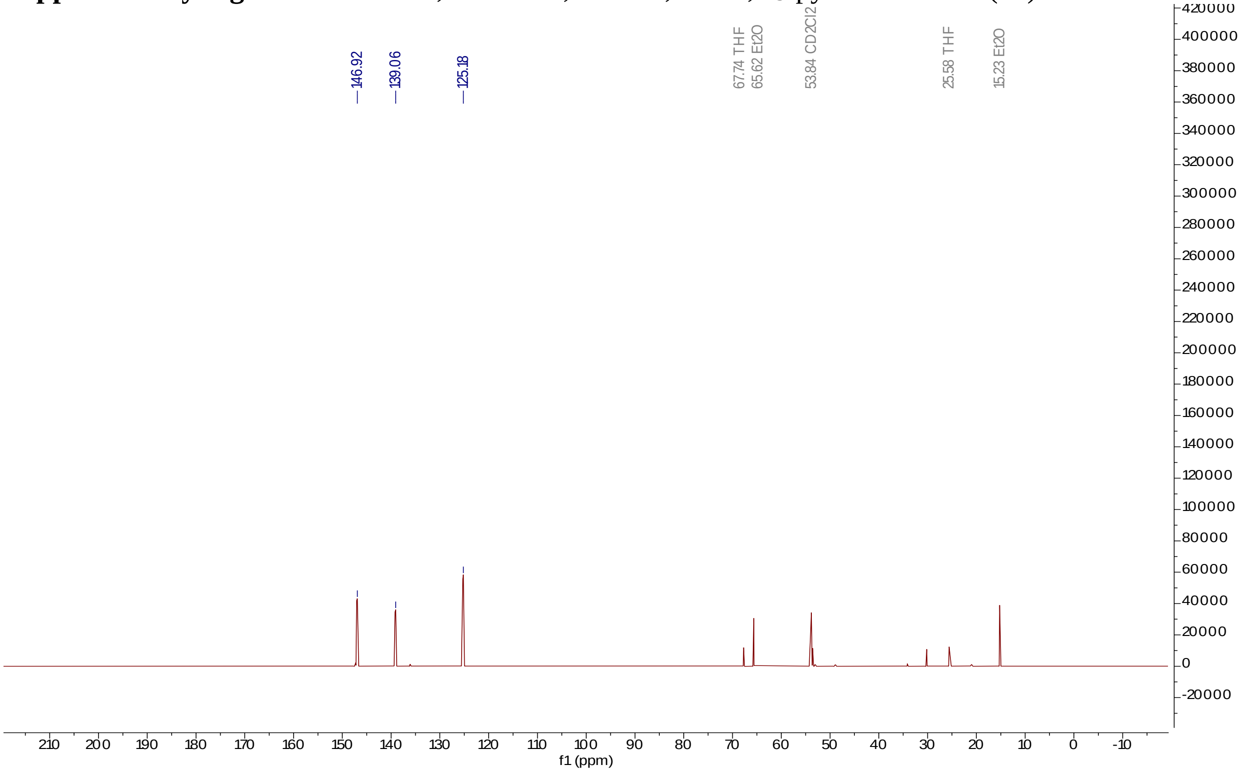

Supplementary Figure 3: ^{1}H NMR, 800 MHz, CD_2Cl_2, 25 °C, ***d*₅-2**

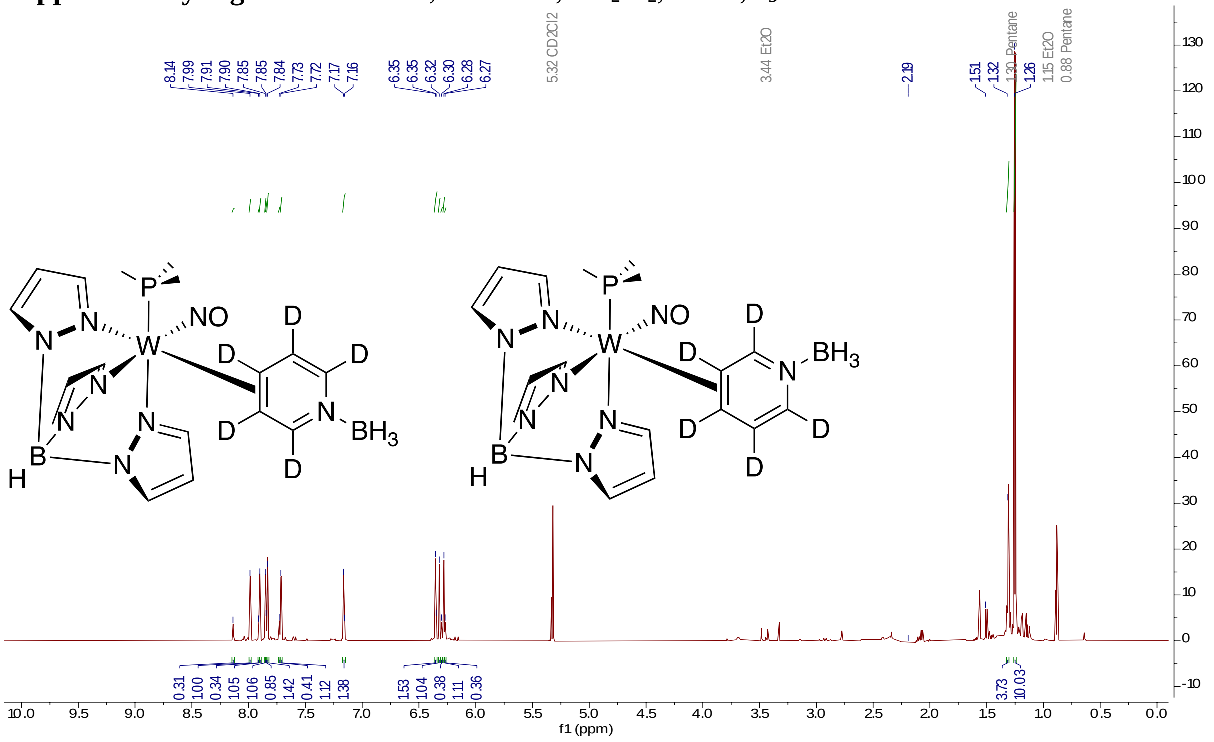

Supplementary Figure 4: ^{13}C NMR, 200 MHz, CD_2Cl_2, 25 °C, ***d*₅-2**

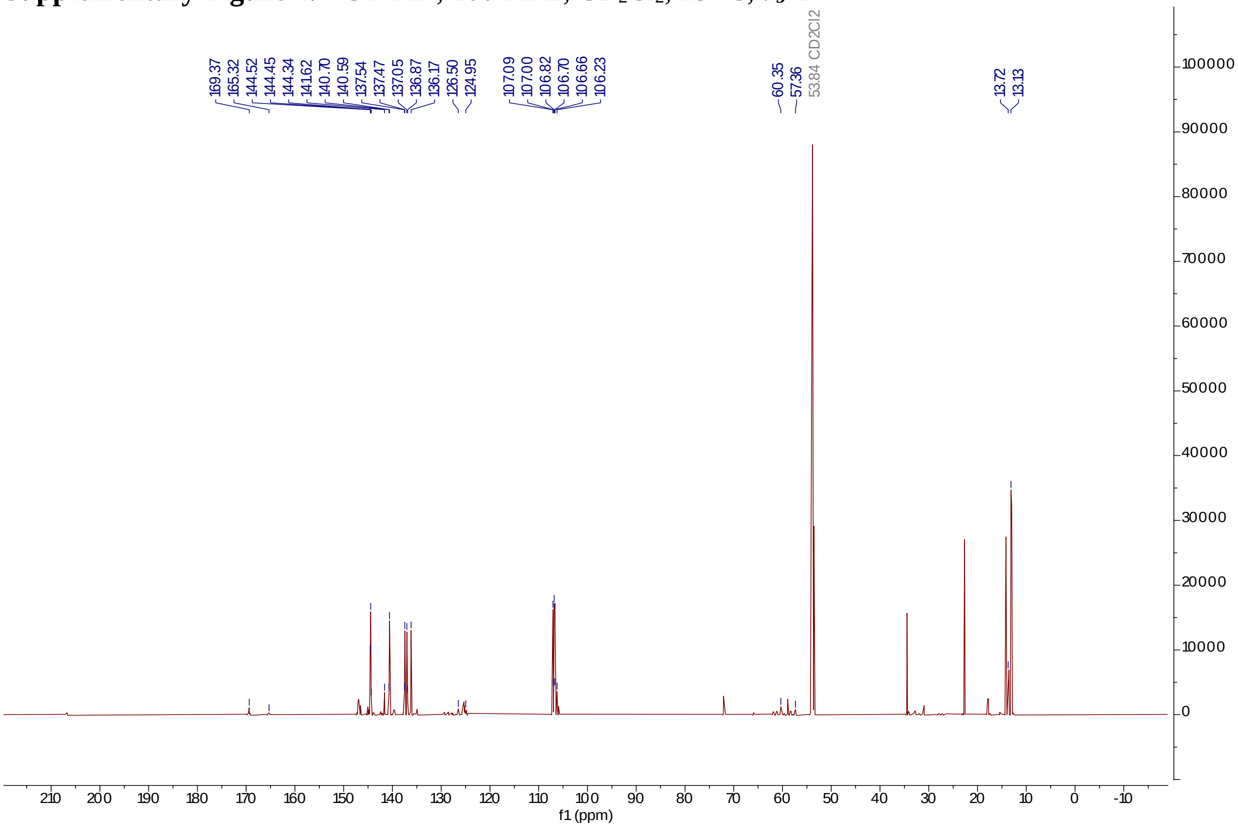

Supplementary Figure 5: ^{1}H NMR, 800 MHz, CD_2Cl_2, 25 °C, ***d*$_5$-3**

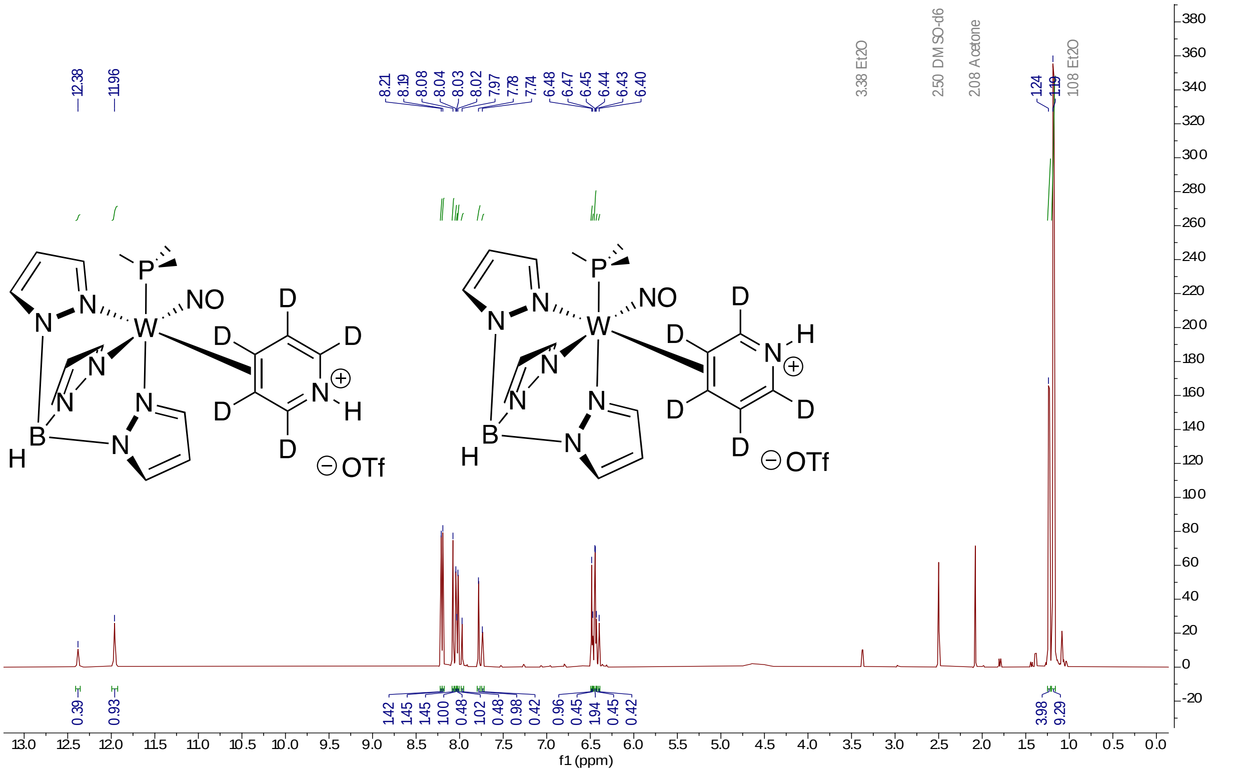

Supplementary Figure 6: ^{13}C NMR, 200 MHz, CD_2Cl_2, 25 °C, ***d*$_5$-3**

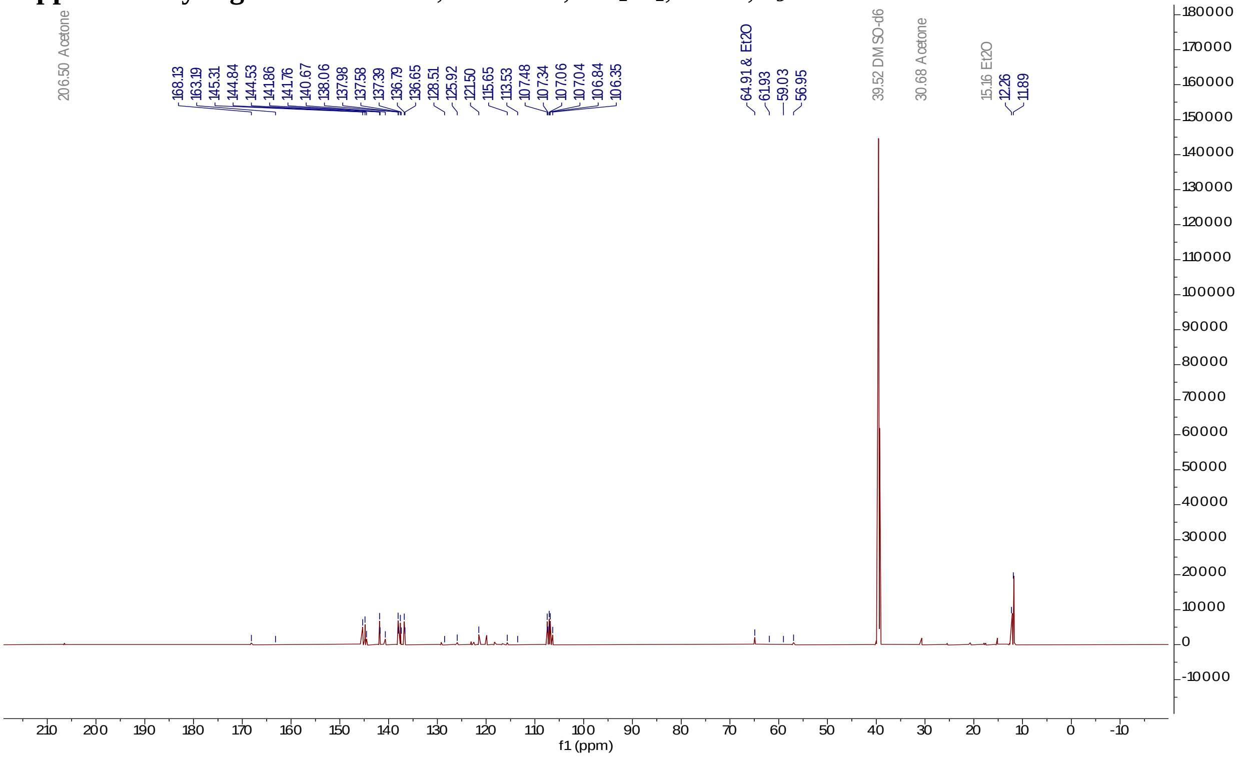

Supplementary Figure 7: ^{1}H NMR, 800 MHz, CD_2Cl_2, 25 °C, ***d*$_5$-4D**

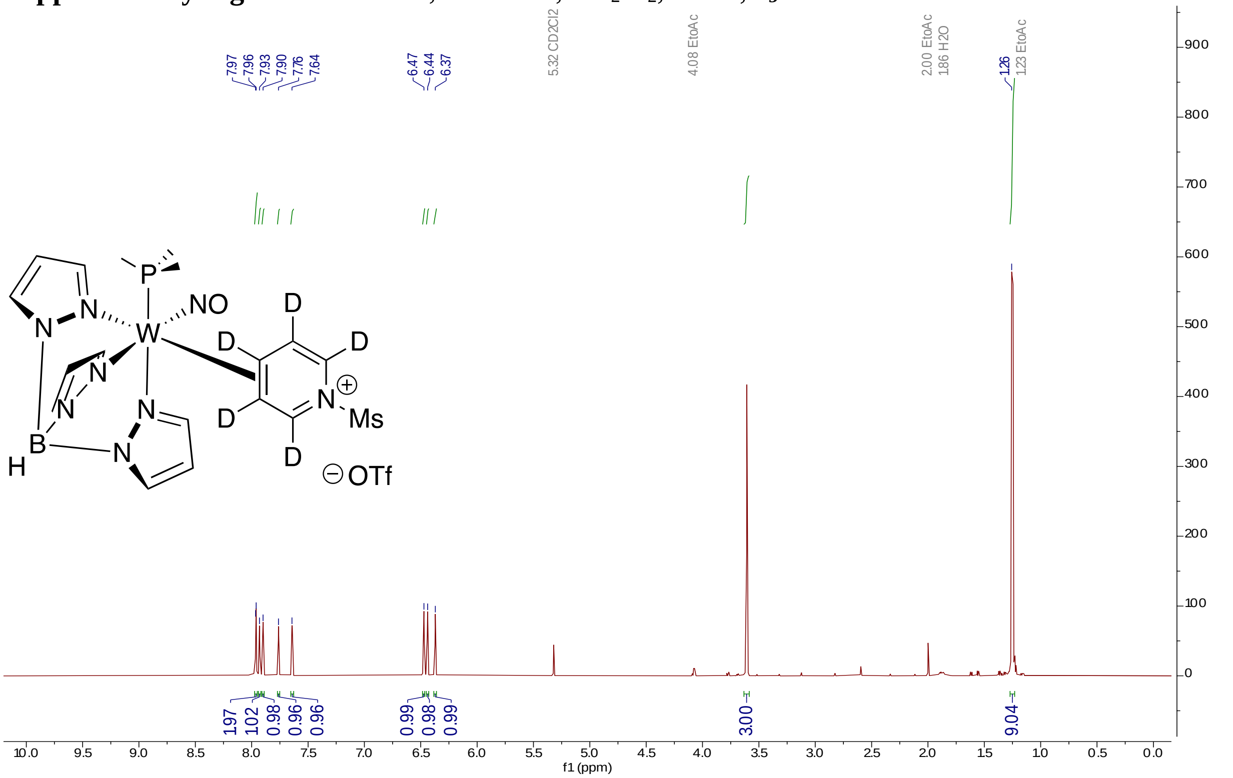

Supplementary Figure 8: ^{13}C NMR, 200 MHz, CD_2Cl_2, 25 °C, ***d*$_5$-4D**

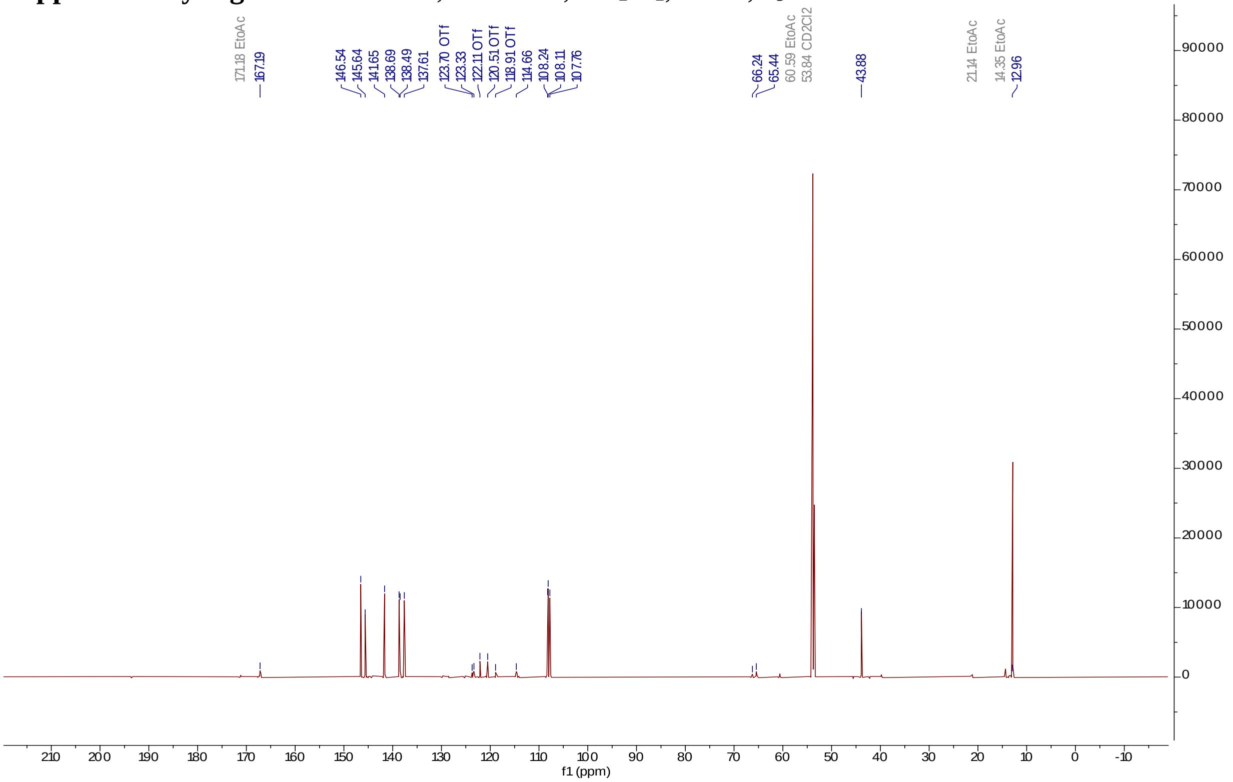

Supplementary Figure 9: ^{1}H NMR, 800 MHz, CD_2Cl_2, 25 °C, **5**

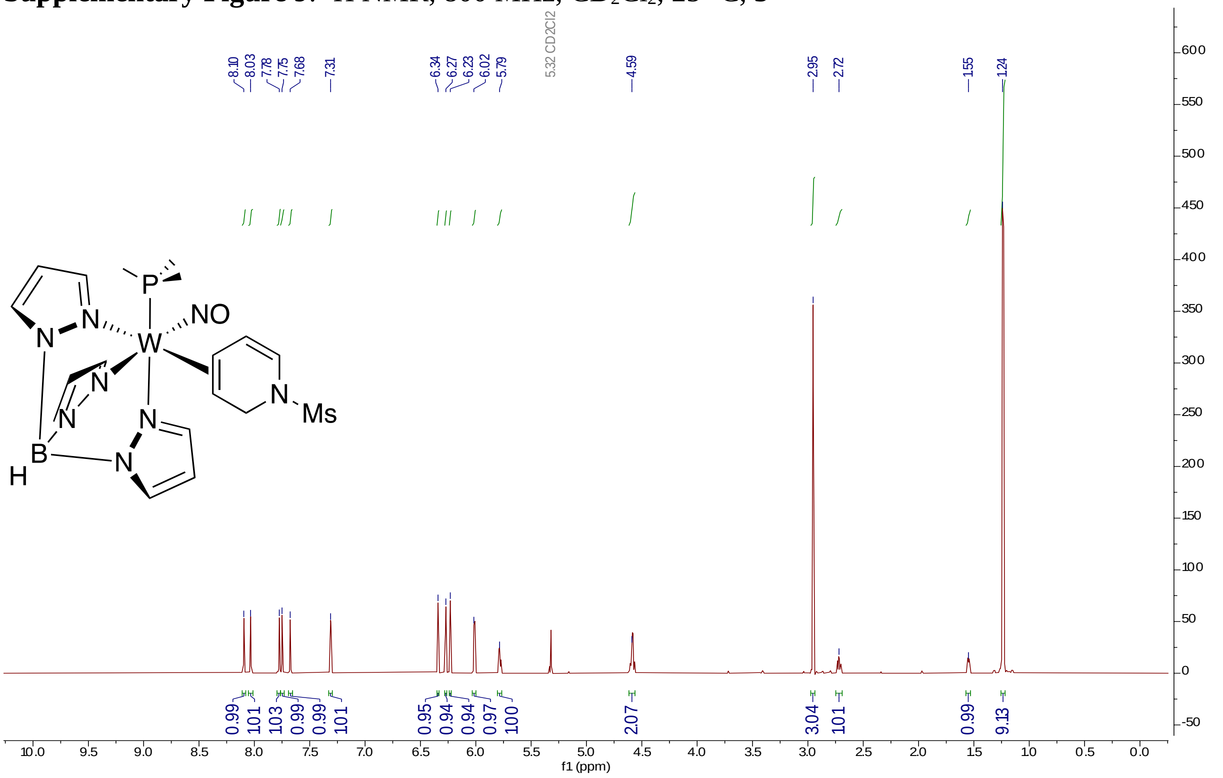

Supplementary Figure 10: ^{13}C NMR, 200 MHz, CD_2Cl_2, 25 °C, **5**

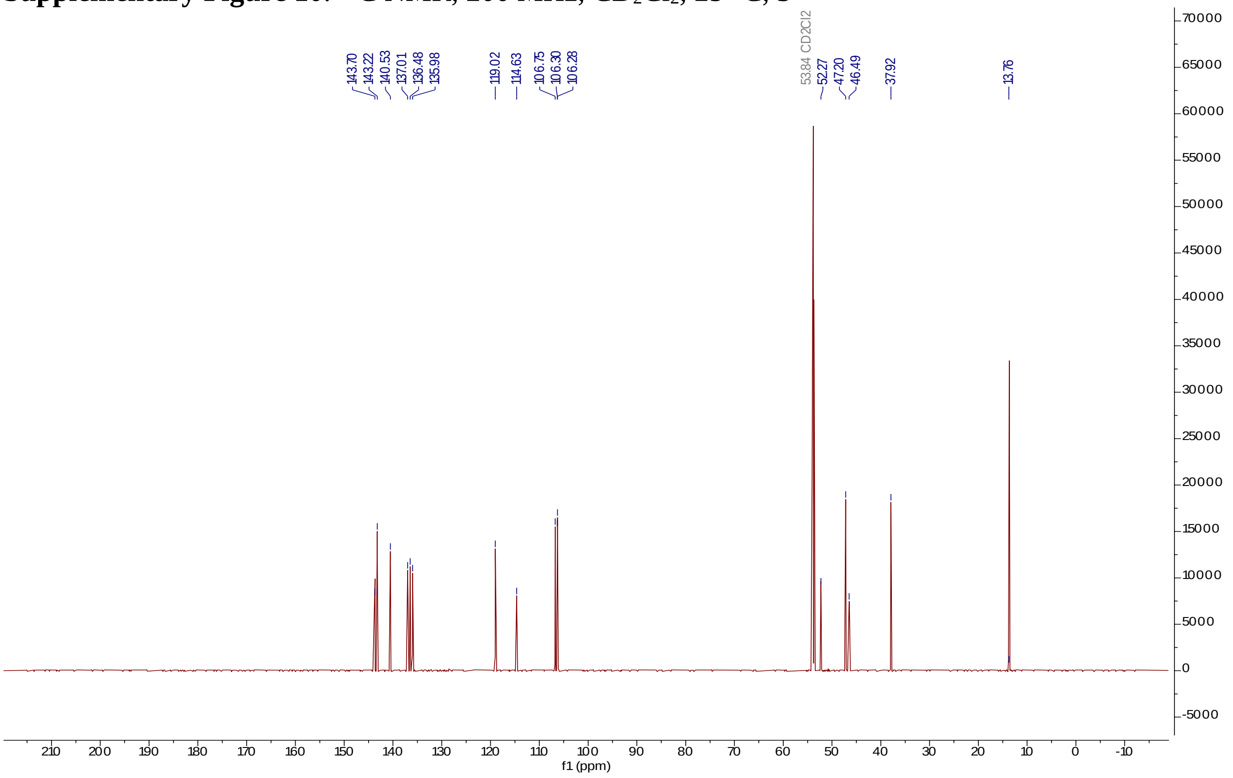

Supplementary Figure 11: ^{1}H NMR, 800 MHz, CD_2Cl_2, 25 °C, ***d*₁-5**

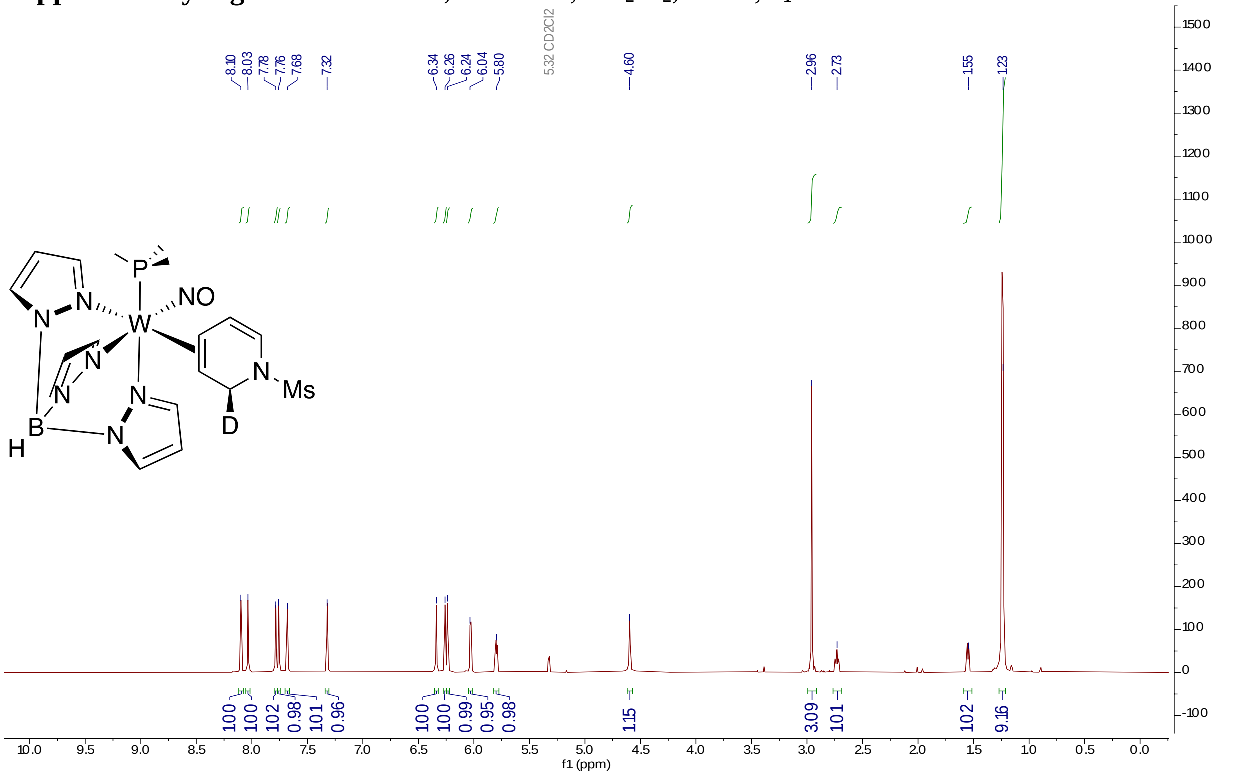

Supplementary Figure 12: ^{13}C NMR, 200 MHz, CD_2Cl_2, 25 °C, ***d*₁-5**

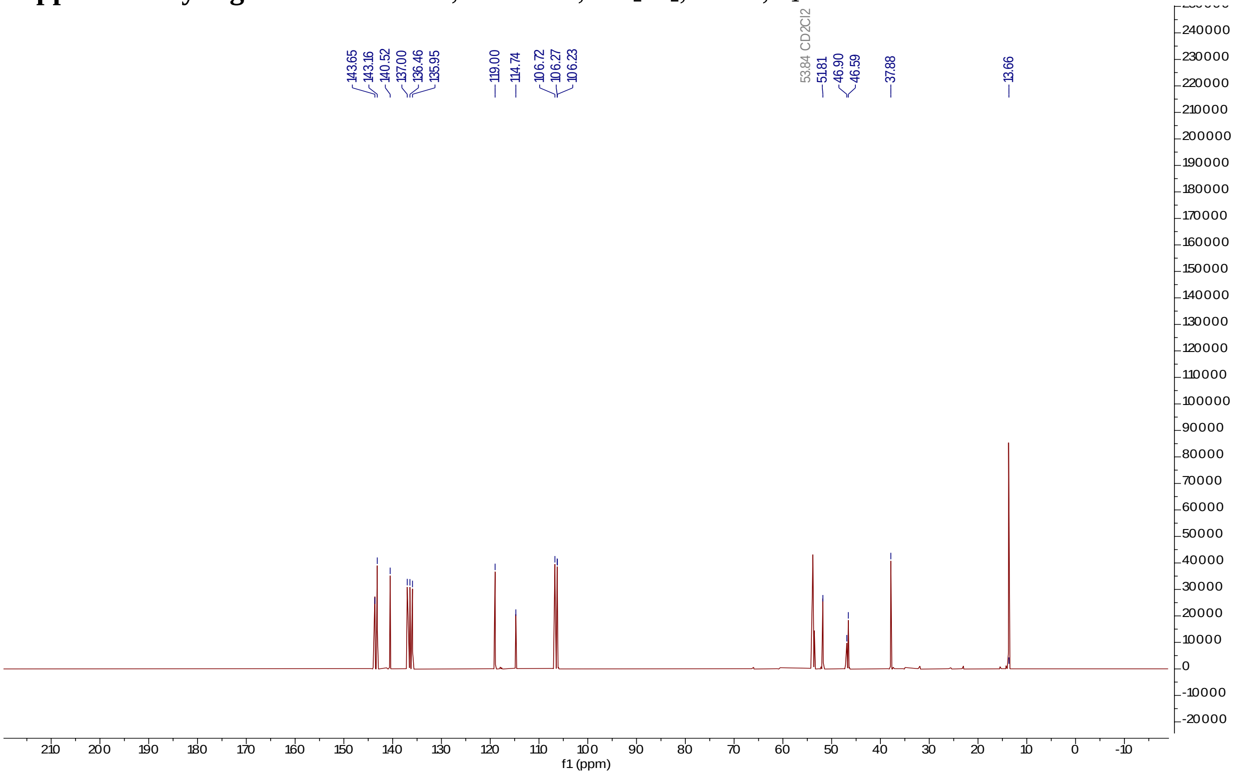

Supplementary Figure 13: ^{1}H NMR, 800 MHz, CD_2Cl_2, 25 °C, ***d*₅-5**

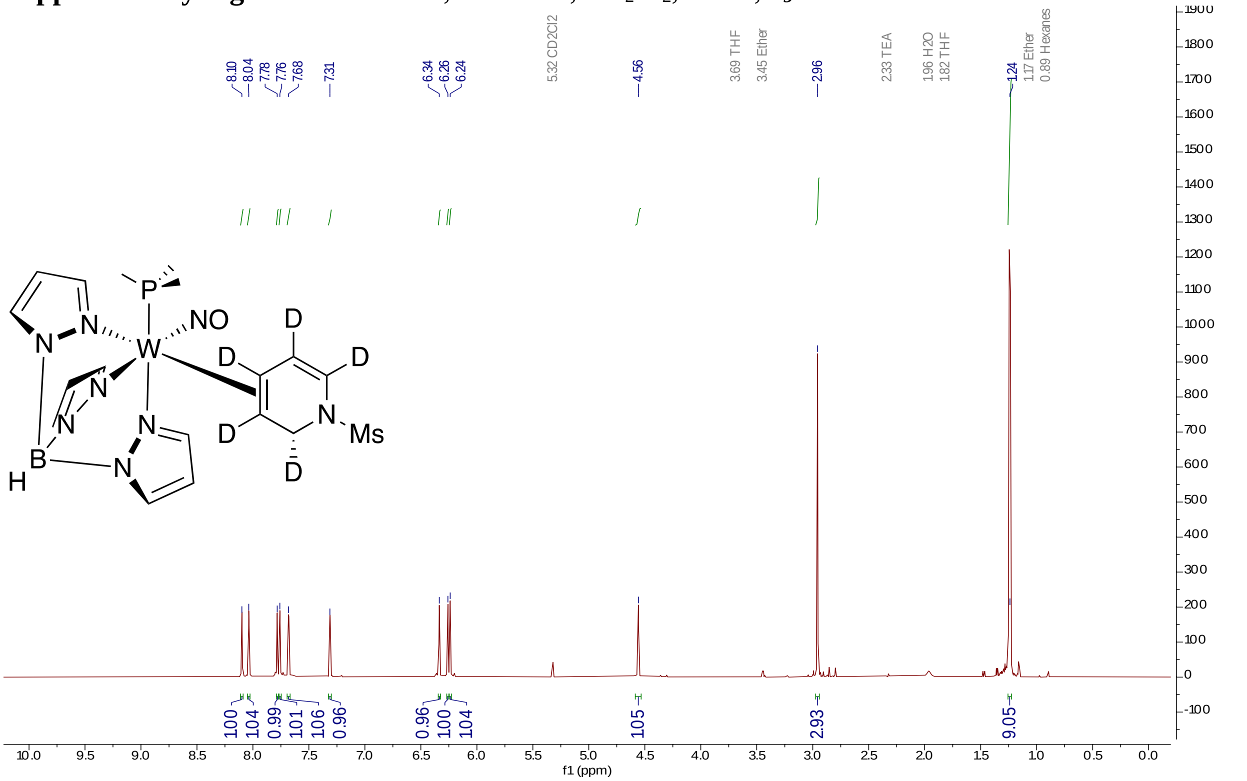

Supplementary Figure 14: ^{13}C NMR, 200 MHz, CD_2Cl_2, 25 °C, ***d*₅-5**

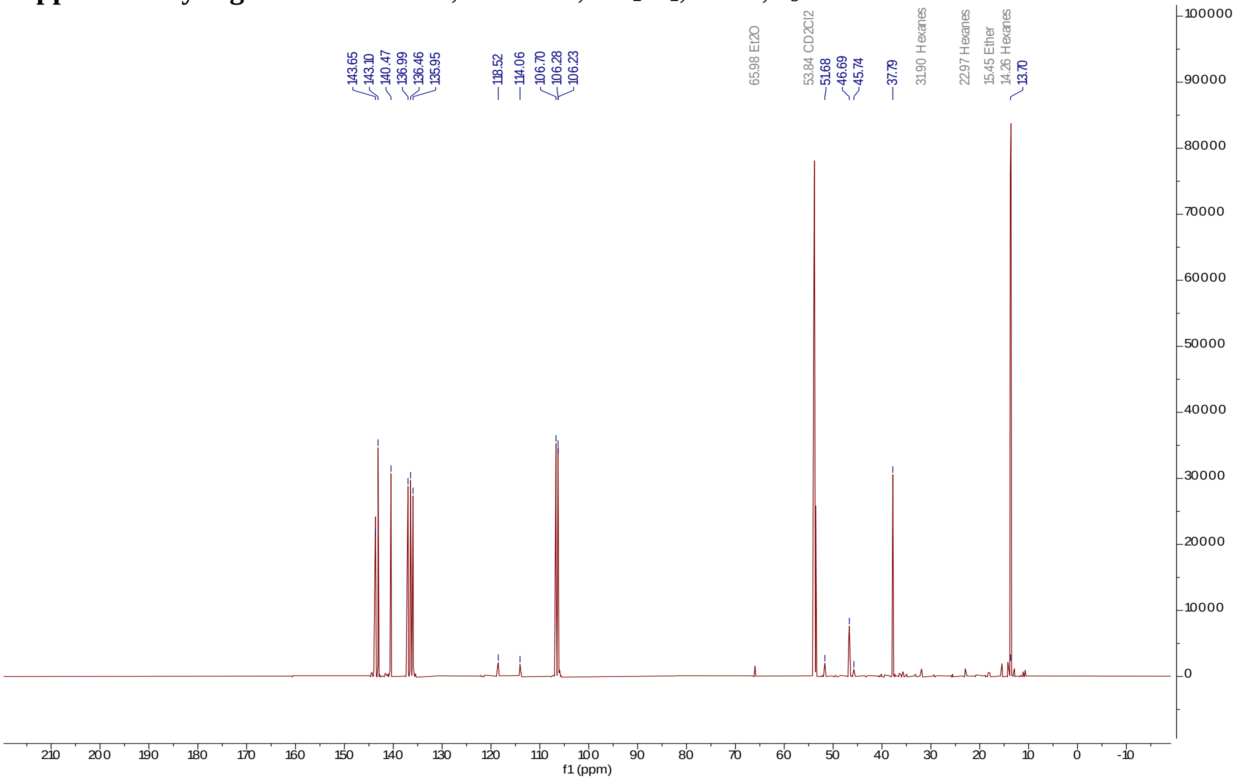

Supplementary Figure 15: ^{1}H NMR, 800 MHz, CD_2Cl_2, 25 °C, ***d*$_6$-5**

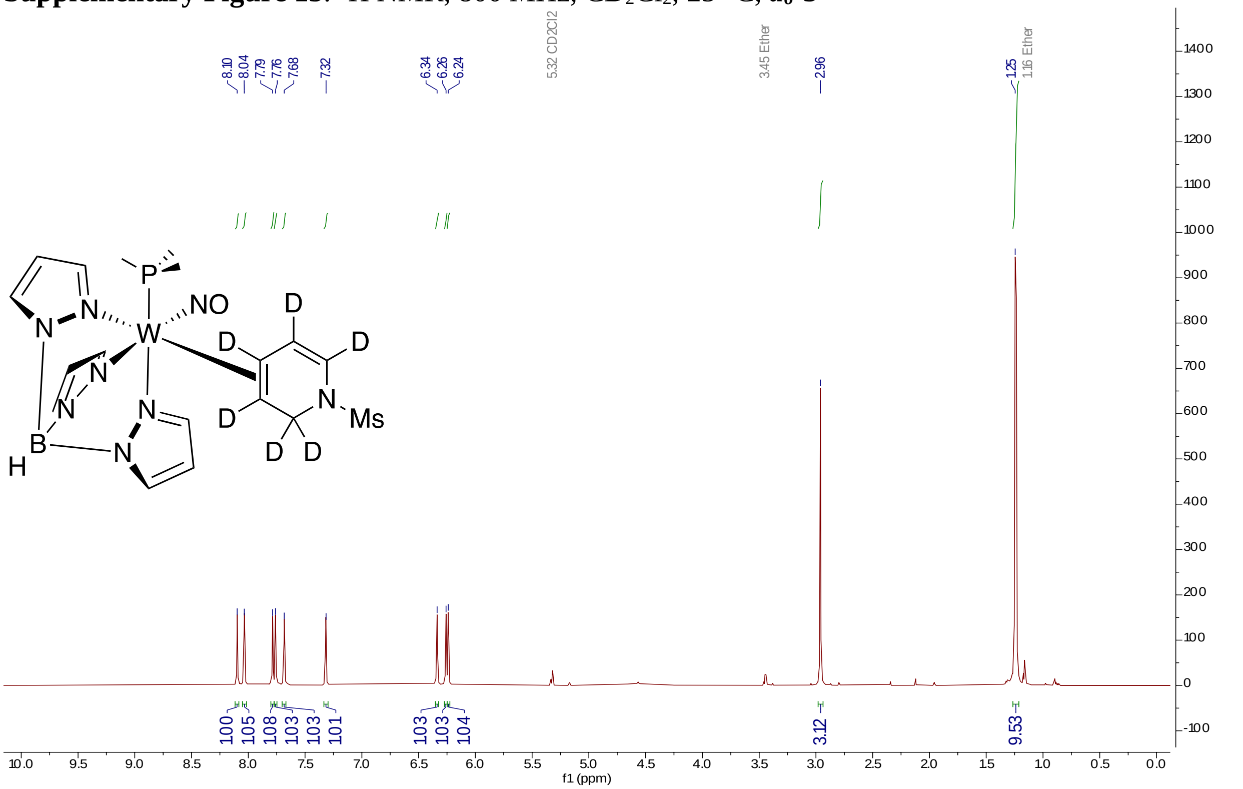

Supplementary Figure 16: ^{13}C NMR, 200 MHz, CD_2Cl_2, 25 °C, ***d*$_6$-5**

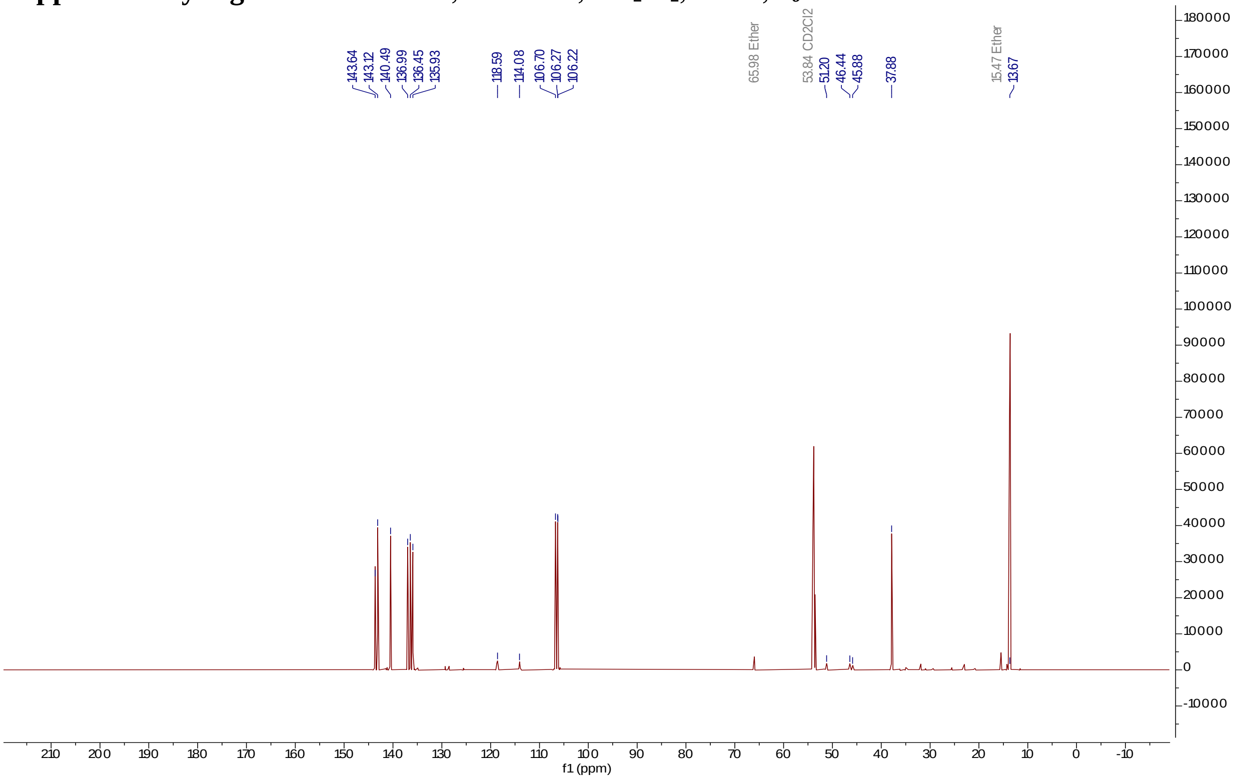

Supplementary Figure 17: ^{1}H NMR, 800 MHz, CD_2Cl_2, 25 °C, **6**

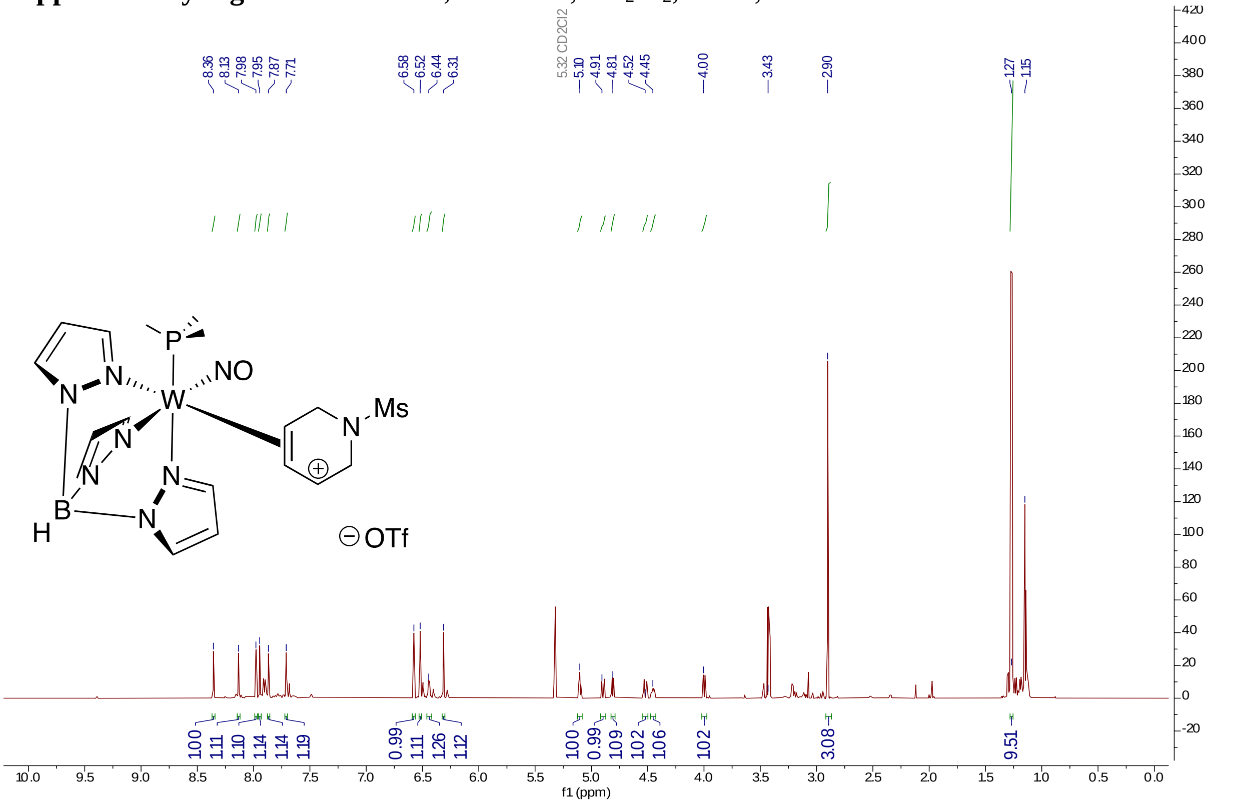

Supplementary Figure 18: ^{13}C NMR, 200 MHz, CD_2Cl_2, 25 °C, **6**

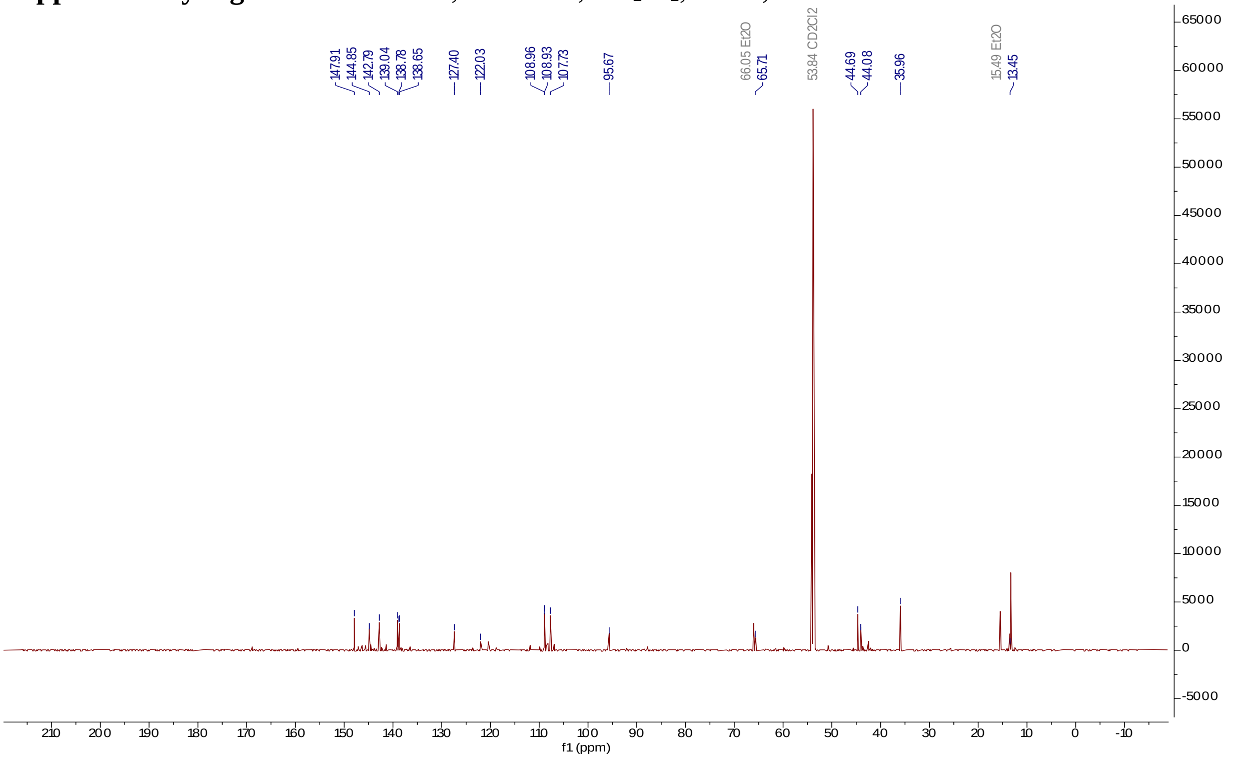

Supplementary Figure 19: NOESY NMR, 800 MHz, CD_2Cl_2, 25 °C, **6**

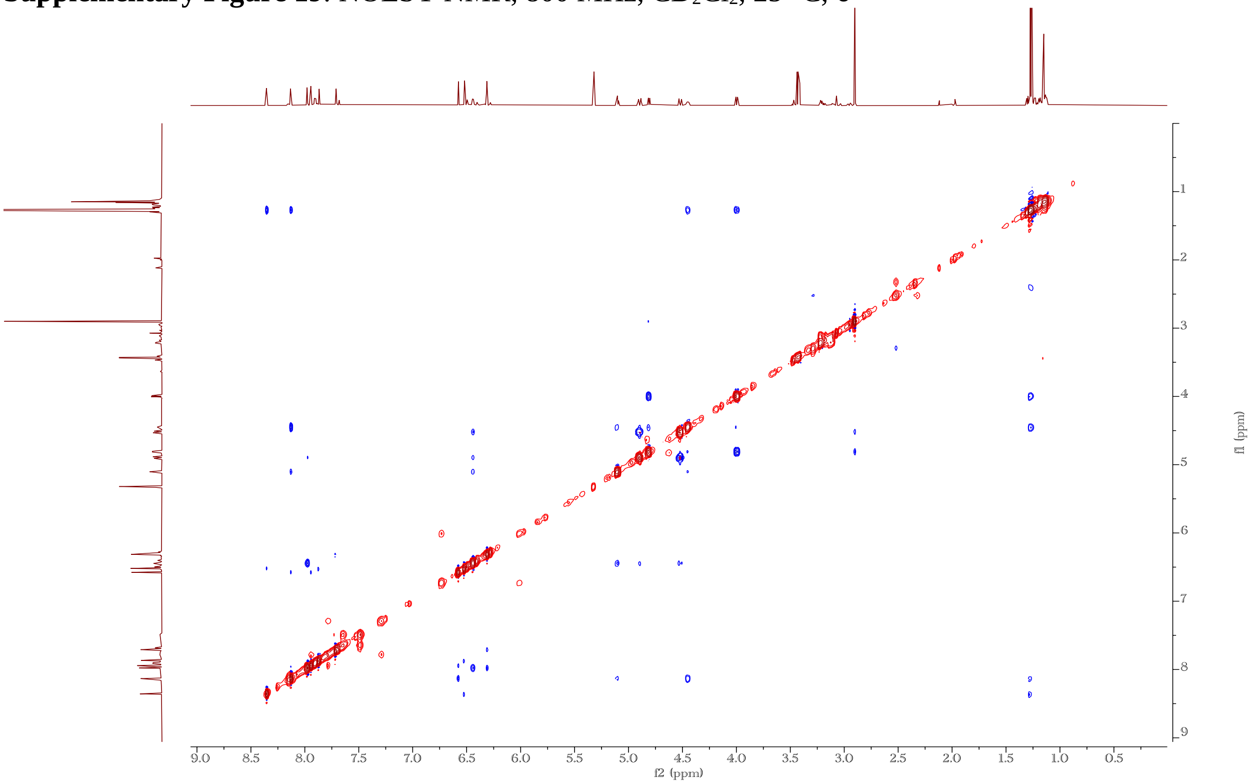

Supplementary Figure 20: COSY NMR, 800 MHz, CD_2Cl_2, 25 °C, **6**

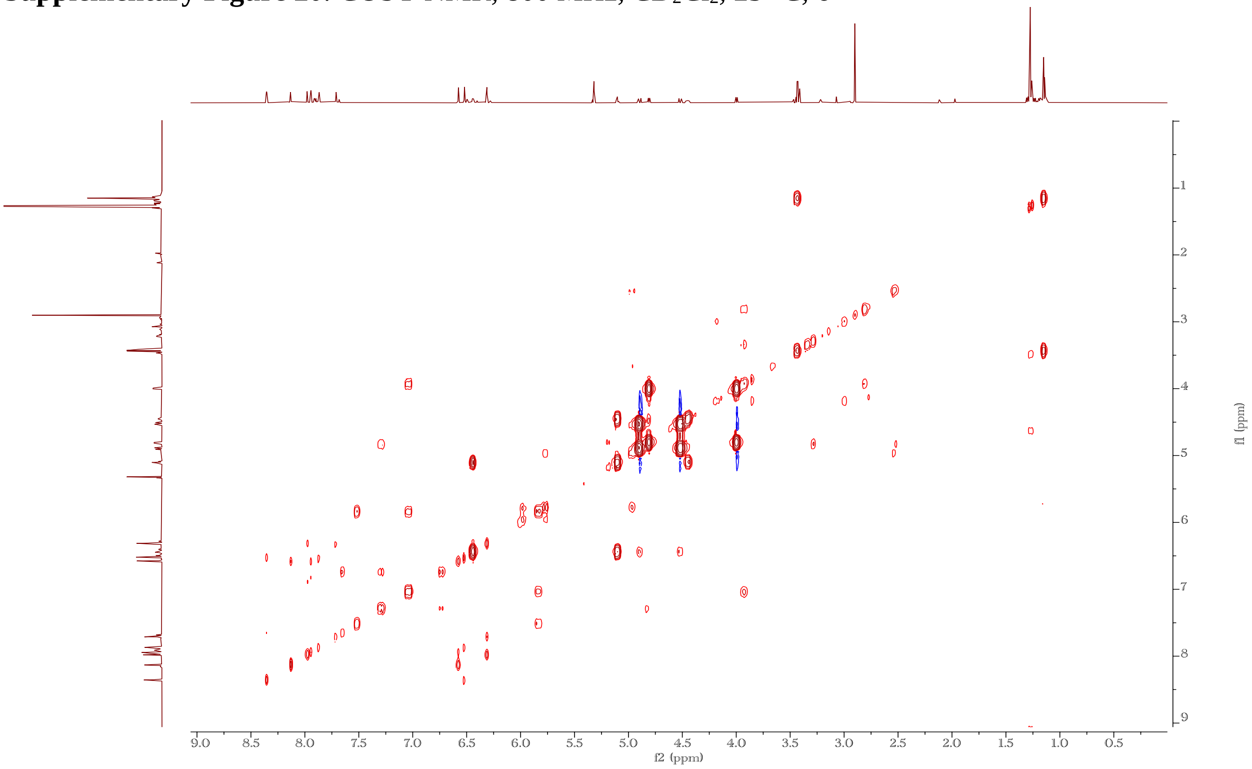

Supplementary Figure 21: HSQC NMR, 800 MHz, CD_2Cl_2, 25 °C, **6**

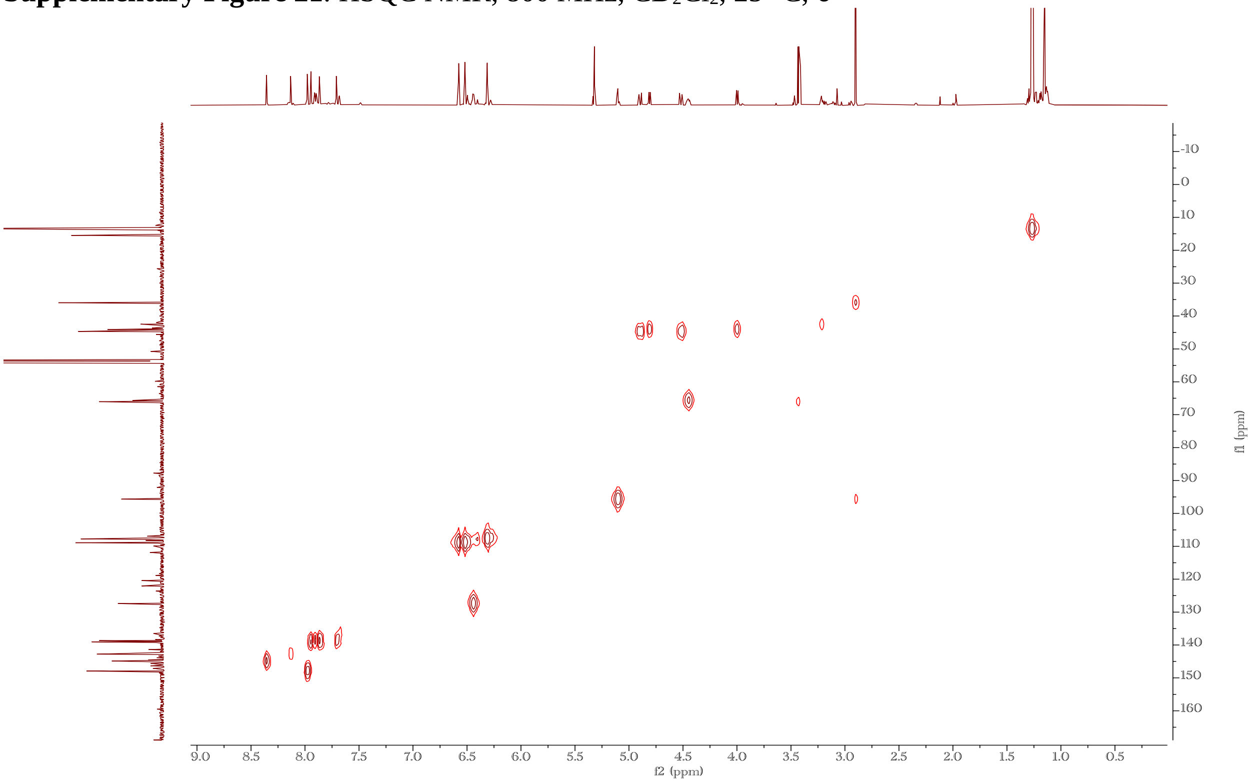

Supplementary Figure 22: HMBC NMR, 800 MHz, CD_2Cl_2, 25 °C, **6**

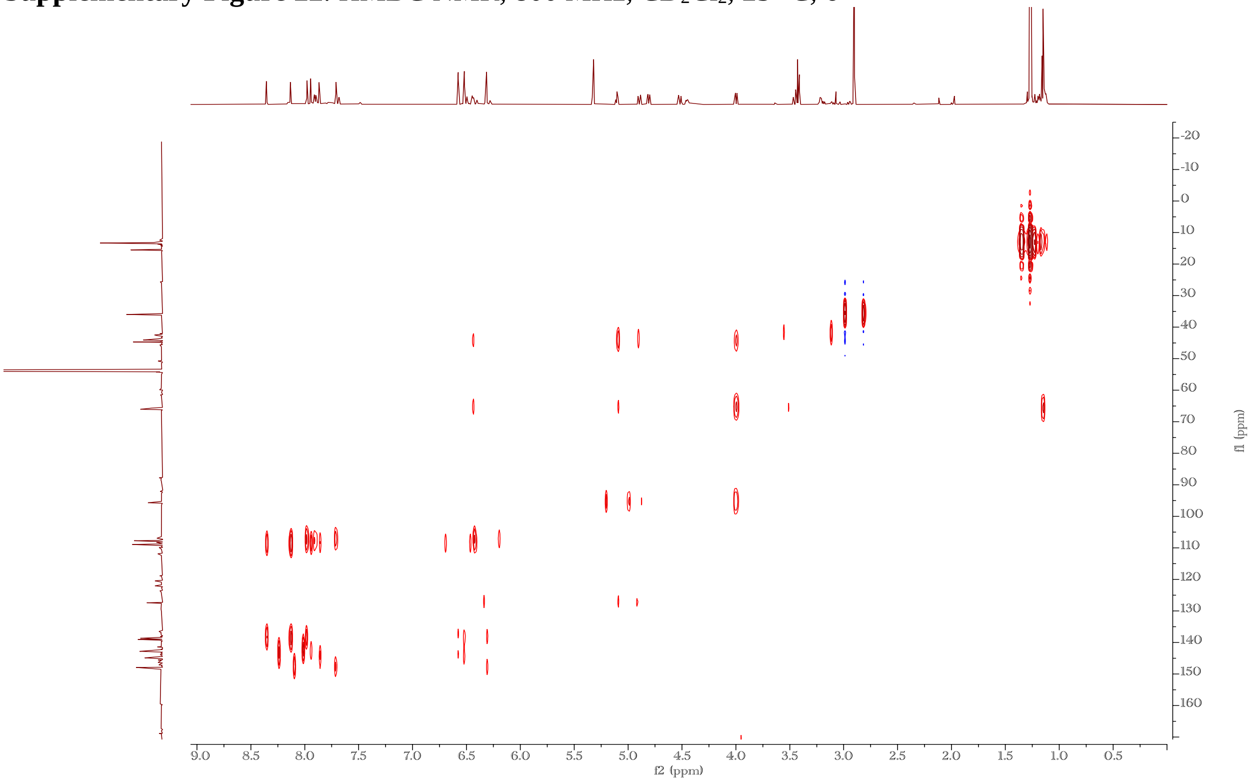

Supplementary Figure 23: ^{1}H NMR, 800 MHz, CD_2Cl_2, 25 °C, ***d₁*-6**

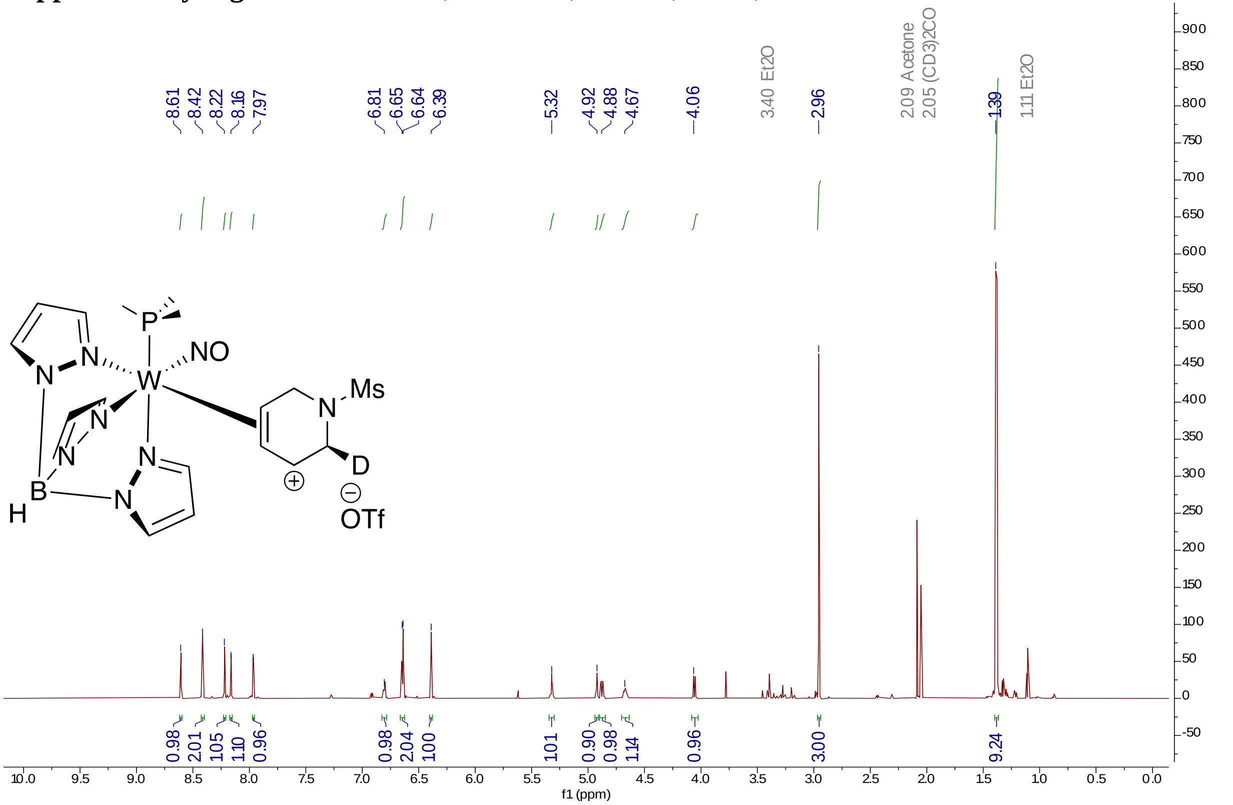

Supplementary Figure 24: ^{13}C NMR, 200 MHz, CD_2Cl_2, 25 °C, ***d₁*-6**

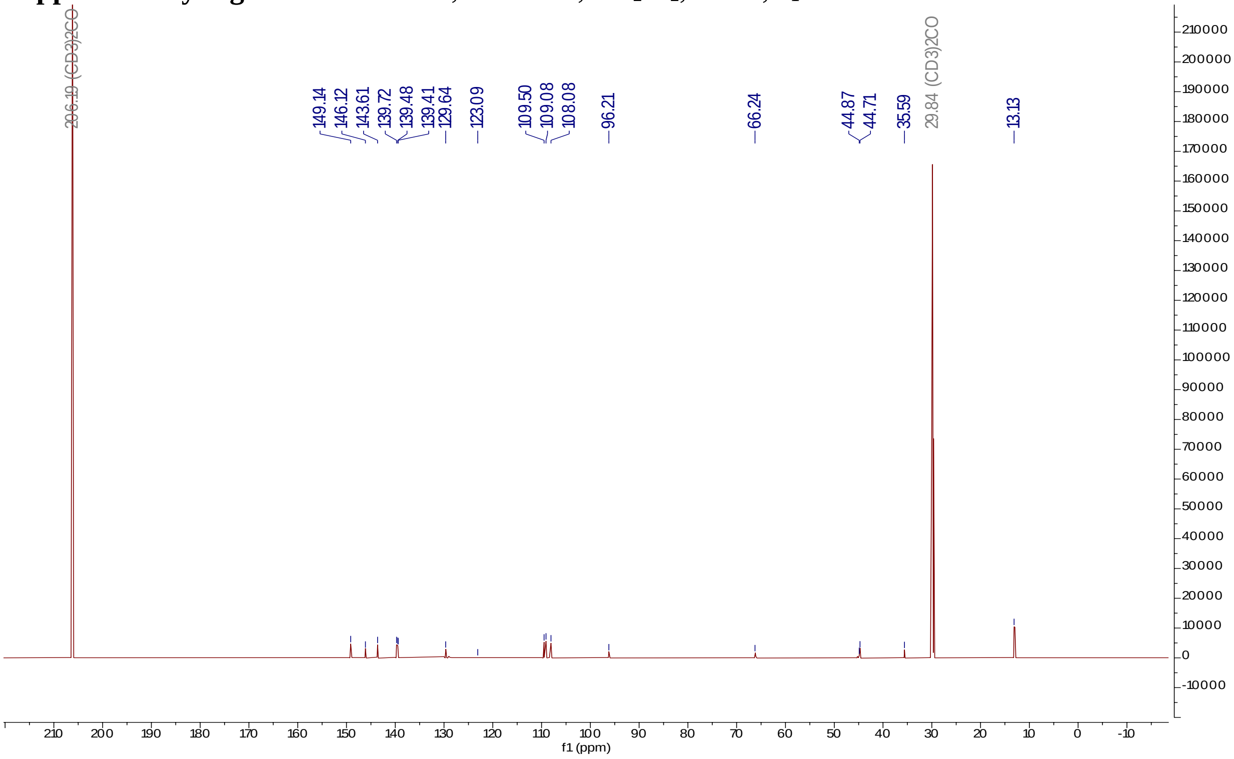

Supplementary Figure 25: ^{1}H NMR, 800 MHz, CD_2Cl_2, 25 °C, ***d*₅-6**

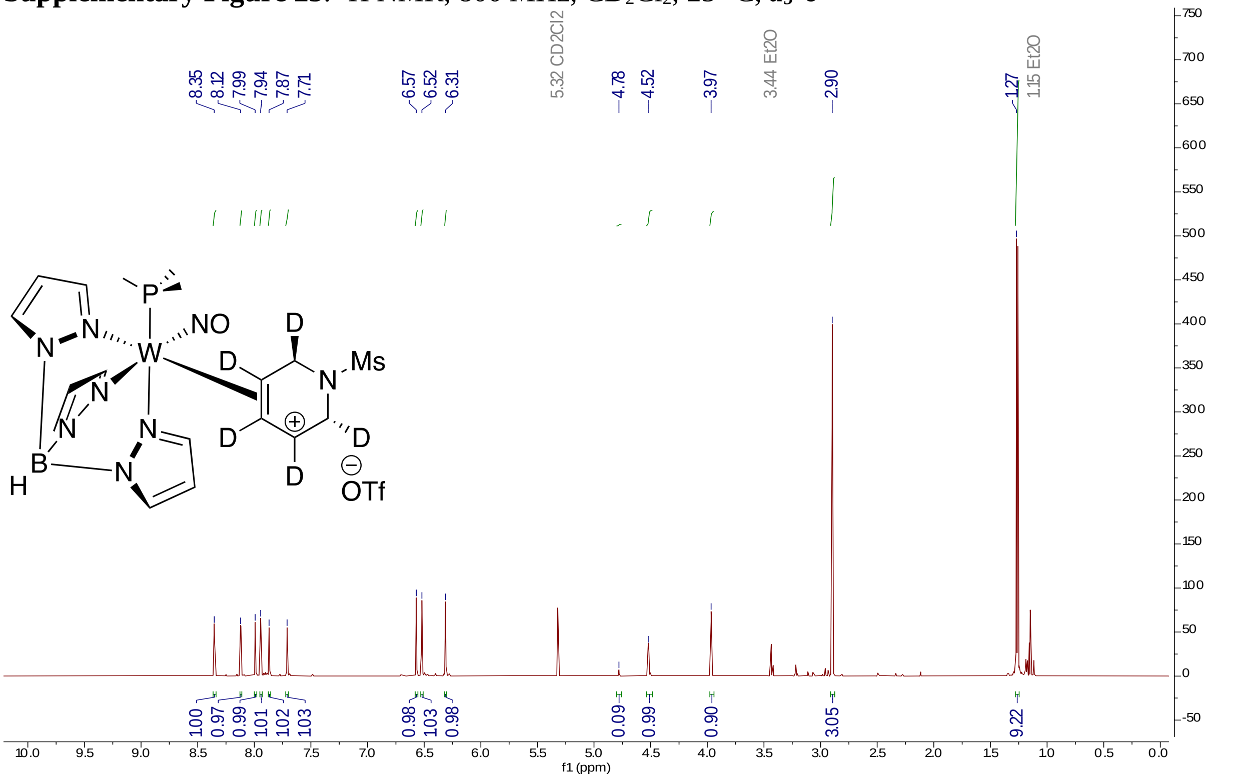

Supplementary Figure 26: ^{13}C NMR, 200 MHz, CD_2Cl_2, 25 °C, ***d*₅-6**

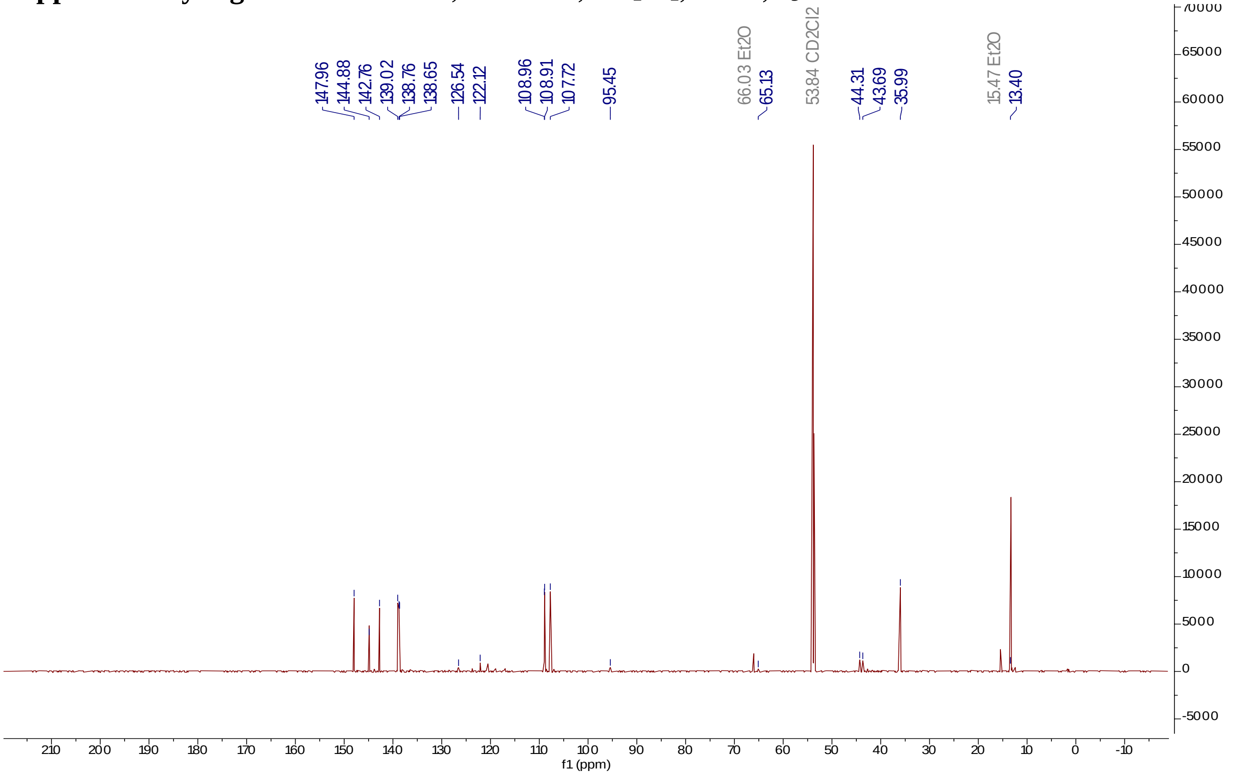

Supplementary Figure 27: ^{1}H NMR, 800 MHz, CD_2Cl_2, 25 °C, ***d*$_6$-6**

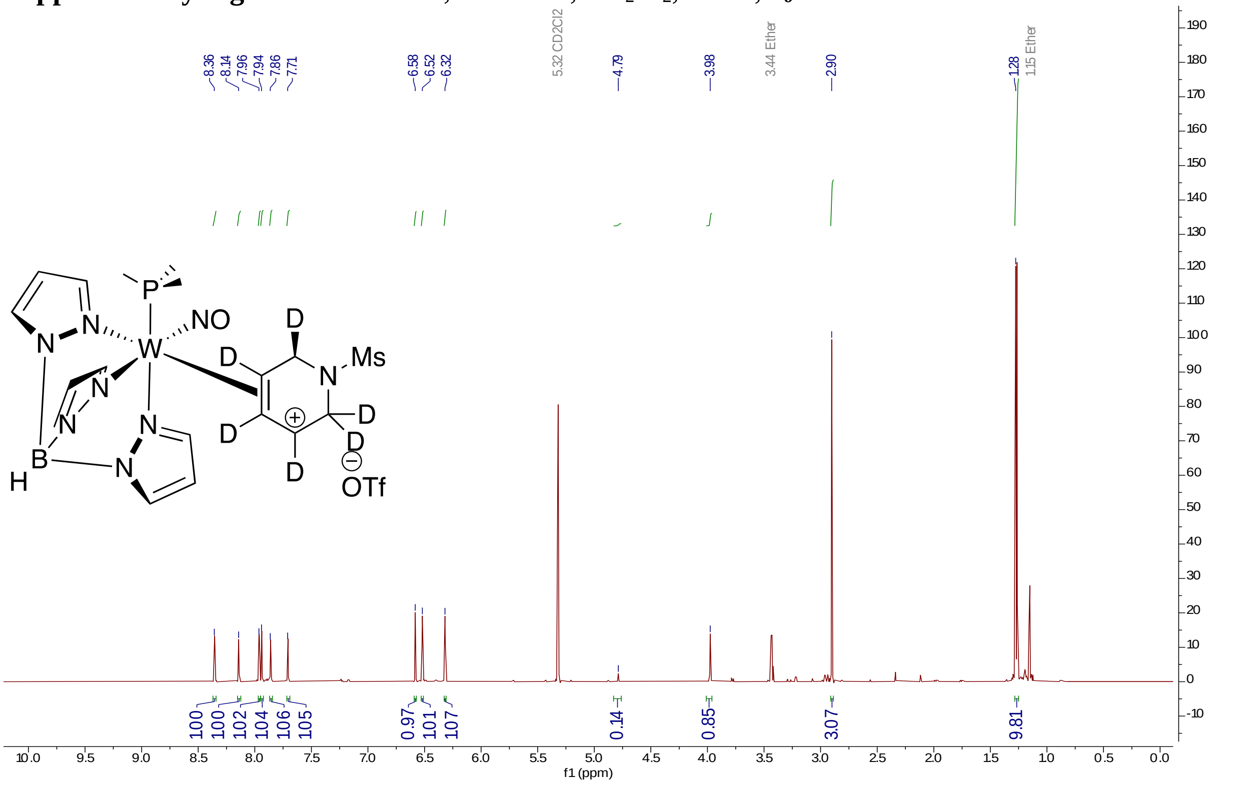

Supplementary Figure 28: ^{13}C NMR, 200 MHz, CD_2Cl_2, 25 °C, ***d*$_6$-6**

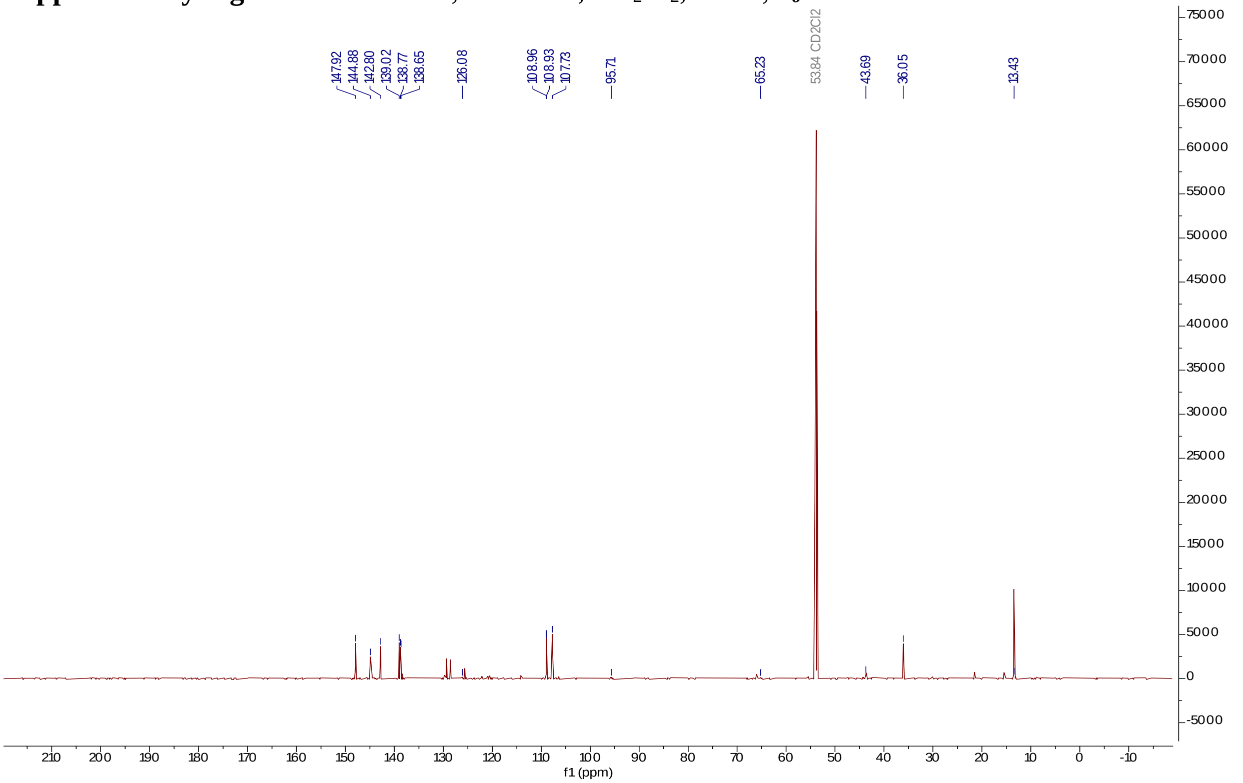

Supplementary Figure 29: ^{1}H NMR, 800 MHz, CD_2Cl_2, 25 °C, ***d*₇-6**

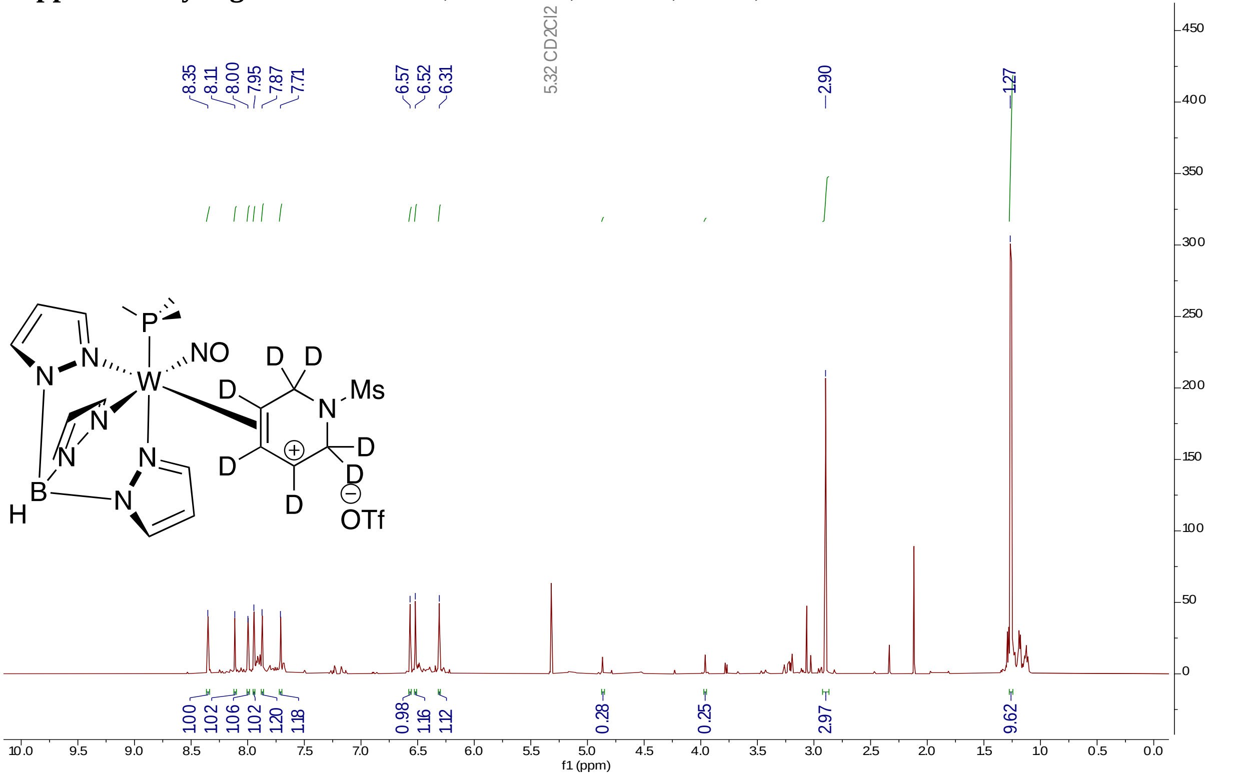

Supplementary Figure 30: ^{13}C NMR, 200 MHz, CD_2Cl_2, 25 °C, ***d*₇-6**

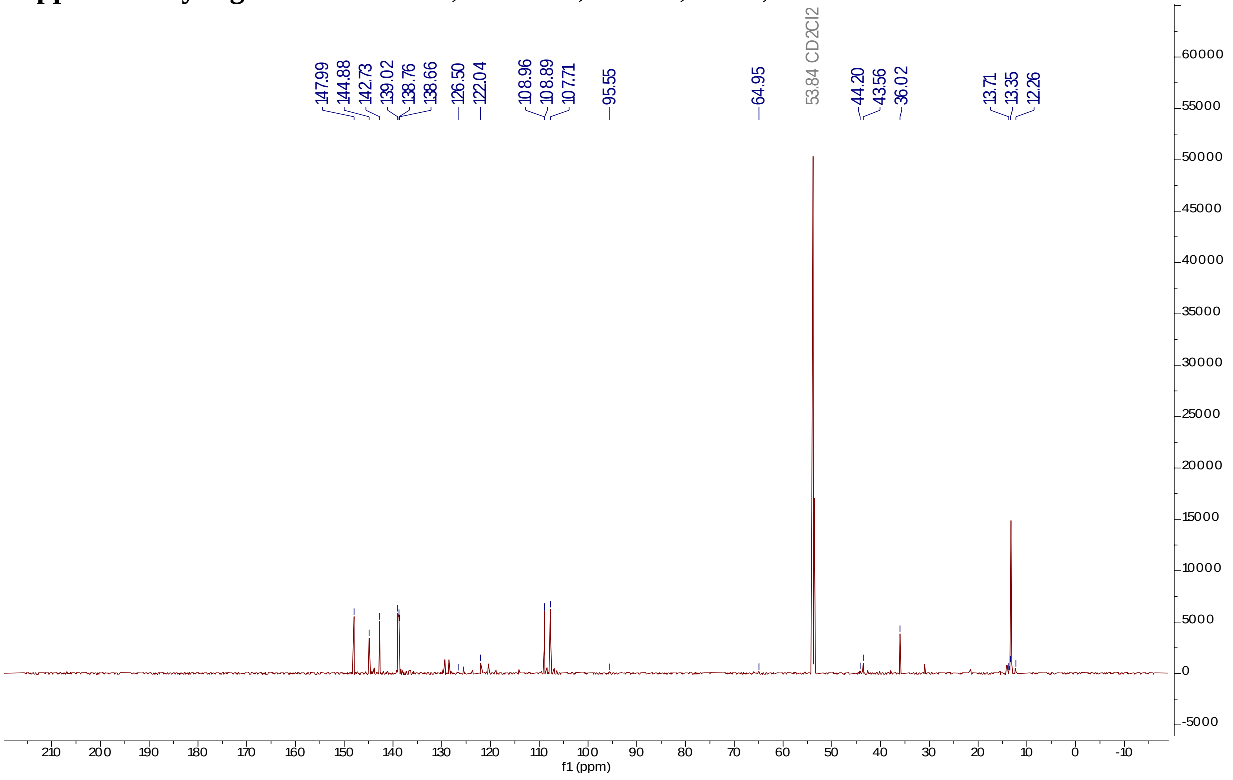

Supplementary Figure 31: ^1H NMR, 800 MHz, CD_2Cl_2, 25 °C, **7**

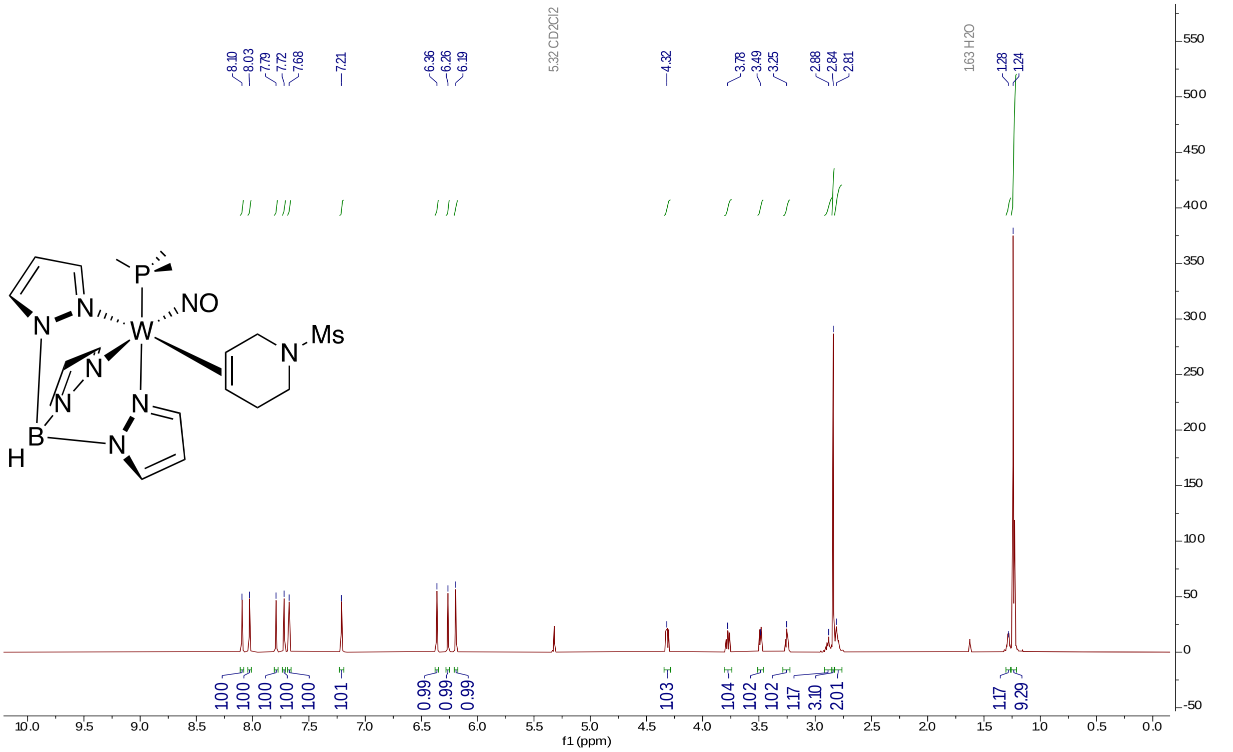

Supplementary Figure 32: ^{13}C NMR, 200 MHz, CD_2Cl_2, 25 °C, **7**

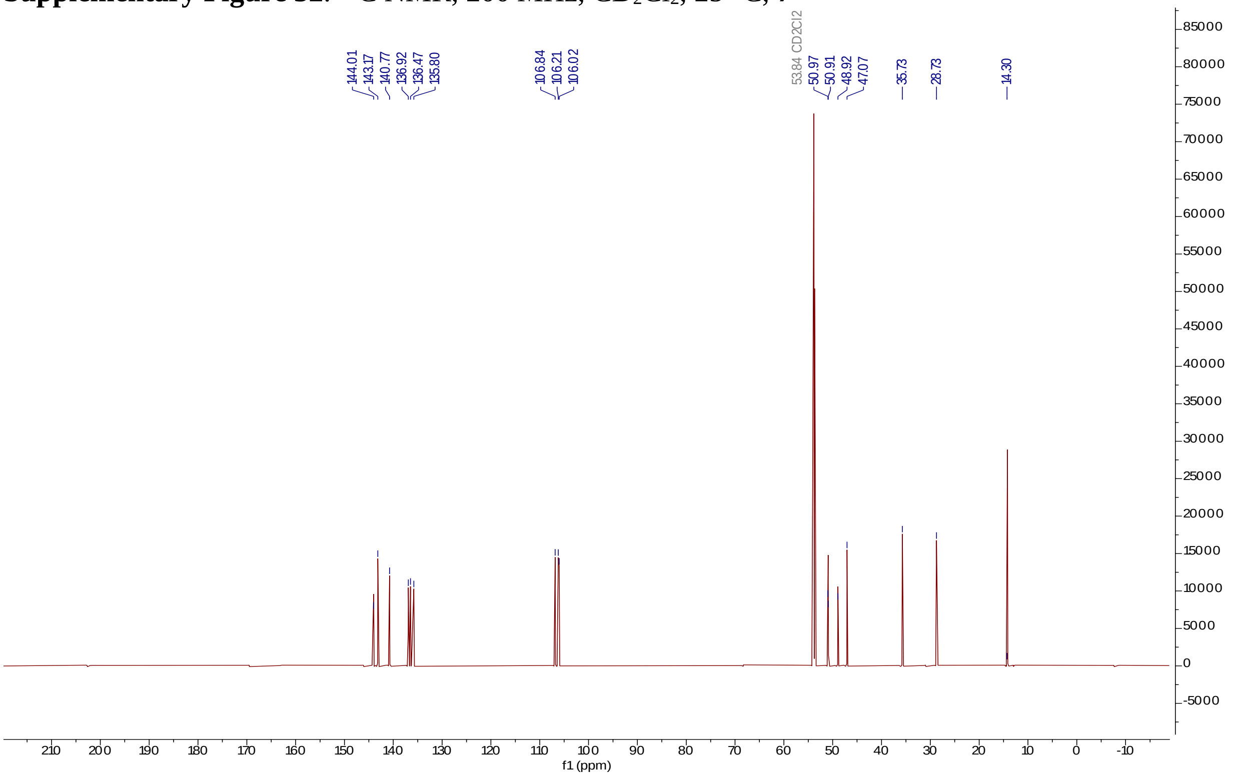

Supplementary Figure 33: ^{1}H NMR, 800 MHz, CD_2Cl_2, 25 °C, ***Rel-(2S)-d$_1$-7***

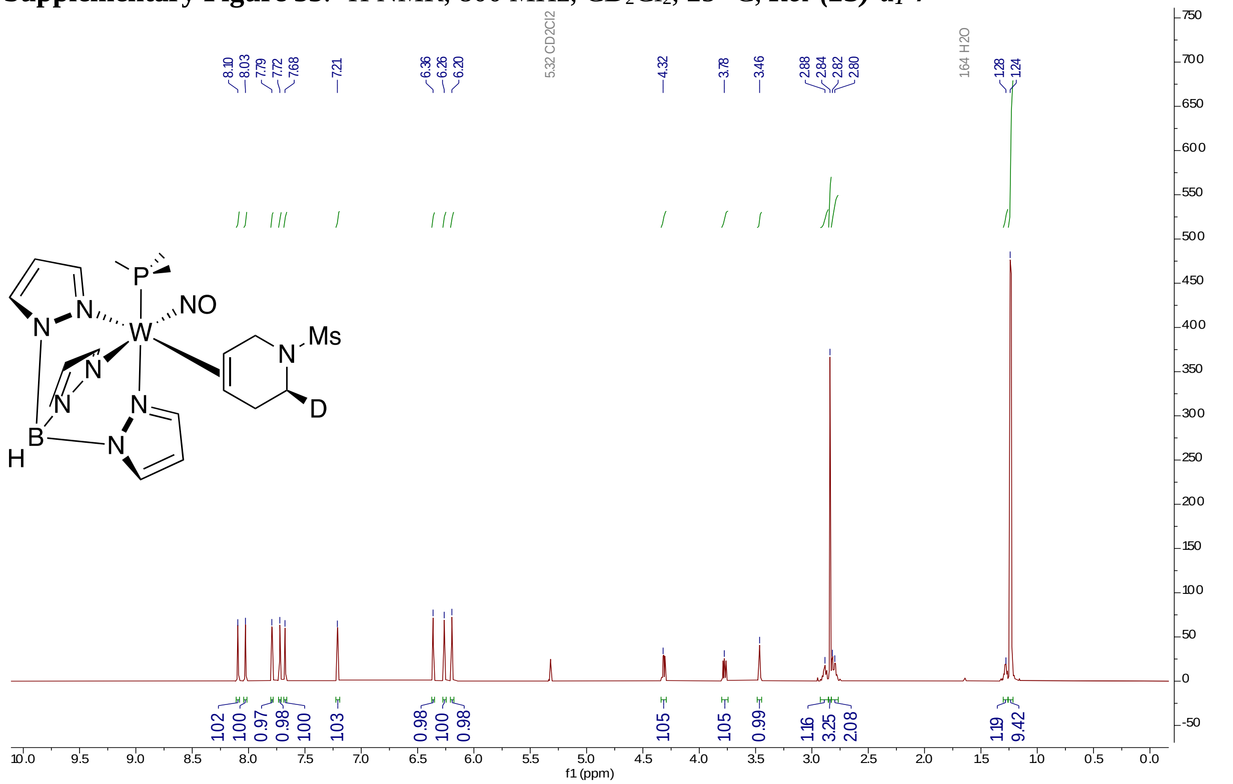

Supplementary Figure 34: ^{13}C NMR, 200 MHz, CD_2Cl_2, 25 °C, ***Rel-(2S)-d$_1$-7***

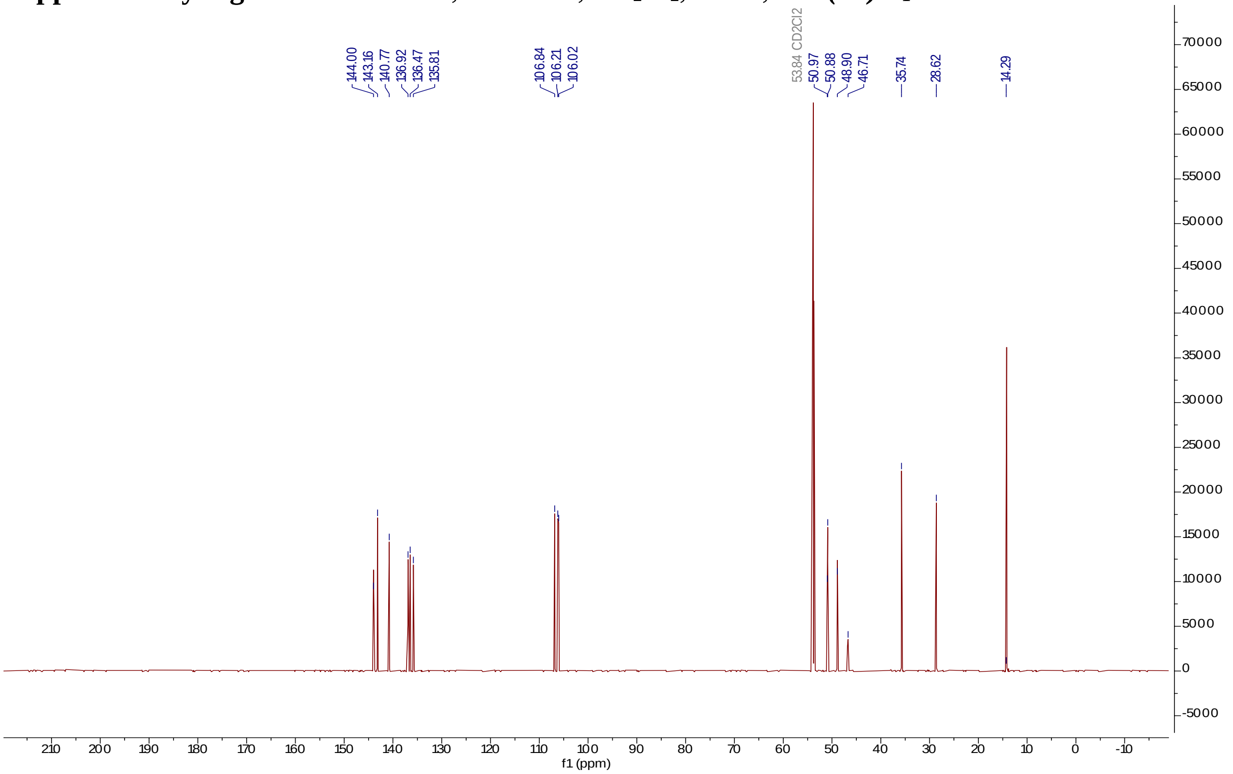

Supplementary Figure 35: ^{1}H NMR, 800 MHz, CD_2Cl_2, 25 °C, ***Rel-(3S)-d₁-7***

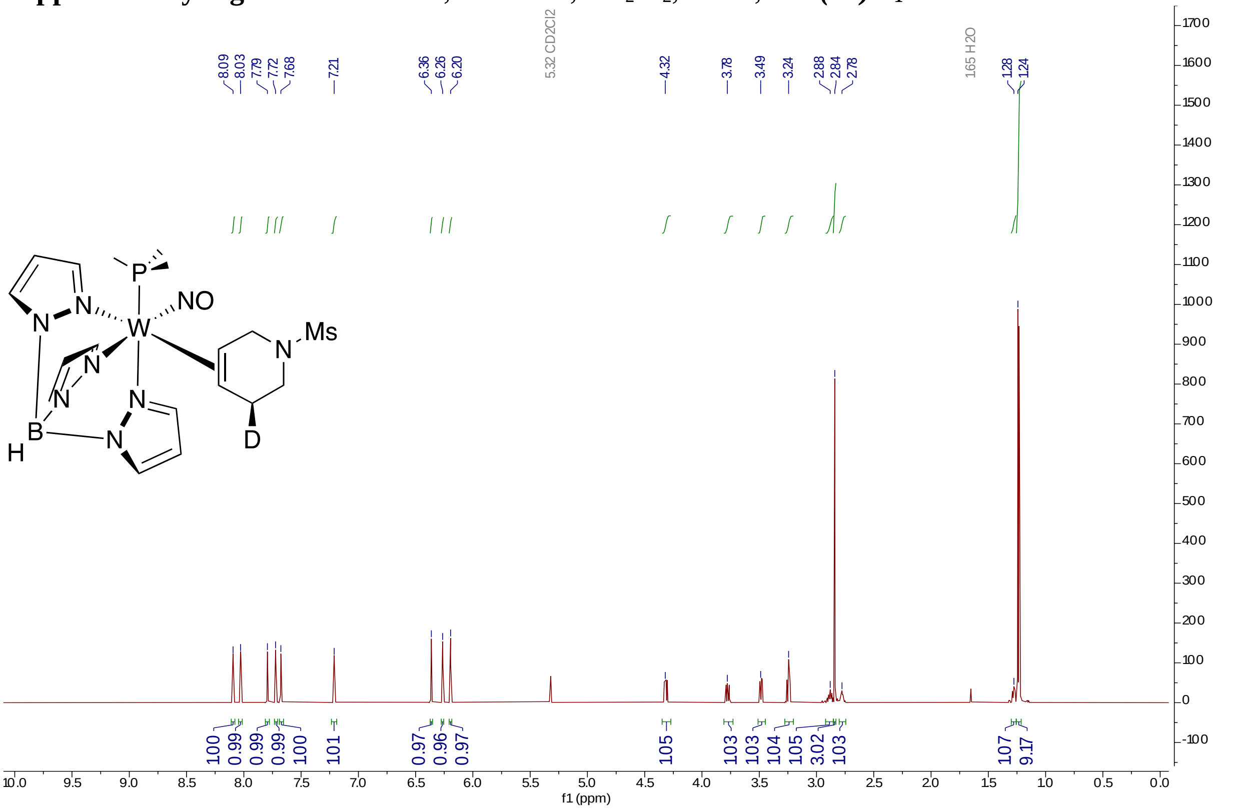

Supplementary Figure 36: ^{13}C NMR, 200 MHz, CD_2Cl_2, 25 °C, ***Rel-(3S)-d₁-7***

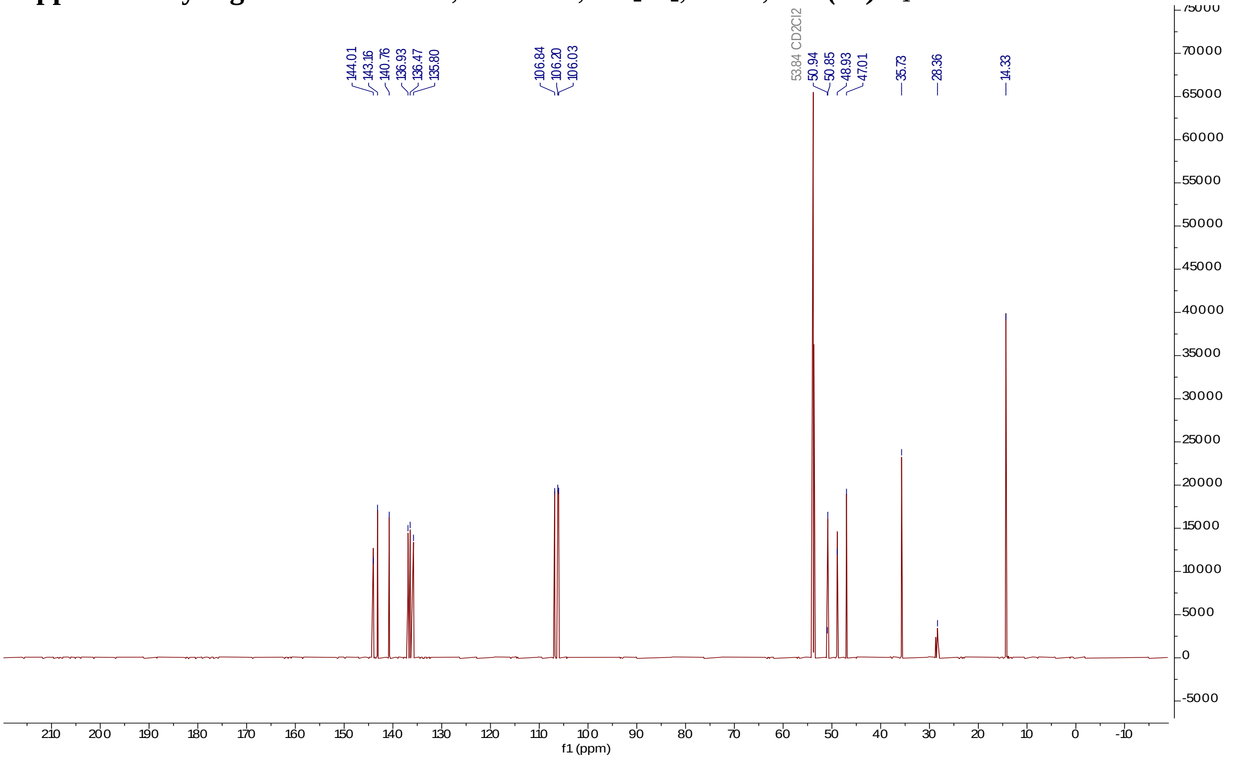

Supplementary Figure 37: ^{1}H NMR, 800 MHz, CD_2Cl_2, 25 °C, ***Rel-(2S,3S)-d₂-7***

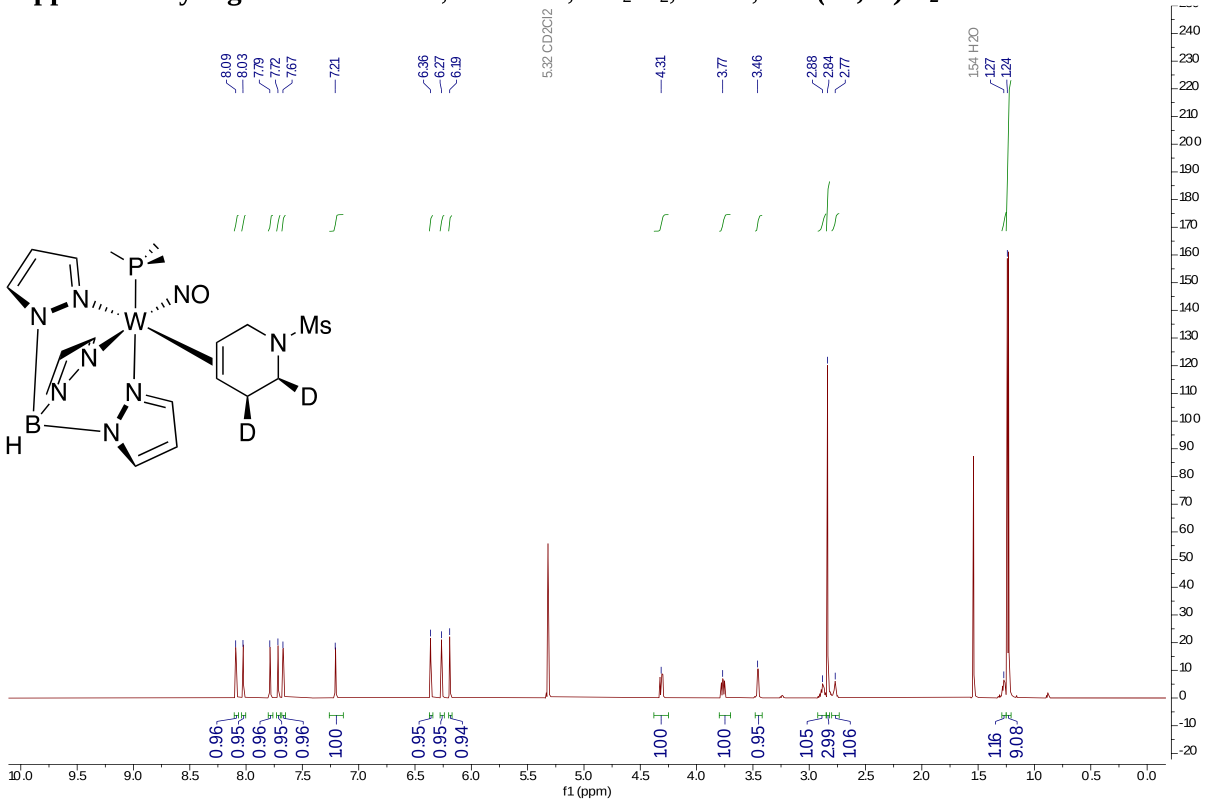

Supplementary Figure 38: ^{13}C NMR, 200 MHz, CD_2Cl_2, 25 °C, ***Rel-(2S,3S)-d₂-7***

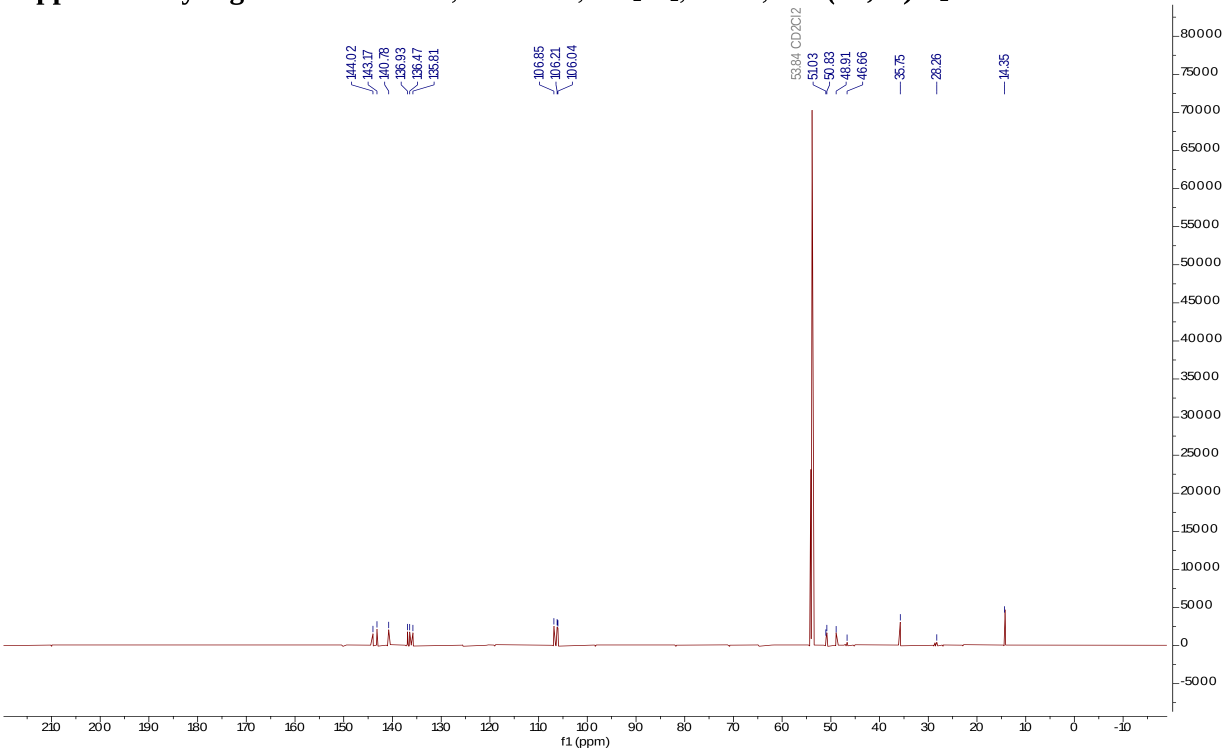

Supplementary Figure 39: ^{1}H NMR, 800 MHz, CD_2Cl_2, 25 °C, ***Rel-(2R,3R,6R)-d$_5$-7***

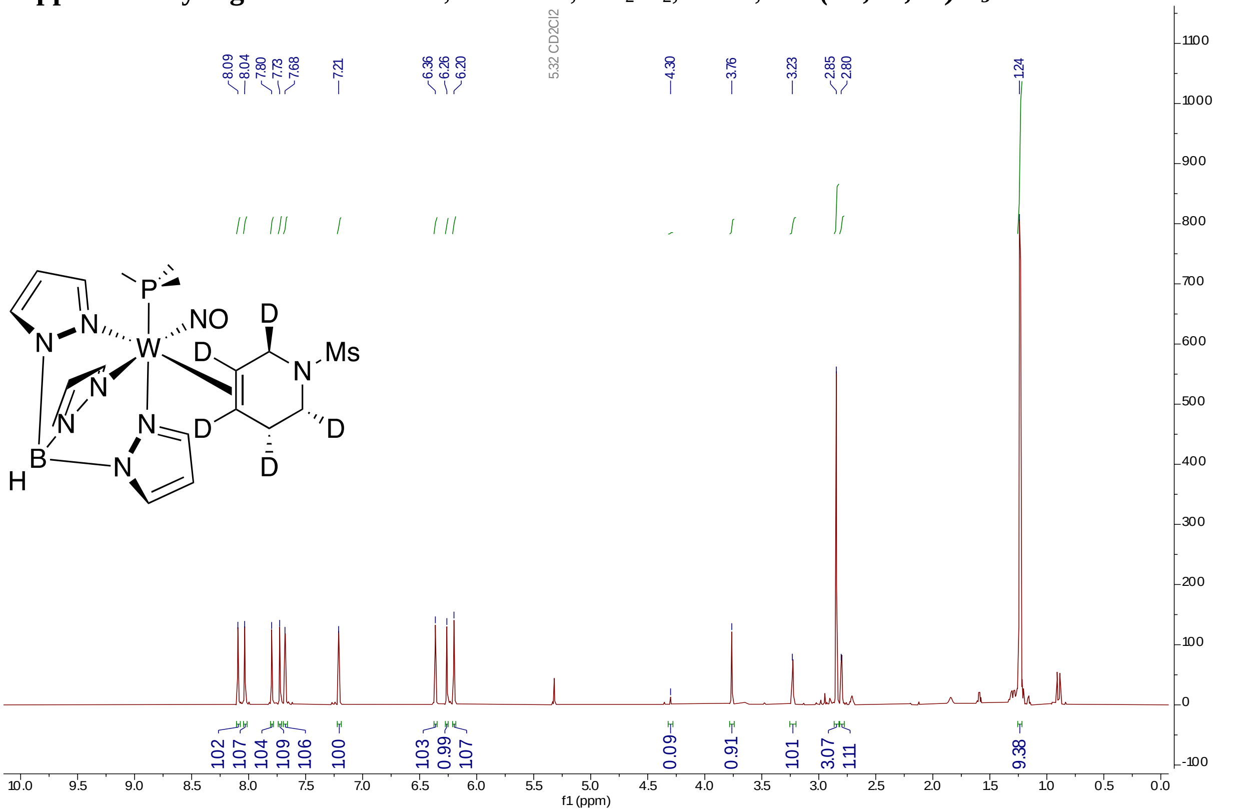

Supplementary Figure 40: ^{13}C NMR, 200 MHz, CD_2Cl_2, 25 °C, ***Rel-(2R,3R,6R)-d$_5$-7***

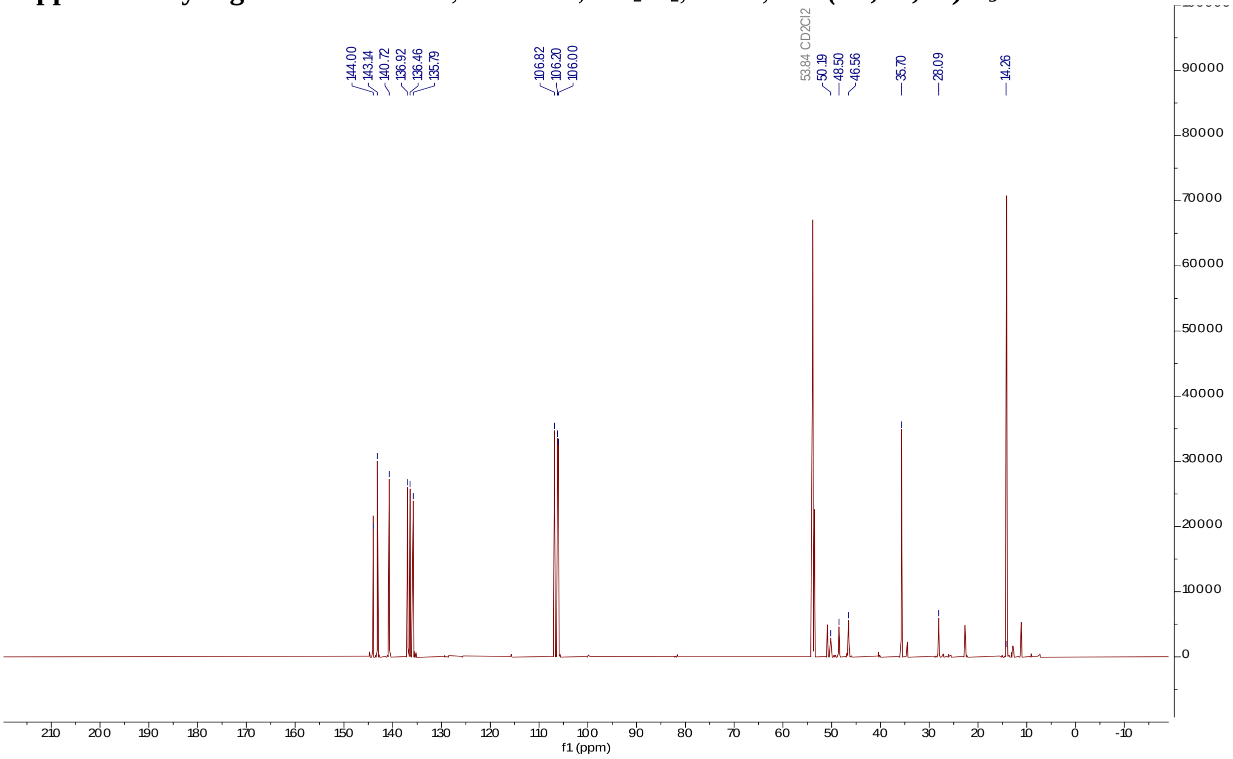

Supplementary Figure 41: ^{1}H NMR, 800 MHz, CD_2Cl_2, 25 °C, ***Rel-(3R,6R)-d$_6$-7***

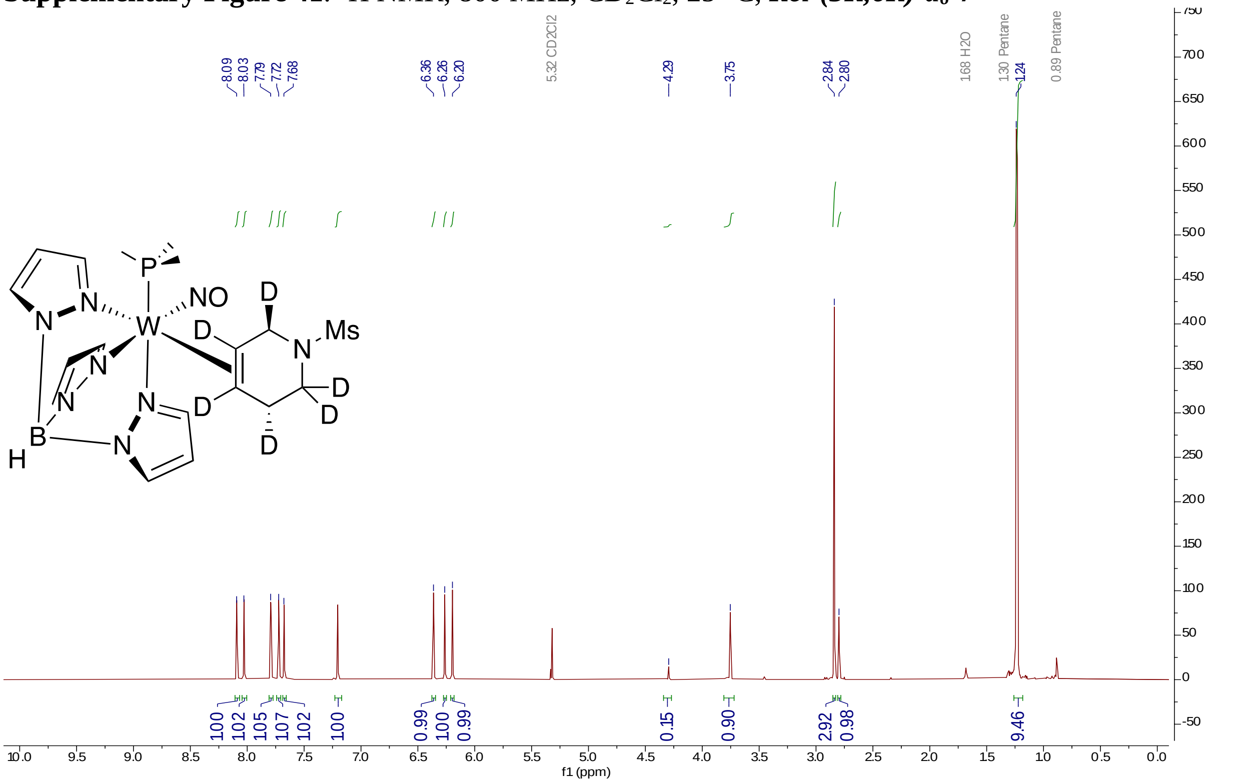

Supplementary Figure 42: ^{13}C NMR, 200 MHz, CD_2Cl_2, 25 °C, ***Rel-(3R,6R)-d$_6$-7***

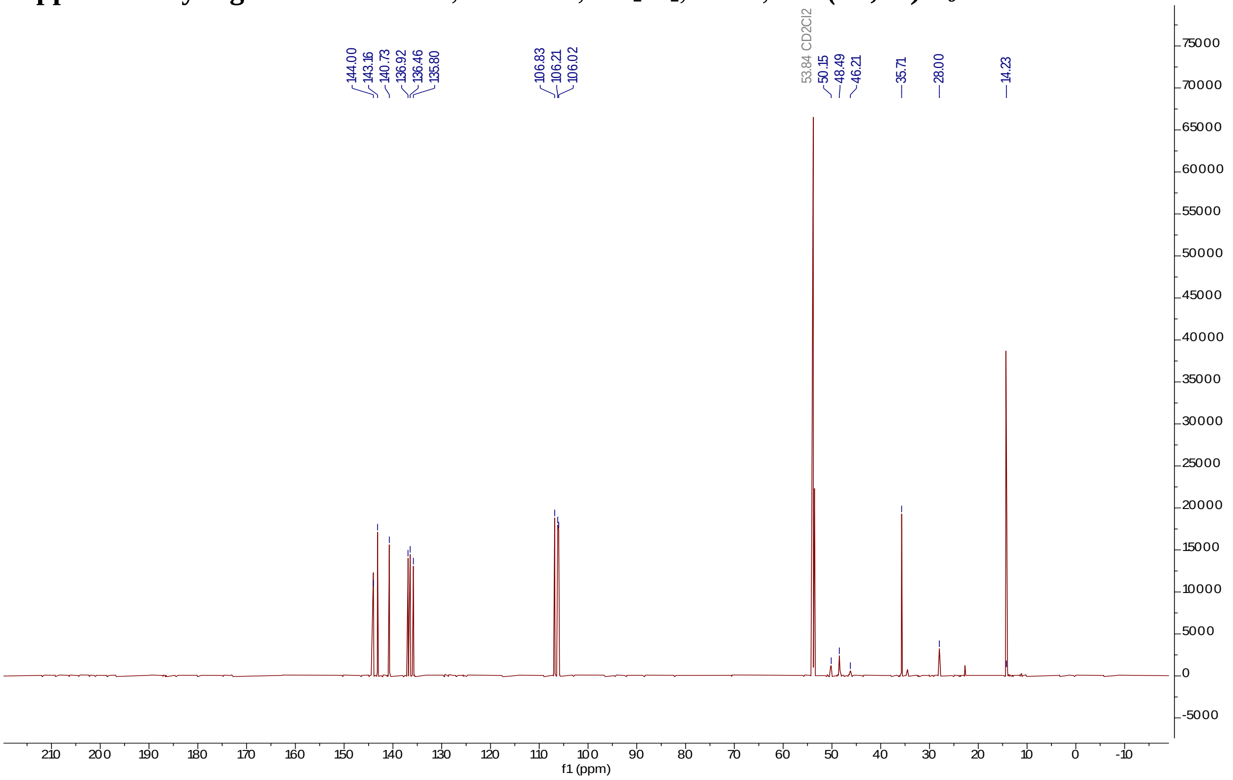

Supplementary Figure 43: ^{1}H NMR, 800 MHz, CD_2Cl_2, 25 °C, ***Rel-(2R,6R)-d$_6$-7***

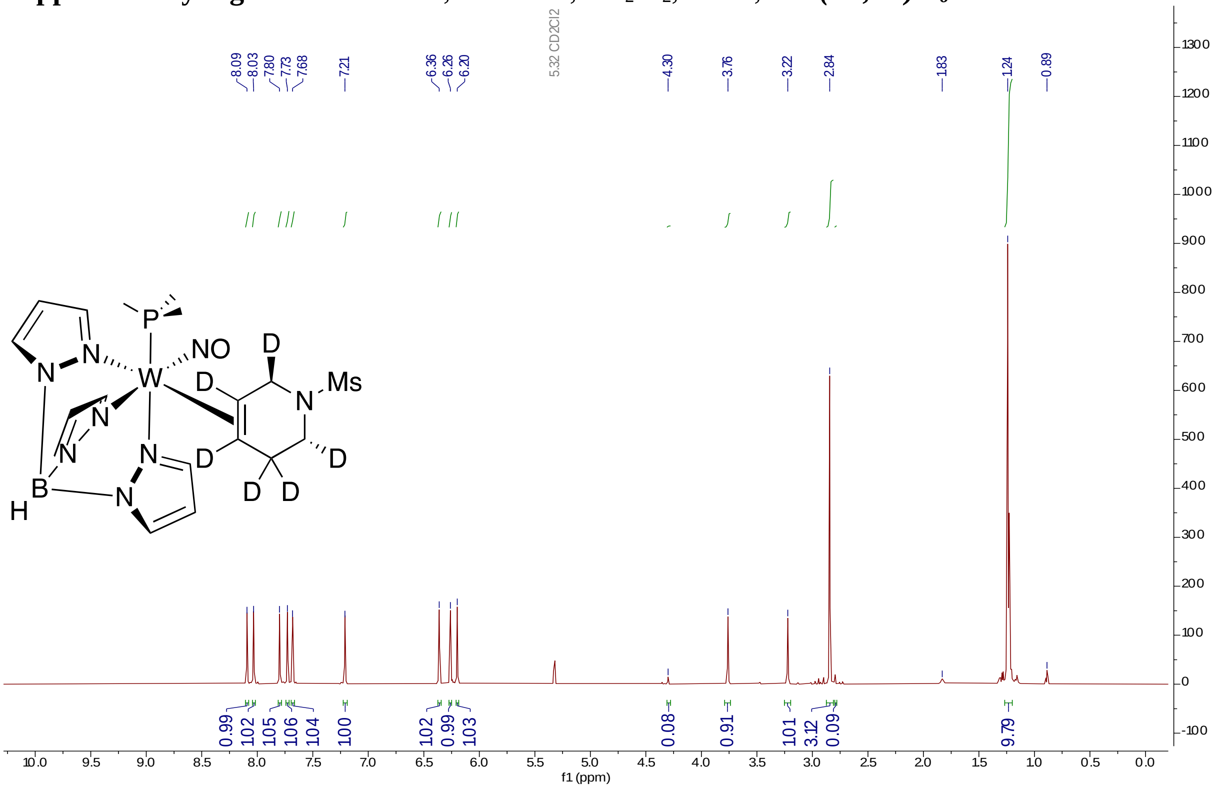

Supplementary Figure 44: ^{13}C NMR, 200 MHz, CD_2Cl_2, 25 °C, ***Rel-(2R,6R)-d$_6$-7***

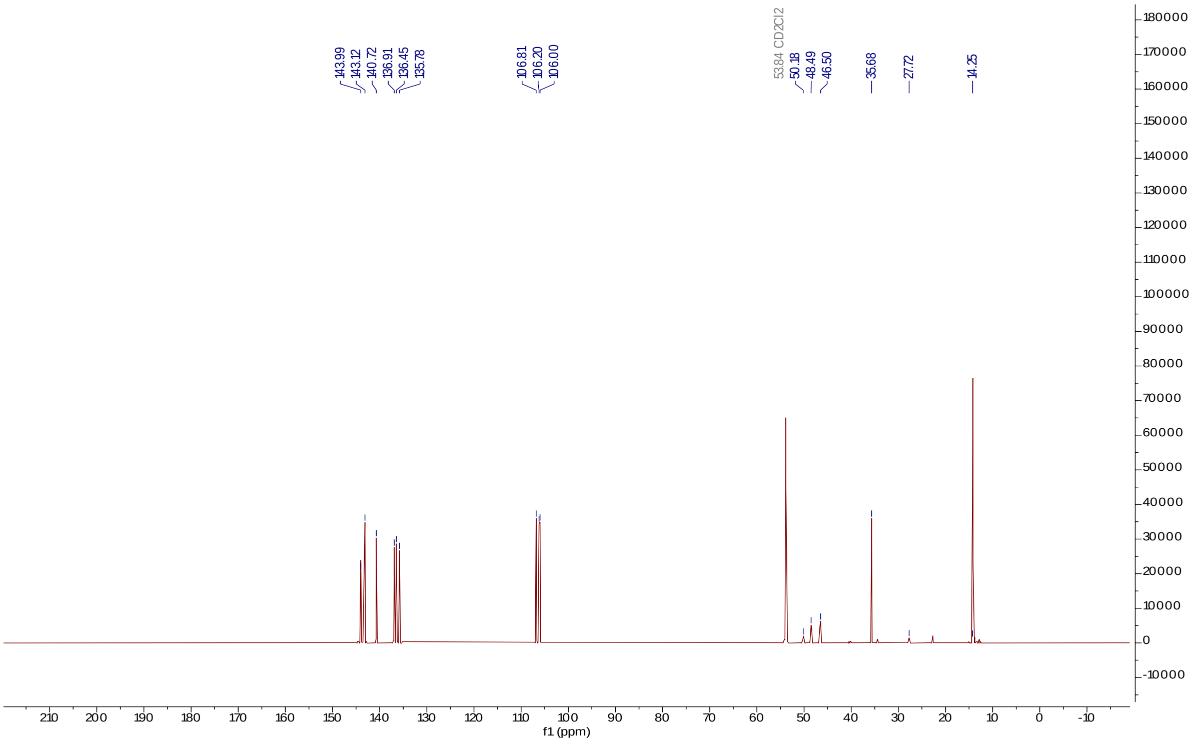

Supplementary Figure 45: ^{1}H NMR, 800 MHz, CD_2Cl_2, 25 °C, ***Rel-(6R)-d$_7$-7***

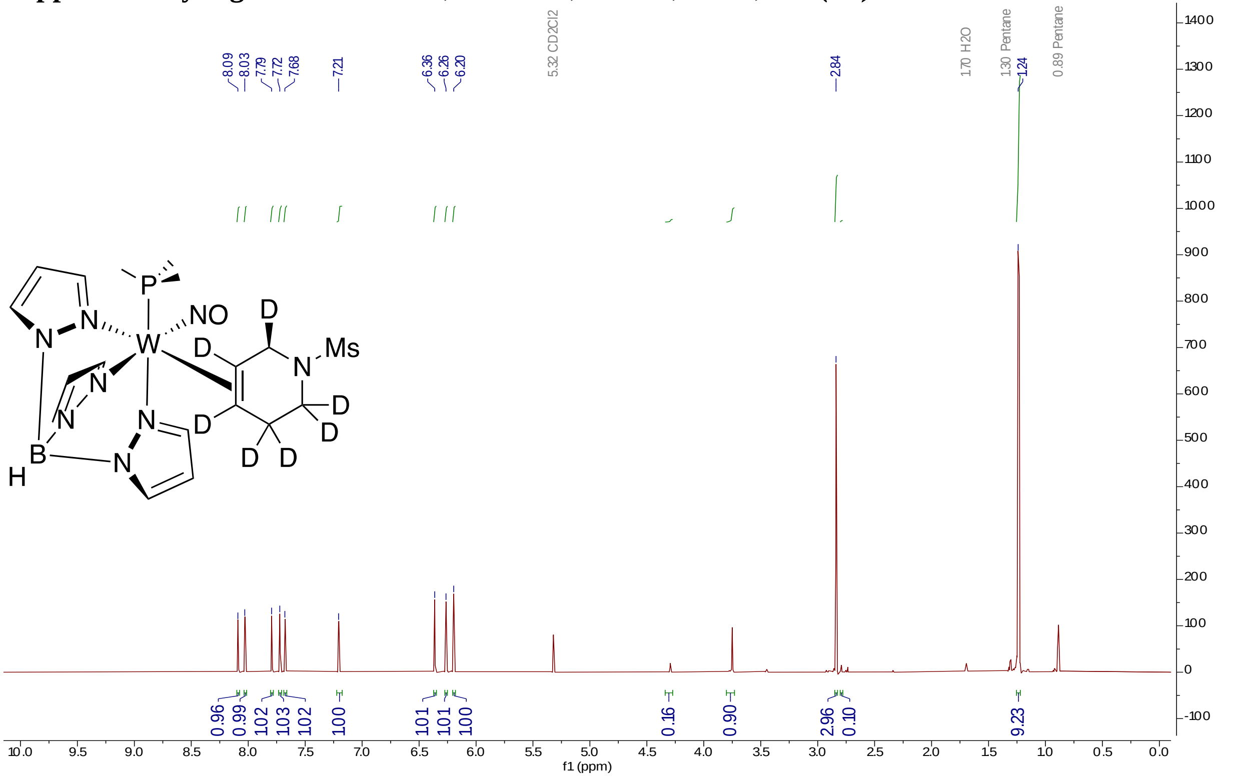

Supplementary Figure 46: ^{13}C NMR, 200 MHz, CD_2Cl_2, 25 °C, ***Rel-(6R)-d$_7$-7***

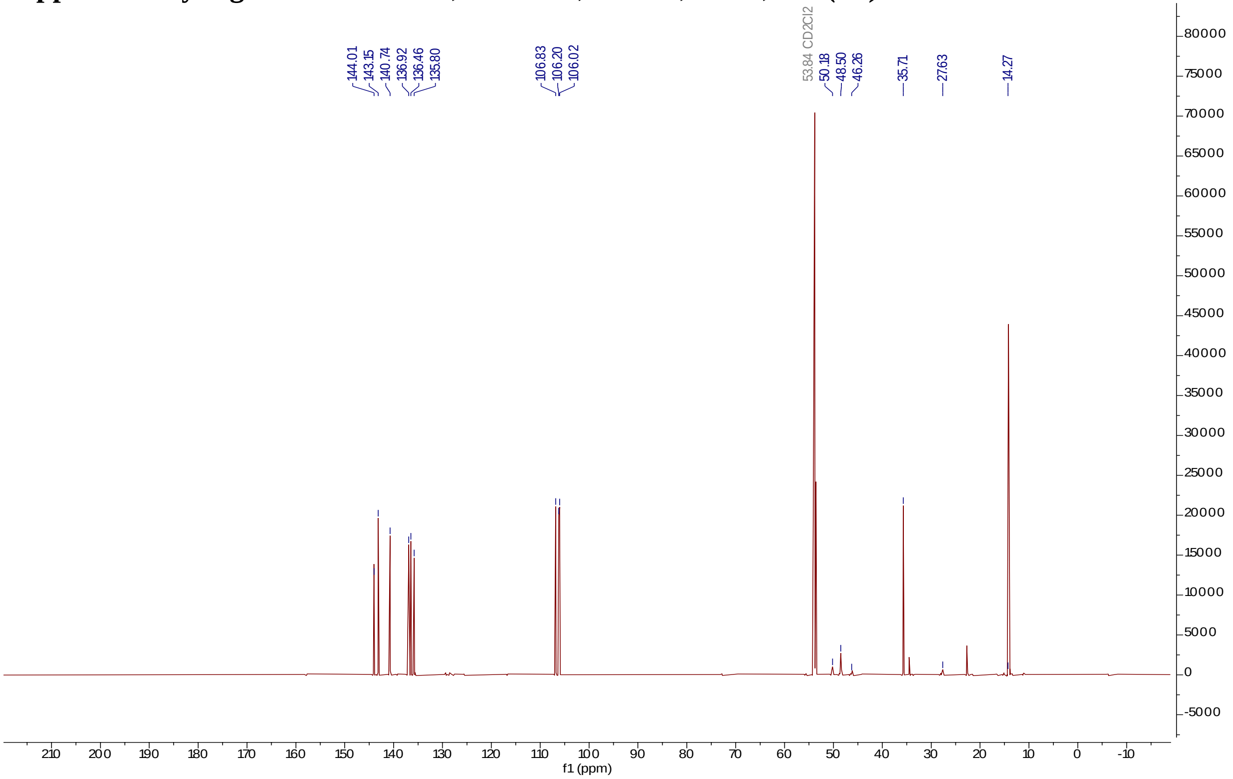

Supplementary Figure 47: ^{1}H NMR, 800 MHz, CD_2Cl_2, 25 °C, ***Rel-(3R)-d$_7$-7***

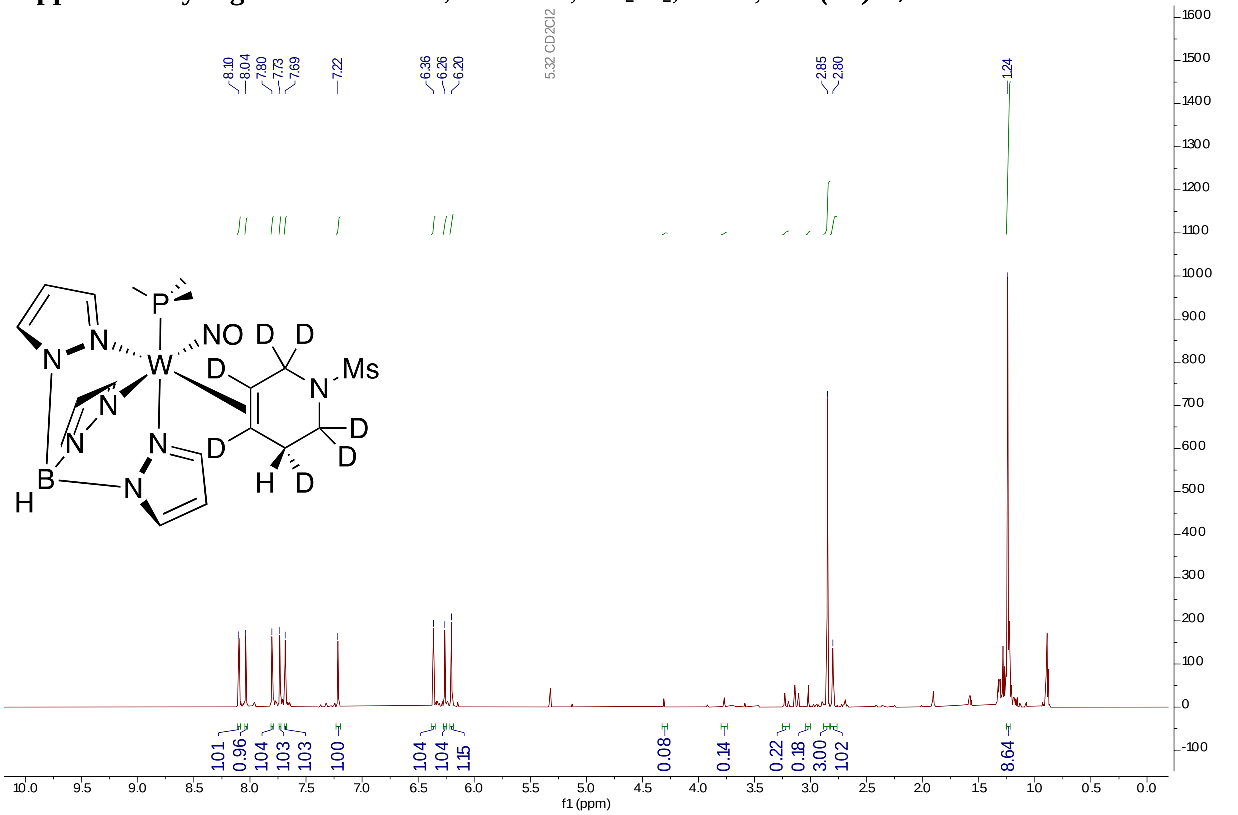

Supplementary Figure 48: ^{13}C NMR, 200 MHz, CD_2Cl_2, 25 °C, ***Rel-(3R)-d$_7$-7***

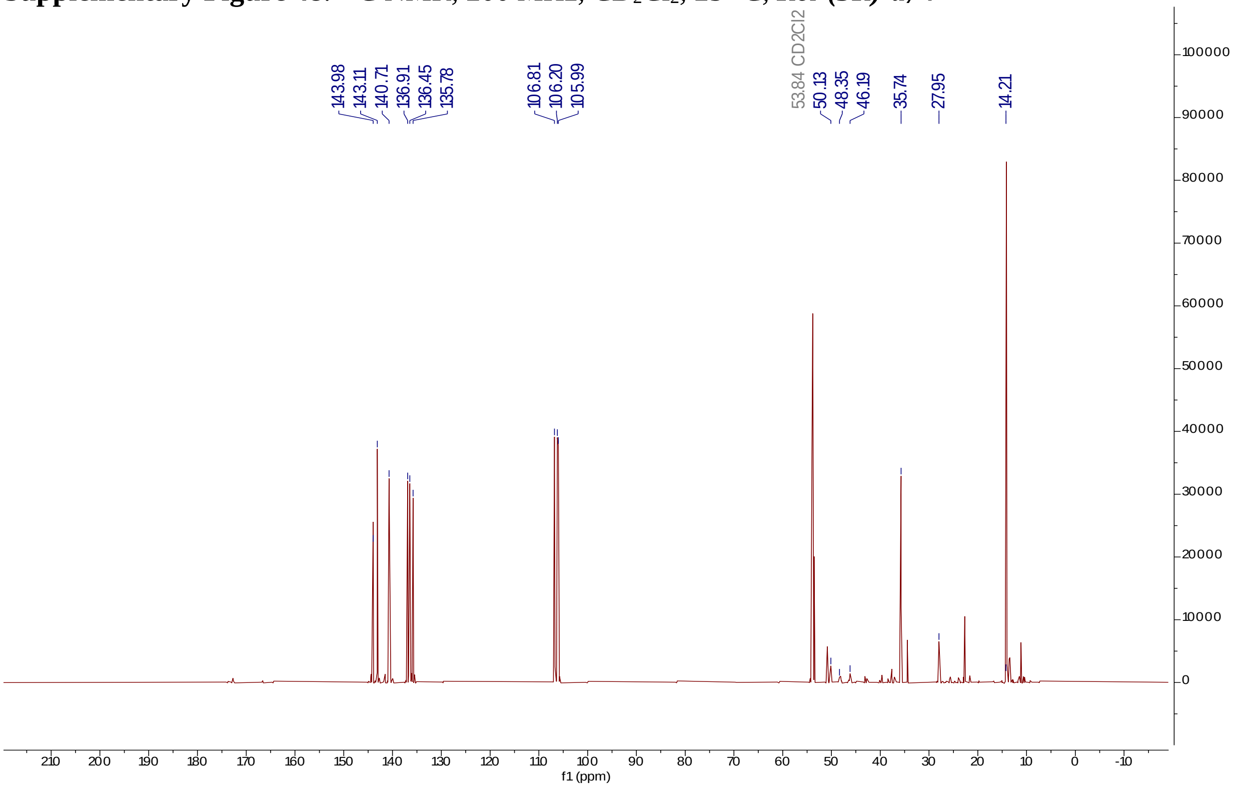

Supplementary Figure 49: ^{1}H NMR, 800 MHz, CD_2Cl_2, 25 °C, ***d*₈-7**

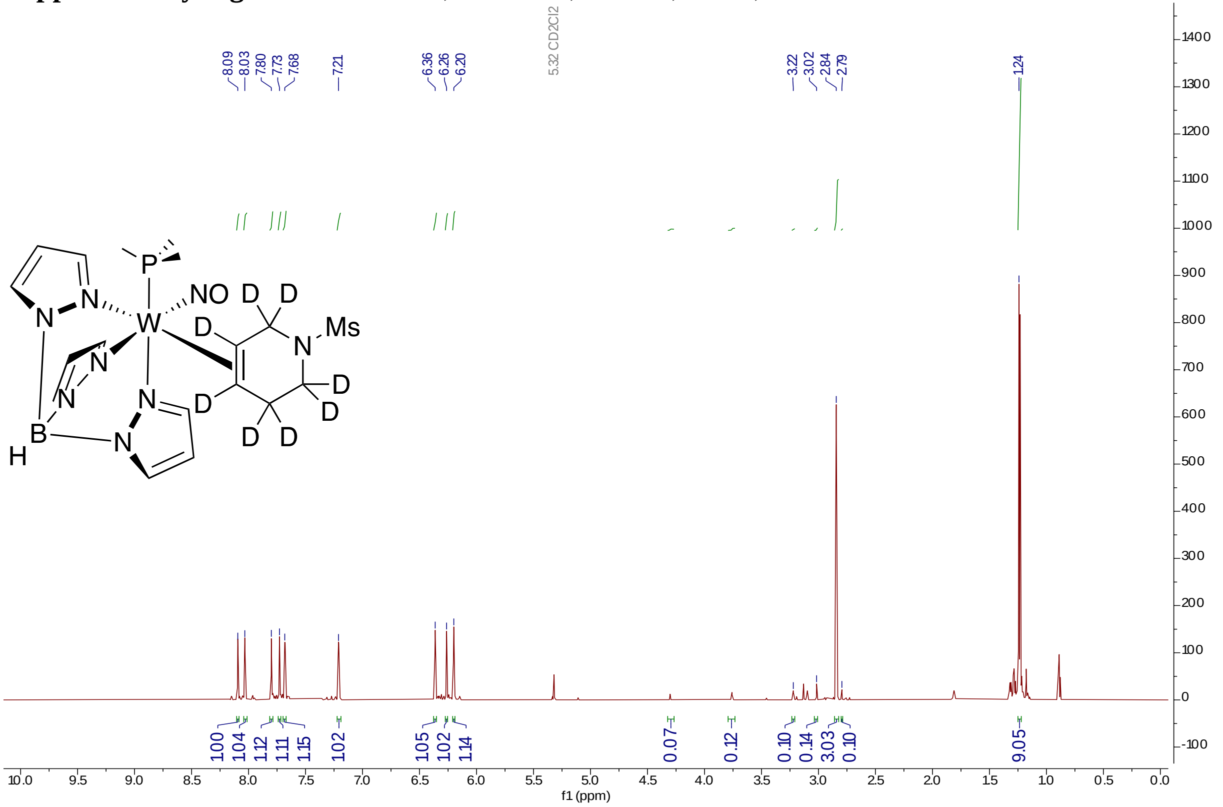

Supplementary Figure 50: ^{13}C NMR, 200 MHz, CD_2Cl_2, 25 °C, ***d*₈-7**

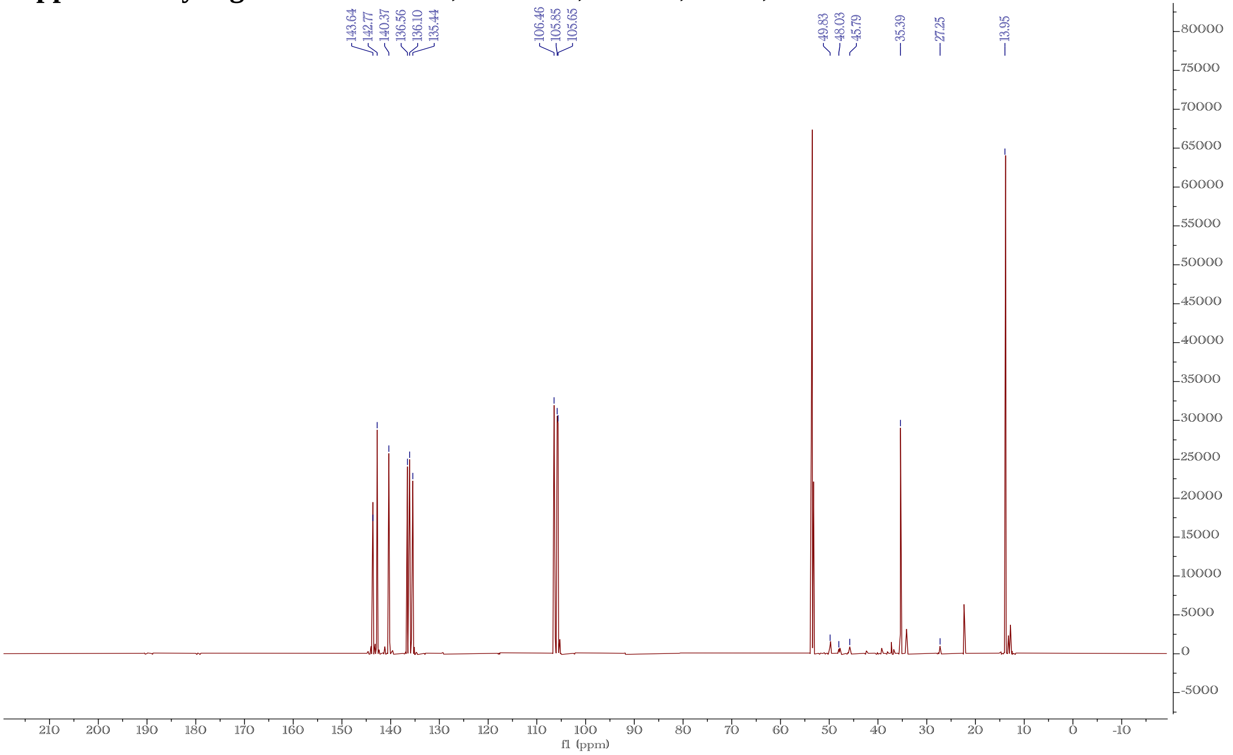

Supplementary Figure 51: ^{1}H NMR, 800 MHz, CD_2Cl_2, 25 °C, **14**

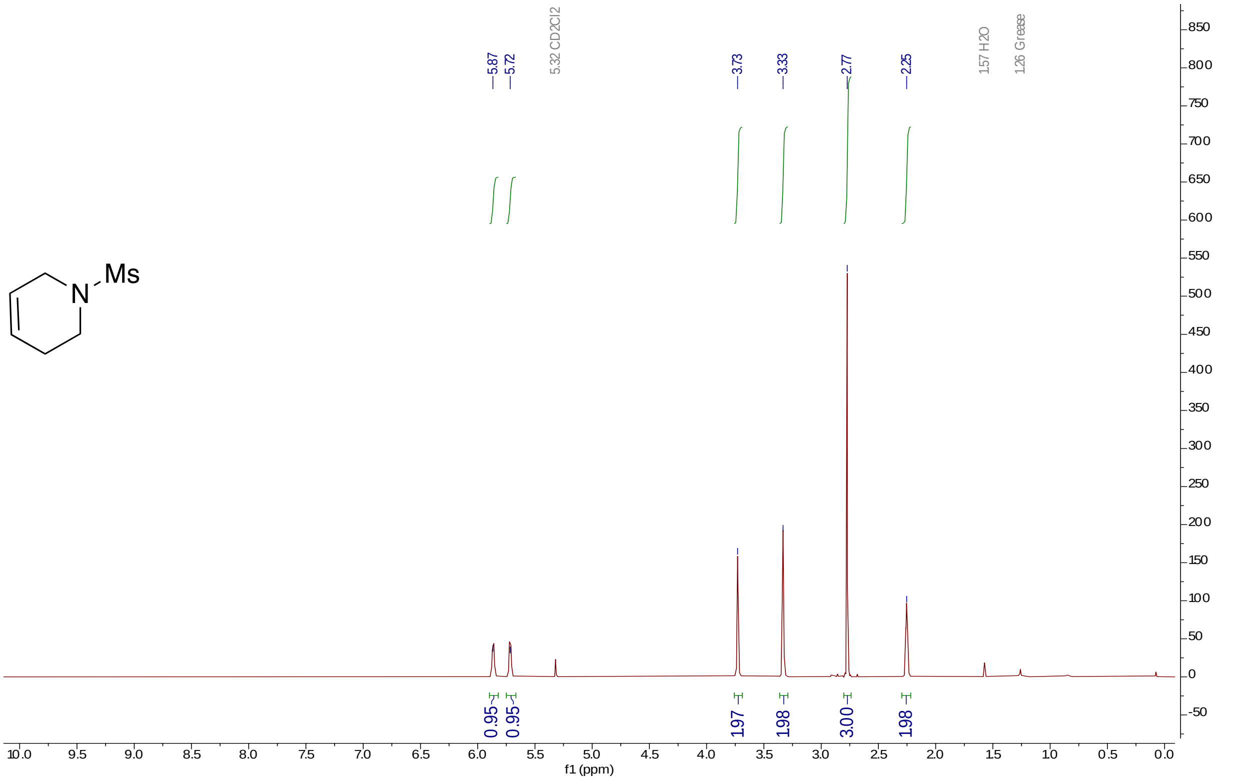

Supplementary Figure 52: ^{13}C NMR, 200 MHz, CD_2Cl_2, 25 °C, **14**

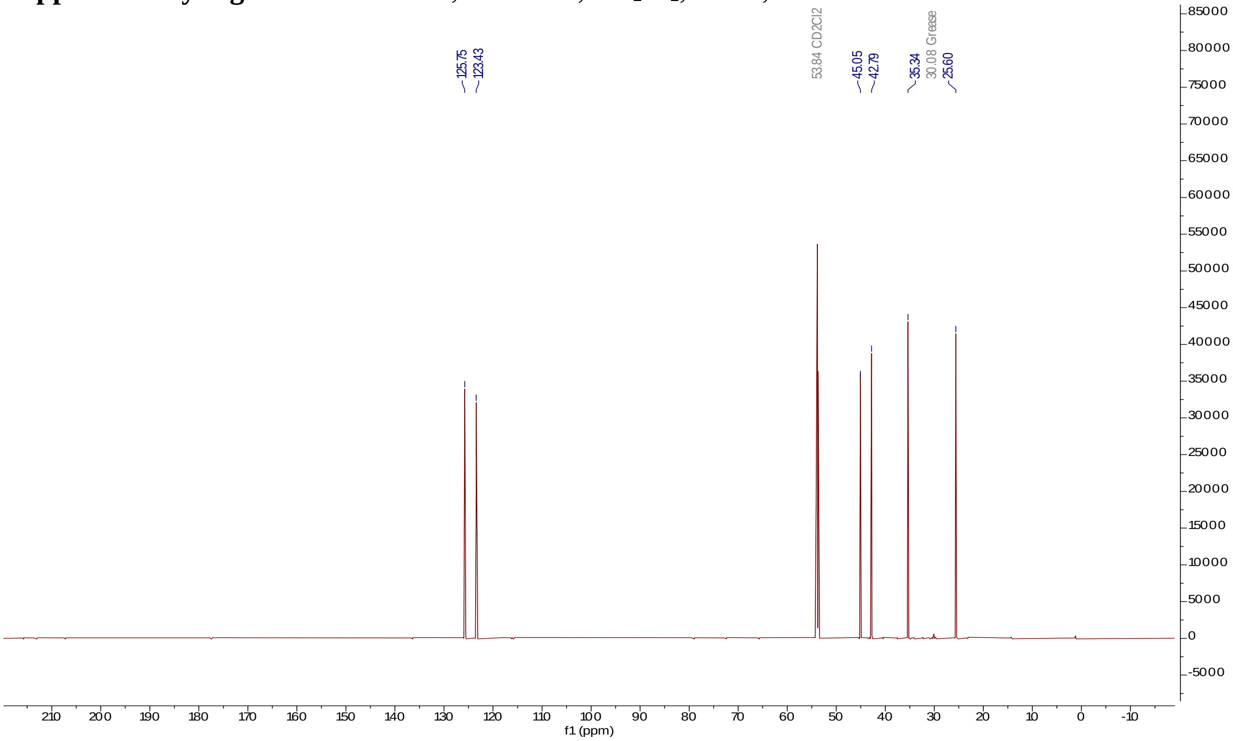

Supplementary Figure 53: ^{1}H NMR, 800 MHz, CD_2Cl_2, 25 °C, ***Rel-(2S)-d₁-14***

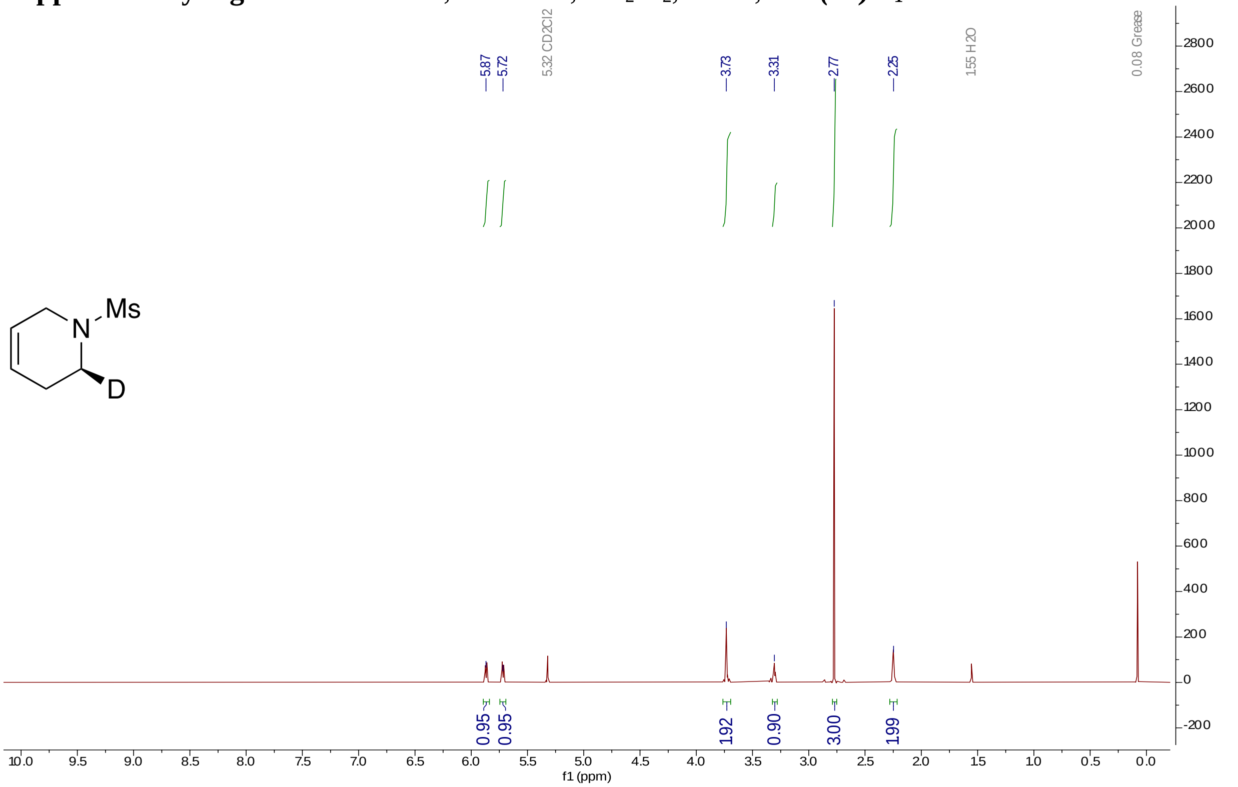

Supplementary Figure 54: ^{13}C NMR, 200 MHz, CD_2Cl_2, 25 °C, ***Rel-(2S)-d₁-14***

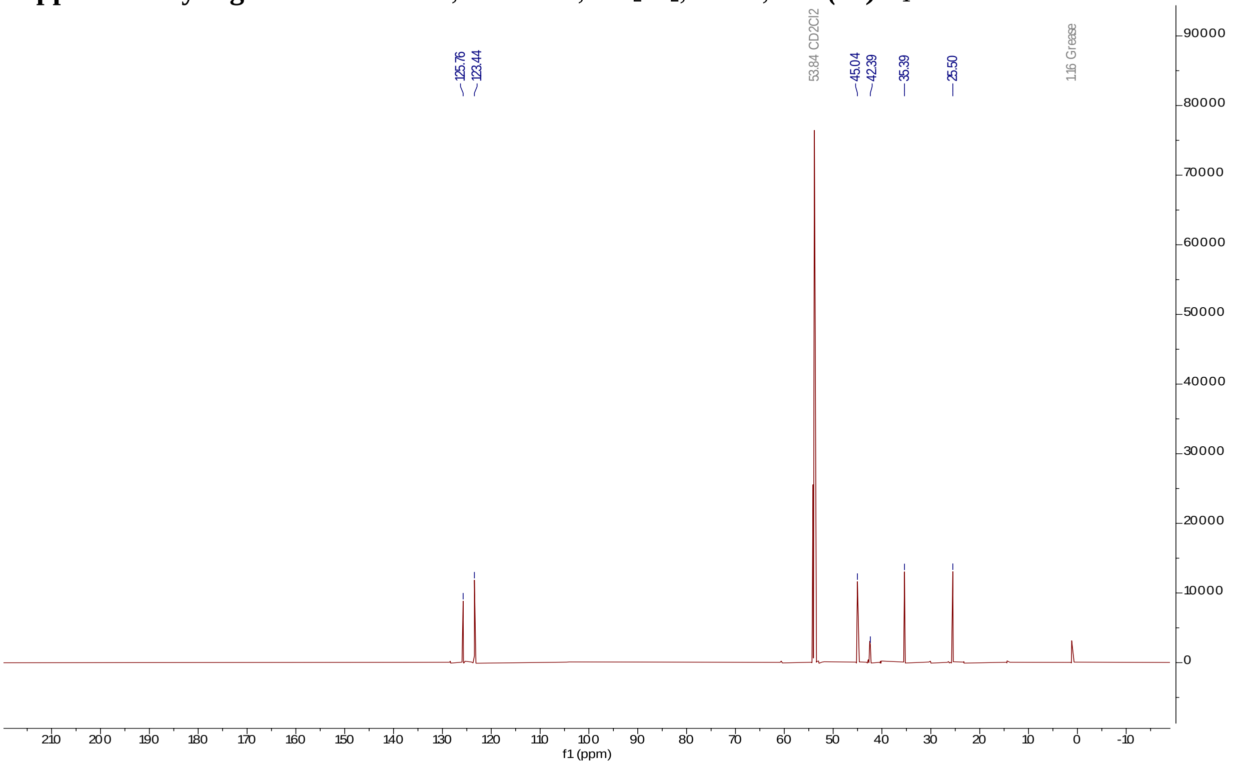

Supplementary Figure 55: ^{1}H NMR, 800 MHz, CD_2Cl_2, 25 °C, ***Rel-(3S)-d₁-14***

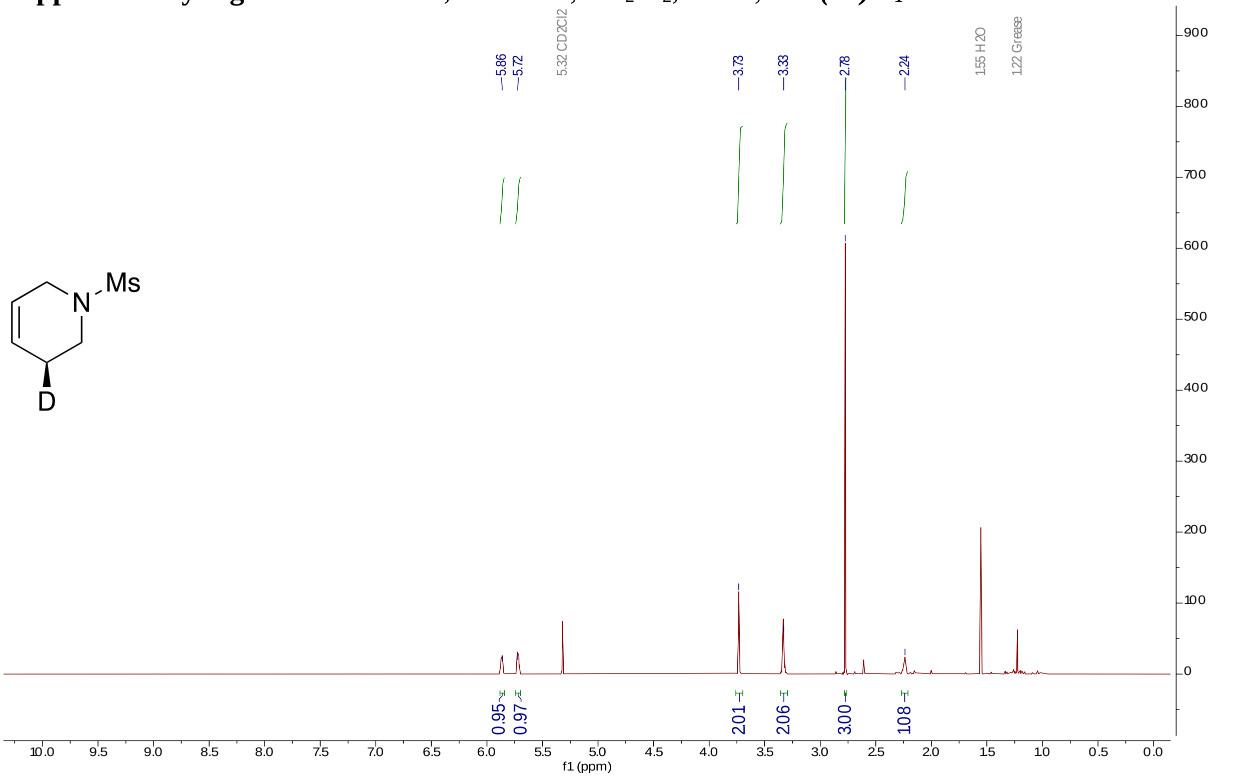

Supplementary Figure 56: ^{13}C NMR, 200 MHz, CD_2Cl_2, 25 °C, ***Rel-(3S)-d₁-14***

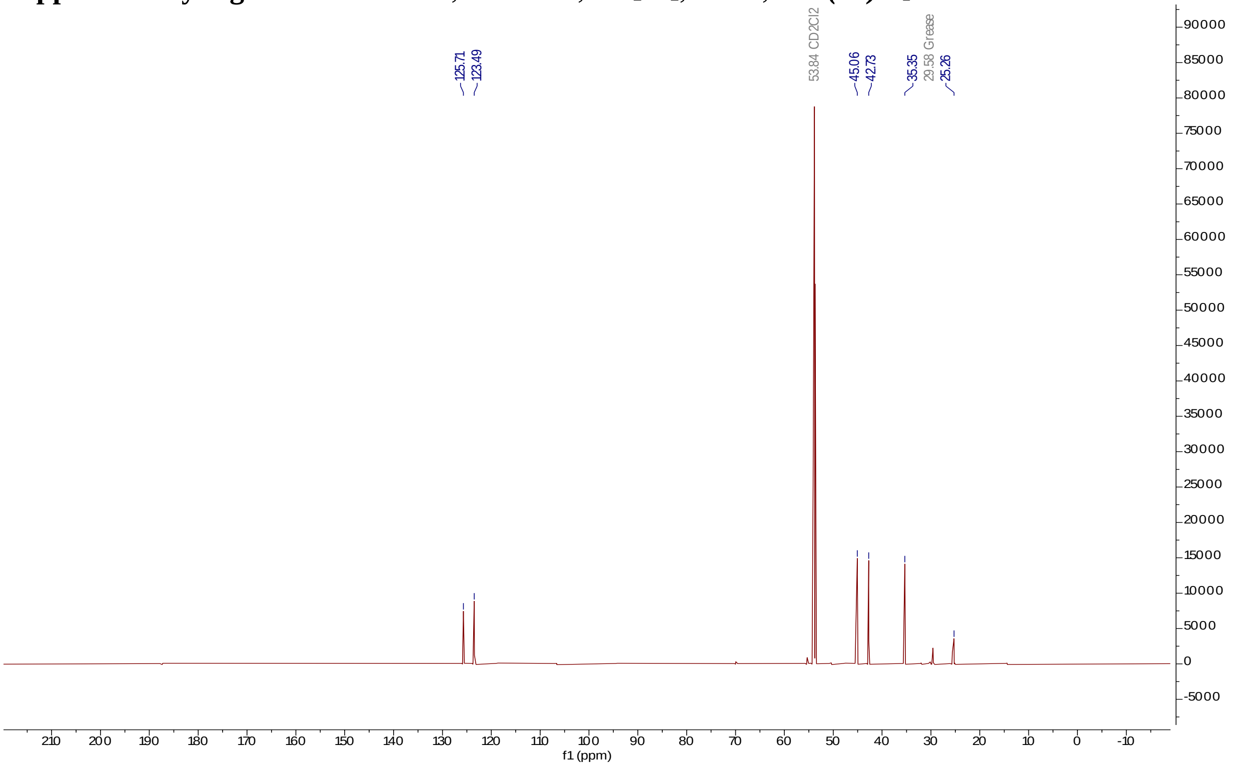

Supplementary Figure 57: ^{1}H NMR, 800 MHz, CD_2Cl_2, 25 °C, ***Rel-(2S,3S)-d_2-14***

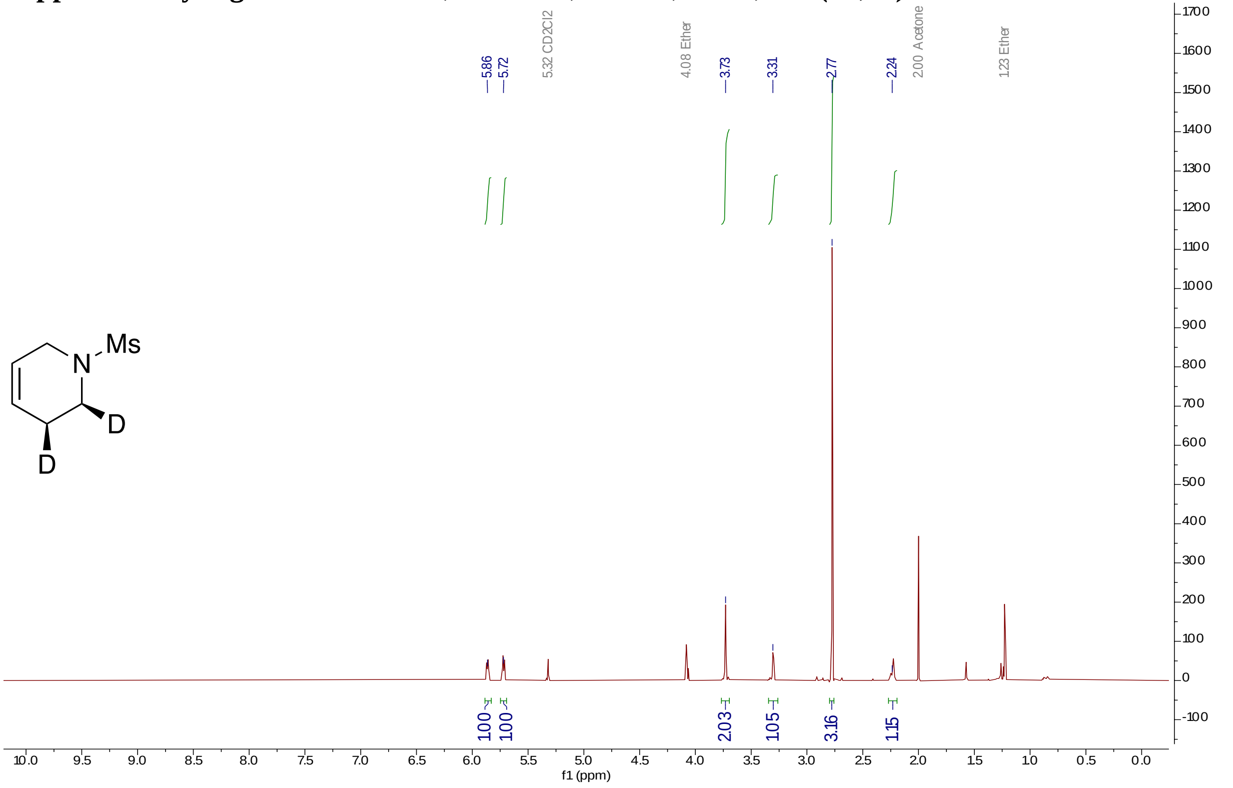

Supplementary Figure 58: ^{13}C NMR, 200 MHz, CD_2Cl_2, 25 °C, ***Rel-(2S,3S)-d_2-14***

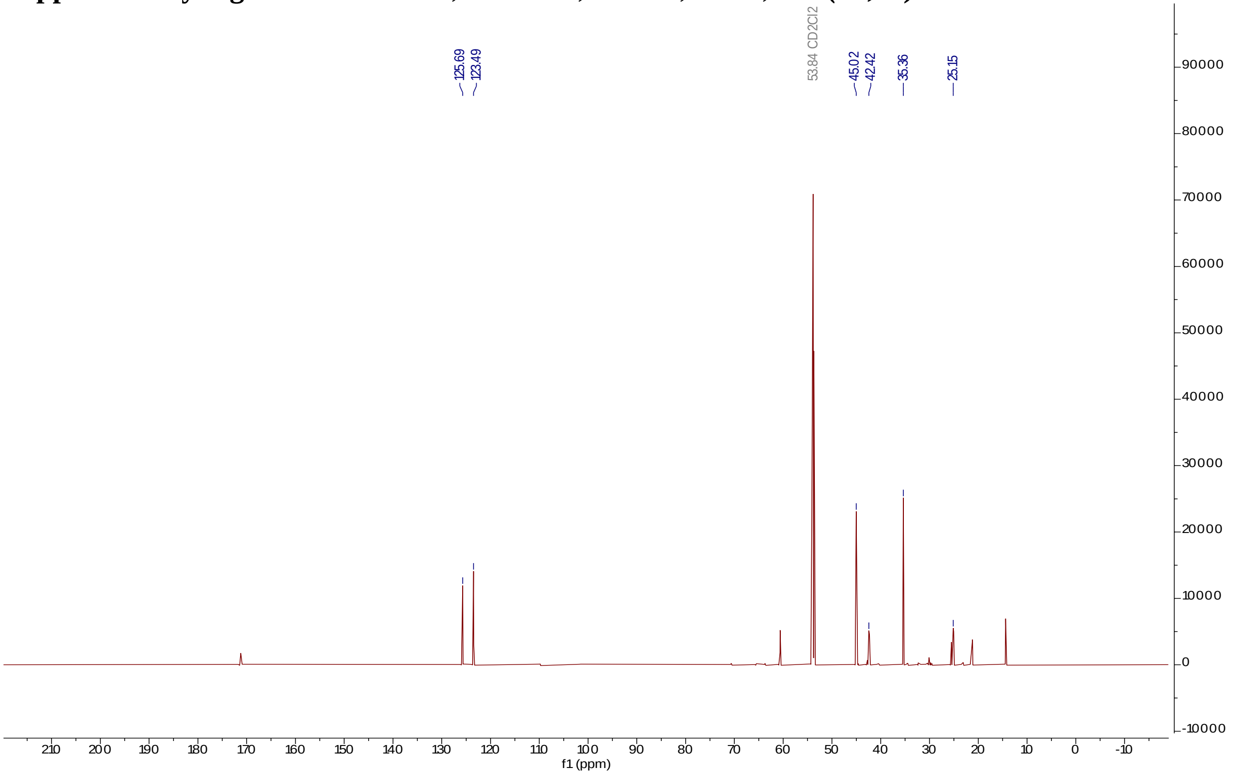

Supplementary Figure 59: ^{1}H NMR, 800 MHz, CD_2Cl_2, 25 °C, ***Rel-(2R,3R,6R)-d$_5$-14***

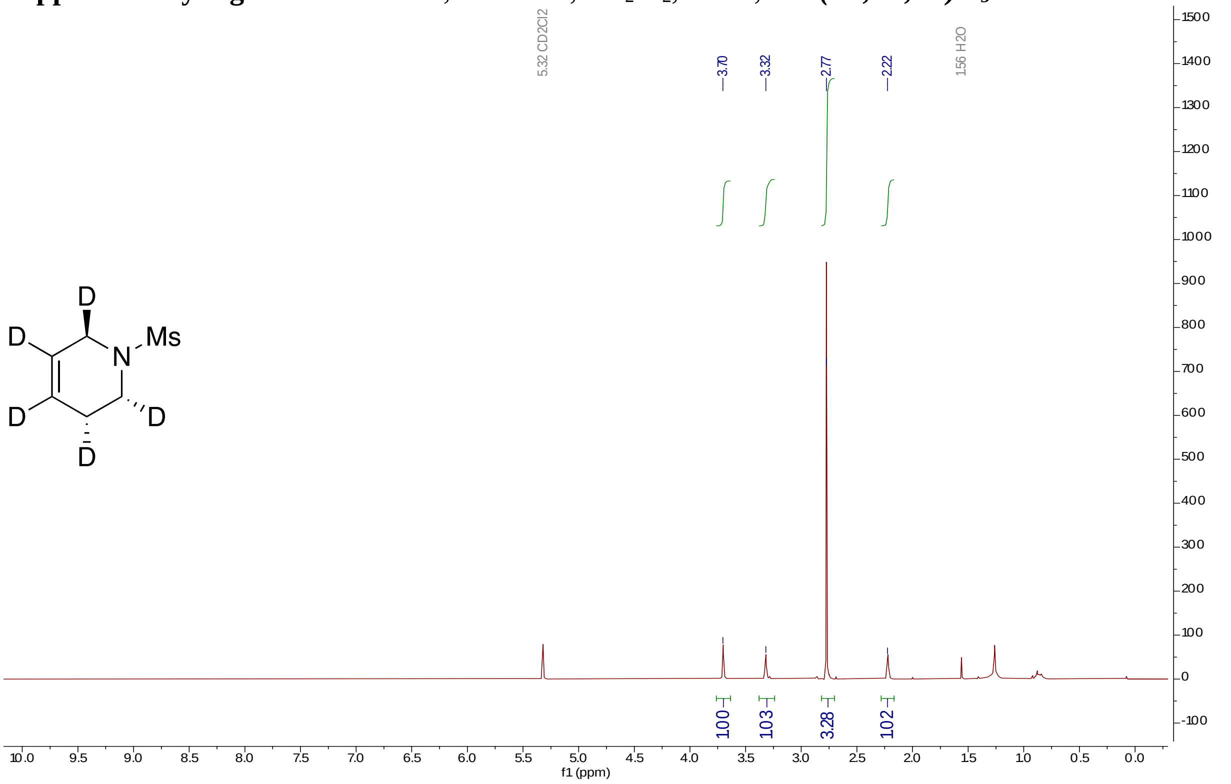

Supplementary Figure 60: ^{13}C NMR, 200 MHz, CD_2Cl_2, 25 °C, ***Rel-(2R,3R,6R)-d$_5$-14***

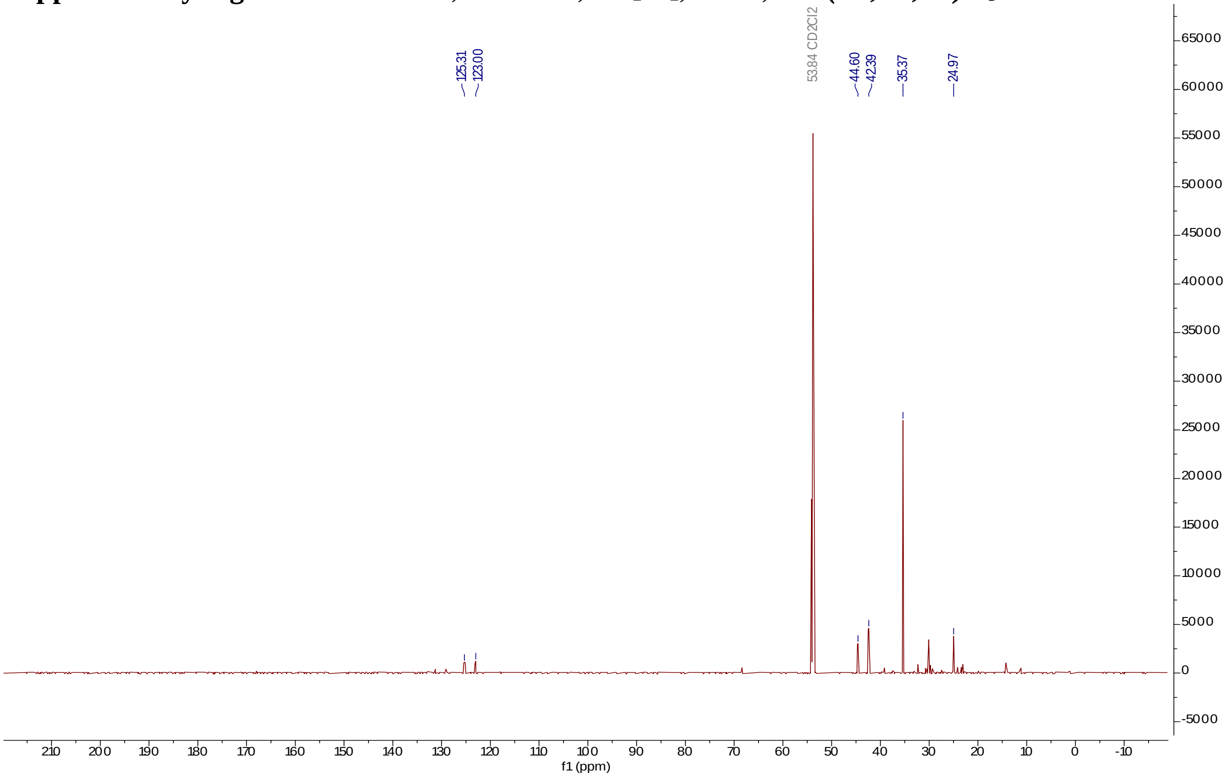

Supplementary Figure 61: ^{1}H NMR, 800 MHz, CD_2Cl_2, 25 °C, ***Rel-(3R,6R)-d$_6$-14***

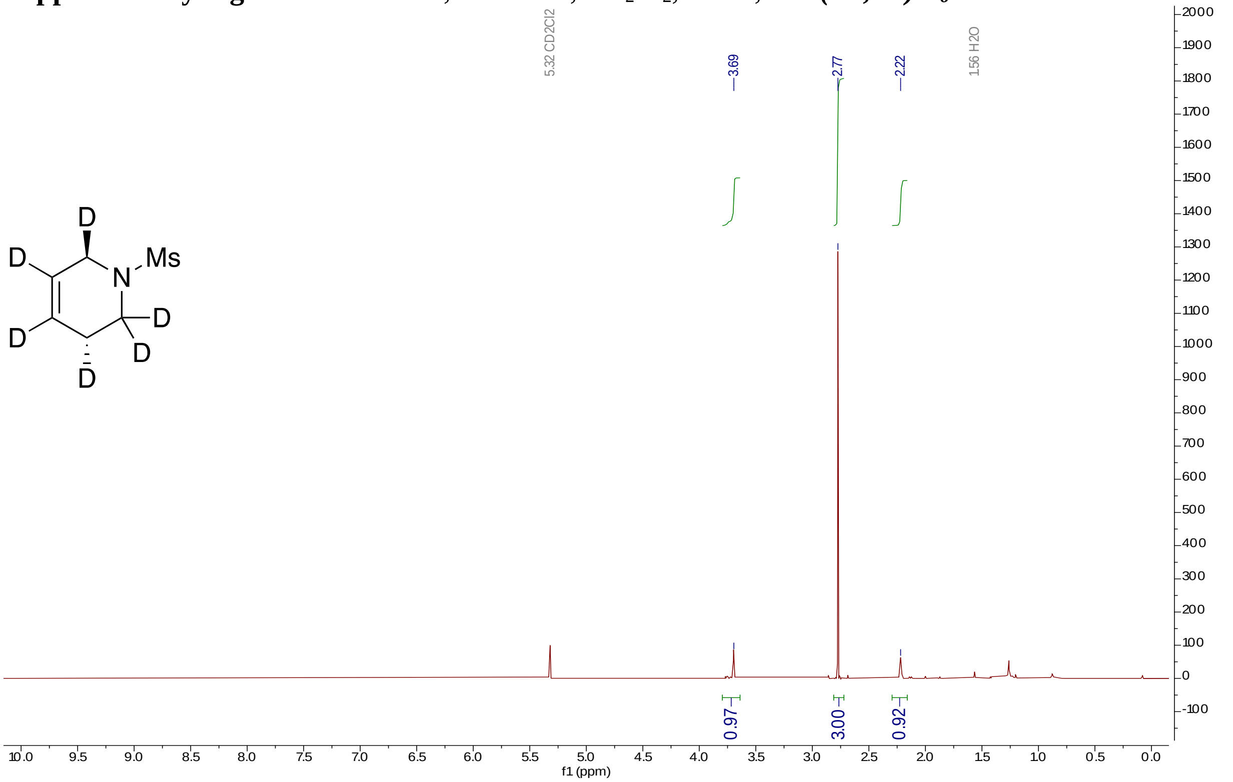

Supplementary Figure 62: ^{13}C NMR, 200 MHz, CD_2Cl_2, 25 °C, ***Rel-(3R,6R)-d$_6$-14***

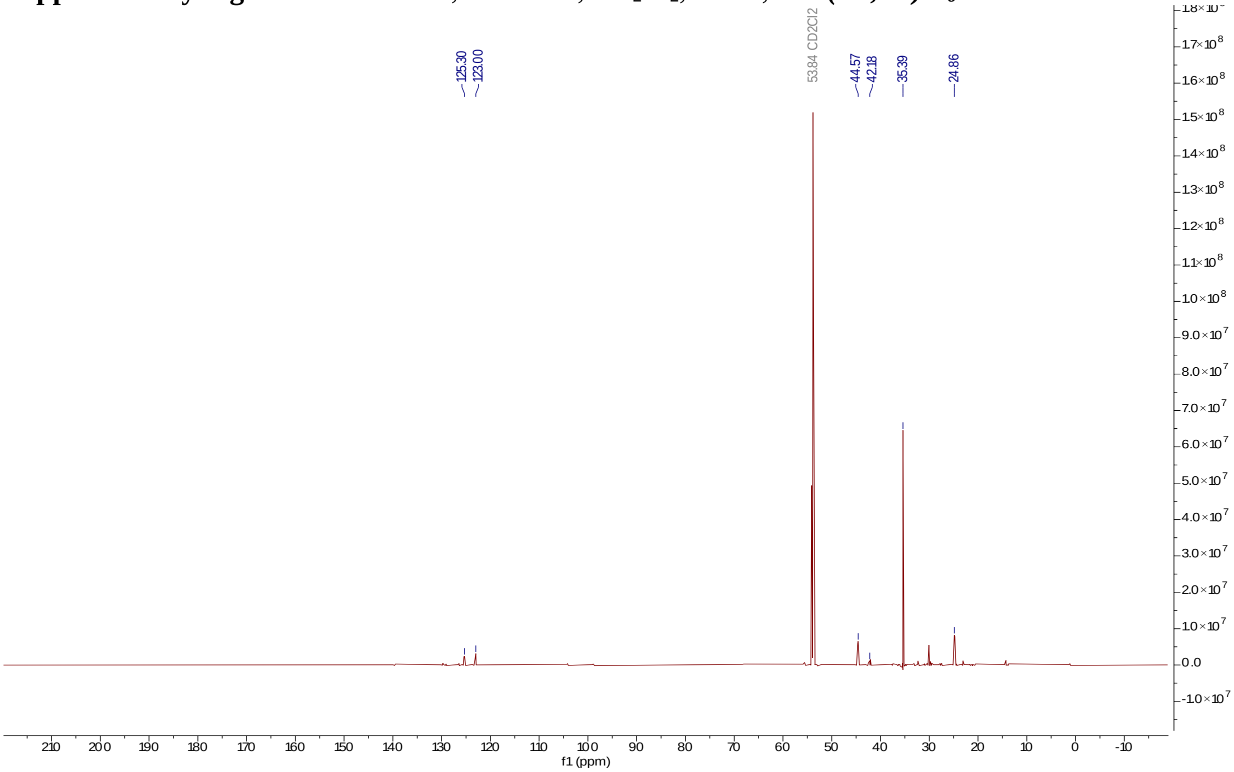

Supplementary Figure 63: ^{1}H NMR, 800 MHz, CD_2Cl_2, 25 °C, ***Rel-(2R,6R)-d_6-14***

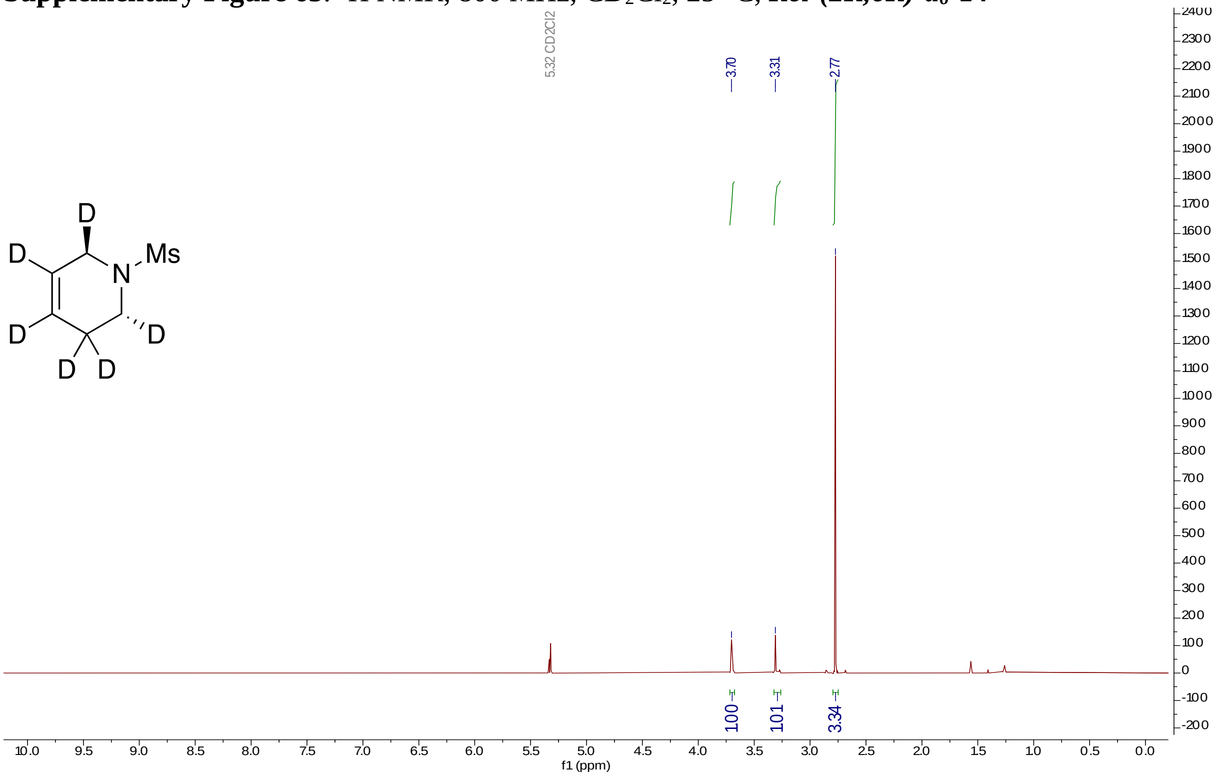

Supplementary Figure 64: ^{13}C NMR, 200 MHz, CD_2Cl_2, 25 °C, ***Rel-(2R,6R)-d_6-14***

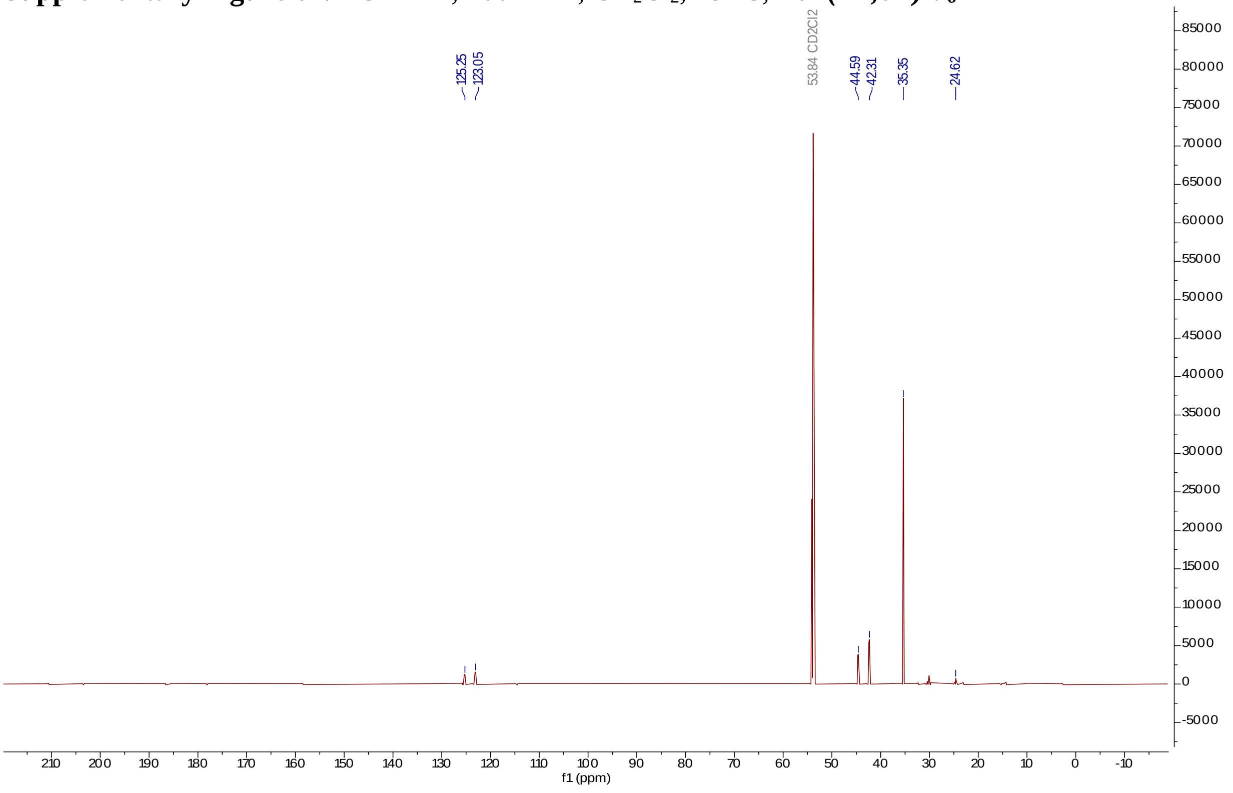

Supplementary Figure 65: ^{1}H NMR, 800 MHz, CD_2Cl_2, 25 °C, ***Rel-(3R)-d$_7$*-14**

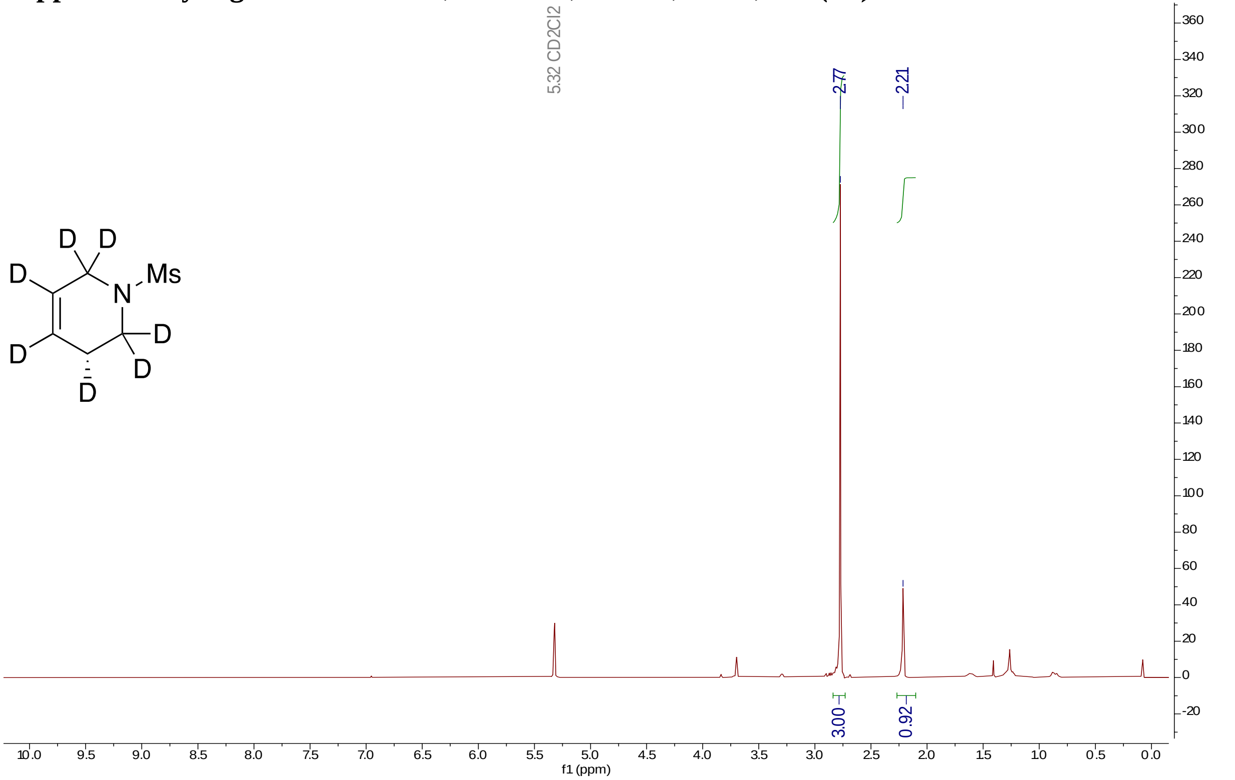

Supplementary Figure 66: ^{13}C NMR, 200 MHz, CD_2Cl_2, 25 °C, ***Rel-(3R)-d$_7$*-14**

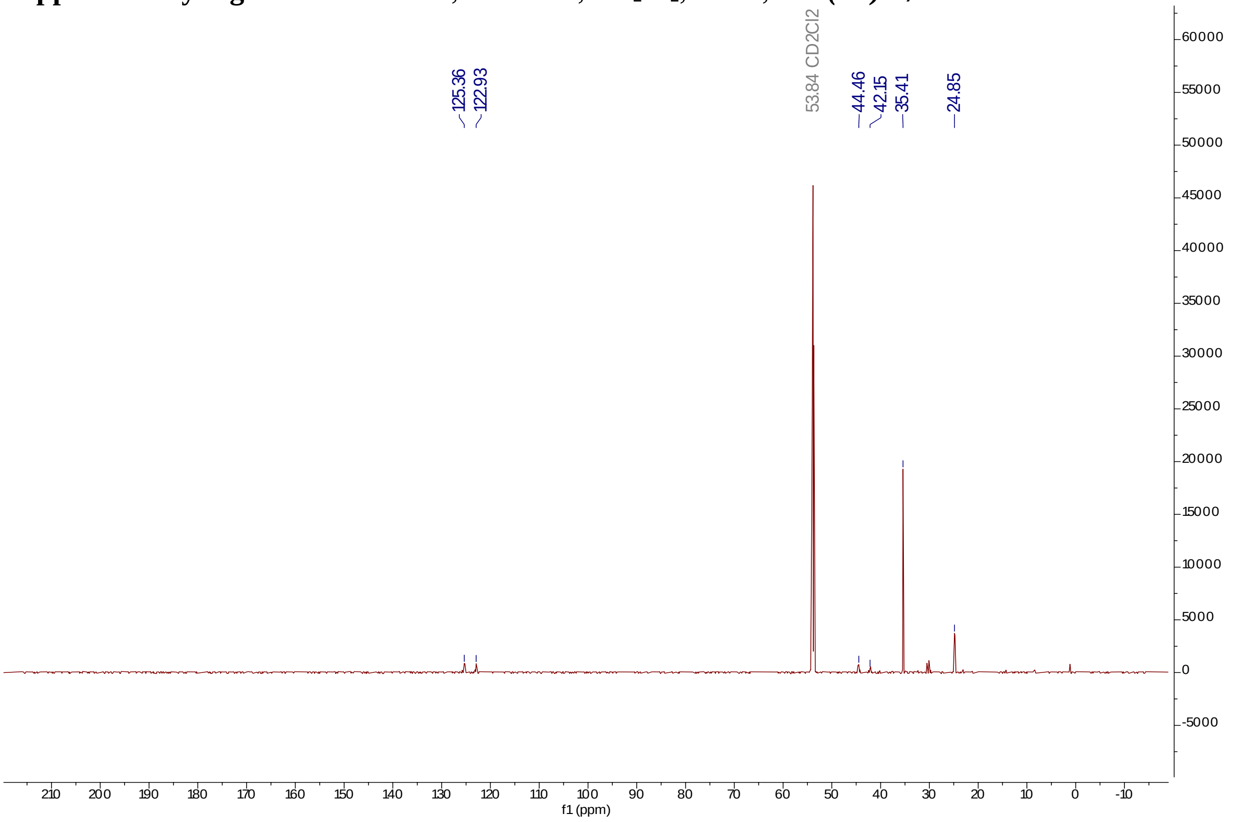

Supplementary Figure 67: ^{1}H NMR, 800 MHz, CD_2Cl_2, 25 °C, ***Rel-(6R)-d$_7$-14***

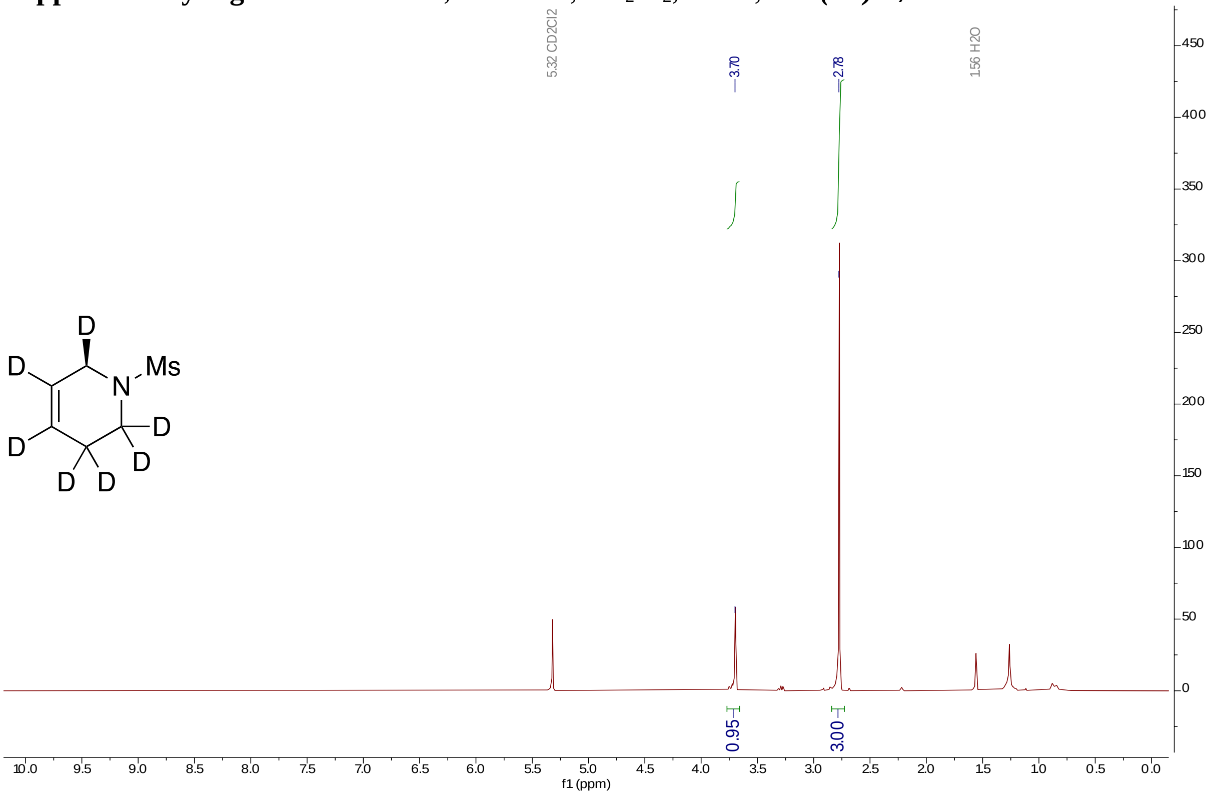

Supplementary Figure 68: ^{13}C NMR, 200 MHz, CD_2Cl_2, 25 °C, ***Rel-(6R)-d$_7$-14***

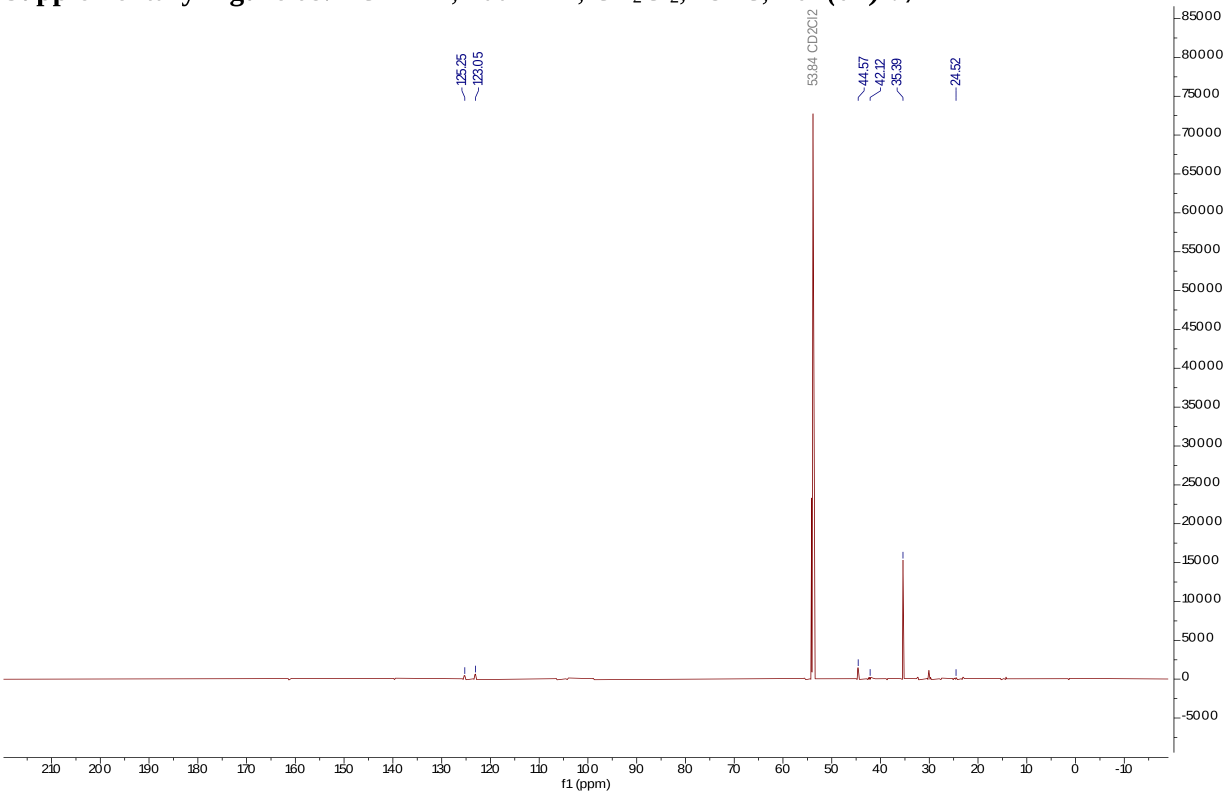

Supplementary Figure 69: ^{1}H NMR, 800 MHz, CD_2Cl_2, 25 °C, ***d*₈-14**

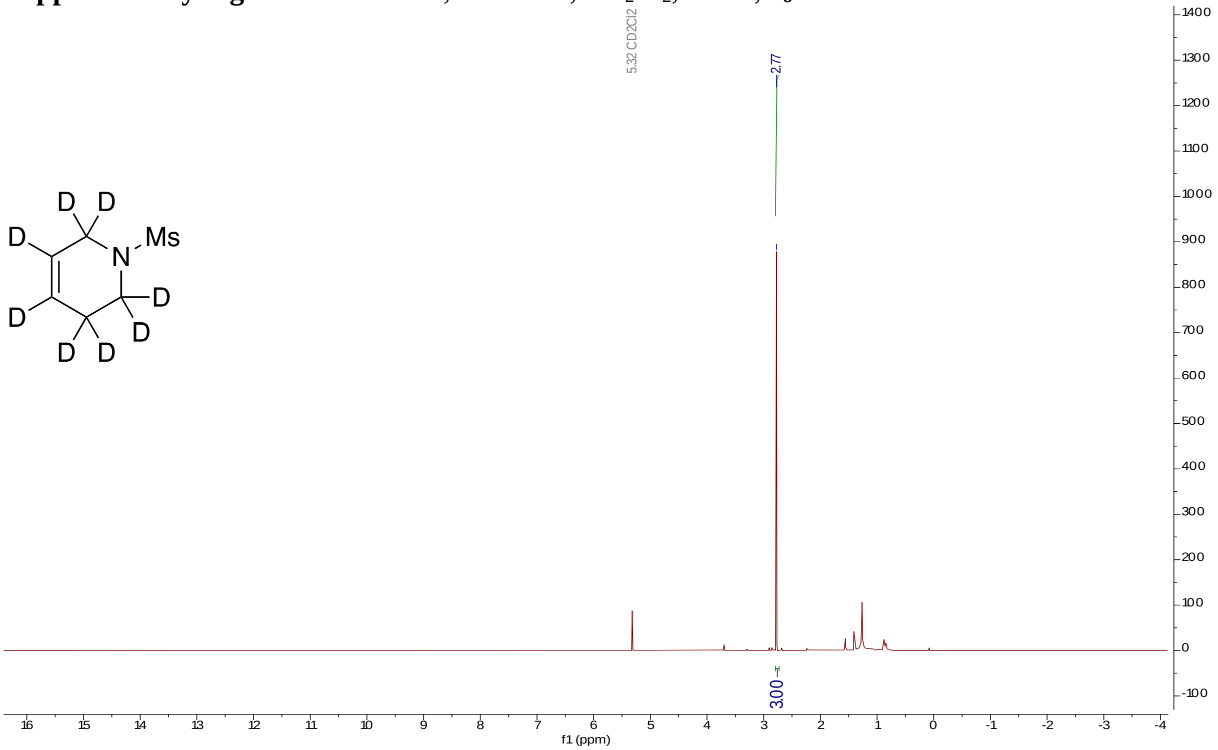

Supplementary Figure 70: ^{13}C NMR, 200 MHz, CD_2Cl_2, 25 °C, ***d*₈-14**

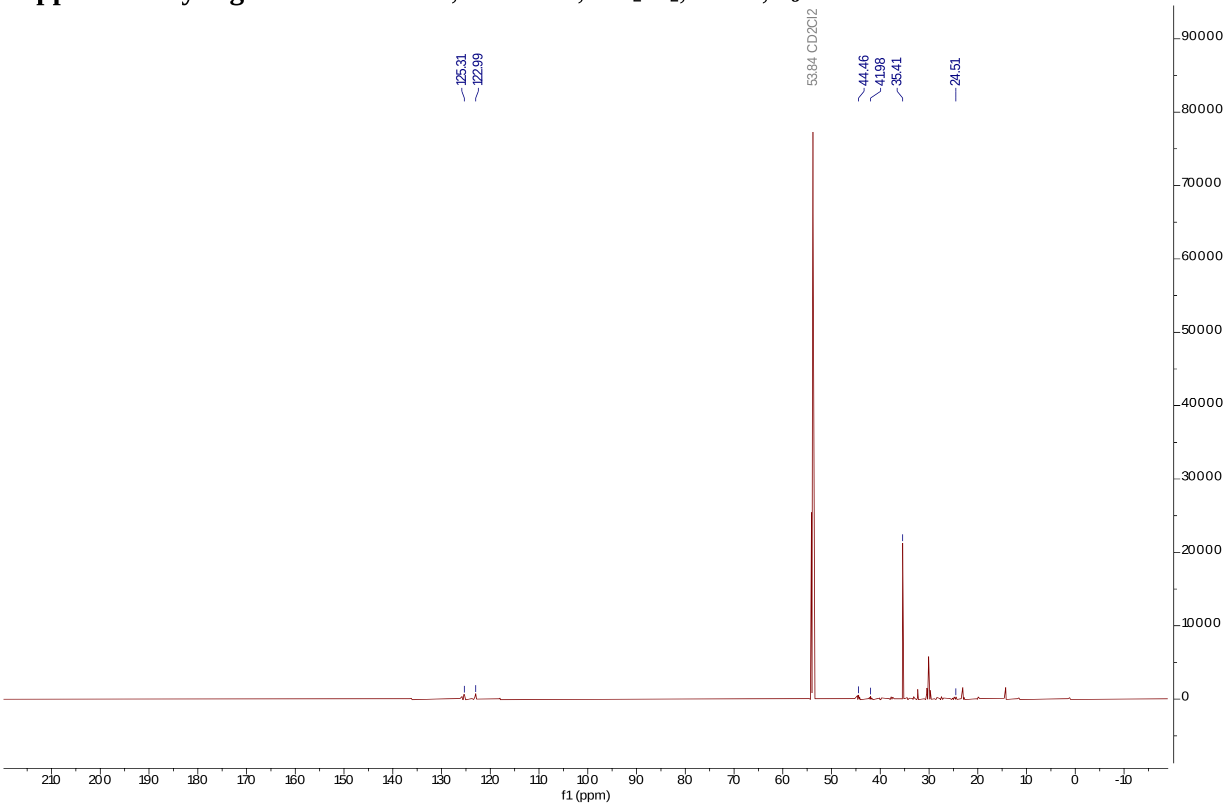

Supplementary Figure 71: ^{1}H NMR, 800 MHz, CD_2Cl_2, 25 °C, **8**

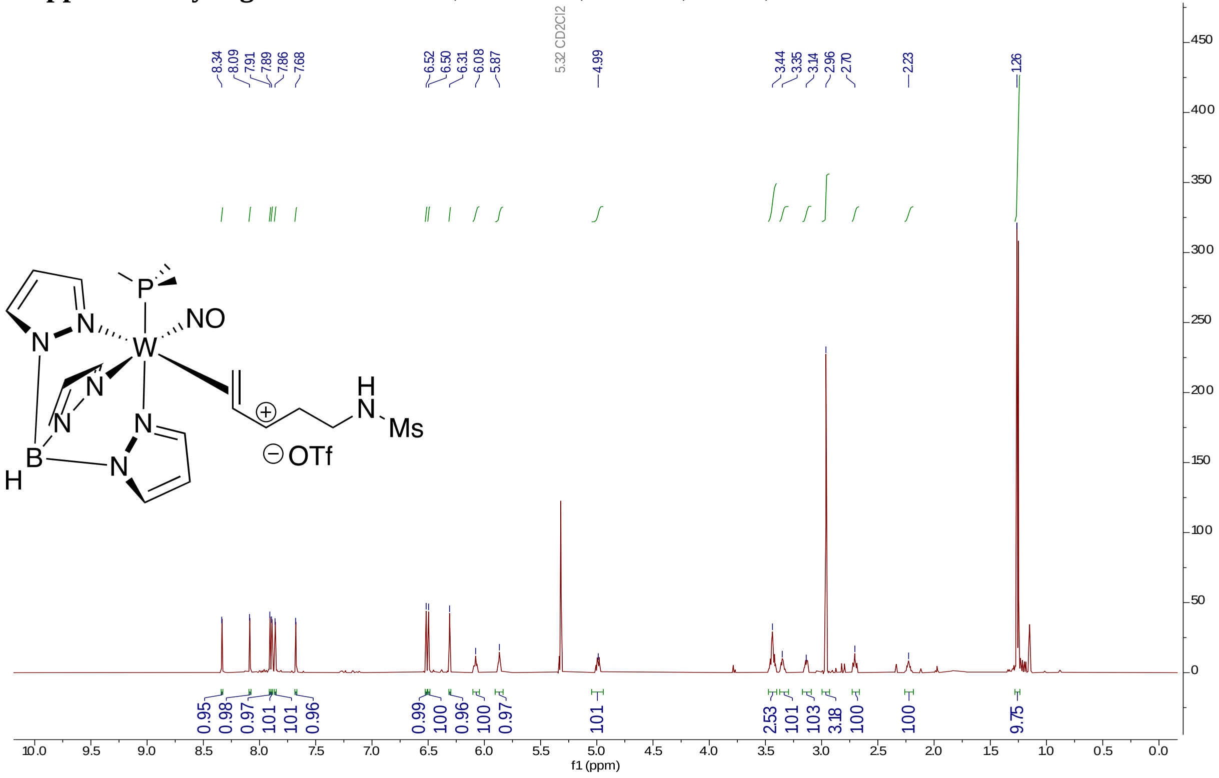

Supplementary Figure 72: ^{13}C NMR, 200 MHz, CD_2Cl_2, 25 °C, **8**

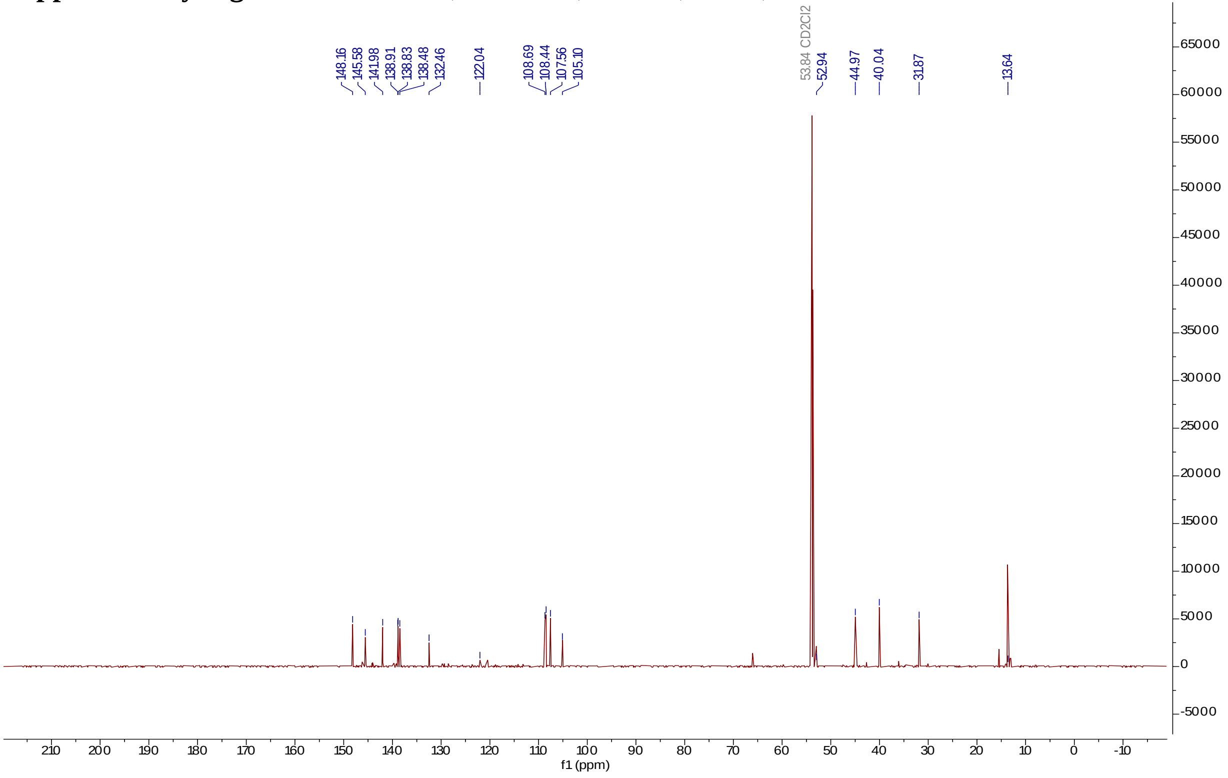

Supplementary Figure 73: NOESY NMR, 800 MHz, CD_2Cl_2, 25 °C, **8**

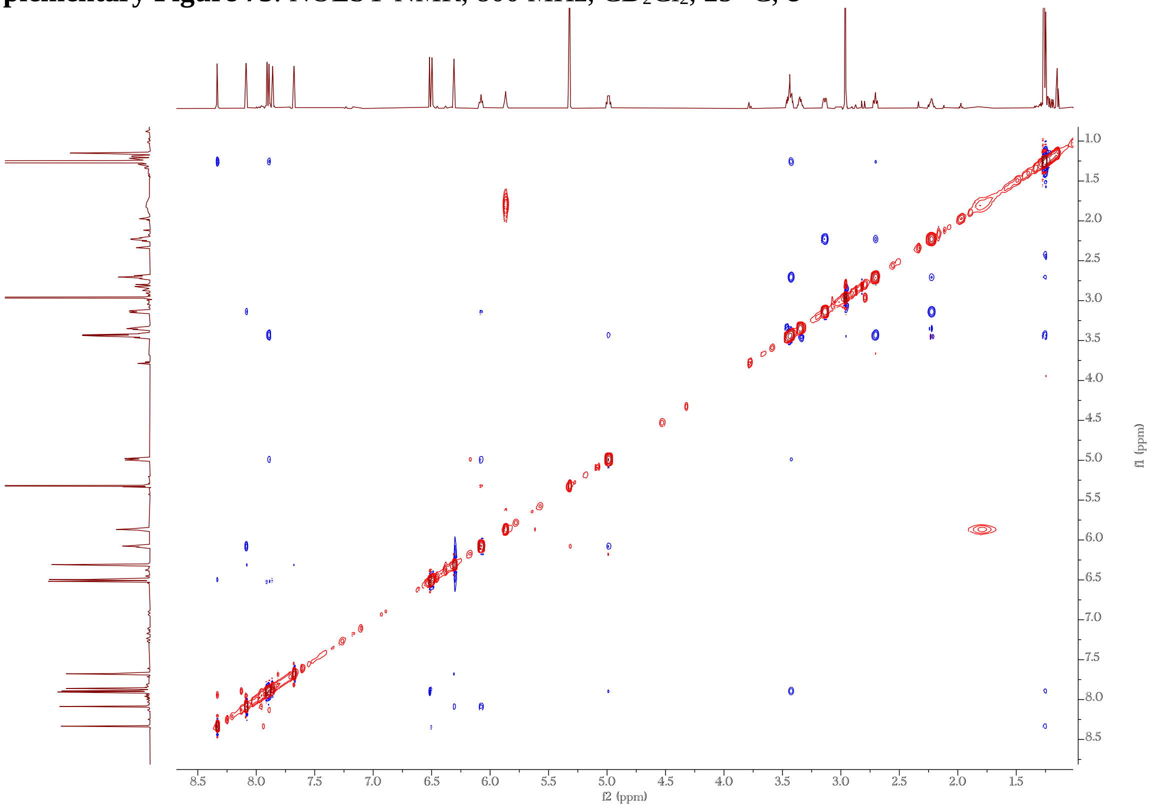

Supplementary Figure 74: COSY NMR, 800 MHz, CD_2Cl_2, 25 °C, **8**

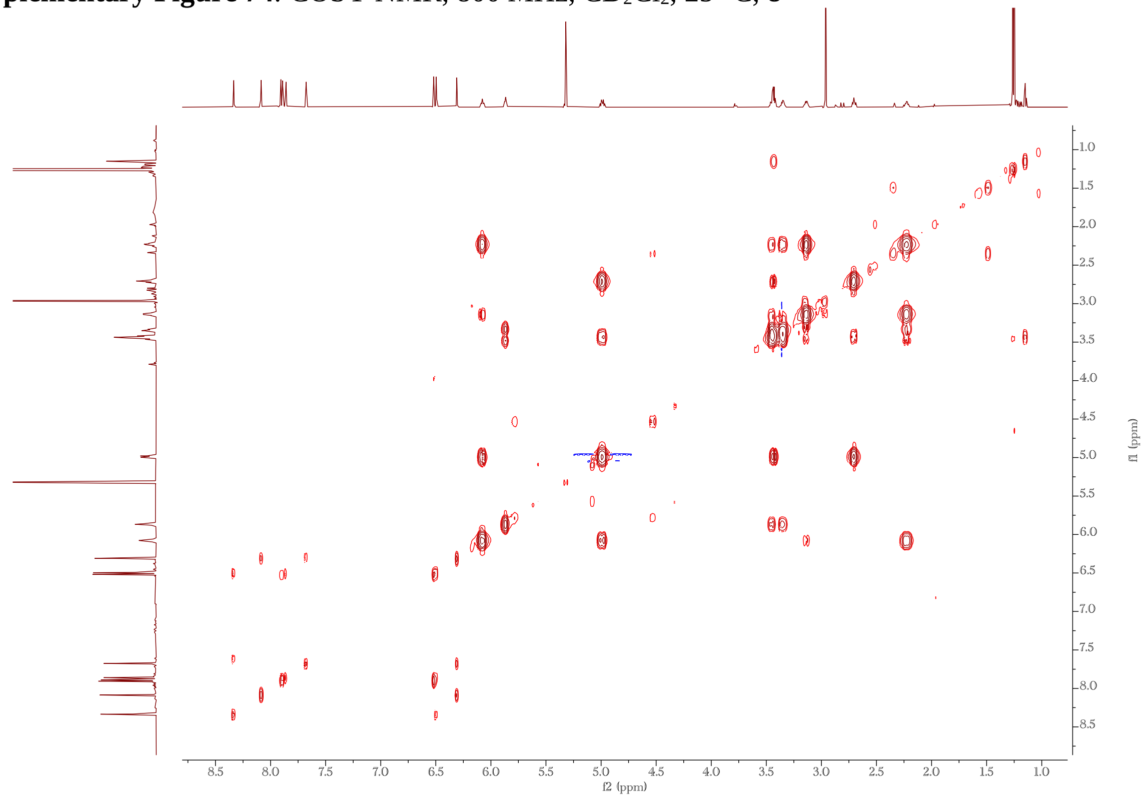

Supplementary Figure 75: HSQC NMR, 800 MHz, CD_2Cl_2, 25 °C, **8**

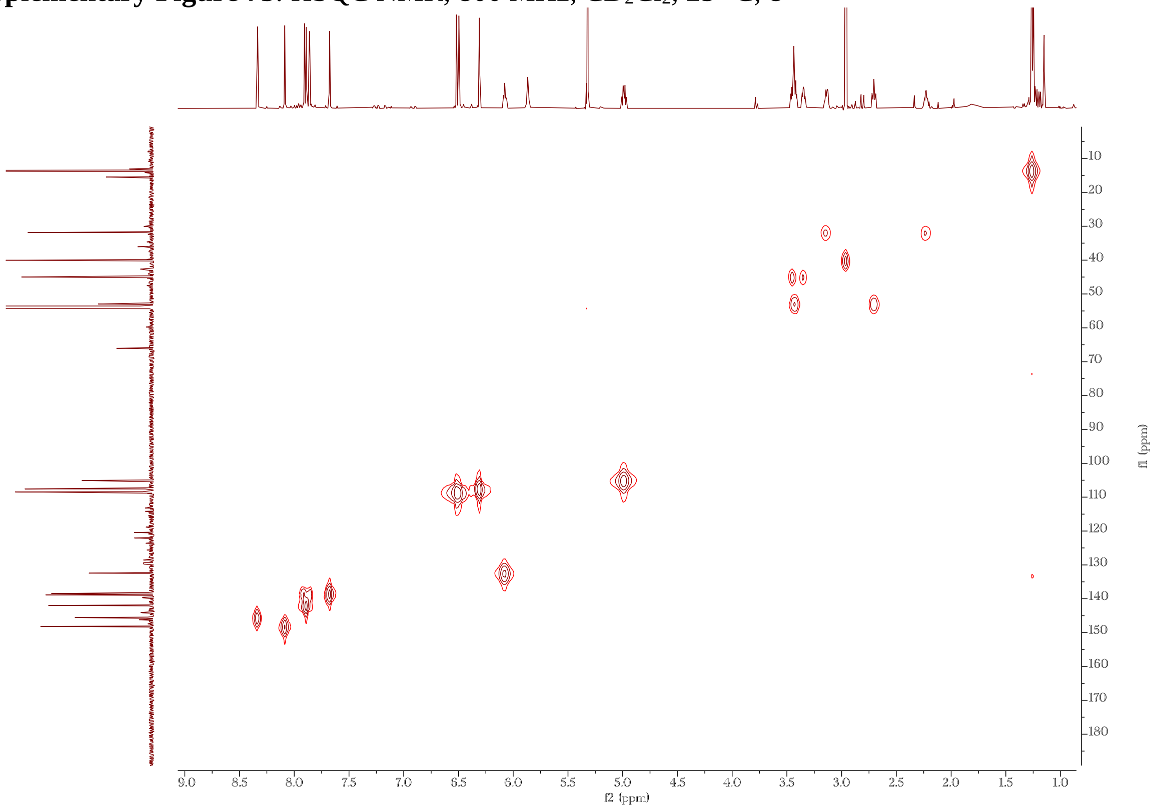

Supplementary Figure 76: HMBC NMR, 800 MHz, CD_2Cl_2, 25 °C, **8**

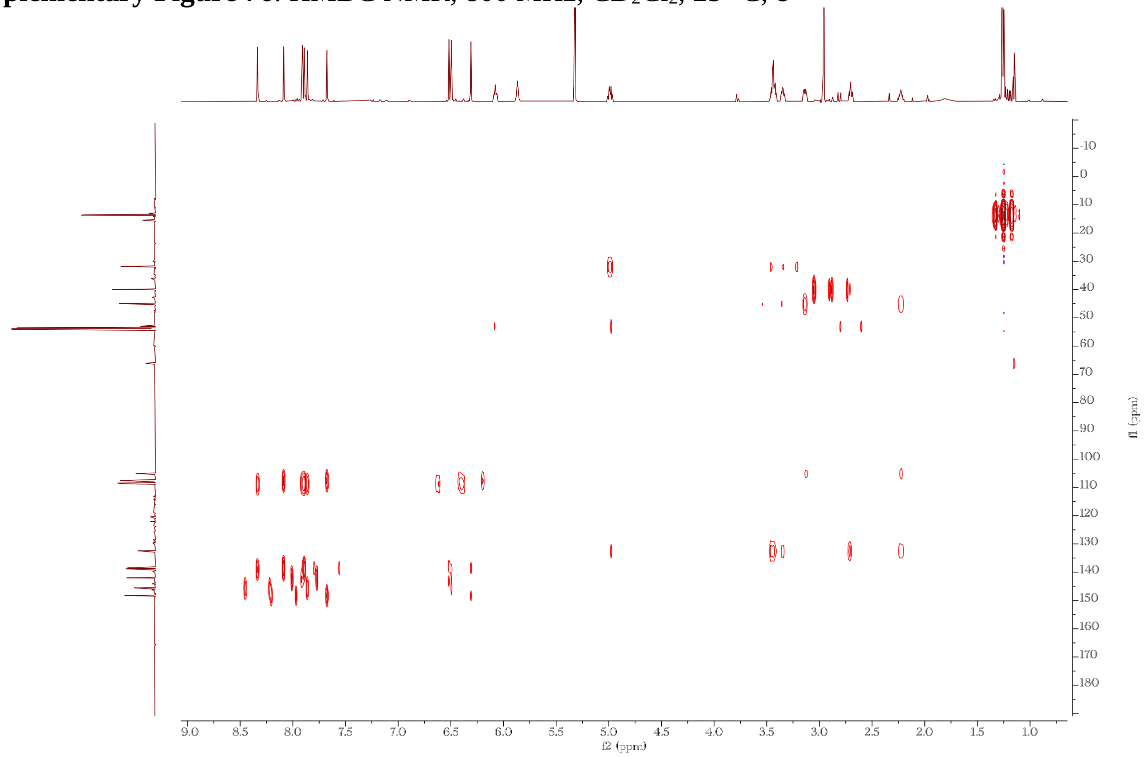

Supplementary Figure 77: ^{1}H NMR, 800 MHz, CD_2Cl_2, 25 °C, **9**

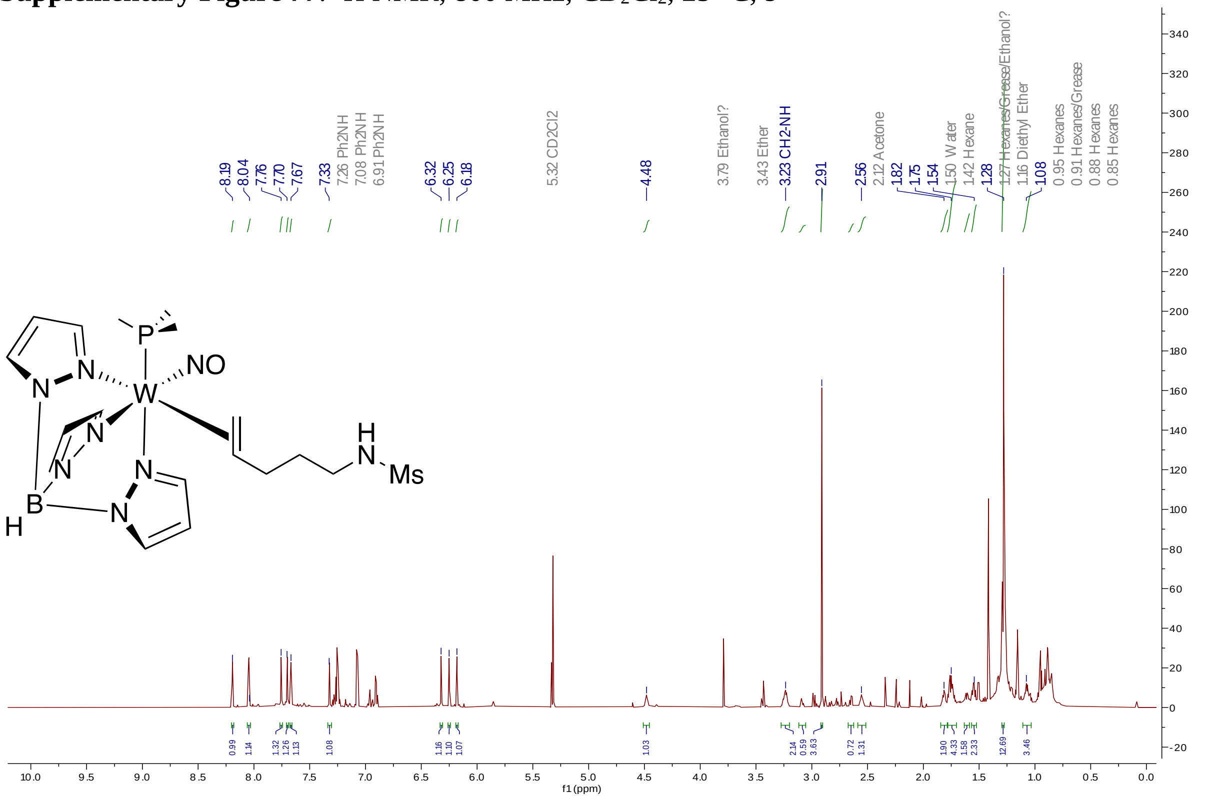

Supplementary Figure 78: ^{13}C NMR, 200 MHz, CD_2Cl_2, 25 °C, **9**

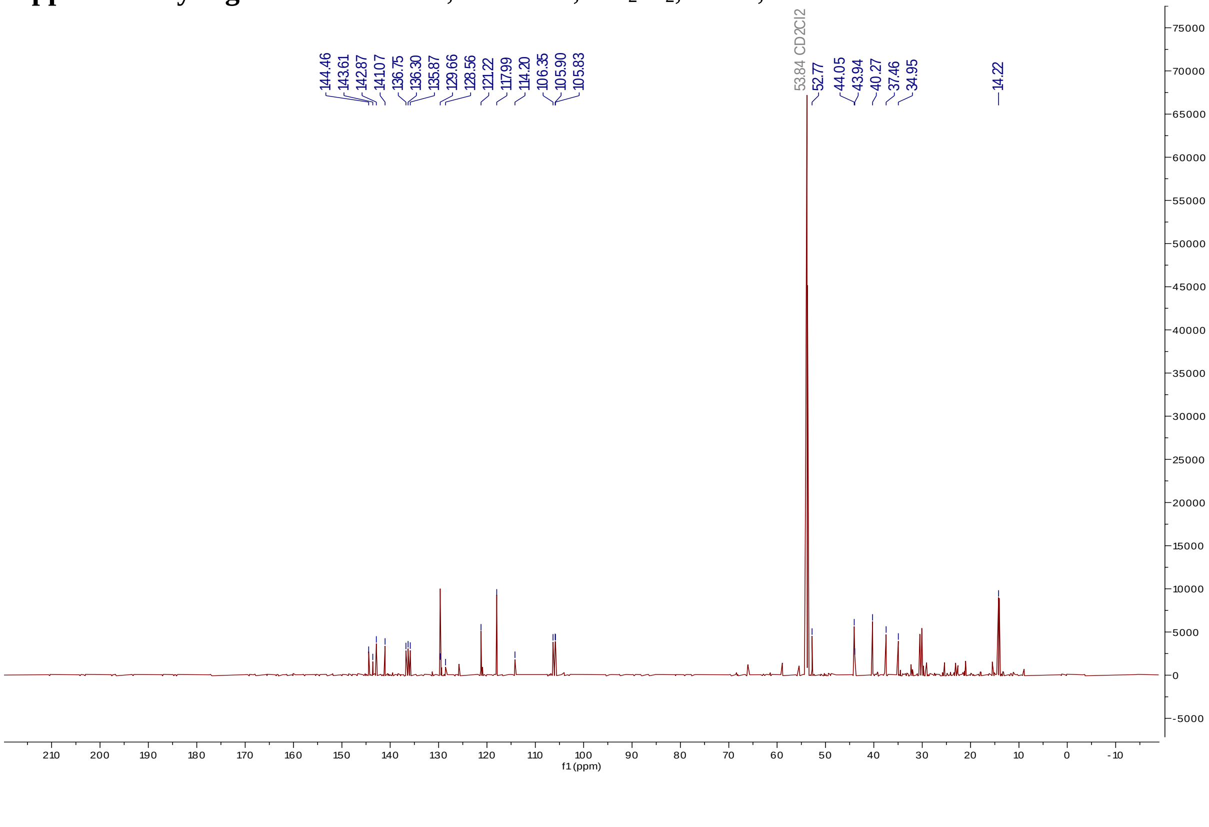

Supplementary Figure 79: NOESY NMR, 800 MHz, CD_2Cl_2, 25 °C, **9**

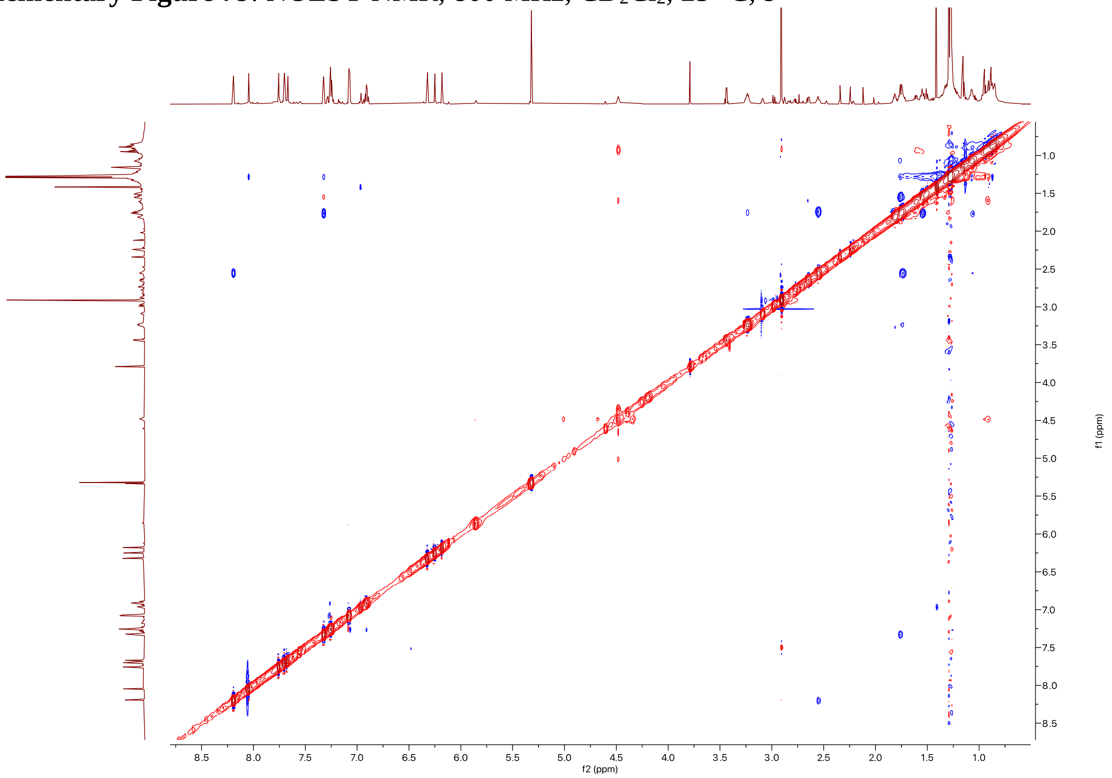

Supplementary Figure 80: COSY NMR, 800 MHz, CD_2Cl_2, 25 °C, **9**

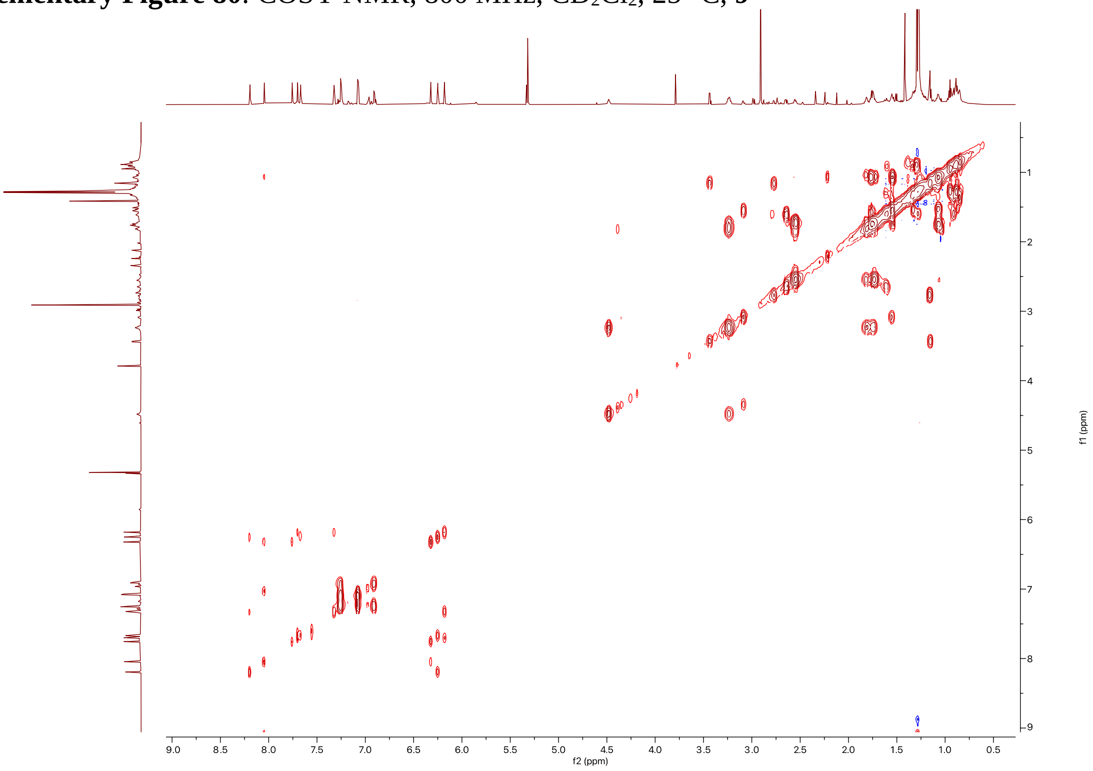

Supplementary Figure 81: HSQC NMR, 800 MHz, CD_2Cl_2, 25 °C, **9**

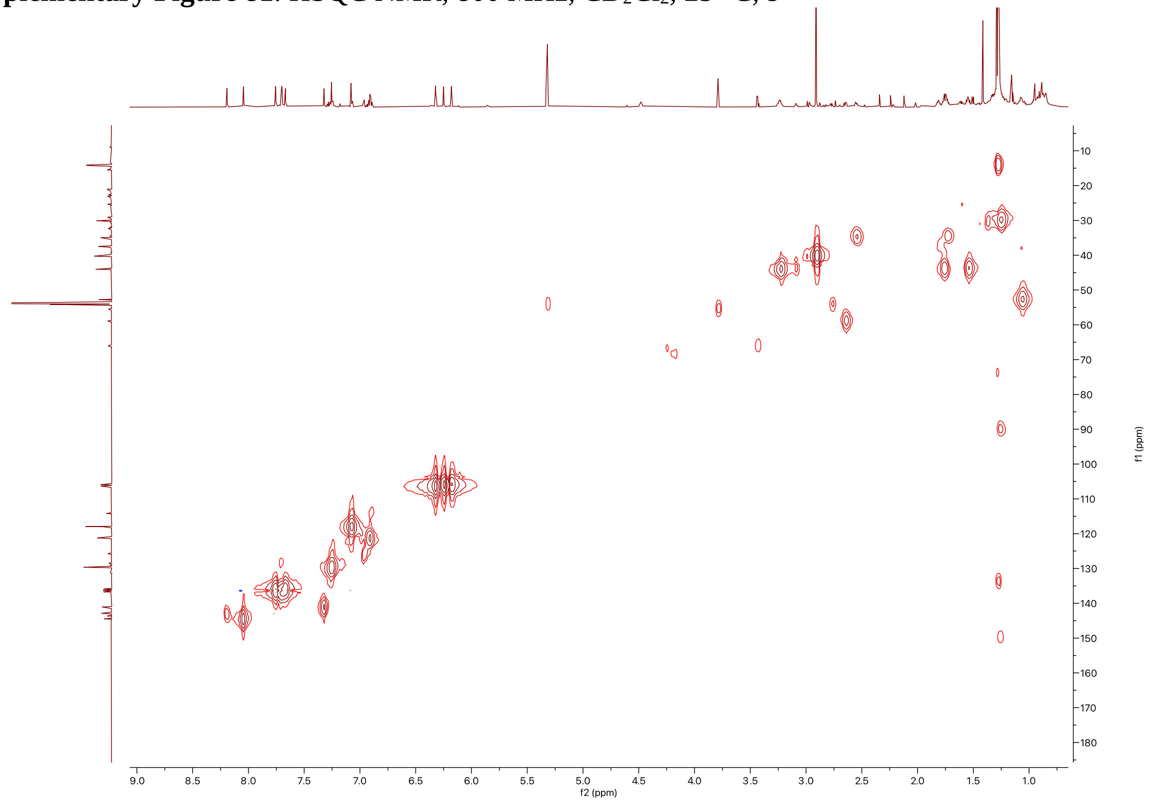

Supplementary Figure 82: HMBC NMR, 800 MHz, CD_2Cl_2, 25 °C, **9**

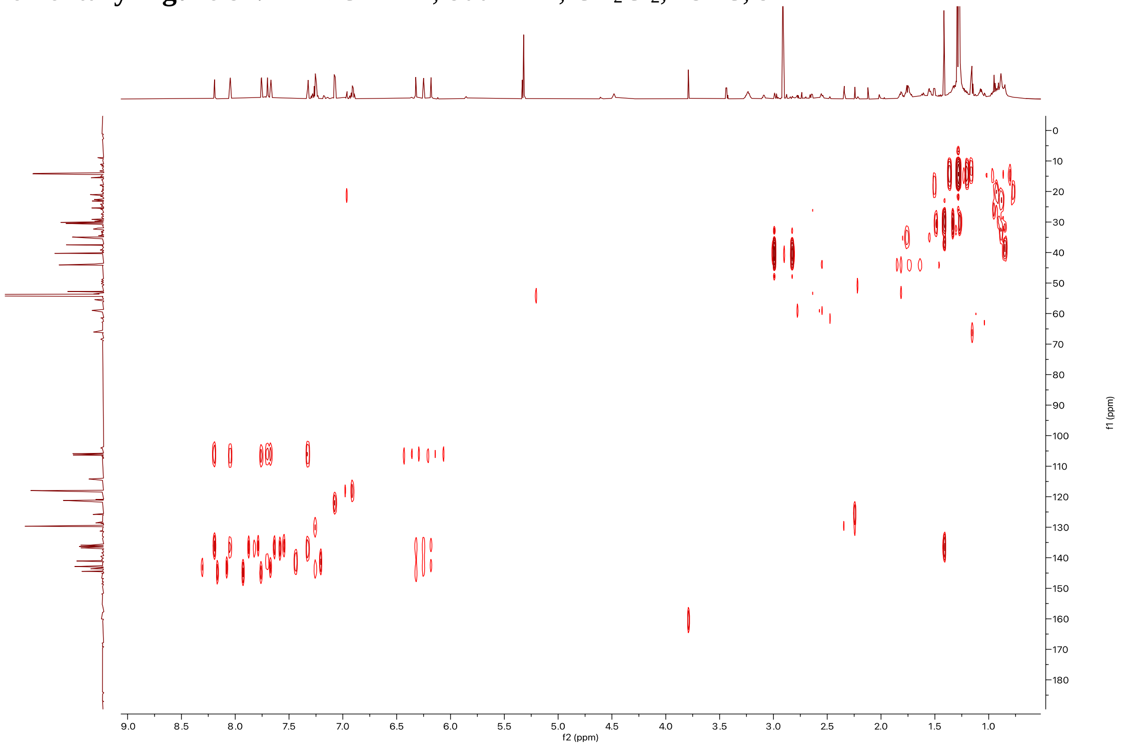

Supplementary Figure 83: ^{1}H NMR, 800 MHz, CD_2Cl_2, 25 °C, ***d*$_5$-11**

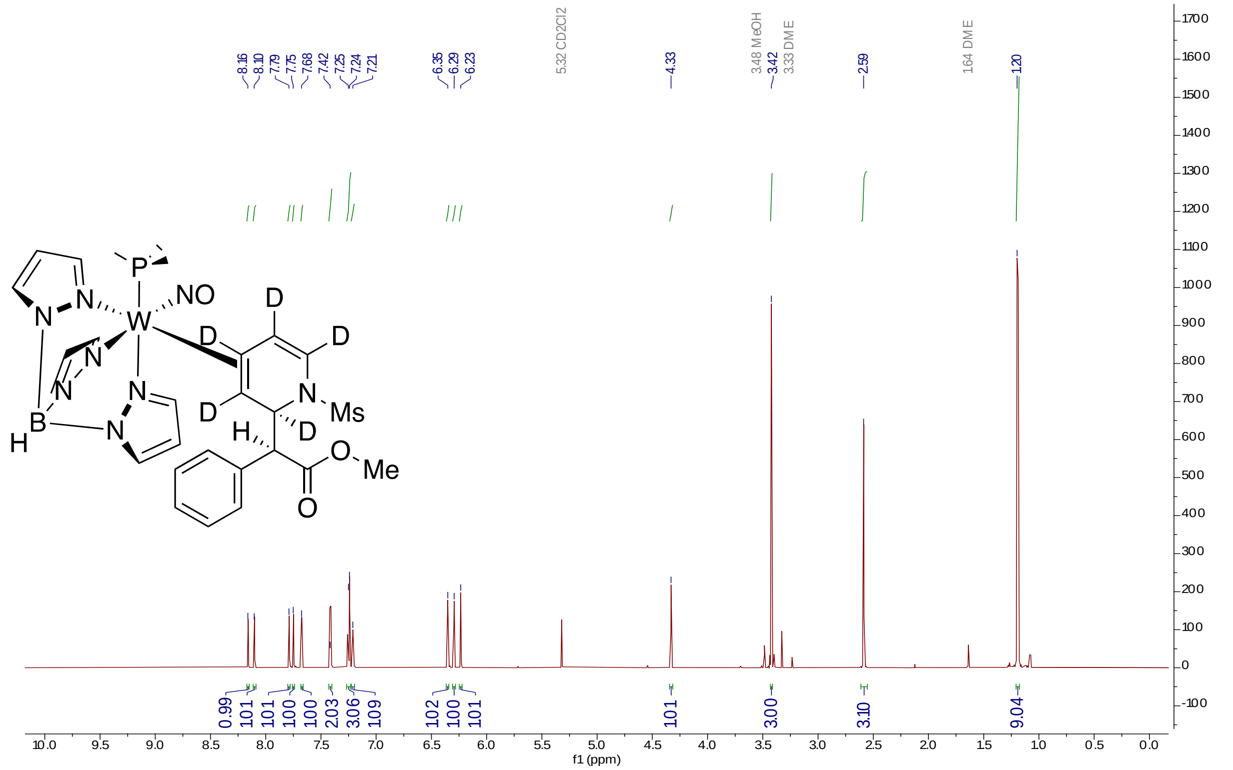

Supplementary Figure 84: ^{13}C NMR, 200 MHz, CD_2Cl_2, 25 °C, ***d*$_5$-11**

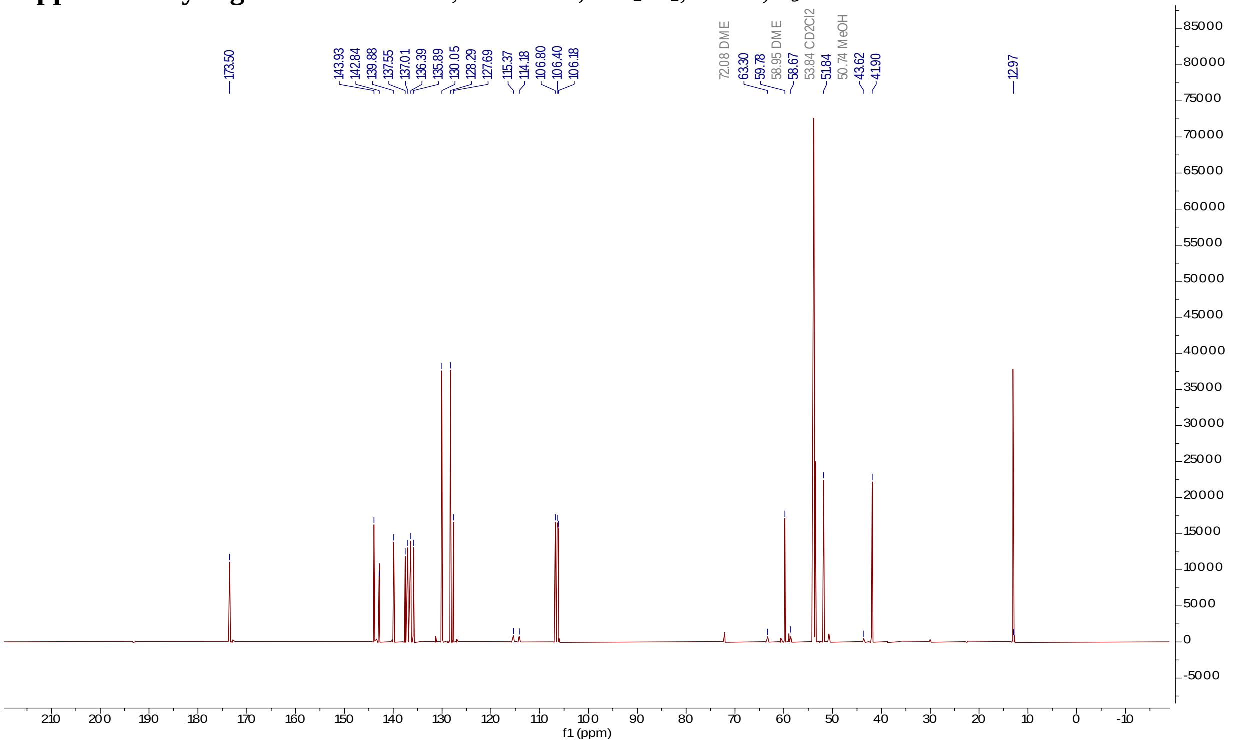

Supplementary Figure 85: ^{1}H NMR, 800 MHz, CD_2Cl_2, 25 °C, ***d*$_1$-12**

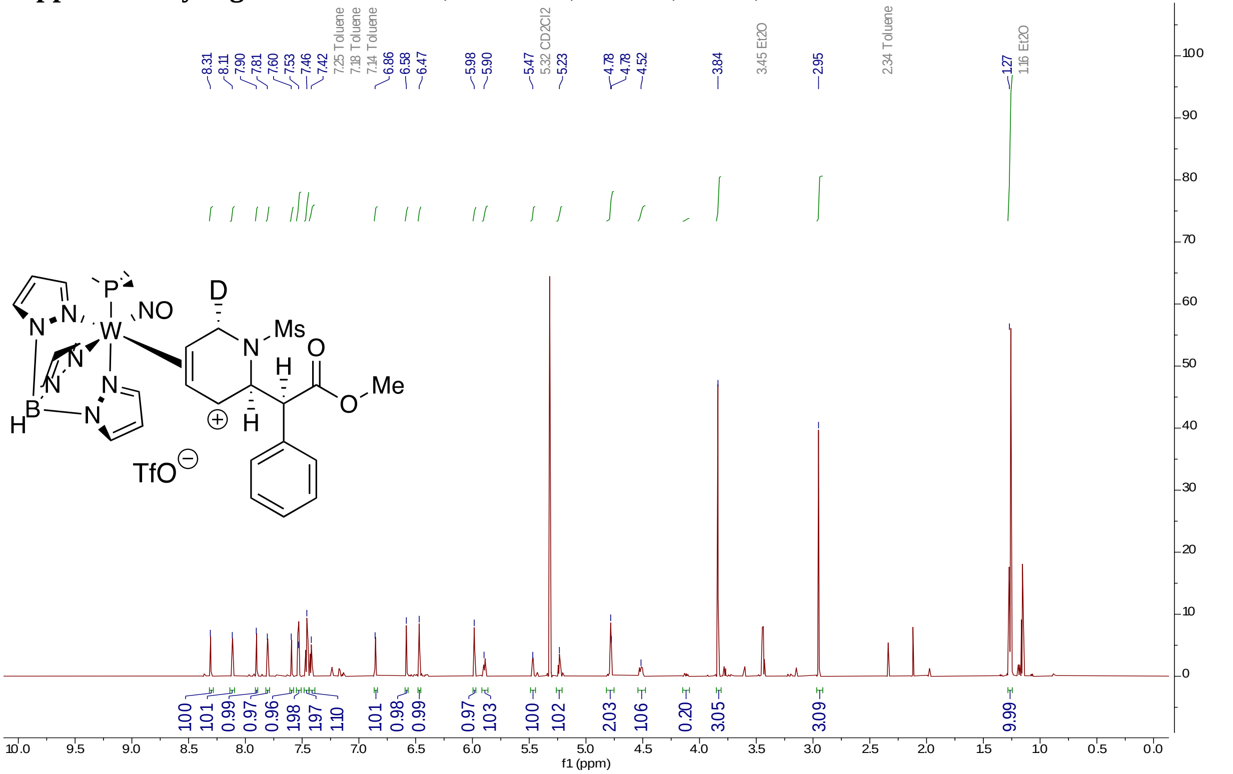

Supplementary Figure 86: ^{13}C NMR, 200 MHz, CD_2Cl_2, 25 °C, ***d*$_1$-12**

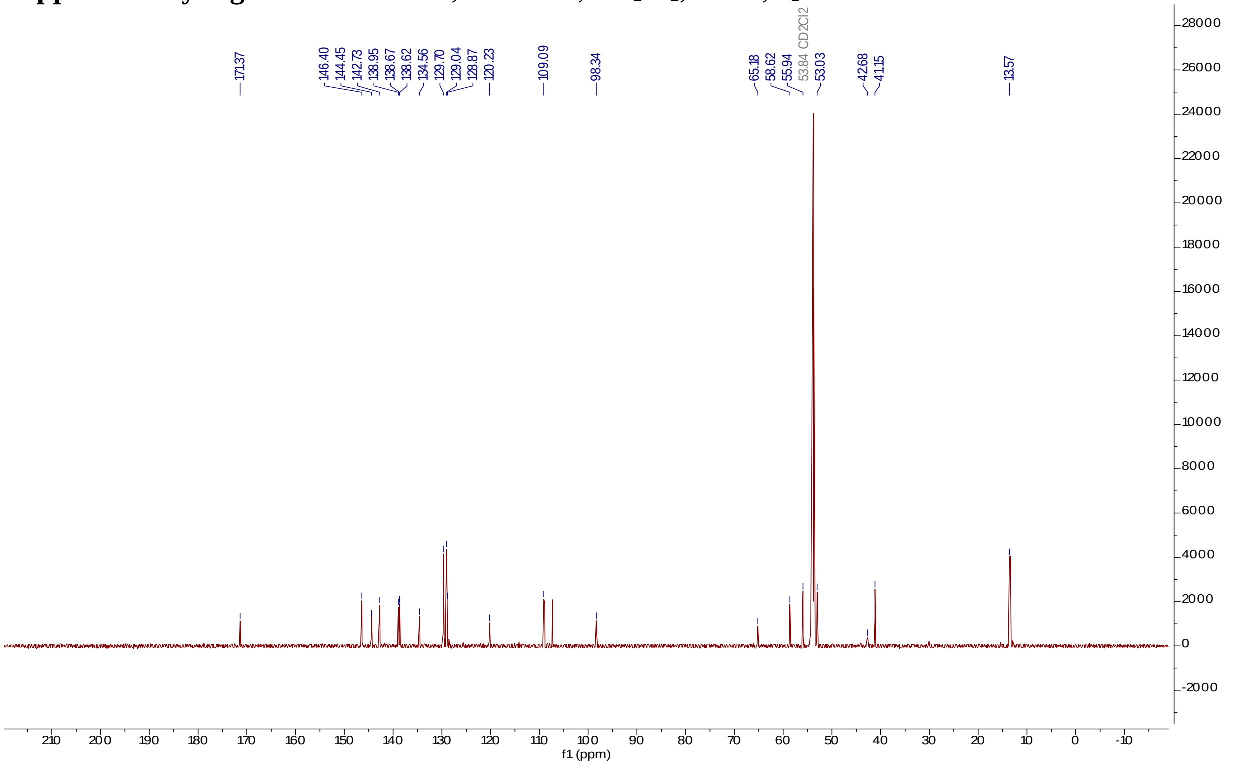

Supplementary Figure 87: ^{1}H NMR, 800 MHz, CD_2Cl_2, 25 °C, ***d*₅-12**

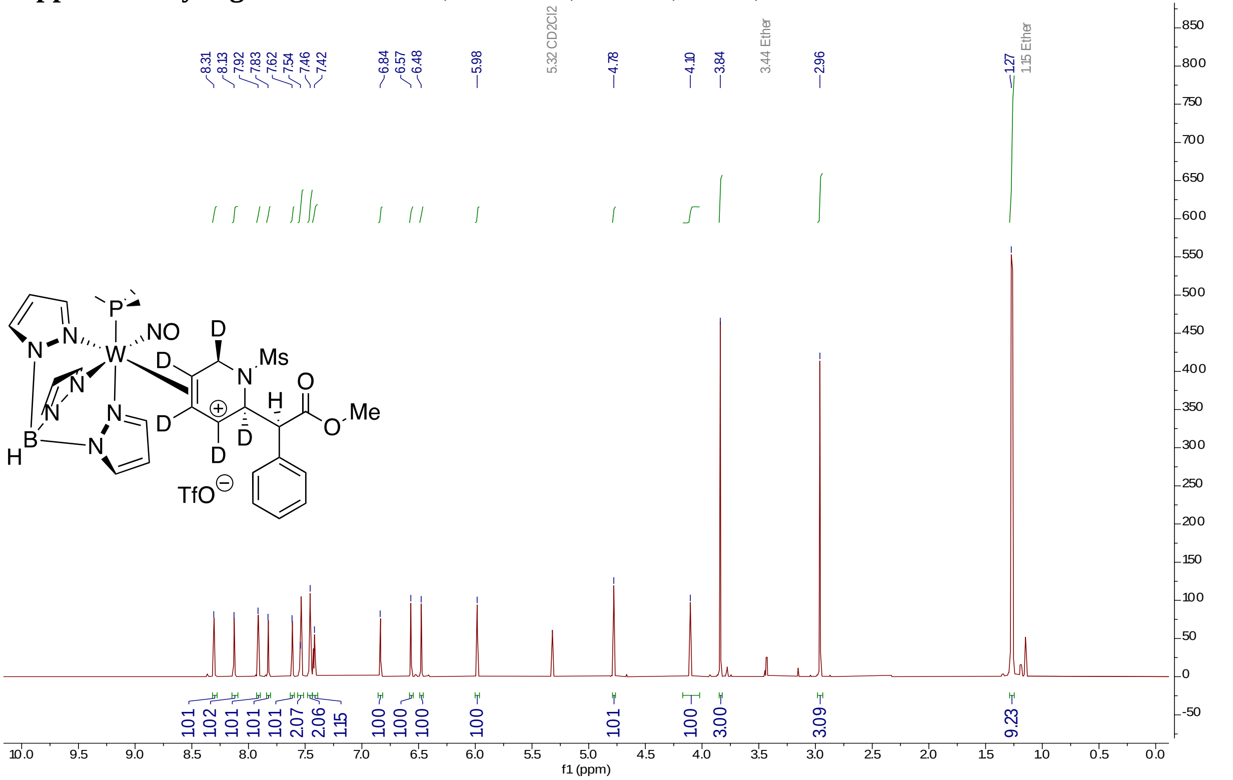

Supplementary Figure 88: ^{13}C NMR, 200 MHz, CD_2Cl_2, 25 °C, ***d*₅-12**

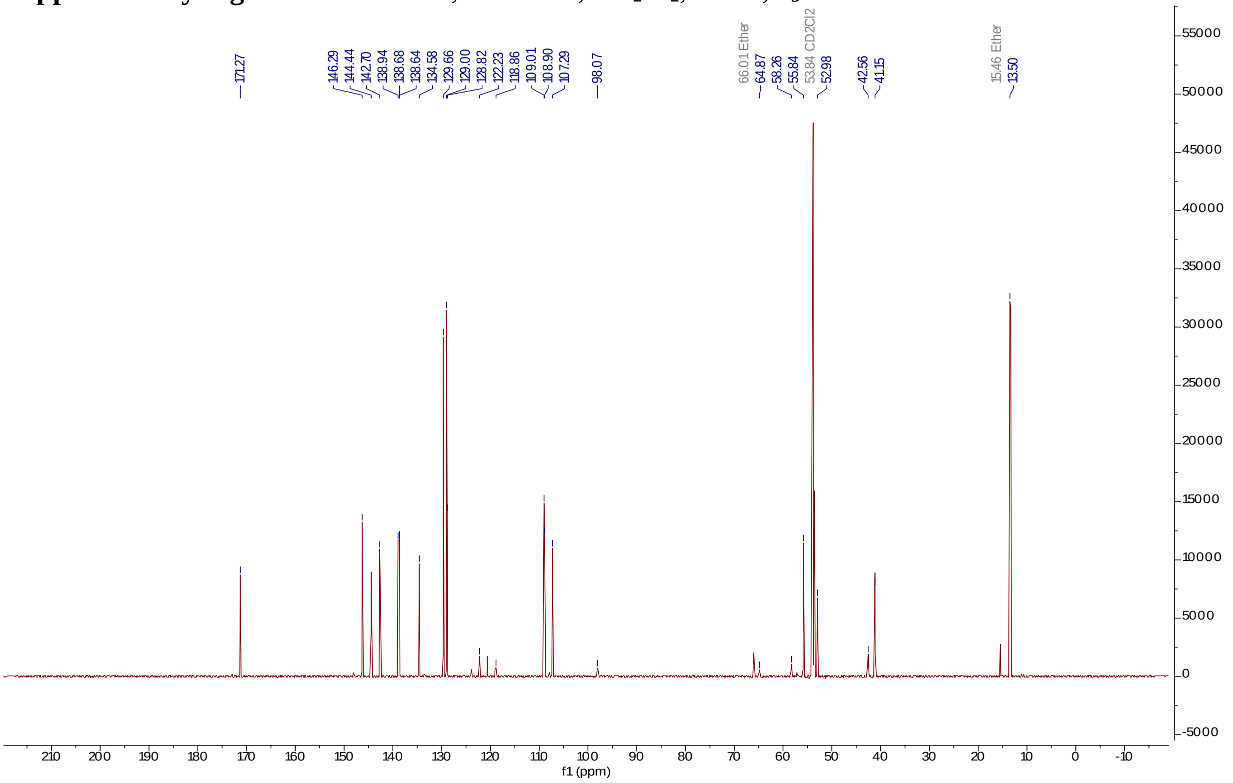

Supplementary Figure 89: ^{1}H NMR, 800 MHz, CD_2Cl_2, 25 °C, ***d*$_6$-12**

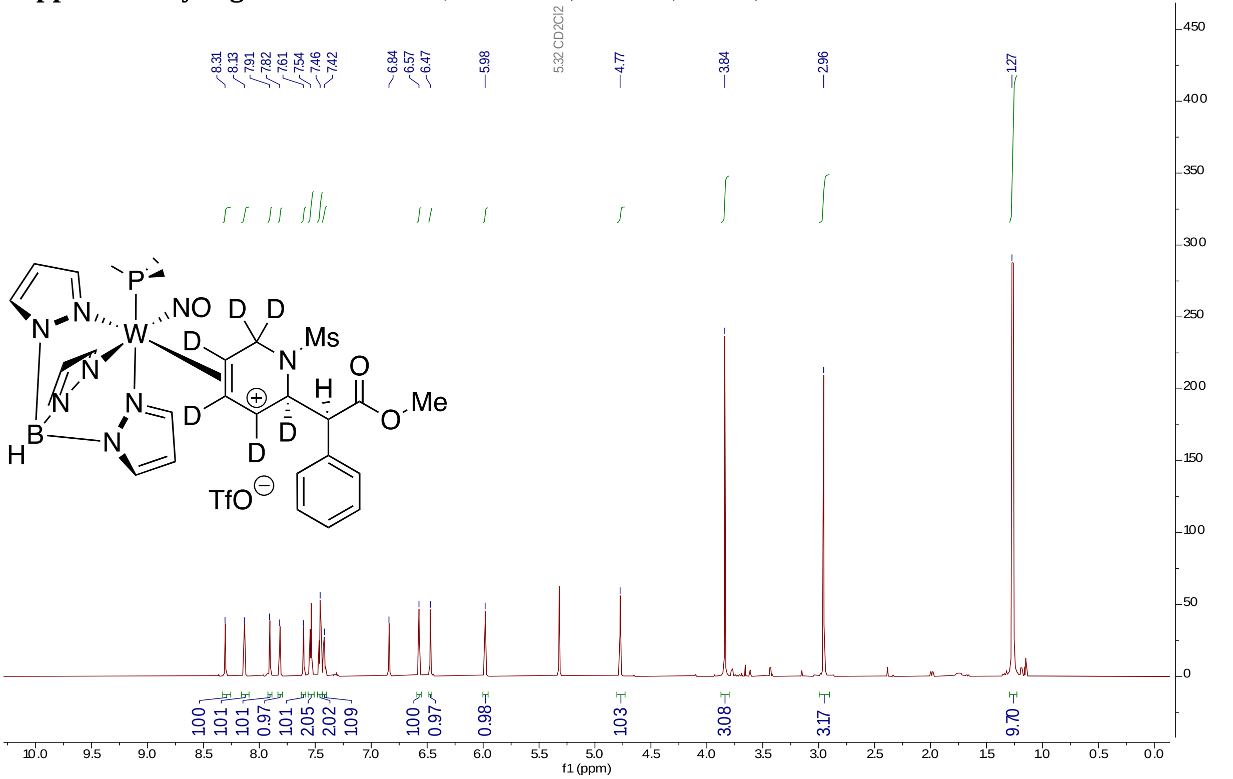

Supplementary Figure 90: ^{13}C NMR, 200 MHz, CD_2Cl_2, 25 °C, ***d*$_6$-12**

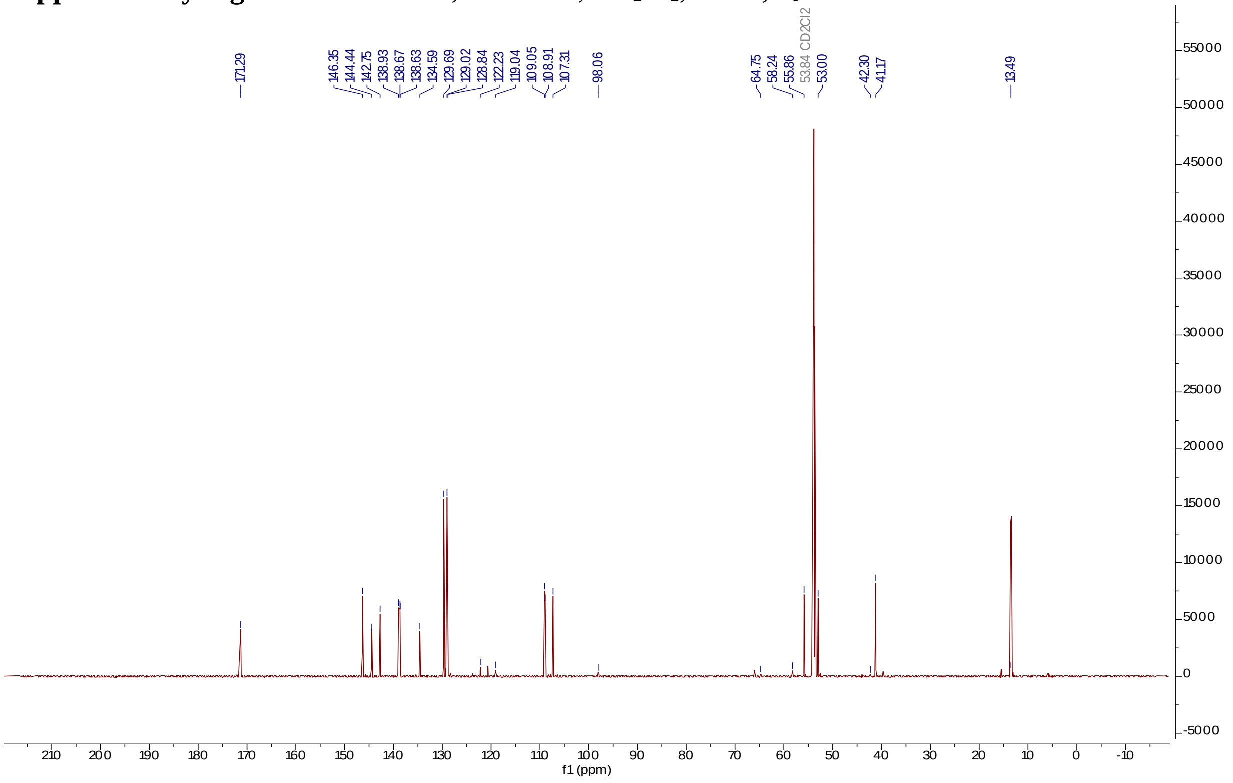

Supplementary Figure 91: ^{1}H NMR, 800 MHz, CD_2Cl_2, 25 °C, ***Rel-(5R)-d₁-13***

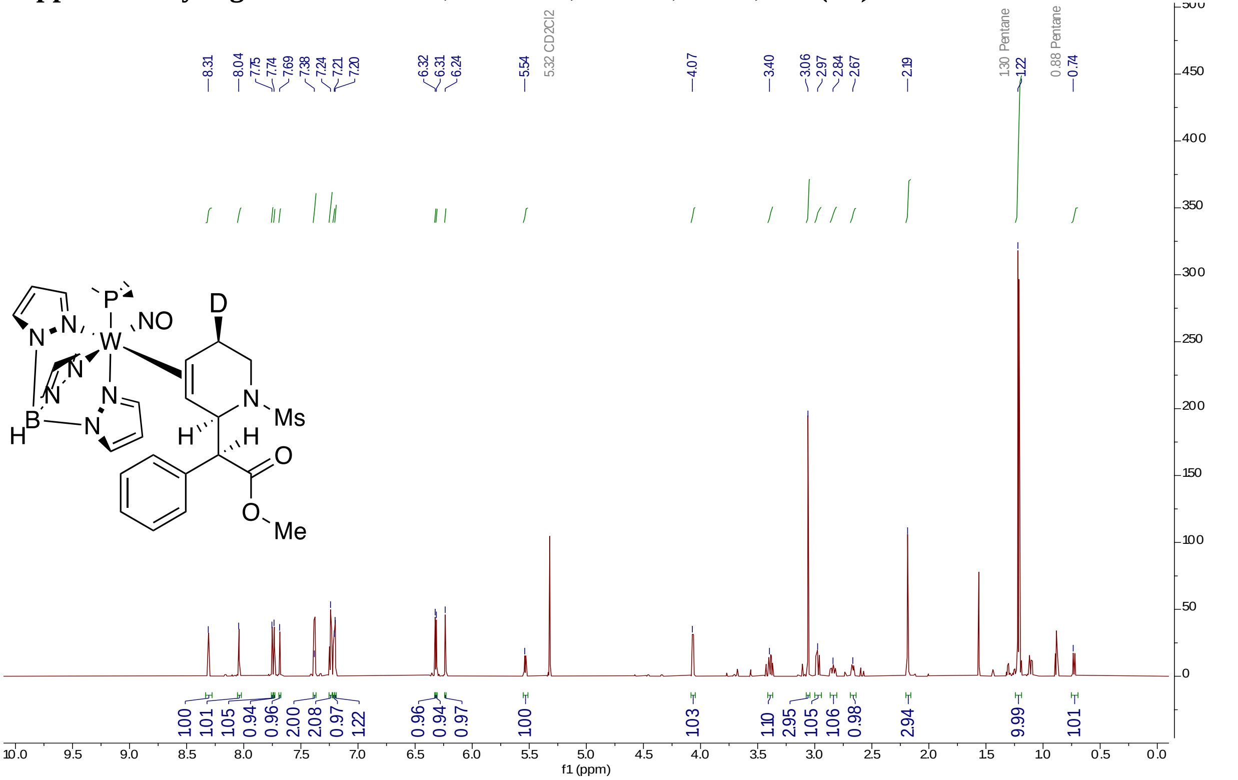

Supplementary Figure 92: ^{13}C NMR, 200 MHz, CD_2Cl_2, 25 °C, ***Rel-(5R)-d₁-13***

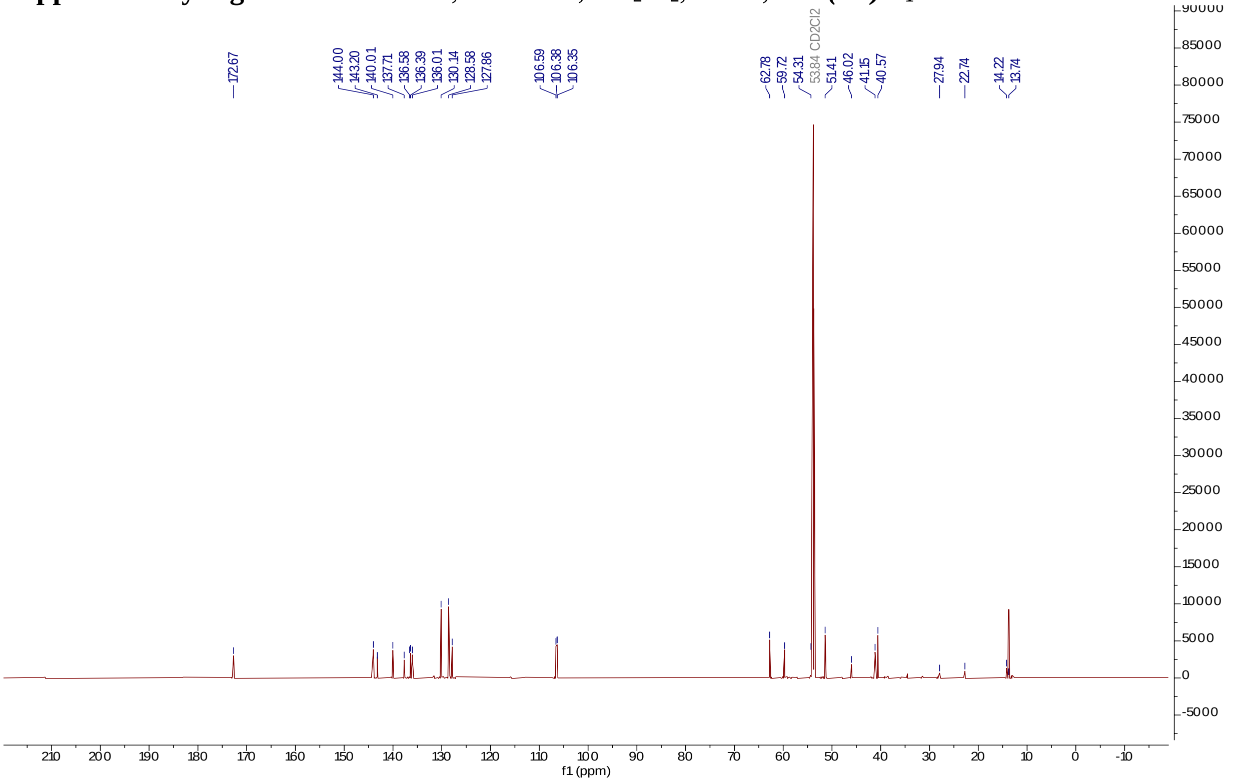

Supplementary Figure 93: ^{1}H NMR, 800 MHz, CD_2Cl_2, 25 °C, ***Rel-(6S)-d$_1$*-13**

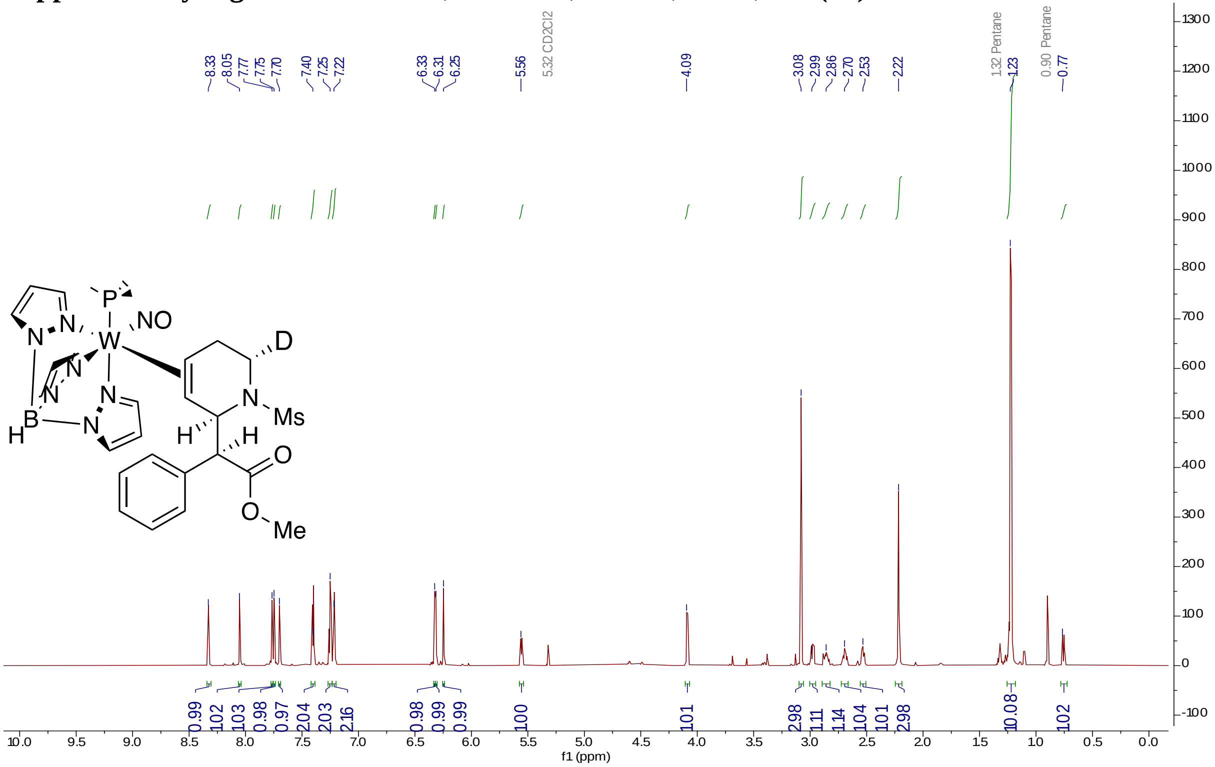

Supplementary Figure 94: ^{13}C NMR, 200 MHz, CD_2Cl_2, 25 °C, ***Rel-(6S)-d$_1$*-13**

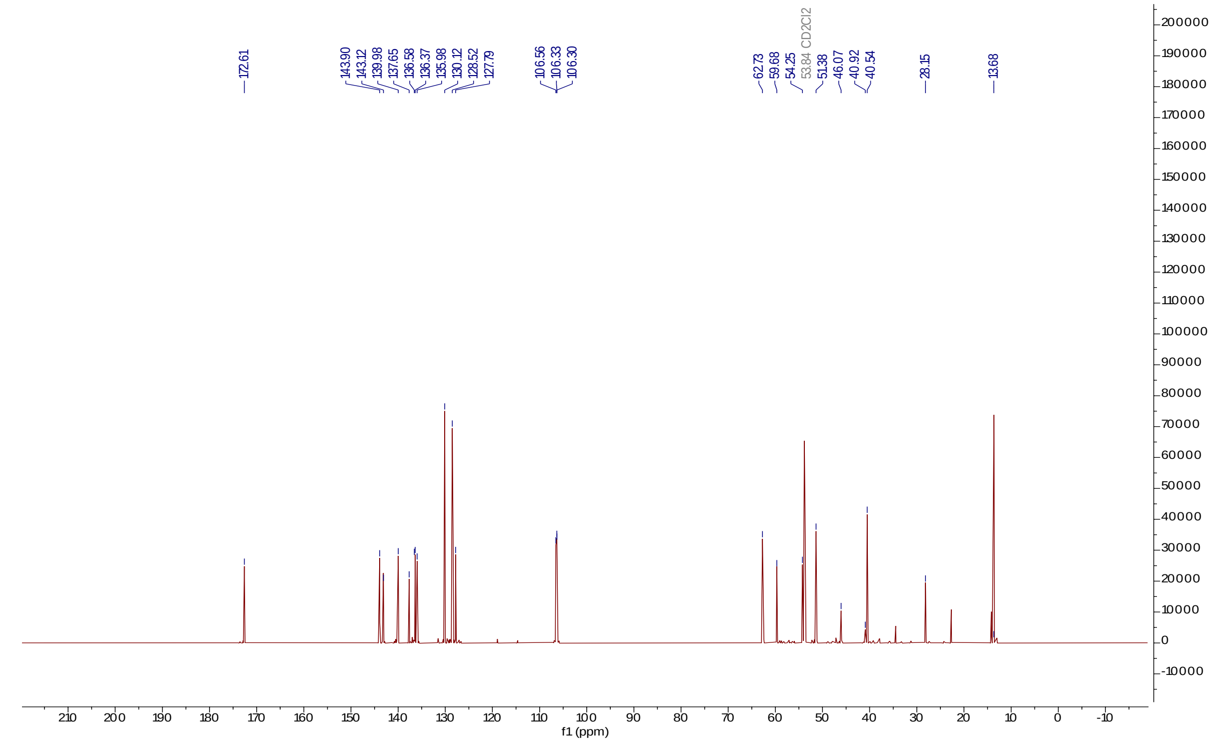

Supplementary Figure 95: ^{1}H NMR, 800 MHz, CD_2Cl_2, 25 °C, ***Rel-(5R,6S)-d$_2$-13***

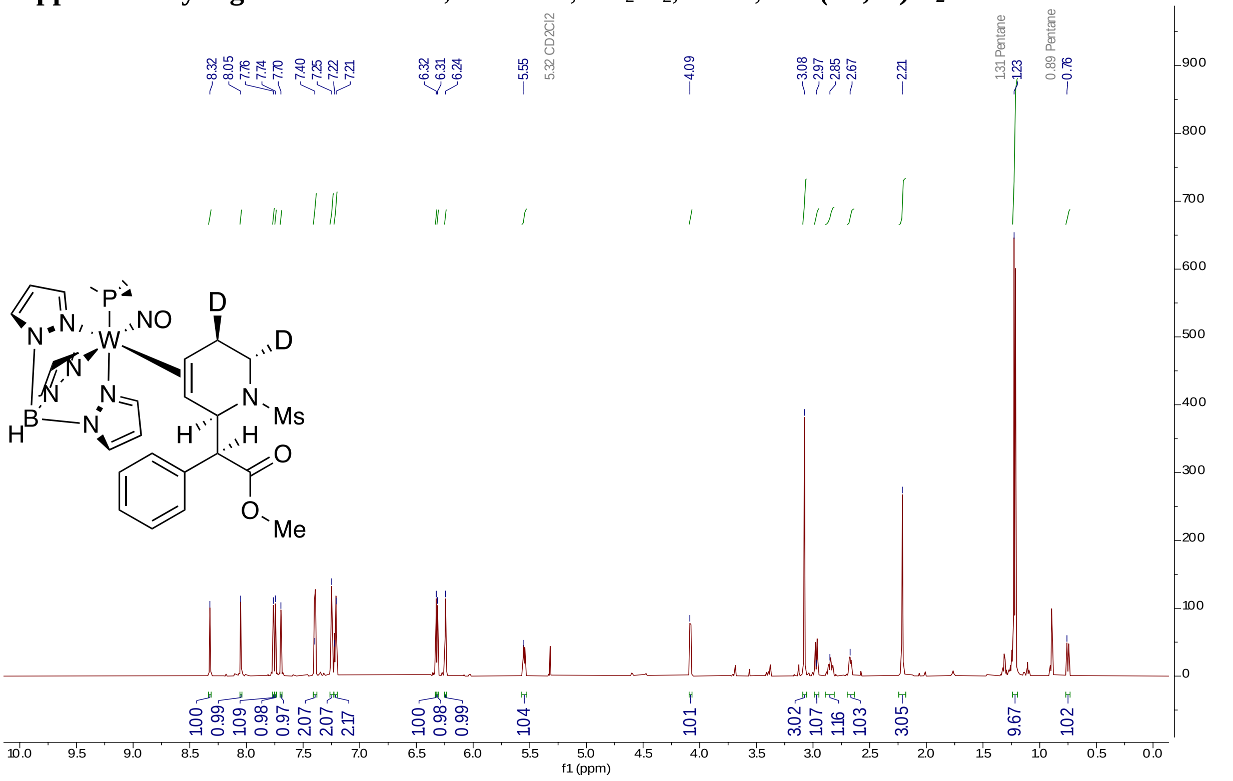

Supplementary Figure 96: ^{13}C NMR, 200 MHz, CD_2Cl_2, 25 °C, ***Rel-(5R,6S)-d$_2$-13***

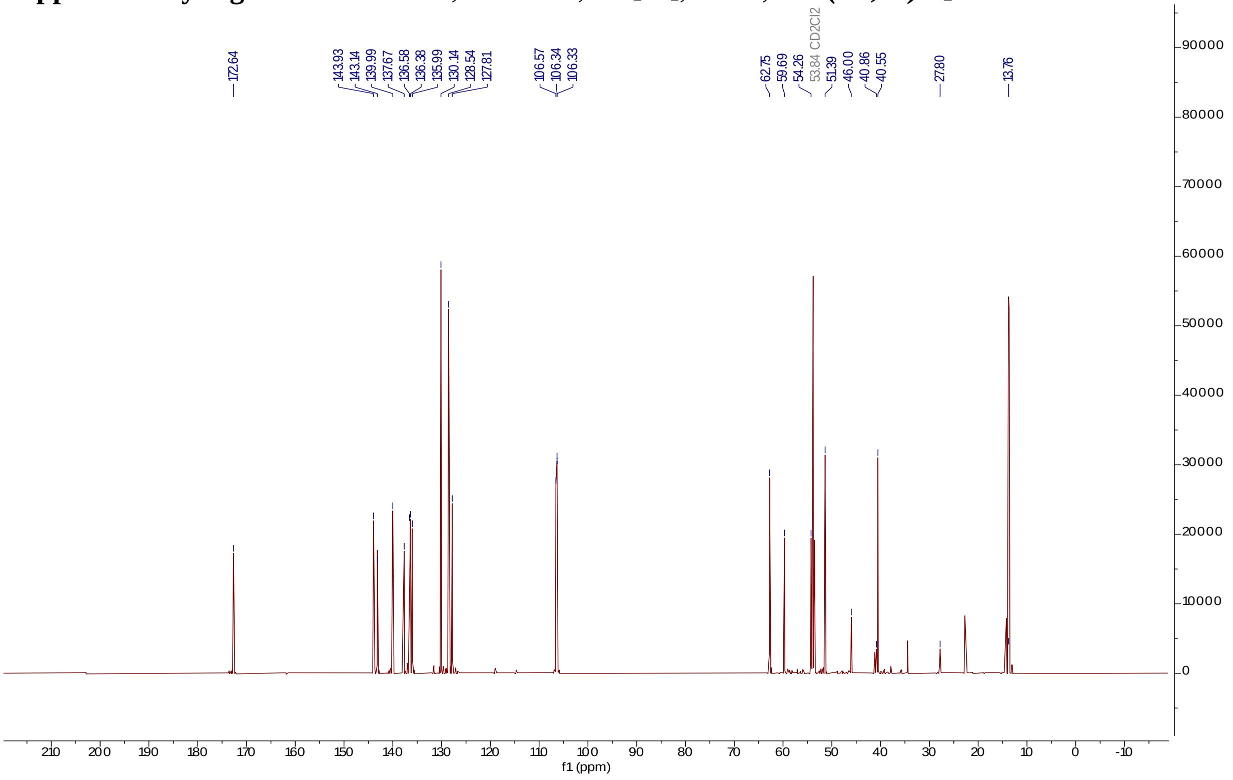

Supplementary Figure 97: ^{1}H NMR, 800 MHz, CD_2Cl_2, 25 °C, ***Rel-(5S,6R)-d$_5$-13***

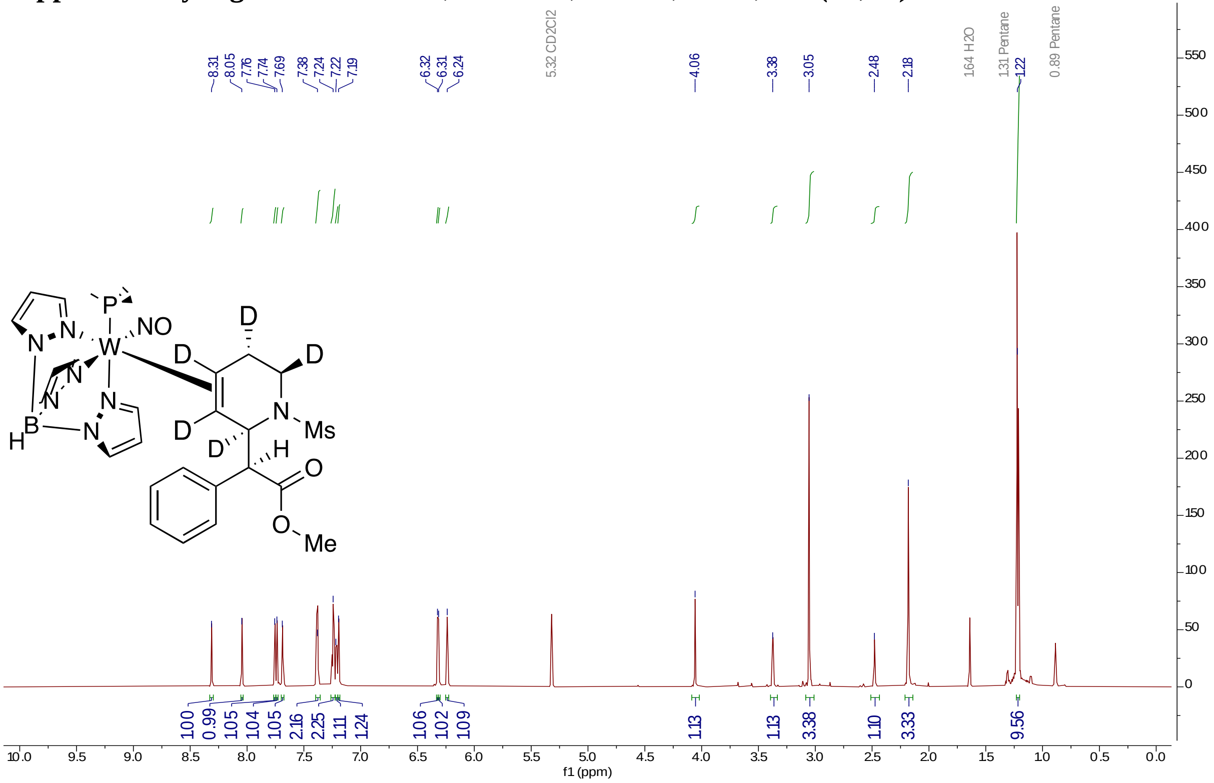

Supplementary Figure 98: ^{13}C NMR, 200 MHz, CD_2Cl_2, 25 °C, ***Rel-(5S,6R)-d$_5$-13***

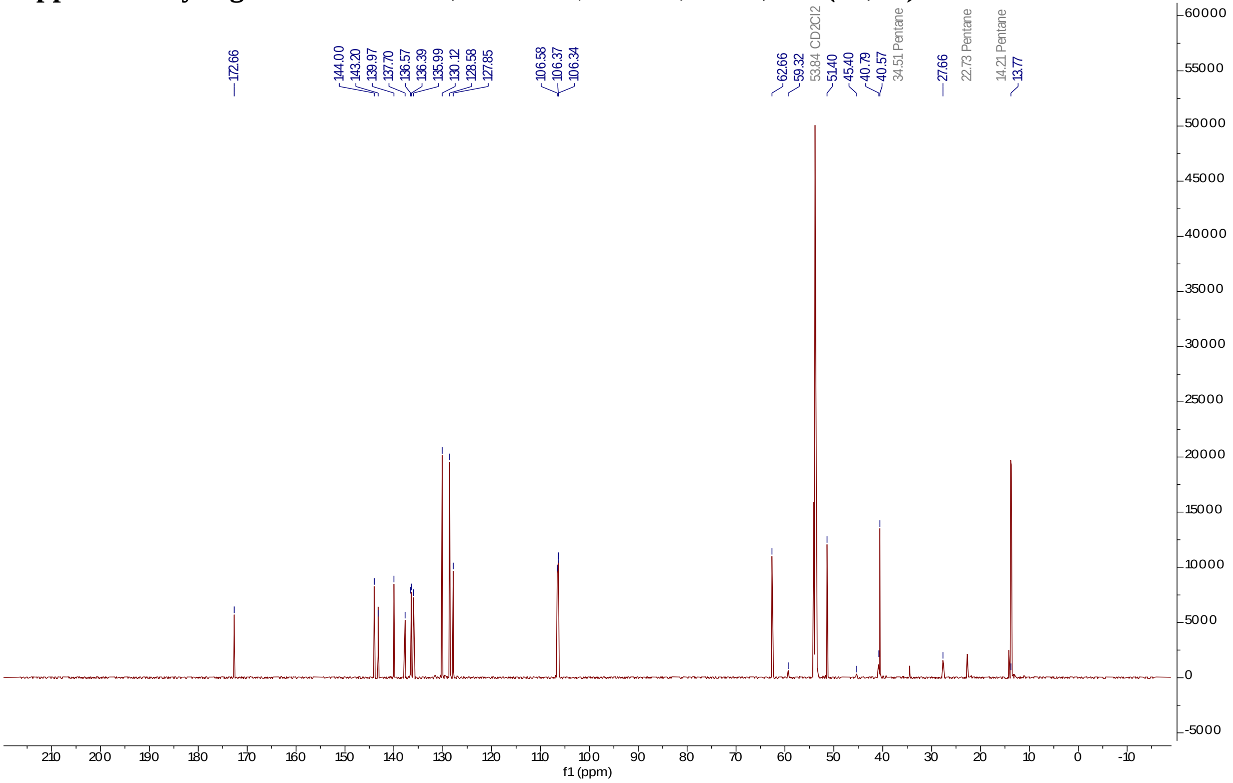

Supplementary Figure 99: ^{1}H NMR, 800 MHz, CD_2Cl_2, 25 °C, ***Rel-(6R)-d₆-13***

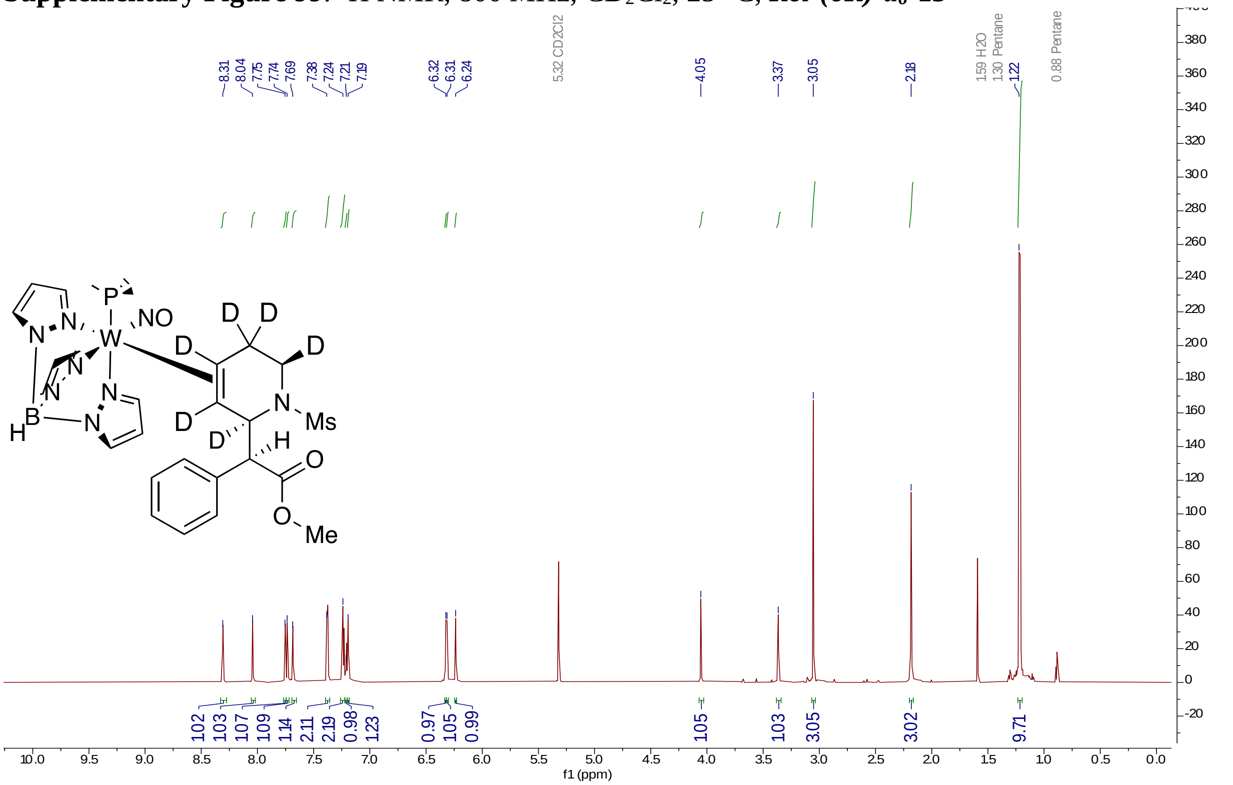

Supplementary Figure 100: ^{13}C NMR, 200 MHz, CD_2Cl_2, 25 °C, ***Rel-(6R)-d₆-13***

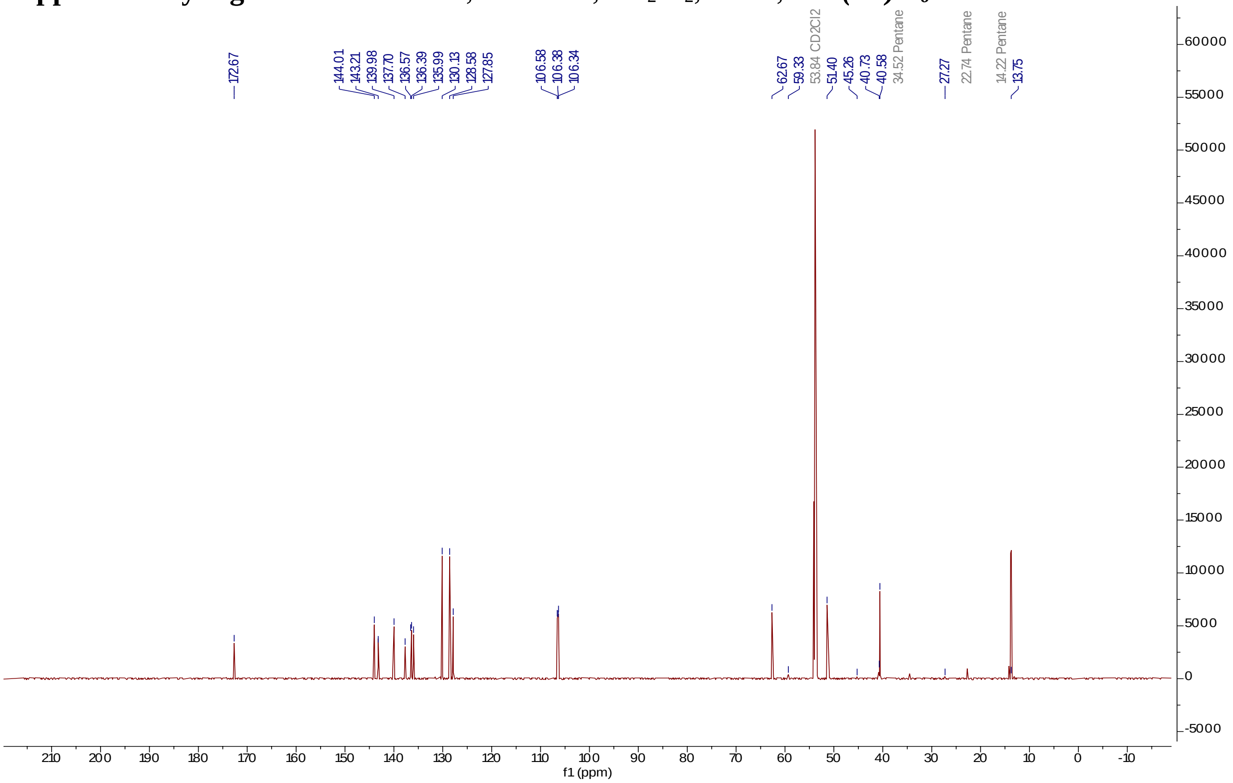

Supplementary Figure 101: ^{1}H NMR, 800 MHz, CD_2Cl_2, 25 °C, ***Rel-(5S)-d*$_6$*-13***

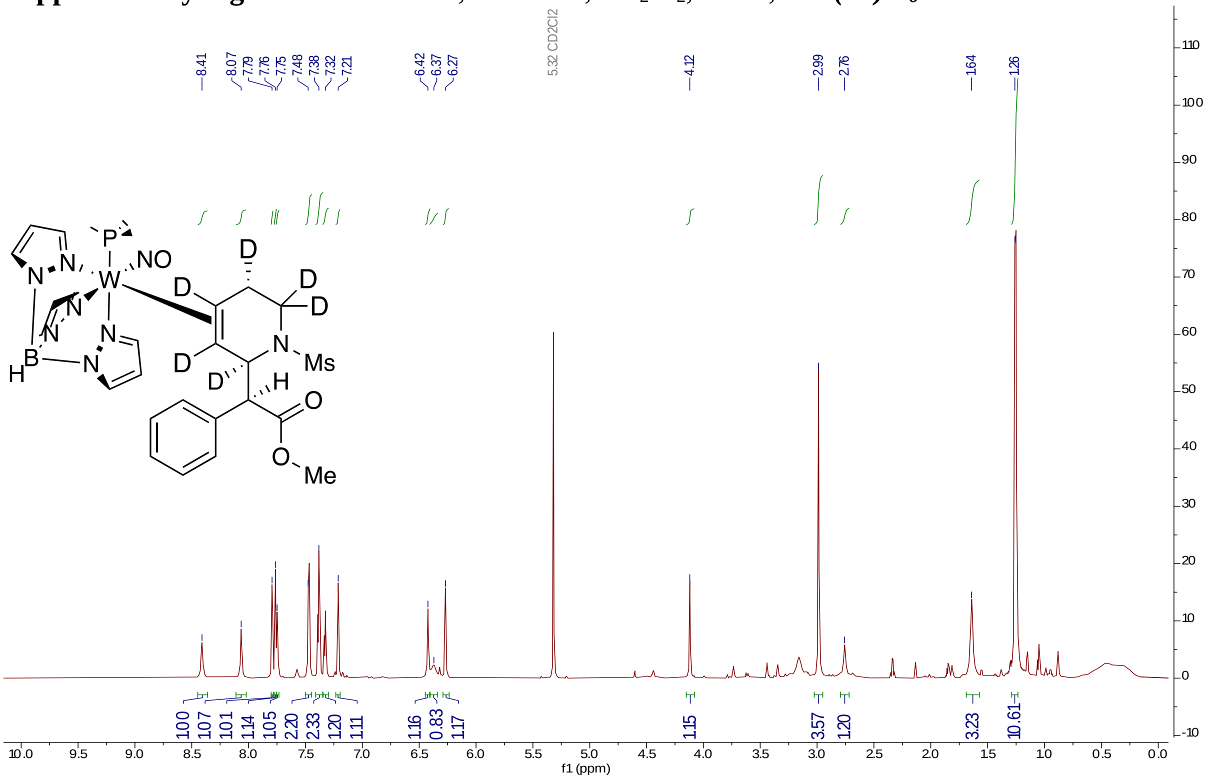

Supplementary Figure 102: ^{13}C NMR, 200 MHz, CD_2Cl_2, 25 °C, ***Rel-(5S)-d*$_6$*-13***

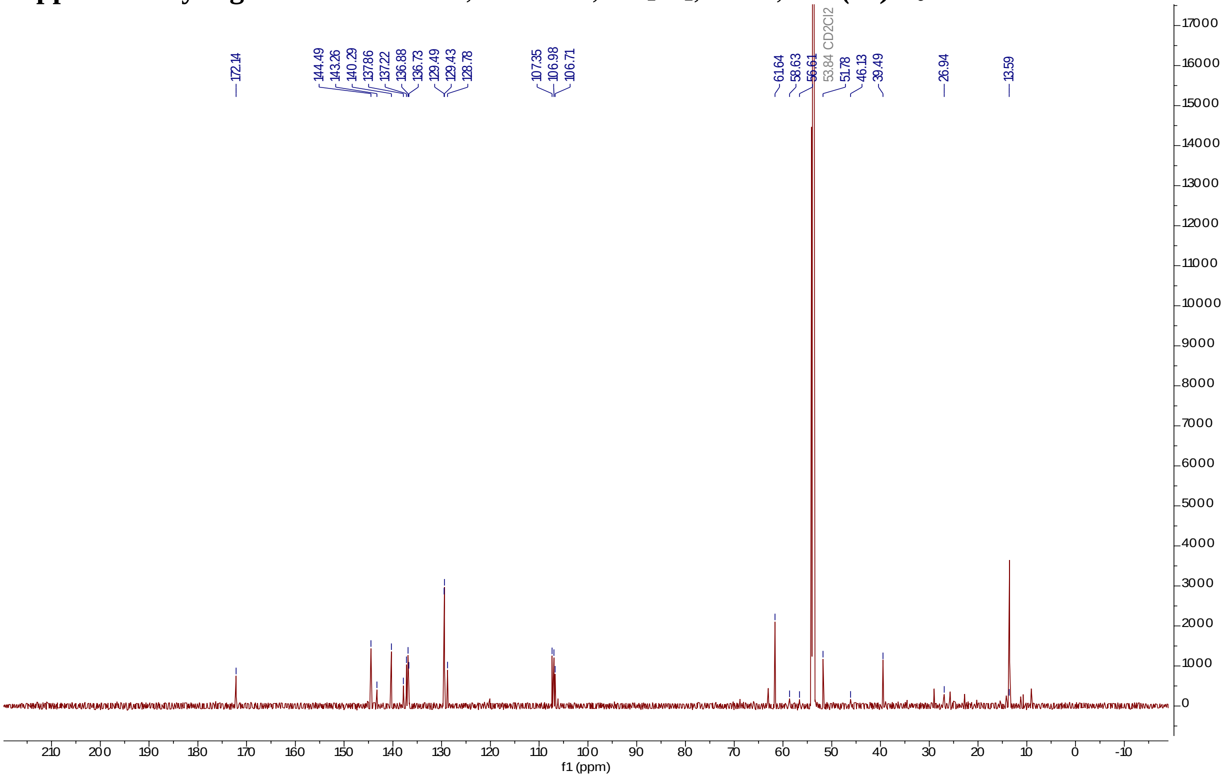

Supplementary Figure 103: ^{1}H NMR, 800 MHz, CD_2Cl_2, 25 °C, ***d*$_7$-13**

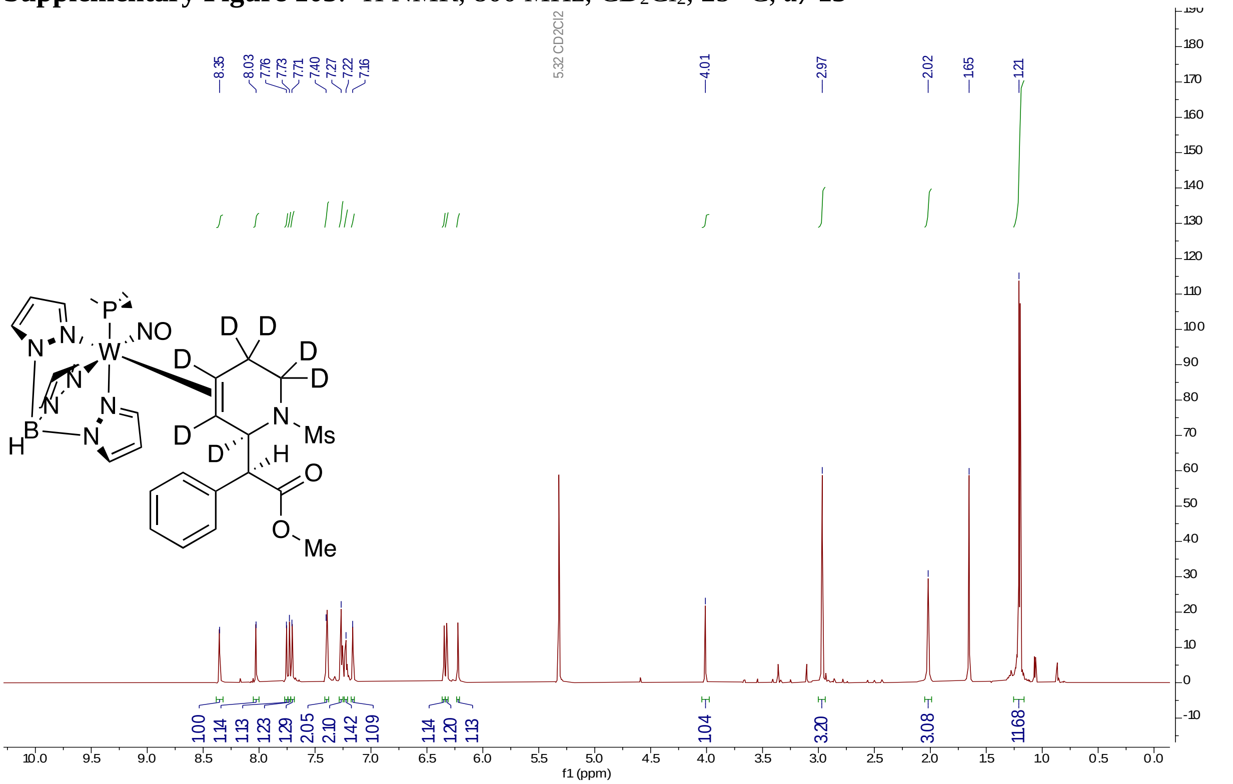

Supplementary Figure 104: ^{13}C NMR, 200 MHz, CD_2Cl_2, 25 °C, ***d*$_7$-13**

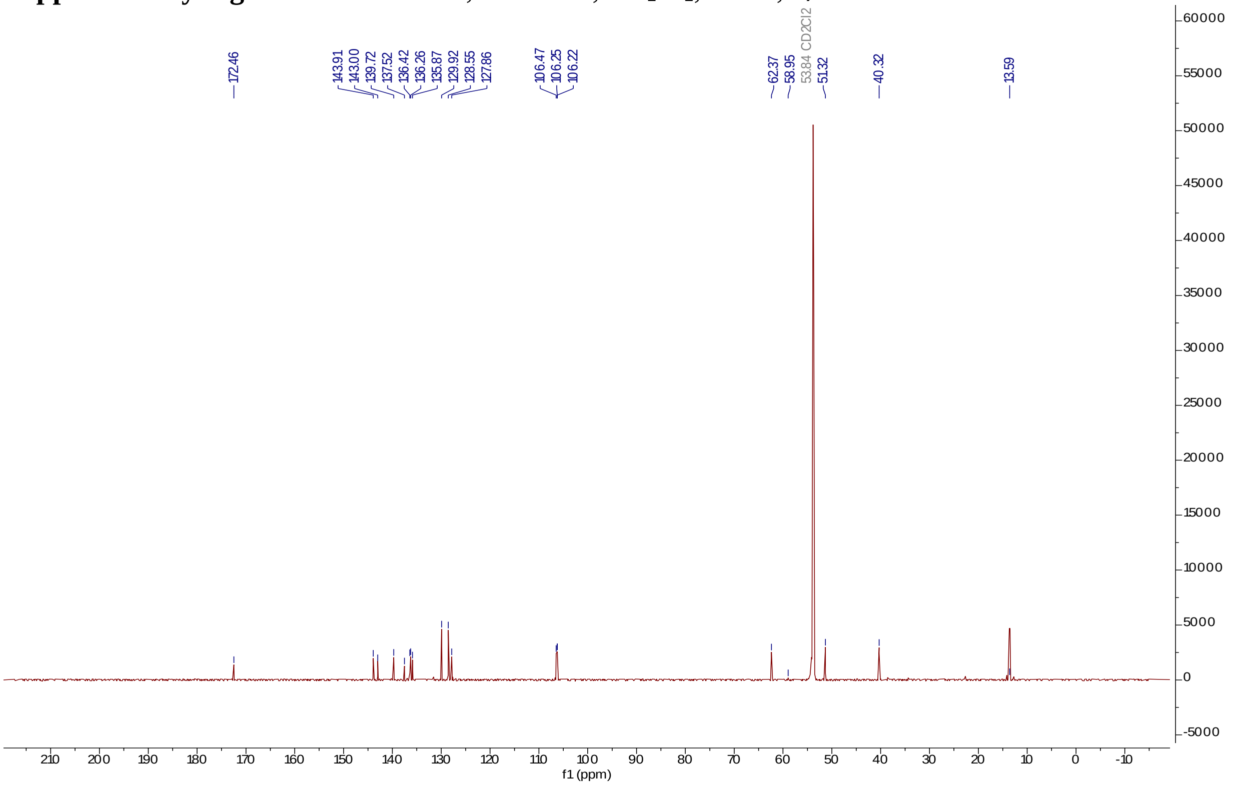

Supplementary Figure 105: ^{1}H NMR, 800 MHz, d_6-acetone, 25 °C, ***Rel-(5R)-d_1-15***

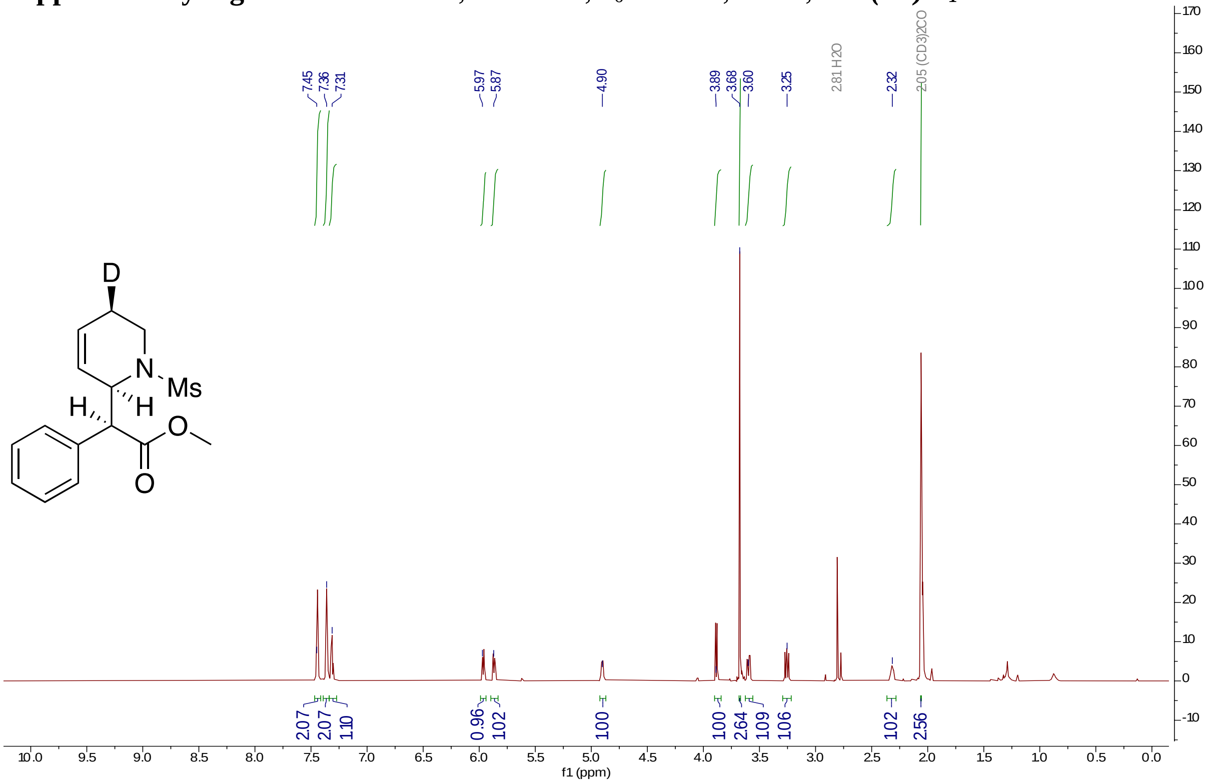

Supplementary Figure 106: ^{13}C NMR, 200 MHz, d_6-acetone, 25 °C, ***Rel-(5R)-d_1-15***

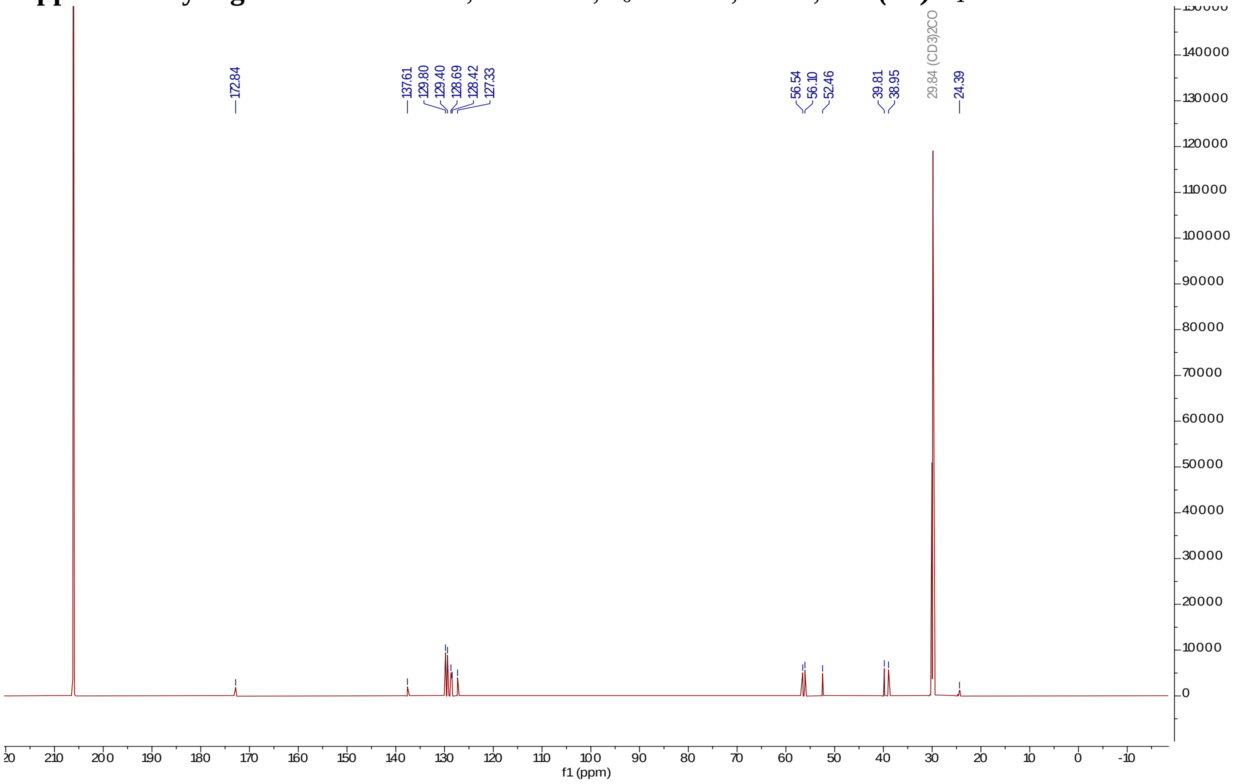

Supplementary Figure 107: ^{1}H NMR, 800 MHz, d_6-acetone, 25 °C, ***Rel-(6S)-d_1*-15**

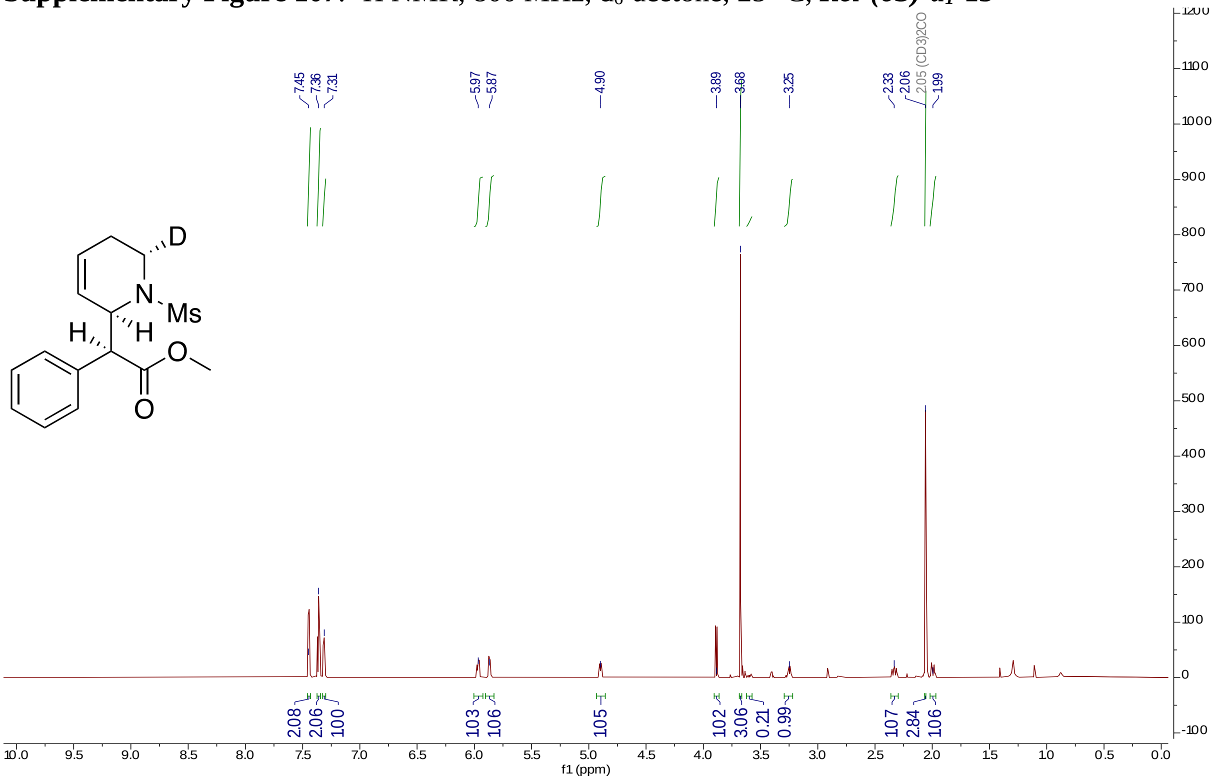

Supplementary Figure 108: ^{13}C NMR, 200 MHz, d_6-acetone, 25 °C, ***Rel-(6S)-d_1*-15**

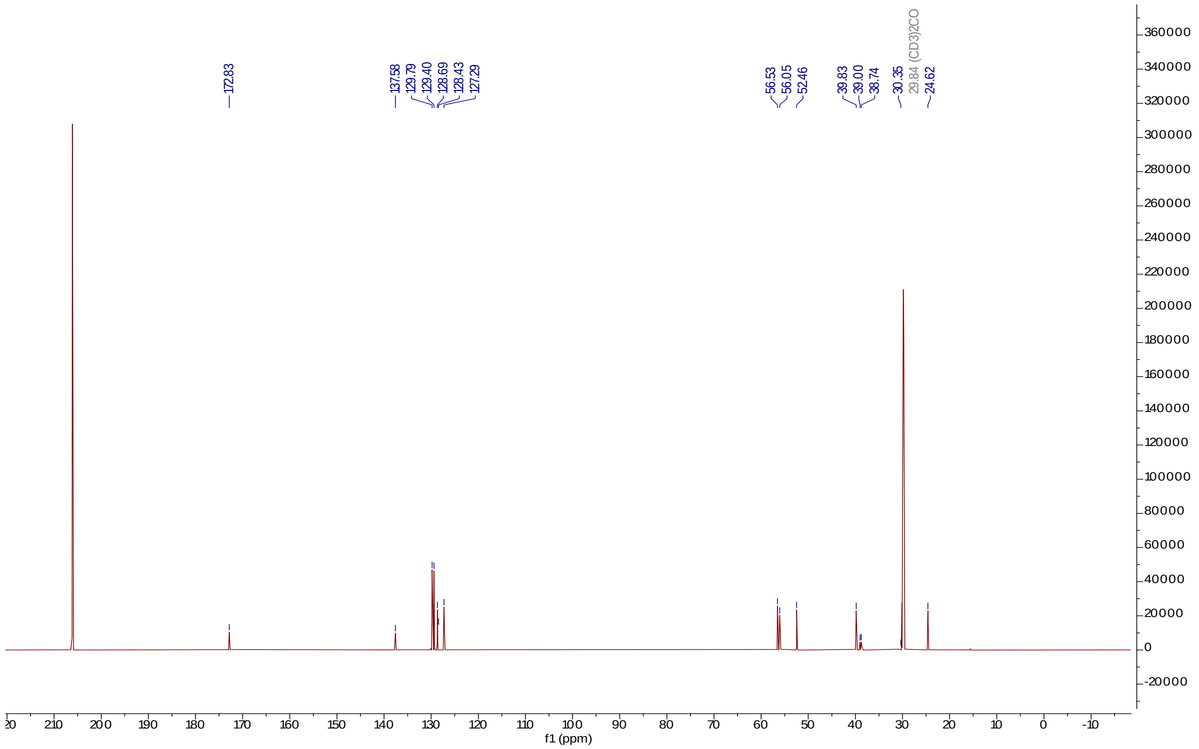

Supplementary Figure 109: ^{1}H NMR, 800 MHz, d$_6$-acetone, 25 °C, ***Rel-(5R,6S)-d$_2$*-15**

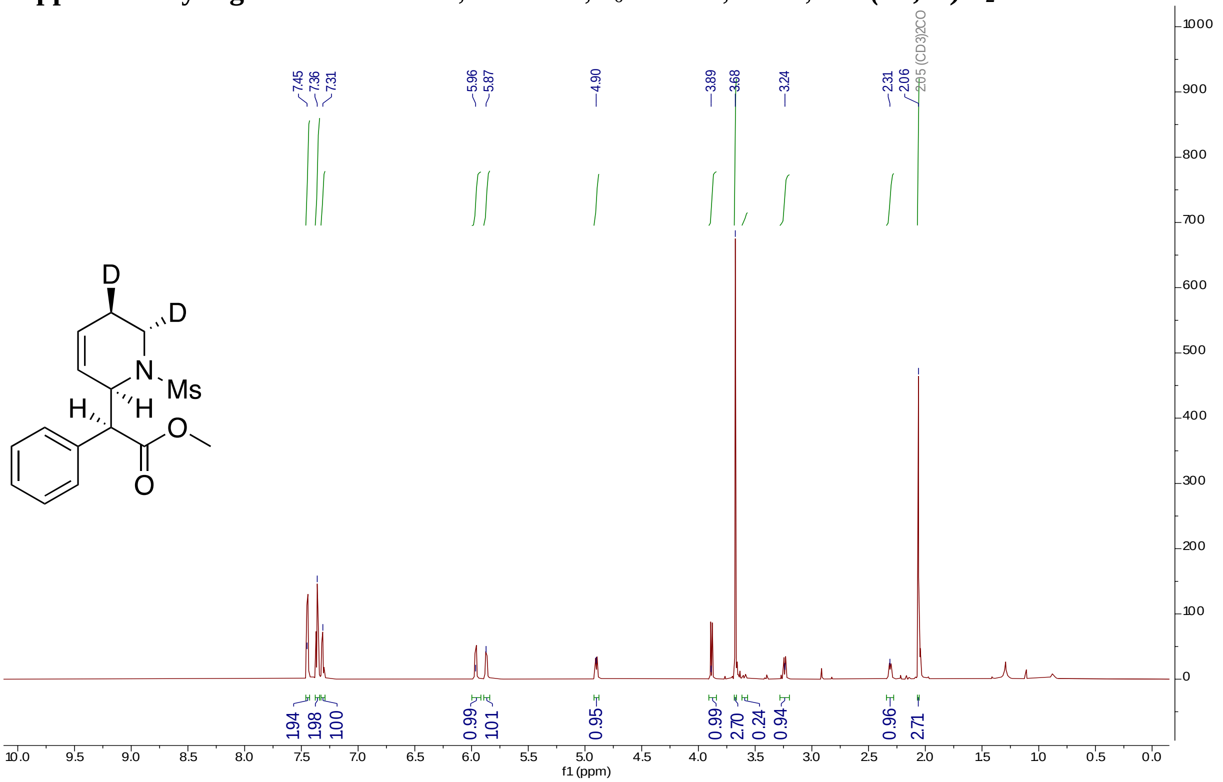

Supplementary Figure 110: ^{13}C NMR, 200 MHz, d$_6$-acetone, 25 °C, ***Rel-(5R,6S)-d$_2$*-15**

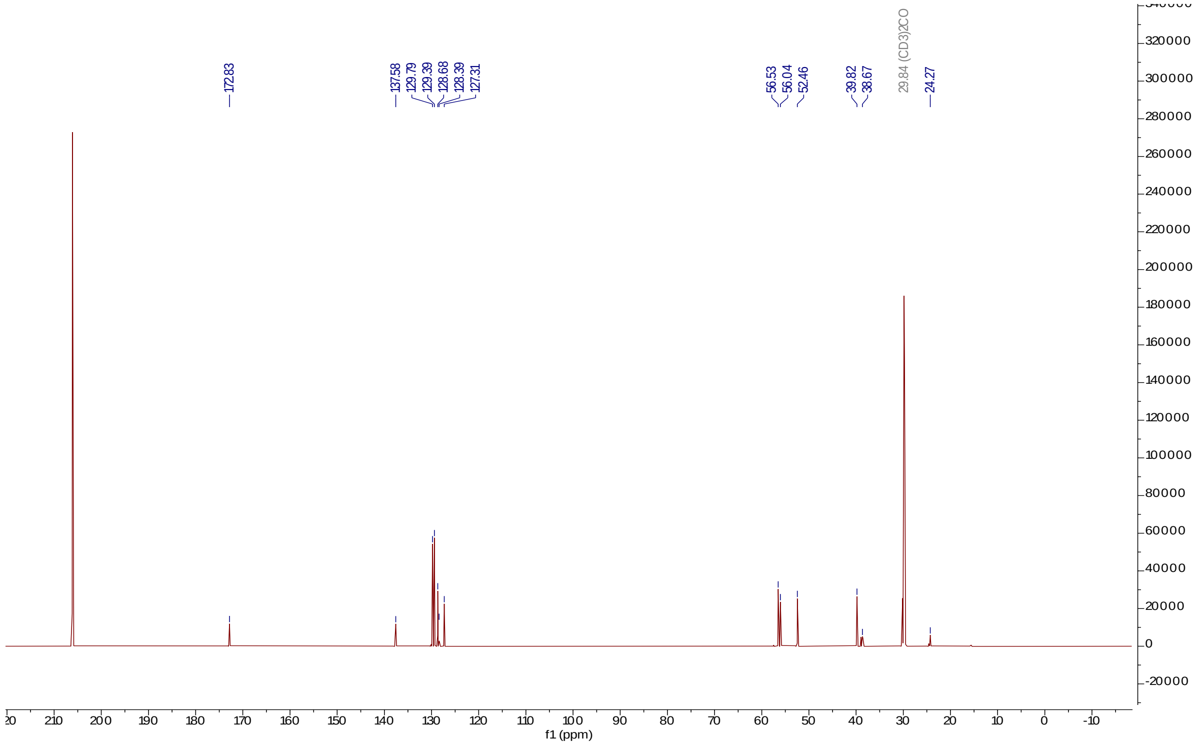

Supplementary Figure 111: ^{1}H NMR, 800 MHz, d_6-acetone, 25 °C, ***Rel-(5S,6R)-d$_5$-15***

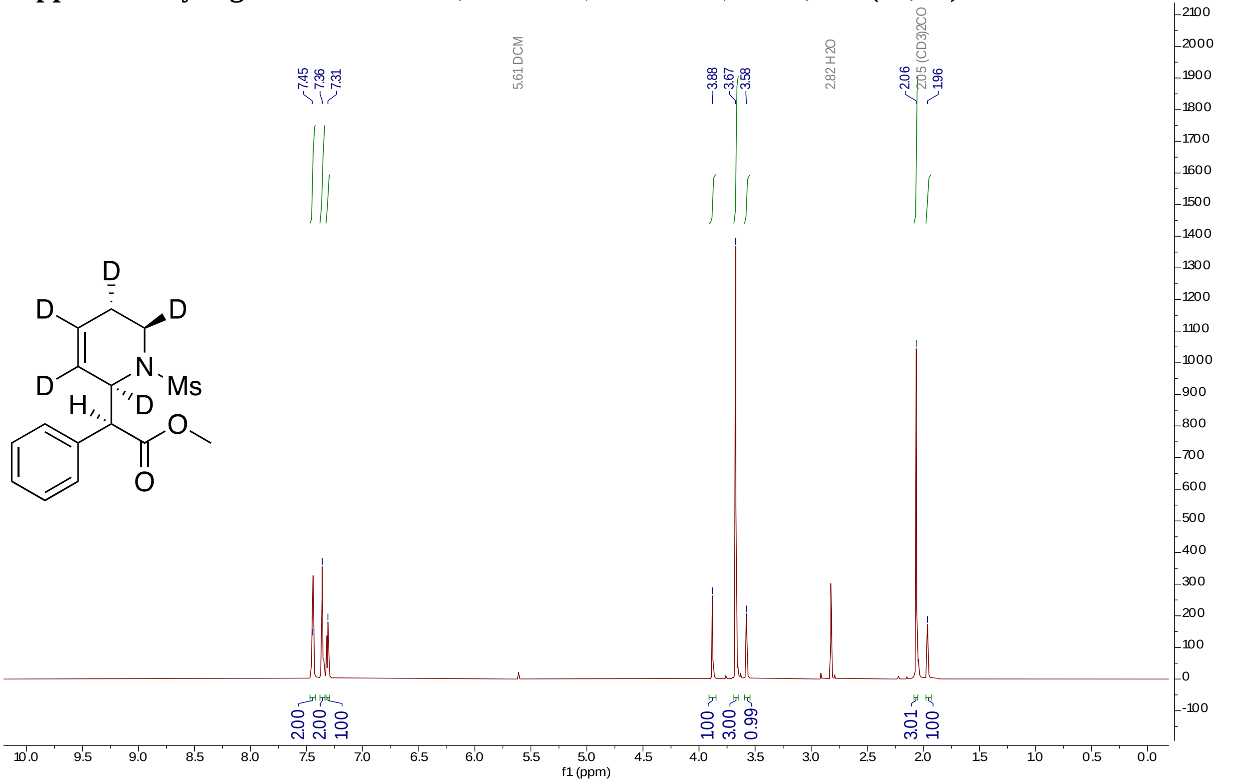

Supplementary Figure 112: ^{13}C NMR, 200 MHz, d_6-acetone, 25 °C, ***Rel-(5S,6R)-d$_5$-15***

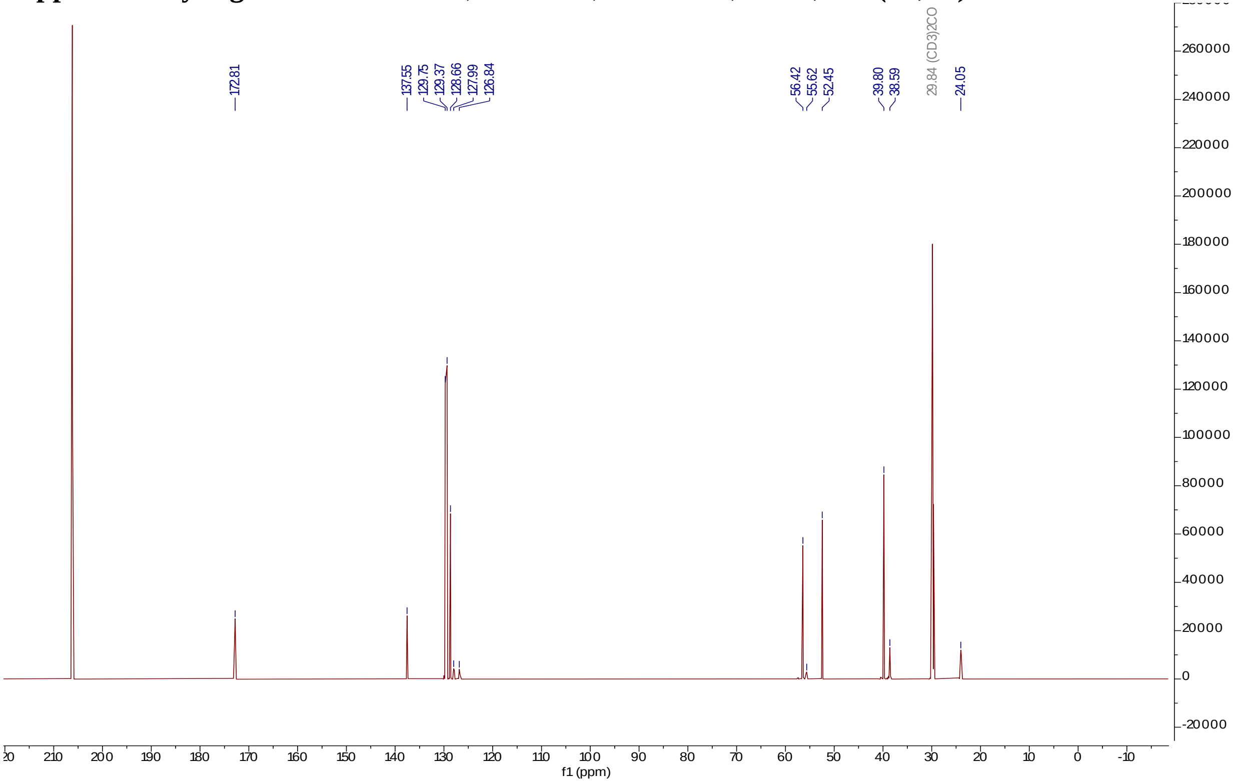

Supplementary Figure 113: ^{1}H NMR, 800 MHz, d_6-acetone, 25 °C, ***Rel-(6R)-d_6-15***

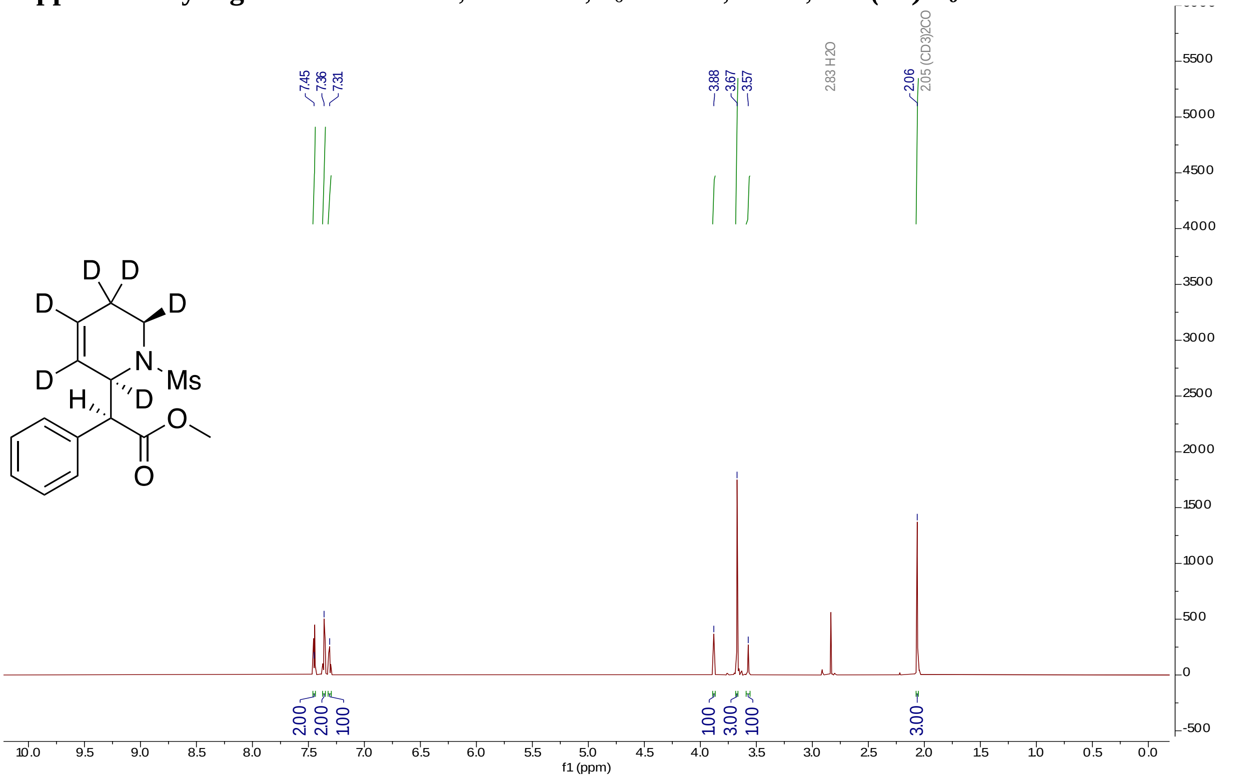

Supplementary Figure 114: ^{13}C NMR, 200 MHz, d_6-acetone, 25 °C, ***Rel-(6R)-d_6-15***

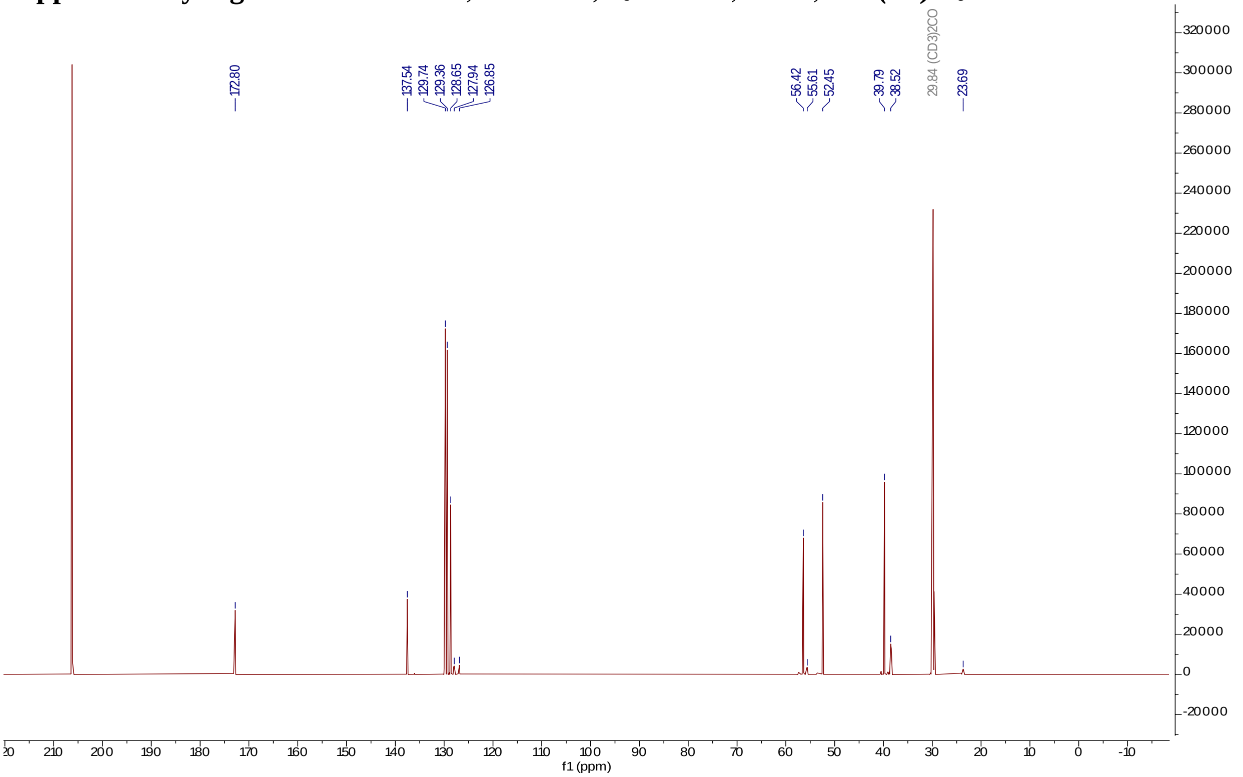

Supplementary Figure 115: ^{1}H NMR, 800 MHz, d_6-acetone, 25 °C, ***Rel-(5S)-d₆-15***

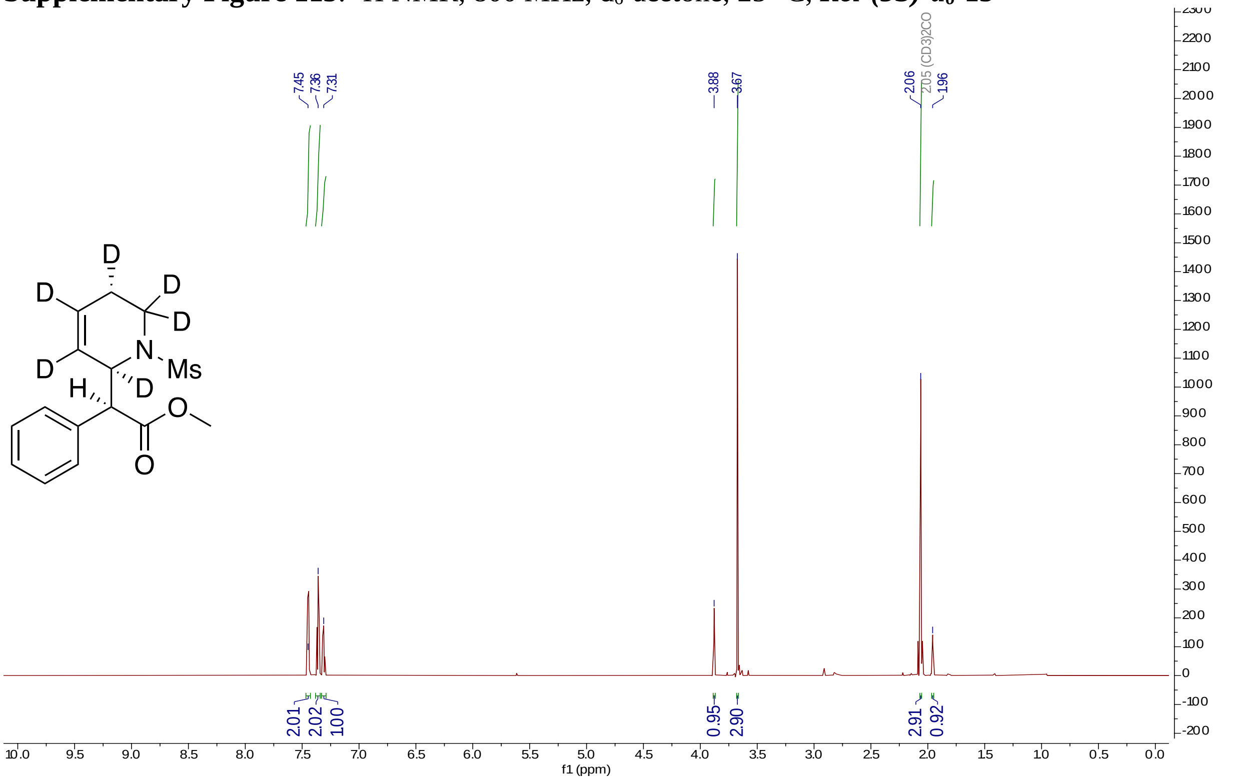

Supplementary Figure 116: ^{13}C NMR, 200 MHz, d_6-acetone, 25 °C, ***Rel-(5S)-d₆-15***

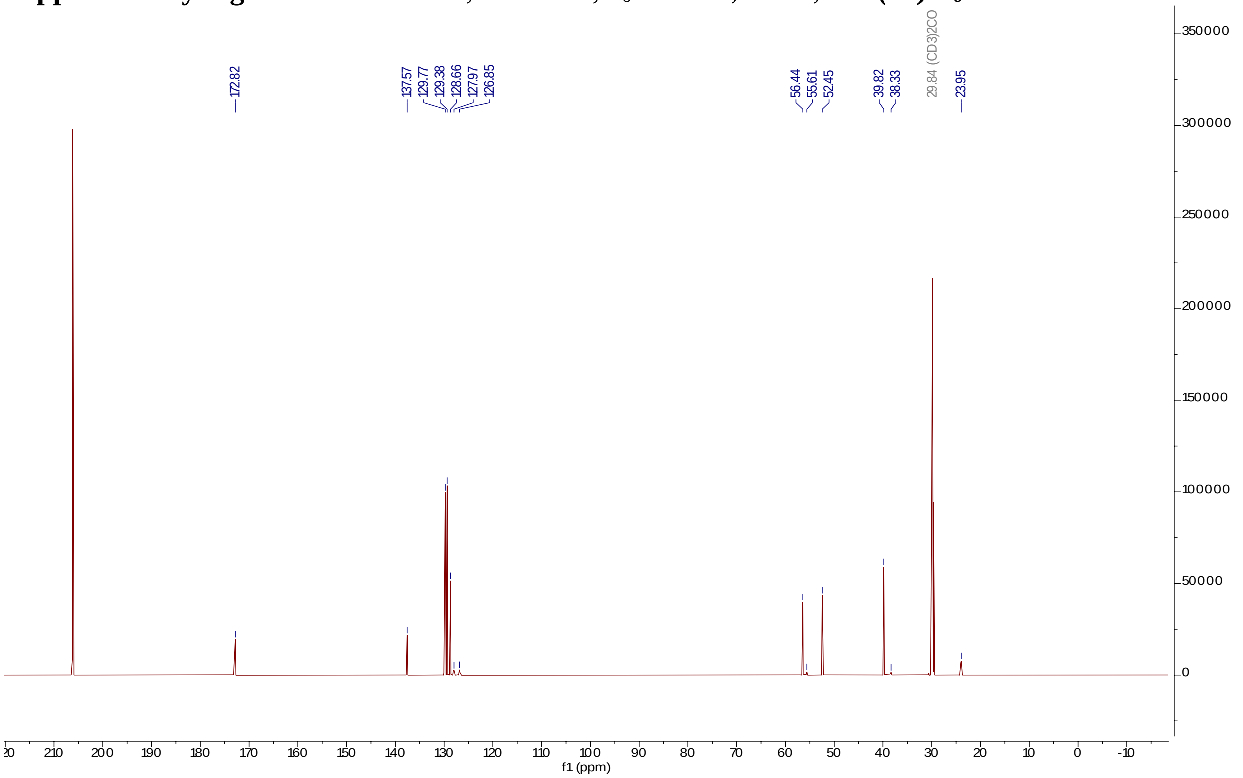

Supplementary Figure 117: ^{1}H NMR, 800 MHz, CD_2Cl_2, 25 °C, ***d*$_7$-15**

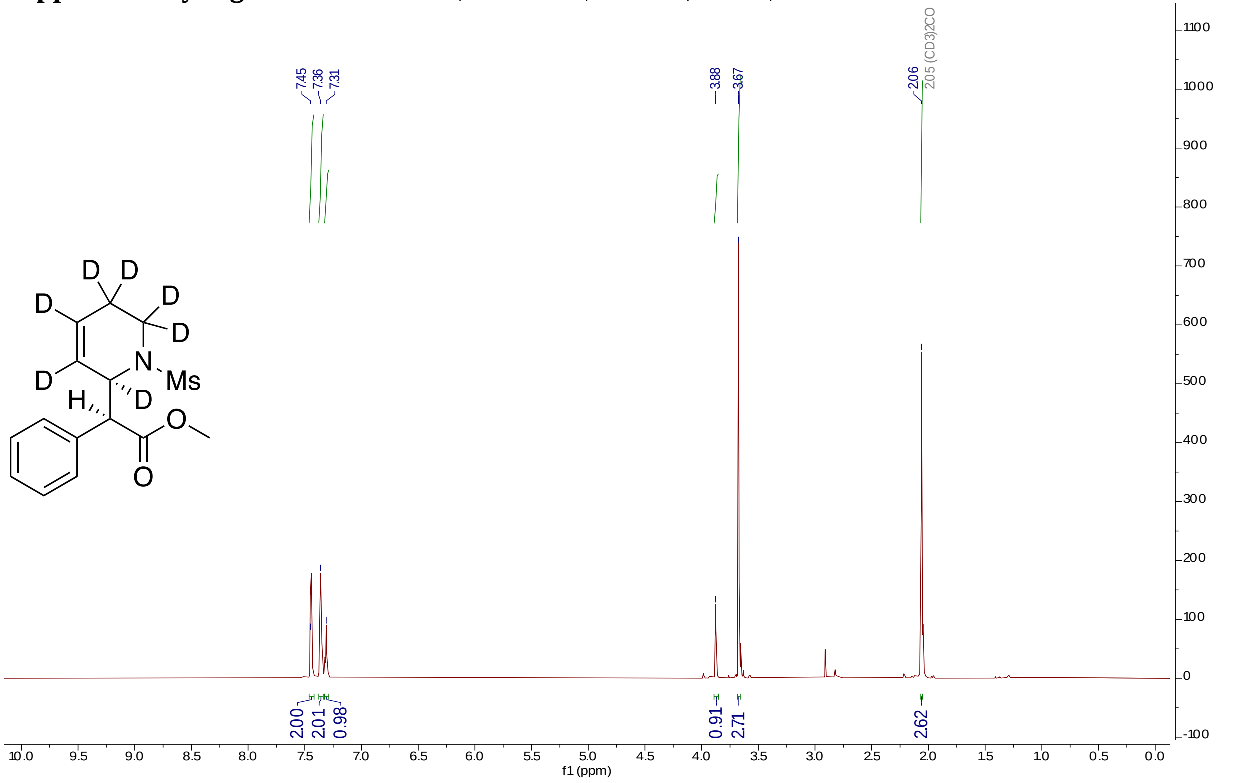

Supplementary Figure 118: ^{13}C NMR, 200 MHz, d_6-acetone, 25 °C, ***d*$_7$-15**

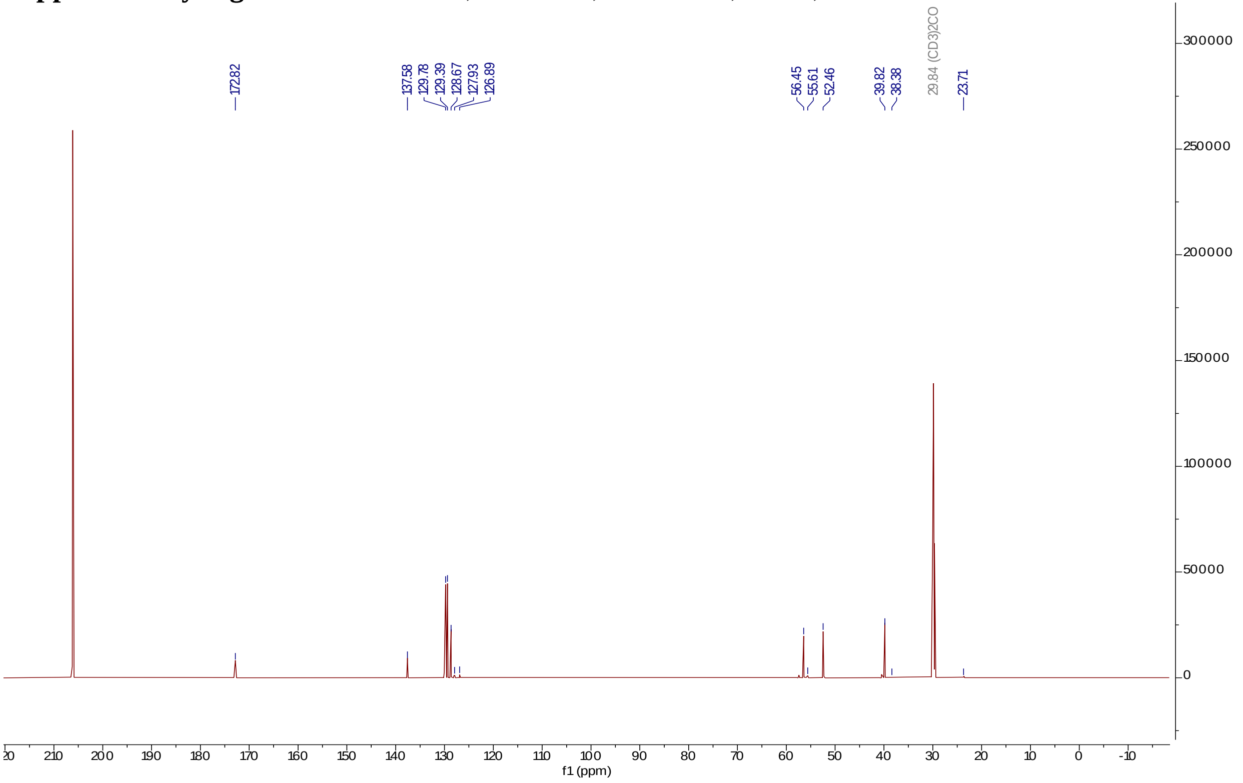

Supplementary Figure 119: ^{1}H NMR, 800 MHz, CD_2Cl_2, 25 °C, **16**

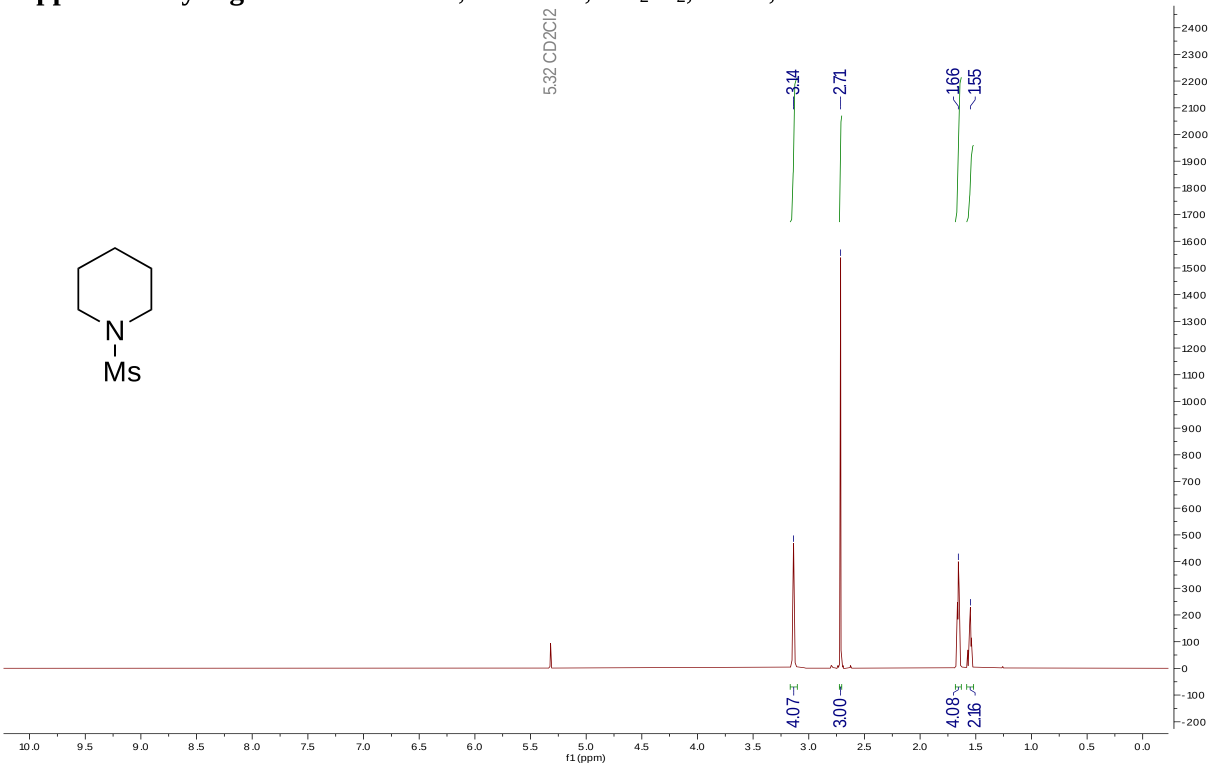

Supplementary Figure 1120: ^{13}C NMR, 200 MHz, CD_2Cl_2, 25 °C, **16**

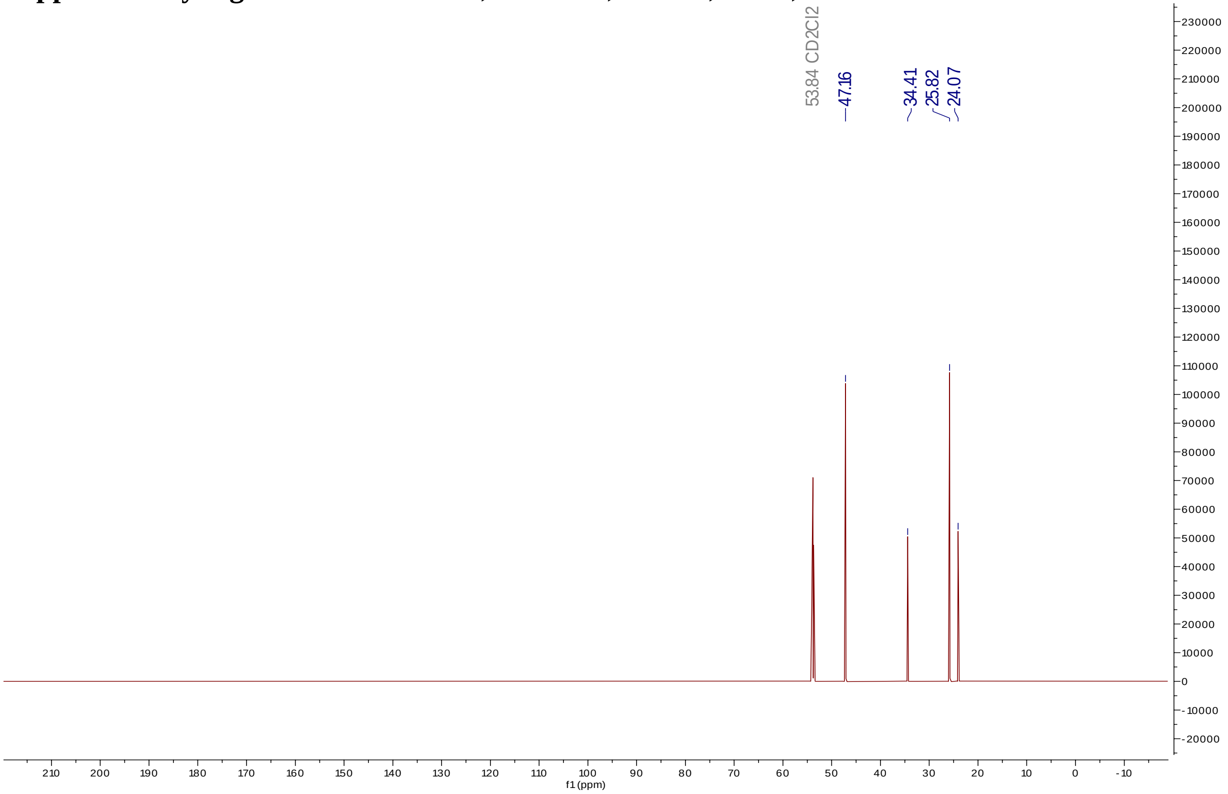

Supplementary Figure 121: ^{13}C NMR, 800 MHz, d_6-acetone, 25 °C, Attempted hydrogenation of ***(6'R)-d$_6$-15***

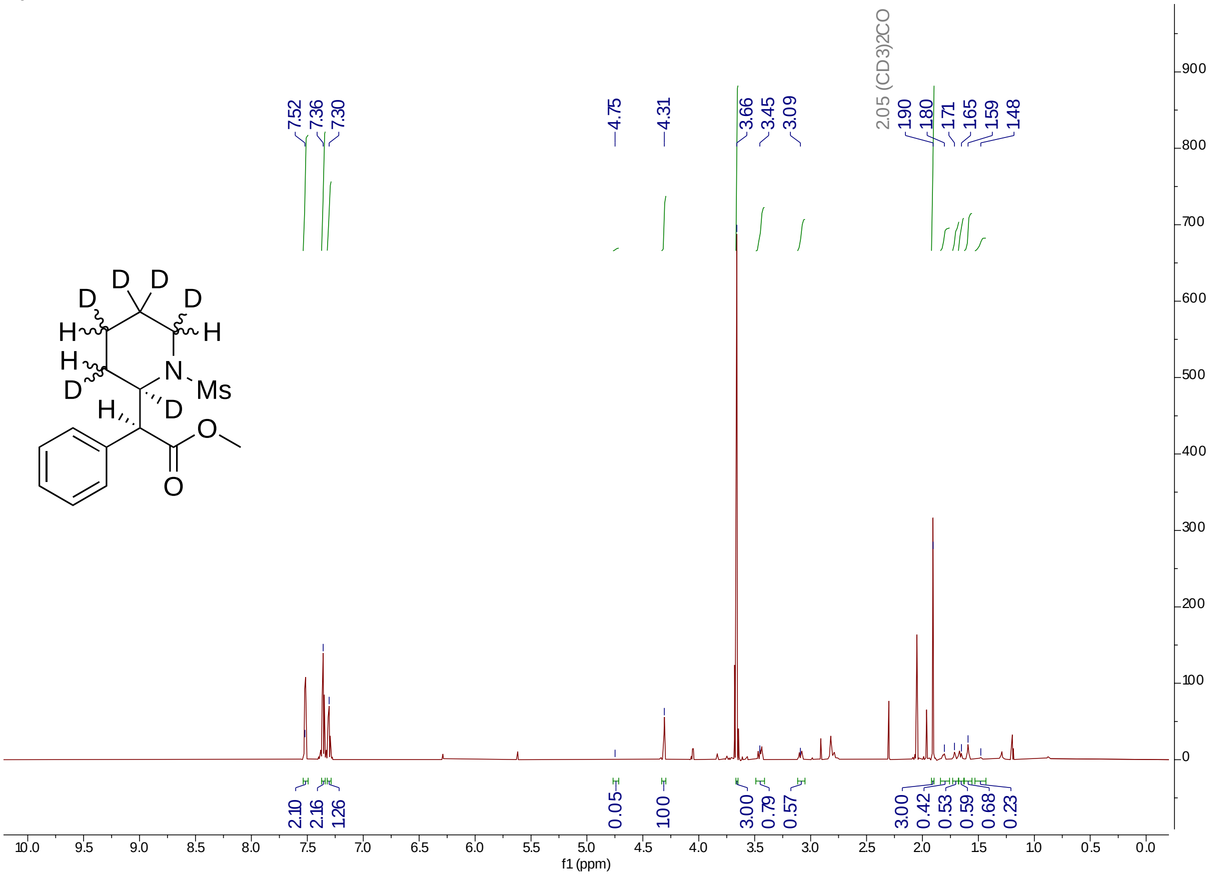

SC-XRD DATA:

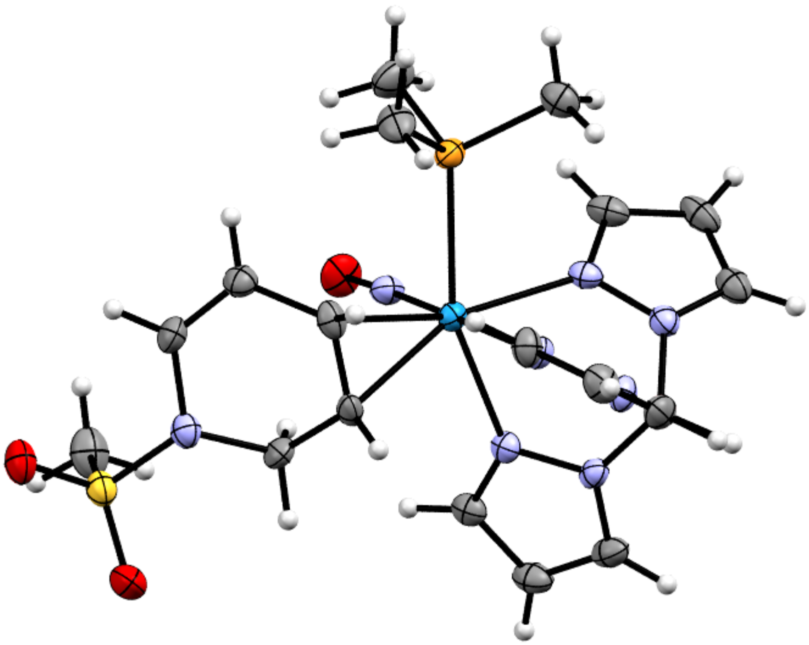

Supplementary Figure 122: ORTEP/ellipsoid diagram of **5**.

Supplementary Table 1: SC-XRD data for **5**.

CCDC	Chemical Formula	FW (g/mol)
2298721	$C_{18}H_{28}BN_8O_3PSW$	662.17
T (K)	**λ** (Å)	Crystal size (mm)
100(2)	0.71073	0.056 x 0.129 x 0.159
Crystal habit	Crystal system	Space group
Colorless plate	Monoclinic	$P2_1/n$
a (Å)	b (Å)	c (Å)
9.8554(10)	19.675(2)	13.2017(13)
α (°)	β (°)	γ (°)
90	106.438(3)	90
V (Å^3)	Z	ρ_{calc} (g/cm^3)
2455.2(4)	4	1.791
μ (mm^{-1})	F(000)	θ range (°)
4.892	1304	1.91 to 28.35
Index ranges	Data/restraints/parameters	Goodness-of-fit on F^2
$-13 \leq h \leq 13$<br>$-26 \leq k \leq 26$<br>$-17 \leq l \leq 17$	6127 / 0 / 314	1.011
R_1 [I>2σ(I)]	wR_2 [all data]	
0.0348	0.0672	

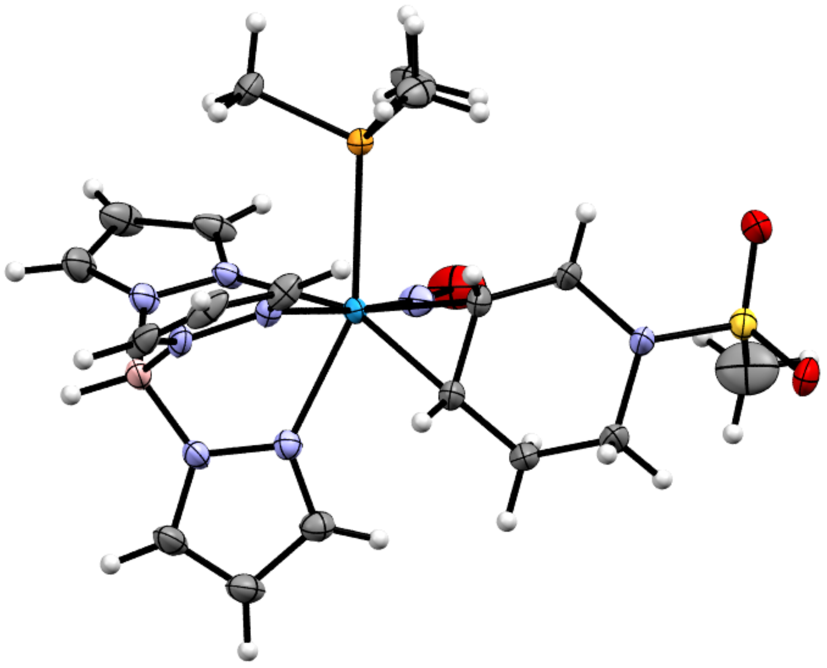

Supplementary Figure 123: ORTEP/ellipsoid diagram of **7**.

Supplementary Table 2: SC-XRD data for **7**.

CCDC	Chemical Formula	FW (g/mol)
2298722	$C_{18}H_{30}BN_8O_3PSW$	664.19
T (K)	λ (Å)	Crystal size (mm)
100(2)	0.71073	0.077 x 0.129 x 0.141
Crystal habit	Crystal system	Space group
Yellow prism	Monoclinic	P 2_1/c
a (Å)	b (Å)	c (Å)
7.9593(4)	25.6443(14)	12.5253(7)
α (°)	β (°)	γ (°)
90	103.734(2)	90
V ($Å^3$)	Z	ρ_{calc} (g/cm^3)
2483.5(2)	4	1.776
μ (mm^{-1})	F(000)	θ range (°)
4.837	1312	2.31 to 30.05
Index ranges	Data/restraints/parameters	Goodness-of-fit on F^2
$-11 \leq h \leq 11$<br>$-36 \leq k \leq 35$<br>$-17 \leq l \leq 16$	7241 / 0 / 314	1.229
R_1 [I>2σ(I)]	wR_2 [all data]	
0.0277	0.0527	

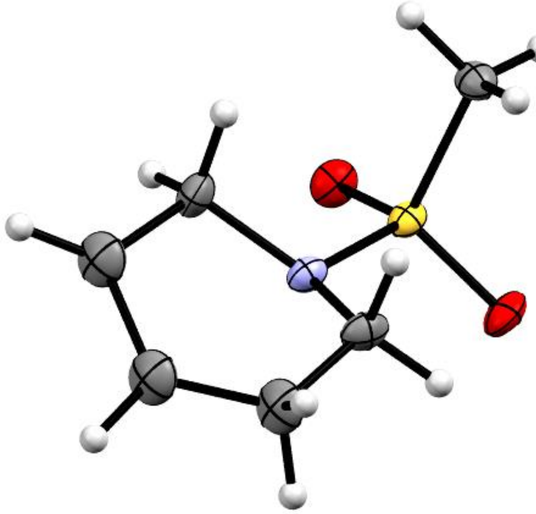

Supplementary Figure 124: ORTEP/ellipsoid diagram of **14**.

Supplementary Table 3: SC-XRD data for **14**.

CCDC	Chemical Formula	FW (g/mol)
2298723	$C_6H_{11}NO_2S$	161.22
T (K)	λ (Å)	Crystal size (mm)
100(2)	0.71073	0.087 x 0.095 x 0.266
Crystal habit	Crystal system	Space group
Colorless plate	Monoclinic	P 2_1/c
a (Å)	b (Å)	c (Å)
14.2783(10)	5.6636(4)	9.7732(6)
α (°)	β (°)	γ (°)
90	100.875(3)	90
V ($Å^3$)	Z	ρ_{calc} (g/cm^3)
776.13(9)	4	1.380
μ (mm^{-1})	F(000)	θ range (°)
0.357	344	2.90 to 30.54
Index ranges	Data/restraints/parameters	Goodness-of-fit on F^2
$-20 \leq h \leq 19$<br>$-7 \leq k \leq 8$<br>$-13 \leq l \leq 13$	2356 / 0 / 90	1.163
R_1 [I>2σ(I)]	wR_2 [all data]	
0.0583	0.1178	

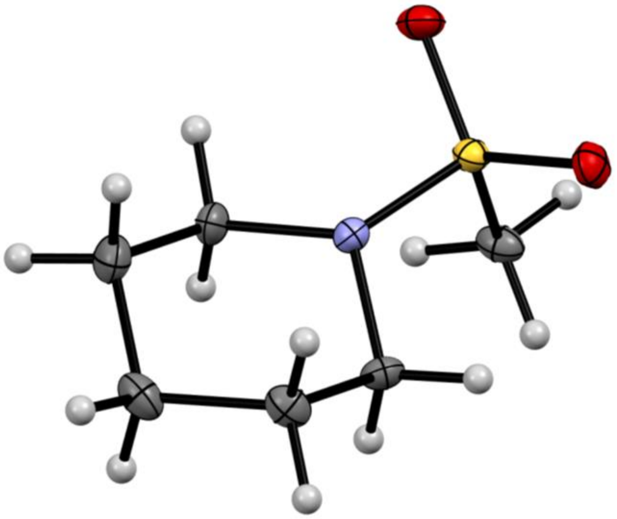

Supplementary Figure 125: ORTEP/ellipsoid diagram of **16**.

Supplementary Table 4: SC-XRD data for **16**.

CCDC	Chemical Formula	FW (g/mol)
2298724	$C_6H_{13}NO_2S$	163.23
T (K)	**λ** (Å)	Crystal size (mm)
100(2)	0.71073	0.046 x 0.150 x 0.195
Crystal habit	Crystal system	Space group
Colorless plate	Monoclinic	$P\ 2_1/c$
a (Å)	b (Å)	c (Å)
14.2951(7)	5.6581(2)	10.0681(5)
α (°)	β (°)	γ (°)
90	100.777(2)	90
V (Å^3)	Z	ρ_{calc} (g/cm^3)
799.98(6)	4	1.355
μ (mm^{-1})	F(000)	θ range (°)
0.347	352	2.90 to 28.26
Index ranges	Data/restraints/parameters	Goodness-of-fit on F^2
$-19 \leq h \leq 19$<br>$-7 \leq k \leq 6$<br>$-13 \leq l \leq 13$	1980 / 0 /92	1.264
R_1 [I>2σ(I)]	wR_2 [all data]	
0.0521	0.0964	

MOLECULAR ROTATIONAL RESONANCE SPECTROSCOPY DATA
Prepared by: Brooks H. Pate

This section describes the methods for identifying deuterated versions of (N-mesyl)-tetrahydropyridine (**14**) (simply called THP in this document) using broadband molecular rotational resonance (MRR) spectroscopy. A future manuscript will provide details on the analysis methods. This document summarizes the essential analysis methods and results.

Subsections:
(A) Structure Issues in the MRR Analysis
(B) Prediction of Rotational Spectra for Deuterated Samples
(C) Experimental Method and Illustration of Spectrum Analysis Approach
(D) Confidence in the Identification of Deuterated Analytes
(E) Sample Composition and Validation using NMR Spectroscopy

Molecular rotational resonance (MRR) spectroscopy measures the spectrum for allowed transitions between the quantized energy levels of free rotation of the analyte.[5] The energy levels depend on the principal moments-of-inertia for rotation about the center-of-mass. Each chemically distinct isotopic variant of the molecule has unique principal moments that are reported as the rotational constants (A, B, C) – a quantity inversely proportional to the principal moments-of-inertia.[6,7] In the analysis of deuterated versions of an analyte, an equilibrium geometry from quantum chemistry is used to predict the rotational constants for each unique isotopic variant. These predicted constants are used to calculate the rotational spectrum and the predicted spectrum is used to identify the species in the experiment. The steps in this process are described in this document.

(A) Structure Issues in the MRR Analysis

The THP analyte has two low-energy conformational isomers for the axial and equatorial position of the mesyl protecting group. These isomers are shown below in Fig. S126. The structures shown in Fig. S126 are the equilibrium geometries obtained using the B2PLYPD3 def2TVP method[8] in Gaussian 16.[9] Both conformers are identified in the rotational spectrum. The equatorial isomer has higher spectral intensity and is used to determine the sample composition. The synthetic chemistry performs deuteration of the THP for C-H bonds on the heterocycle ring. Deuteration on the mesyl group is not considered.

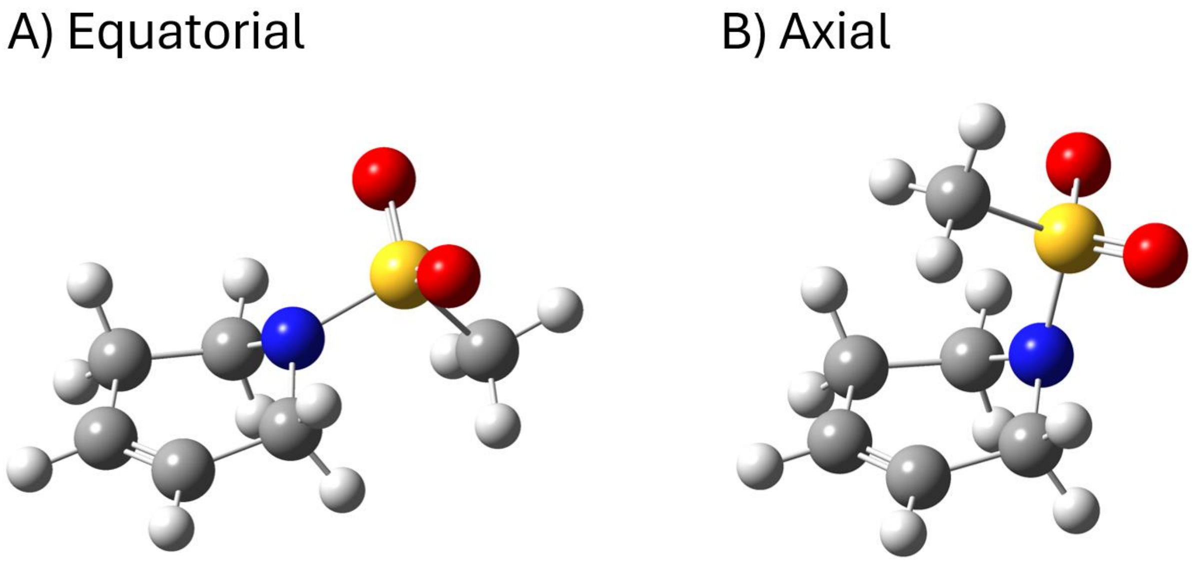

Supplementary Figure 126: The equilibrium geometries of the equatorial and axial conformational isomers of **14** are shown. Both conformers are observed in the pulsed-jet rotational spectrum. The composition analysis of deuterated versions of the sample use the spectrum from the equatorial conformer.

Placing a single deuterium at one of the three methylene sites creates a chiral center. In these cases, the transient chirality of the ring pucker geometry leads to two rotationally distinct isomers being present in the sample. The presence of two isomers due to the ring pucker is illustrated in Fig. S127 using one of the samples analyzed in this work. For the incorporation of deuterium and any of the 6 methylene positions, there are $2^6 = 64$ isomers. However, there are only 36 chemically distinct deuterated species when the ring pucker structural element is considered.

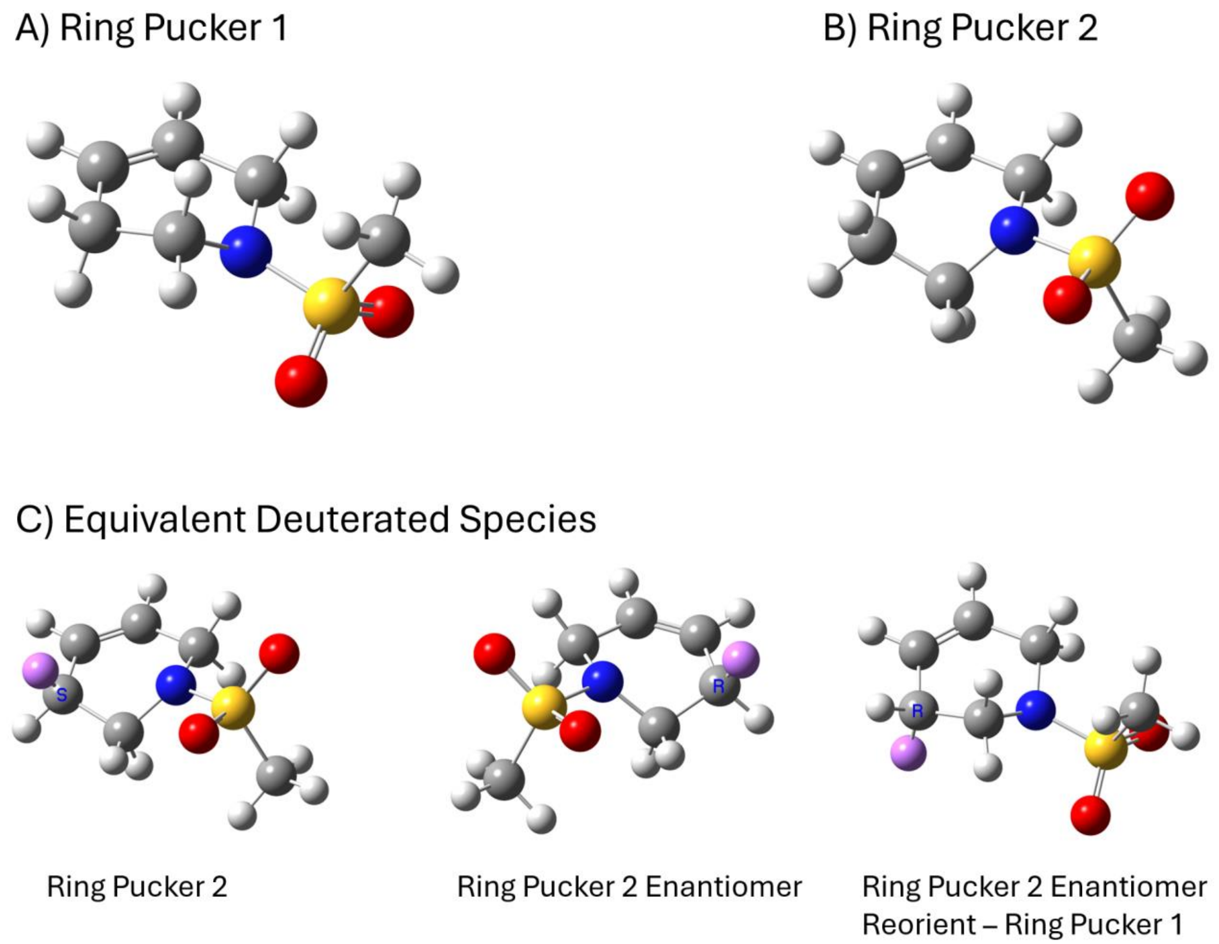

Supplementary Figure 127: The structural features of the ring pucker transient chirality are illustrated. Panel A and B show the two ring pucker isomers which change the position of the nitrogen from below-to-above the ring. A molecule with ring pucker 2 geometry and a single deuteration site – creating a chiral center – is shown in C. The mirror image of this structure – which is the enantiomer of the original structure – is shown in the middle. When the enantiomer is rearranged, it is seen that this enantiomer has the opposite ring pucker transient chirality – i.e. the ring pucker 1 geometry – and the isotopically chiral center has its absolute configuration changed. Because enantiomers have identical rotational constants, this result shows that the rotational constants for any ring pucker 2 geometry can be calculated from the ring pucker 1 geometry by inverting the chirality at any isotopically chiral carbon atoms.

The starting point of isotopic analysis is a spectroscopic assignment of the undeuterated molecule, THP-d_0. The MRR spectrum has the characteristic fine structure associated with the ^{14}N nuclear quadrupole splitting. Estimates of the nuclear quadrupole spectroscopic parameters are available from the quantum chemistry calculations. The results of a fit of the MRR spectrum of THP-d_0 are given in Table 1 where the experimental parameters are compared to the estimates from quantum chemistry. This analysis is used to improve the predicted spectra of possible deuterated versions of THP through scaling the theoretical estimates of the molecular rotational constants. As spectra of deuterated versions of THP became available, the scale factors were refined by taking the average over the different measurements. The final average scale factors used to predict the rotational constants of deuterated THP samples are also given in Supplementary Table 5.

Supplementary Table 5: The Theory[(1)] and Fit Rotational Constants for ***d₀*-14** using A-Reduction of the Watson Hamiltonian (Ir-Representation)

Parameter	Experiment	Theory	Scale Factor	Average Scale[(2)]
A / MHz	2348.90935(17)[(3)]	2344.73	1.00178	1.00171
B / MHz	852.648910(88)	850.95	1.00199	1.00217
C / MHz	750.070070(69)	748.35	1.00229	1.00236
Δ_J / kHz	0.02250(77)			
Δ_{JK} / kHz	0.1190(32)			
Δ_K / kHz	[0][(4)]			
δ_J / kHz	0.00314(25)			
δ_K / kHz	-0.245(14)			
1.5 χ_{aa} / MHz	3.9732(22)	4.225	0.940	
0.25(χ_{bb}-χ_{cc}) / MHz	1.78600(62)	1.872	0.954	
$N_{transitions}$	232			
σ rms / kHz	3.6			

(1): The theory value for the dipole moment vector in the principal axis system is: (2.86D, -0.18D, 3.85 D)
(2): The average scale factor is obtained from the fits to all synthetic targets analyzed in this study.
(3): The values in parenthesis are the 1σ uncertainty in the last two digits for the fit value.
(4): This parameter is not fit and held at zero.

(B) Prediction of Rotational Spectra for Deuterated Samples

The analysis method uses predictions of the rotational spectra for different deuterated versions of THP and this methodology is described in this section.[10,11] As shown in the next section, it is possible to predict the rotational transition frequencies to about 1 MHz accuracy which makes it straightforward to identify the rotational spectrum of a selected deuterated THP molecule. The crucial issue of whether the assigned spectrum can confidently be identified with a single isotopic variant of THP is addressed in Section D.

For THP analysis, we rely on the equilibrium geometry of the equatorial conformer to predict the spectrum. Under the Born-Oppenheimer approximation, the equilibrium geometry of all isotopic variants is the same. The initial estimate of the rotational spectrum calculates the rotational constants for a selected deuteration pattern by simply changing the nuclear masses in the equilibrium geometry. The rotational constants are obtained by diagonalizing the inertia tensor of this deuterated THP to get the principal moments-of-inertia. Note that the orientation of the principal axis system will rotate for each different deuteration pattern. The rotation matrix that diagonalizes the inertia tensor is also used to transform the nuclear quadrupole hyperfine tensor and the dipole moment vector to give these quantities in the deuteration pattern specific principal axis system.

The accuracy of the spectrum prediction is increased by scaling the spectroscopic constants calculated from the theoretical equilibrium geometry. The scale factors for the rotational constants are reported in Supplementary Table 5. The first-order nuclear quadrupole constants are scaled by the ratio of the experimental values and the theoretical values for THP-d_0. The distortion constants for the spectrum predictions remain fixed at the values reported in Supplementary Table 5 for THP-d_0 – this is a common approximation used in rotational spectroscopy. Note that the effects of vibrational anharmonicity in the C-H bond, which makes the C-D bond slightly shorter, are not considered in the spectrum prediction.

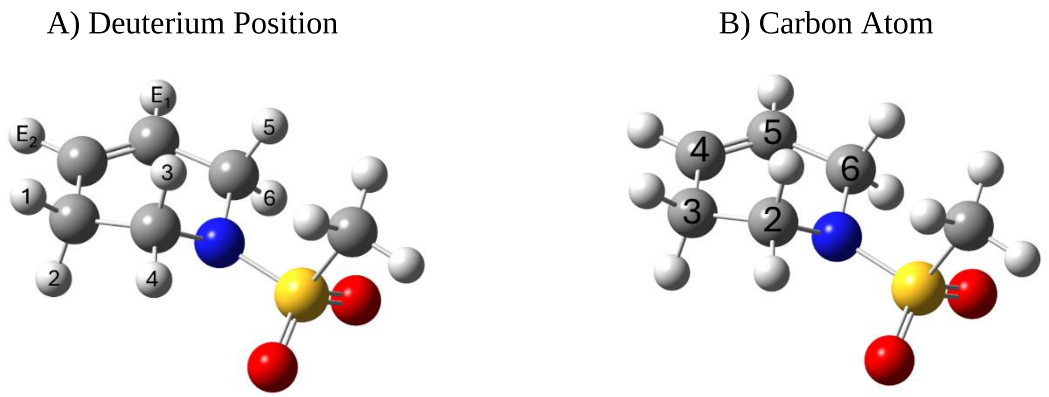

Supplementary Figure 128: A) The labeling convention for the hydrogen atom sites that can be deuterated in the reaction chemistry is shown for the ring pucker 1 geometry of Fig. S127. As described in Fig. S127, the rotational constants for the ring pucker 2 isomers of any deuteration pattern can be calculated from the ring pucker 1 geometry by changing the absolute configuration at any isotopically chiral carbon atoms. B) The reaction products are named based on the carbon atom that has the deuterium substitution. The labeling of the carbon atoms follows the standard scheme for heterocycle rings where the nitrogen is position 1.

The rotational constants and nitrogen quadrupole constants are calculated for all chemically distinct deuterated version of THP (with deuteration restricted to the heterocycle ring). The six methylene hydrogen positions can be substituted for deuterium in the synthesis. In this analysis, a specific deuteration of THP is specified by indicating whether a hydrogen (0) or deuterium (1) is placed at each of these substitution sites. The site numbering convention is indicated in Fig. S128. In cases where there is one or more chiral centers created by placing a single deuterium on a methylene carbon, there are two ring pucker isomers that will be present in the pulsed jet sample. The predicted spectroscopic constants for these ring-pucker pairs are kept grouped together. The rotational constants used to predict the spectrum of a chosen deuteration pattern are reported in Tables S6-9. Each table lists the 36 chemically distinct deuteration patterns (with ring-pucker isomers) for a specific deuteration condition on the ethylene carbons. These sets are: 1) Both ethylene carbon positions have a hydrogen atom – this is called the low-deuteration case. 2) Both ethylene carbon positions have a deuterium atom – this is called the high-deuteration case. 3) Ethylene position 1 has a deuterium. 4) Ethylene position 2 has a deuterium.

Supplementary Table 6: Scaled Predictions of the Rotational Constants and ^{14}N Nuclear Quadrupole Constants for the 36 Chemically Distinct Deuterated Versions of THP in the Low Deuteration Samples with the Two Ring Pucker Isomer.

Total Number of Deuterium Substitutions	Deuterium Isotope Pattern for Ring Pucker 1						A_{pred} (MHz)	B_{pred} (MHz)	C_{pred} (MHz)	$(1.5*\chi_{aa})_{pred}$ (MHz)	$0.25*(\chi_{bb}-\chi_{cc})_{pred}$ (MHz)	Deuterium Isotope Pattern for Ring Pucker 2						A_{pred} (MHz)	B_{pred} (MHz)	C_{pred} (MHz)	$(1.5*\chi_{aa})_{pred}$ (MHz)	$0.25*(\chi_{bb}-\chi_{cc})_{pred}$ (MHz)
0	0	0	0	0	0	0	2348.742	852.80	750.12	3.97	1.79	0	0	0	0	0	0	2348.74	852.80	750.12	3.97	1.79
1	0	0	1	0	0	0	2310.609	847.68	747.64	3.99	1.80	0	0	0	1	0	0	2303.74	852.52	745.45	3.97	1.79
1	0	0	0	0	0	1	2301.985	852.20	745.27	3.97	1.79	0	0	0	0	1	0	2303.32	850.00	746.59	3.98	1.77
1	1	0	0	0	0	0	2304.953	840.61	736.53	3.97	1.80	0	1	0	0	0	0	2305.43	844.02	741.88	3.94	1.76
2	0	1	0	1	0	0	2262.539	843.70	737.40	3.94	1.77	1	0	1	0	0	0	2268.96	835.69	734.17	3.99	1.81
2	0	0	0	1	1	0	2259.618	849.70	741.95	3.98	1.77	0	0	1	0	0	1	2264.91	847.15	742.76	3.99	1.81
2	0	1	0	0	0	1	2259.857	843.43	737.15	3.94	1.77	1	0	0	0	1	0	2260.54	837.93	733.14	3.98	1.78
2	0	0	0	0	1	1	2258.728	849.33	741.88	3.98	1.78	0	0	0	0	1	1	2258.73	849.33	741.88	3.98	1.78
2	0	1	1	0	0	0	2268.662	838.88	739.61	3.96	1.78	1	0	0	1	0	0	2262.33	840.34	732.08	3.97	1.80
2	0	0	1	1	0	0	2267.414	847.45	743.00	3.99	1.81	0	0	1	1	0	0	2267.41	847.45	743.00	3.99	1.81
2	0	1	0	0	1	0	2260.823	841.44	738.31	3.95	1.74	1	0	0	0	0	1	2259.08	840.11	731.80	3.97	1.80
2	0	0	0	1	0	1	2258.169	851.95	740.59	3.97	1.80	0	0	1	0	1	0	2266.66	844.86	744.19	4.00	1.79
2	1	1	0	0	0	0	2264.516	831.99	728.76	3.94	1.78	1	1	0	0	0	0	2264.52	831.99	728.76	3.94	1.78
3	0	1	1	0	1	0	2225.487	836.28	736.13	3.97	1.76	1	0	0	1	0	1	2217.58	839.86	727.35	3.97	1.80
3	1	0	1	1	0	0	2227.988	835.46	729.76	3.99	1.81	0	1	1	1	0	0	2227.47	838.60	735.15	3.96	1.79
3	0	0	1	0	1	1	2223.047	844.25	739.46	4.00	1.79	0	0	0	1	1	1	2216.14	849.05	737.23	3.98	1.78
3	1	0	0	1	1	0	2219.132	837.64	728.72	3.98	1.78	0	1	1	0	0	1	2224.11	838.35	734.84	3.96	1.79
3	1	0	0	0	1	1	2216.777	837.36	728.55	3.98	1.79	0	1	0	0	1	1	2217.36	840.77	733.73	3.95	1.75
3	1	1	0	1	0	0	2223.811	831.68	724.48	3.94	1.78	1	1	1	0	0	0	2229.73	827.05	726.60	3.96	1.79
3	0	0	1	1	1	0	2224.680	844.60	739.59	4.00	1.79	0	0	1	1	0	1	2222.84	846.93	738.12	3.99	1.81
3	1	0	1	0	1	0	2225.930	832.98	730.87	4.00	1.80	0	1	0	1	0	1	2218.08	843.13	732.65	3.94	1.78
3	1	1	0	0	1	0	2220.864	829.51	725.34	3.95	1.76	1	1	0	0	0	1	2219.77	831.49	724.14	3.94	1.78
3	1	0	1	0	0	1	2224.092	835.24	729.42	3.99	1.81	0	1	0	1	1	0	2219.17	841.09	733.86	3.95	1.75
4	0	1	1	1	1	0	2185.451	835.97	731.71	3.97	1.77	1	0	1	1	0	1	2184.19	835.03	725.00	3.99	1.82
4	1	0	1	1	1	0	2186.103	832.73	726.48	4.00	1.80	0	1	1	1	0	1	2183.98	838.09	730.38	3.96	1.80
4	0	0	1	1	1	1	2182.124	844.02	734.85	4.00	1.80	0	0	1	1	1	1	2182.12	844.02	734.85	4.00	1.80
4	0	1	0	1	1	1	2176.757	840.44	729.26	3.95	1.76	1	0	1	0	1	1	2183.10	832.47	726.24	4.00	1.80
4	1	0	0	1	1	1	2176.422	837.09	724.12	3.98	1.79	0	1	1	0	1	1	2182.97	835.67	731.51	3.97	1.77
4	1	1	0	1	1	0	2181.322	829.17	721.09	3.95	1.76	1	1	1	0	0	1	2185.95	826.60	721.95	3.96	1.80
4	1	1	0	0	1	1	2178.174	828.93	720.85	3.95	1.77	1	1	0	0	1	1	2178.17	828.93	720.85	3.95	1.77
4	1	1	1	0	1	0	2187.424	824.55	723.25	3.97	1.77	1	1	0	1	0	1	2180.12	831.20	719.85	3.94	1.79
4	1	1	1	1	0	0	2190.576	826.78	722.35	3.96	1.80	1	1	1	1	0	0	2190.58	826.78	722.35	3.96	1.80
5	1	0	1	1	1	1	2144.273	832.24	721.85	4.00	1.81	0	1	1	1	1	1	2143.93	835.39	727.07	3.96	1.78
5	1	1	0	1	1	1	2139.623	828.62	716.59	3.95	1.77	1	1	1	0	1	1	2145.64	824.03	718.74	3.96	1.78
5	1	1	1	1	0	1	2147.808	826.36	717.69	3.96	1.80	1	1	1	1	1	0	2149.37	824.26	719.03	3.96	1.78
6	1	1	1	1	1	1	2108.523	823.76	714.51	3.96	1.79	1	1	1	1	1	1	2108.52	823.76	714.51	3.96	1.79

Supplementary Table 7: Scaled Predictions of the Rotational Constants and ^{14}N Nuclear Quadrupole Constants for the 36 Chemically Distinct Deuterated Versions of THP in the High Deuteration Samples with the Two Ring Pucker Isomer.

Total Number of Deuterium Substitutions	Deuterium Isotope Pattern for Ring Pucker 1						A_{pred} (MHz)	B_{pred} (MHz)	C_{pred} (MHz)	$(1.5*\chi_{aa})_{pred}$ (MHz)	$0.25*(\chi_{bb}-\chi_{cc})_{pred}$ (MHz)	Deuterium Isotope Pattern for Ring Pucker 2						A_{pred} (MHz)	B_{pred} (MHz)	C_{pred} (MHz)	$(1.5*\chi_{aa})_{pred}$ (MHz)	$0.25*(\chi_{bb}-\chi_{cc})_{pred}$ (MHz)
2	0	0	0	0	0	0	2299.725	818.16	718.72	3.97	1.78	0	0	0	0	0	0	2299.73	818.16	718.72	3.97	1.78
3	0	0	1	0	0	0	2262.366	813.60	716.51	3.99	1.80	0	0	0	1	0	0	2255.81	817.96	714.40	3.97	1.79
3	0	0	0	0	0	1	2255.743	817.62	714.36	3.97	1.79	0	0	0	0	1	0	2256.85	815.61	715.57	3.98	1.76
3	1	0	0	0	0	0	2255.182	807.49	706.43	3.97	1.79	0	1	0	0	0	0	2256.72	810.48	711.27	3.94	1.76
4	0	1	0	1	0	0	2214.858	810.23	707.12	3.94	1.77	1	0	1	0	0	0	2219.93	803.10	704.33	3.99	1.81
4	0	0	0	1	1	0	2214.165	815.38	711.28	3.98	1.77	0	0	1	0	0	1	2219.38	813.12	712.12	3.98	1.80
4	0	1	0	0	0	1	2213.871	809.94	707.01	3.94	1.77	1	0	0	0	1	0	2213.33	805.04	703.41	3.98	1.77
4	0	0	0	0	1	1	2214.836	815.01	711.34	3.98	1.77	0	0	0	0	1	1	2214.84	815.01	711.34	3.98	1.77
4	0	1	1	0	0	0	2220.739	805.88	709.25	3.96	1.78	1	0	0	1	0	0	2213.60	807.30	702.31	3.97	1.80
4	0	0	1	1	0	0	2220.200	813.44	712.23	3.98	1.80	0	0	1	1	0	0	2220.20	813.44	712.23	3.98	1.80
4	0	1	0	0	1	0	2214.654	808.12	708.09	3.95	1.74	1	0	0	0	0	1	2212.10	807.04	702.17	3.97	1.80
4	0	0	0	1	0	1	2212.942	817.44	710.03	3.97	1.79	0	0	1	0	1	0	2220.88	811.02	713.45	3.99	1.78
4	1	1	0	0	0	0	2215.027	799.92	699.39	3.94	1.77	1	1	0	0	0	0	2215.03	799.92	699.39	3.94	1.77
5	0	1	1	0	1	0	2180.022	803.50	706.15	3.96	1.76	1	0	0	1	0	1	2171.56	806.86	698.04	3.97	1.80
5	1	0	1	1	0	0	2179.949	802.94	700.24	3.99	1.81	0	1	1	1	0	0	2180.52	805.68	705.13	3.96	1.78
5	0	0	1	0	1	1	2179.791	810.48	709.18	3.99	1.79	0	0	0	1	1	1	2173.20	814.80	707.04	3.98	1.78
5	1	0	0	1	1	0	2172.900	804.83	699.30	3.98	1.78	0	1	1	0	0	1	2178.85	805.40	704.96	3.96	1.79
5	1	0	0	0	1	1	2172.164	804.52	699.26	3.98	1.78	0	1	0	0	1	1	2173.72	807.51	703.96	3.95	1.75
5	1	1	0	1	0	0	2175.309	799.70	695.42	3.94	1.78	1	1	1	0	0	0	2181.00	795.50	697.46	3.96	1.79
5	0	0	1	1	1	0	2179.869	810.84	709.19	3.99	1.79	0	0	1	1	0	1	2178.27	812.97	707.83	3.98	1.81
5	1	0	1	0	1	0	2179.393	800.62	701.38	3.99	1.79	0	1	0	1	0	1	2173.06	809.72	702.85	3.94	1.77
5	1	1	0	0	1	0	2173.933	797.64	696.34	3.95	1.75	1	1	0	0	0	1	2173.02	799.47	695.22	3.94	1.78
5	1	0	1	0	0	1	2177.794	802.69	700.04	3.98	1.81	0	1	0	1	1	0	2173.97	807.84	703.97	3.95	1.75
6	0	1	1	1	1	0	2140.902	803.27	702.05	3.96	1.77	1	0	1	1	0	1	2138.81	802.54	695.95	3.98	1.81
6	1	0	1	1	1	0	2140.492	800.44	697.31	3.99	1.80	0	1	1	1	0	1	2139.63	805.22	700.83	3.96	1.79
6	0	0	1	1	1	1	2139.775	810.31	704.91	3.99	1.79	0	0	1	1	1	1	2139.78	810.31	704.91	3.99	1.79
6	0	1	0	1	1	1	2134.022	807.26	699.82	3.95	1.76	1	0	1	0	1	1	2139.10	800.15	697.21	3.99	1.80
6	1	0	0	1	1	1	2132.719	804.33	695.15	3.98	1.79	0	1	1	0	1	1	2139.98	802.95	701.98	3.96	1.77
6	1	1	0	1	1	0	2135.316	797.39	692.39	3.95	1.76	1	1	1	0	0	1	2139.90	795.09	693.26	3.96	1.79
6	1	1	0	0	1	1	2133.787	797.12	692.29	3.95	1.76	1	1	0	0	1	1	2133.79	797.12	692.29	3.95	1.76
6	1	1	1	0	1	0	2141.177	793.20	694.47	3.96	1.77	1	1	0	1	0	1	2134.29	799.26	691.24	3.94	1.78
6	1	1	1	1	0	0	2142.790	795.31	693.52	3.96	1.79	1	1	1	1	0	0	2142.79	795.31	693.52	3.96	1.79
7	1	0	1	1	1	1	2101.139	799.99	693.13	3.99	1.80	0	1	1	1	1	1	2101.80	802.74	697.87	3.96	1.78
7	1	1	0	1	1	1	2096.101	796.89	688.33	3.95	1.77	1	1	1	0	1	1	2101.88	792.73	690.39	3.96	1.78
7	1	1	1	1	0	1	2102.634	794.92	689.31	3.96	1.80	1	1	1	1	1	0	2104.00	792.99	690.55	3.96	1.78
8	1	1	1	1	1	1	2065.588	792.53	686.46	3.96	1.78	1	1	1	1	1	1	2065.59	792.53	686.46	3.96	1.78

Supplementary Table 8: Scaled Predictions of the Rotational Constants and ^{14}N Nuclear Quadrupole Constants for the 36 Chemically Distinct Deuterated Versions of THP Deuterium at the E1 Position with the Two Ring Pucker Isomer.

Total Number of Deuterium Substitutions	Deuterium Isotope Pattern for Ring Pucker 1						A_{pred} (MHz)	B_{pred} (MHz)	C_{pred} (MHz)	$(1.5*\chi_{aa})_{pred}$ (MHz)	$0.25*(\chi_{bb}-\chi_{cc})_{pred}$ (MHz)	Deuterium Isotope Pattern for Ring Pucker 2						A_{pred} (MHz)	B_{pred} (MHz)	C_{pred} (MHz)	$(1.5*\chi_{aa})_{pred}$ (MHz)	$0.25*(\chi_{bb}-\chi_{cc})_{pred}$ (MHz)
1	0	0	0	0	0	0	2300.862	840.69	735.92	3.98	1.78	0	0	0	0	0	0	2300.86	840.69	735.92	3.98	1.78
2	0	0	1	0	0	0	2263.626	835.82	733.54	4.00	1.80	0	0	0	1	0	0	2256.92	840.46	731.38	3.98	1.79
2	0	0	0	0	0	1	2256.818	840.09	731.33	3.98	1.79	0	0	0	0	1	0	2258.06	837.96	732.58	3.99	1.76
2	1	0	0	0	0	0	2256.378	829.24	722.89	3.98	1.79	0	1	0	0	0	0	2257.63	832.43	728.02	3.95	1.76
3	0	1	0	1	0	0	2215.768	832.16	723.66	3.95	1.77	1	0	1	0	0	0	2221.27	824.54	720.63	4.00	1.81
3	0	0	0	1	1	0	2215.333	837.71	728.07	3.99	1.77	0	0	1	0	0	1	2220.56	835.28	728.92	4.00	1.81
3	0	1	0	0	0	1	2214.718	831.84	723.53	3.95	1.77	1	0	0	0	1	0	2214.56	826.62	719.69	3.99	1.78
3	0	0	0	0	1	1	2215.988	837.29	728.13	3.99	1.77	0	0	0	0	1	1	2215.99	837.29	728.13	3.99	1.77
3	0	1	1	0	0	0	2221.781	827.53	725.84	3.97	1.78	1	0	0	1	0	0	2214.79	829.02	718.56	3.98	1.80
3	0	0	1	1	0	0	2221.433	835.63	729.04	4.00	1.80	0	0	1	1	0	0	2221.43	835.63	729.04	4.00	1.80
3	0	1	0	0	1	0	2215.614	829.91	724.65	3.96	1.74	1	0	0	0	0	1	2213.20	828.73	718.41	3.98	1.80
3	0	0	0	1	0	1	2213.981	839.88	726.78	3.98	1.80	0	0	1	0	1	0	2222.20	833.06	730.29	4.01	1.78
3	1	1	0	0	0	0	2216.068	821.11	715.43	3.96	1.77	1	1	0	0	0	0	2216.07	821.11	715.43	3.96	1.77
4	0	1	1	0	1	0	2181.100	824.98	722.56	3.98	1.76	1	0	0	1	0	1	2172.65	828.53	714.07	3.98	1.80
4	1	0	1	1	0	0	2181.286	824.36	716.34	4.00	1.81	0	1	1	1	0	0	2181.56	827.30	721.51	3.97	1.79
4	0	0	1	0	1	1	2181.043	832.45	725.80	4.01	1.79	0	0	0	1	1	1	2174.31	837.06	723.60	3.99	1.78
4	1	0	0	1	1	0	2174.116	826.38	715.38	3.99	1.78	0	1	1	0	0	1	2179.81	826.99	721.33	3.97	1.79
4	1	0	0	0	1	1	2173.309	826.05	715.33	3.99	1.78	0	1	0	0	1	1	2174.62	829.24	720.30	3.96	1.75
4	1	1	0	1	0	0	2176.364	820.85	711.27	3.95	1.78	1	1	1	0	0	0	2182.18	816.39	713.36	3.97	1.79
4	0	0	1	1	1	0	2181.153	832.85	725.82	4.01	1.79	0	0	1	1	0	1	2179.43	835.10	724.41	4.00	1.81
4	1	0	1	0	1	0	2180.757	821.90	717.50	4.01	1.79	0	1	0	1	0	1	2173.90	831.58	719.16	3.95	1.78
4	1	1	0	0	1	0	2174.984	818.68	712.20	3.96	1.75	1	1	0	0	0	1	2173.96	820.60	711.05	3.95	1.78
4	1	0	1	0	0	1	2179.028	824.09	716.12	4.00	1.81	0	1	0	1	1	0	2174.91	829.60	720.32	3.96	1.75
5	0	1	1	1	1	0	2141.968	824.72	718.25	3.98	1.77	1	0	1	1	0	1	2140.03	823.92	711.83	4.00	1.81
5	1	0	1	1	1	0	2141.843	821.70	713.23	4.01	1.80	0	1	1	1	0	1	2140.58	826.78	716.99	3.97	1.79
5	0	0	1	1	1	1	2140.986	832.26	721.32	4.01	1.80	0	0	1	1	1	1	2140.99	832.26	721.32	4.01	1.80
5	0	1	0	1	1	1	2134.902	828.96	715.96	3.96	1.76	1	0	1	0	1	1	2140.37	821.38	713.12	4.01	1.80
5	1	0	0	1	1	1	2133.845	825.83	711.02	3.99	1.79	0	1	1	0	1	1	2140.99	824.37	718.17	3.98	1.77
5	1	1	0	1	1	0	2136.373	818.39	708.06	3.96	1.76	1	1	1	0	0	1	2140.98	815.93	708.94	3.97	1.80
5	1	1	0	0	1	1	2134.748	818.10	707.94	3.96	1.76	1	1	0	0	1	1	2134.75	818.10	707.94	3.96	1.76
5	1	1	1	0	1	0	2142.363	813.94	710.19	3.98	1.77	1	1	0	1	0	1	2135.24	820.36	706.87	3.95	1.79
5	1	1	1	1	0	0	2143.988	816.17	709.22	3.97	1.79	1	1	1	1	0	0	2143.99	816.17	709.22	3.97	1.79
6	1	0	1	1	1	1	2102.389	821.20	708.84	4.01	1.80	0	1	1	1	1	1	2102.79	824.14	713.86	3.98	1.78
6	1	1	0	1	1	1	2097.064	817.84	703.79	3.96	1.77	1	1	1	0	1	1	2102.96	813.42	705.91	3.98	1.78
6	1	1	1	1	0	1	2103.718	815.73	704.80	3.97	1.80	1	1	1	1	1	0	2105.19	813.70	706.08	3.98	1.78
7	1	1	1	1	1	1	2066.676	813.19	701.79	3.98	1.79	1	1	1	1	1	1	2066.68	813.19	701.79	3.98	1.79

Supplementary Table 9: Scaled Predictions of the Rotational Constants and ^{14}N Nuclear Quadrupole Constants for the 36 Chemically Distinct Deuterated Versions of THP Deuterium at the E2 Position with the Two Ring Pucker Isomer.

Total Number of Deuterium Substitutions	Deuterium Isotope Pattern for Ring Pucker 1						A_{pred} (MHz)	B_{pred} (MHz)	C_{pred} (MHz)	$(1.5*\chi_{aa})_{pred}$ (MHz)	$0.25*(\chi_{bb}-\chi_{cc})_{pred}$ (MHz)	Deuterium Isotope Pattern for Ring Pucker 2						A_{pred} (MHz)	B_{pred} (MHz)	C_{pred} (MHz)	$(1.5*\chi_{aa})_{pred}$ (MHz)	$0.25*(\chi_{bb}-\chi_{cc})_{pred}$ (MHz)
1	0	0	0	0	0	0	2347.510	829.43	732.10	3.96	1.78	0	0	0	0	0	0	2347.51	829.43	732.10	3.96	1.78
2	0	0	1	0	0	0	2309.215	824.66	729.80	3.97	1.80	0	0	0	1	0	0	2302.51	829.19	727.66	3.96	1.79
2	0	0	0	0	0	1	2300.856	828.90	727.50	3.96	1.79	0	0	0	0	1	0	2302.05	826.82	728.77	3.97	1.76
2	1	0	0	0	0	0	2303.537	818.11	719.30	3.96	1.79	0	1	0	0	0	0	2304.35	821.29	724.35	3.93	1.76
3	0	1	0	1	0	0	2261.443	821.00	720.08	3.93	1.77	1	0	1	0	0	0	2267.36	813.51	717.11	3.97	1.81
3	0	0	0	1	1	0	2258.360	826.55	724.37	3.97	1.77	0	0	1	0	0	1	2263.64	824.19	725.17	3.97	1.81
3	0	1	0	0	0	1	2258.894	820.76	719.86	3.93	1.77	1	0	0	0	1	0	2259.13	815.59	716.11	3.97	1.78
3	0	0	0	0	1	1	2257.553	826.22	724.31	3.97	1.77	0	0	0	0	1	1	2257.55	826.22	724.31	3.97	1.77
3	0	1	1	0	0	0	2267.429	816.49	722.24	3.94	1.78	1	0	0	1	0	0	2260.89	817.87	715.07	3.96	1.80
3	0	0	1	1	0	0	2266.019	824.46	725.40	3.97	1.80	0	0	1	1	0	0	2266.02	824.46	725.40	3.97	1.80
3	0	1	0	0	1	0	2259.741	818.88	720.98	3.94	1.74	1	0	0	0	0	1	2257.81	817.66	714.81	3.96	1.80
3	0	0	0	1	0	1	2257.047	828.68	723.05	3.96	1.80	0	0	1	0	1	0	2265.24	822.02	726.56	3.98	1.78
3	1	1	0	0	0	0	2263.201	810.10	711.98	3.93	1.77	1	1	0	0	0	0	2263.20	810.10	711.98	3.93	1.77
4	0	1	1	0	1	0	2224.254	814.05	718.96	3.95	1.76	1	0	0	1	0	1	2216.28	817.45	710.57	3.96	1.80
4	1	0	1	1	0	0	2226.374	813.31	712.92	3.97	1.81	0	1	1	1	0	0	2226.21	816.24	718.01	3.94	1.79
4	0	0	1	0	1	1	2221.735	821.48	722.06	3.98	1.79	0	0	0	1	1	1	2214.98	825.98	719.89	3.97	1.78
4	1	0	0	1	1	0	2217.710	815.34	711.90	3.97	1.78	0	1	1	0	0	1	2223.01	816.01	717.72	3.94	1.79
4	1	0	0	0	1	1	2215.494	815.08	711.75	3.97	1.78	0	1	0	0	1	1	2216.38	818.27	716.63	3.94	1.75
4	1	1	0	1	0	0	2222.456	809.83	707.91	3.93	1.78	1	1	1	0	0	0	2228.24	805.48	709.97	3.94	1.79
4	0	0	1	1	1	0	2223.270	821.79	722.18	3.98	1.79	0	0	1	1	0	1	2221.57	824.00	720.76	3.97	1.81
4	1	0	1	0	1	0	2224.352	810.97	714.00	3.98	1.79	0	1	0	1	0	1	2217.10	820.49	715.58	3.93	1.78
4	1	1	0	0	1	0	2219.579	807.77	708.75	3.94	1.75	1	1	0	0	0	1	2218.60	809.65	707.59	3.93	1.78
4	1	0	1	0	0	1	2222.651	813.11	712.60	3.97	1.81	0	1	0	1	1	0	2218.08	818.56	716.76	3.94	1.75
5	0	1	1	1	1	0	2184.205	813.78	714.75	3.95	1.77	1	0	1	1	0	1	2182.73	812.93	708.39	3.97	1.81
5	1	0	1	1	1	0	2184.513	810.76	709.82	3.98	1.80	0	1	1	1	0	1	2182.85	815.79	713.47	3.94	1.79
5	0	0	1	1	1	1	2180.827	821.27	717.68	3.98	1.80	0	0	1	1	1	1	2180.83	821.27	717.68	3.98	1.80
5	0	1	0	1	1	1	2175.769	817.98	712.39	3.94	1.76	1	0	1	0	1	1	2181.66	810.51	709.61	3.98	1.80
5	1	0	0	1	1	1	2175.132	814.85	707.53	3.97	1.79	0	1	1	0	1	1	2181.85	813.50	714.57	3.95	1.77
5	1	1	0	1	1	0	2180.007	807.47	704.71	3.94	1.76	1	1	1	0	0	1	2184.62	805.08	705.55	3.94	1.80
5	1	1	0	0	1	1	2177.021	807.25	704.49	3.94	1.76	1	1	0	0	1	1	2177.02	807.25	704.49	3.94	1.76
5	1	1	1	0	1	0	2185.976	803.13	706.81	3.95	1.77	1	1	0	1	0	1	2178.91	809.40	703.51	3.93	1.79
5	1	1	1	1	0	0	2189.051	805.25	705.93	3.94	1.80	1	1	1	1	0	0	2189.05	805.25	705.93	3.94	1.80
6	1	0	1	1	1	1	2142.827	810.31	705.42	3.98	1.80	0	1	1	1	1	1	2142.80	813.25	710.35	3.95	1.78
6	1	1	0	1	1	1	2138.445	806.98	700.43	3.94	1.77	1	1	1	0	1	1	2144.33	802.66	702.52	3.95	1.78
6	1	1	1	1	0	1	2146.444	804.87	701.50	3.94	1.80	1	1	1	1	1	0	2147.89	802.88	702.79	3.95	1.78
7	1	1	1	1	1	1	2107.193	802.43	698.49	3.95	1.79	1	1	1	1	1	1	2107.19	802.43	698.49	3.95	1.79

(C) Experimental Method and Illustration of Spectrum Analysis Approach

Experimental Method

The spectrum was measured using a 2-8 GHz chirped-pulse Fourier transform microwave spectrometer using three pulsed solenoid nozzles for sample injection.[12,13] The THP samples were delivered as a liquid with about 10 mg per sample. This sample was transferred to instrument heating reservoirs[14] by dissolving the sample in about 100 μL dichloromethane (including washing the walls of the glass sample vials). At the start of the spectrum acquisition, the sample reservoirs were maintained at 45 °C. A dry scroll pump was used to pull vacuum on the reservoirs through the neon gas input lines to remove the volatile solvent. The removal of DCM was verified by a measurement of the broadband spectrum. Once the solvent was removed, the sample was heated to 115 °C – the optimum temperature found in the initial measurements using the THP-d_0 sample. The time domain signal averages were performed until the sample was consumed – 30-100 kavg were accumulated depending on the amount of analyte available. Total measurement time was approximately 1 hour.

Spectra are available upon request: bp2k@virginia.edu

Illustration of Spectrum Analysis

The identification of deuterium labeled THP species is illustrated for one of the ***Rel-(2S,3S)-d*₂-14** samples – where the number labels designate the carbon atoms that are deuterated (Fig. S128B) - that was analyzed in this work. That analysis (see Section E) identified 5 chemically distinct species present at 1% abundance or higher. As discussed in Section A, the ***Rel-(2S,3S)-d*₂-14** sample populates two ring pucker isomers in the pulsed jet expansion. As seen in Fig. S129, the experimental spectrum is dominated by the rotational spectrum of the target THP-d_2 sample. The figure shows the experimental spectrum (black) compared to the calculated spectrum using the fit rotational constants for the two ring-pucker isomers of ***Rel-(2S,3S)-d*₂-14** (red). There are two important points in this Fig. S126) The calculated spectrum only shows the transitions from the a-type dipole moment component. This set of transitions has the highest spectral intensity, in part because there is not extensive ^{14}N hyperfine splitting (compared to the c-type transitions, for example, where the dipole moment component is larger). The a-type transitions are used exclusively for the spectrum analysis. 2) There are many transitions of comparable intensity that are not a part of the calculated spectrum. Many of these transitions are the spectrum of the axial conformational isomer of THP shown in Fig. S126.

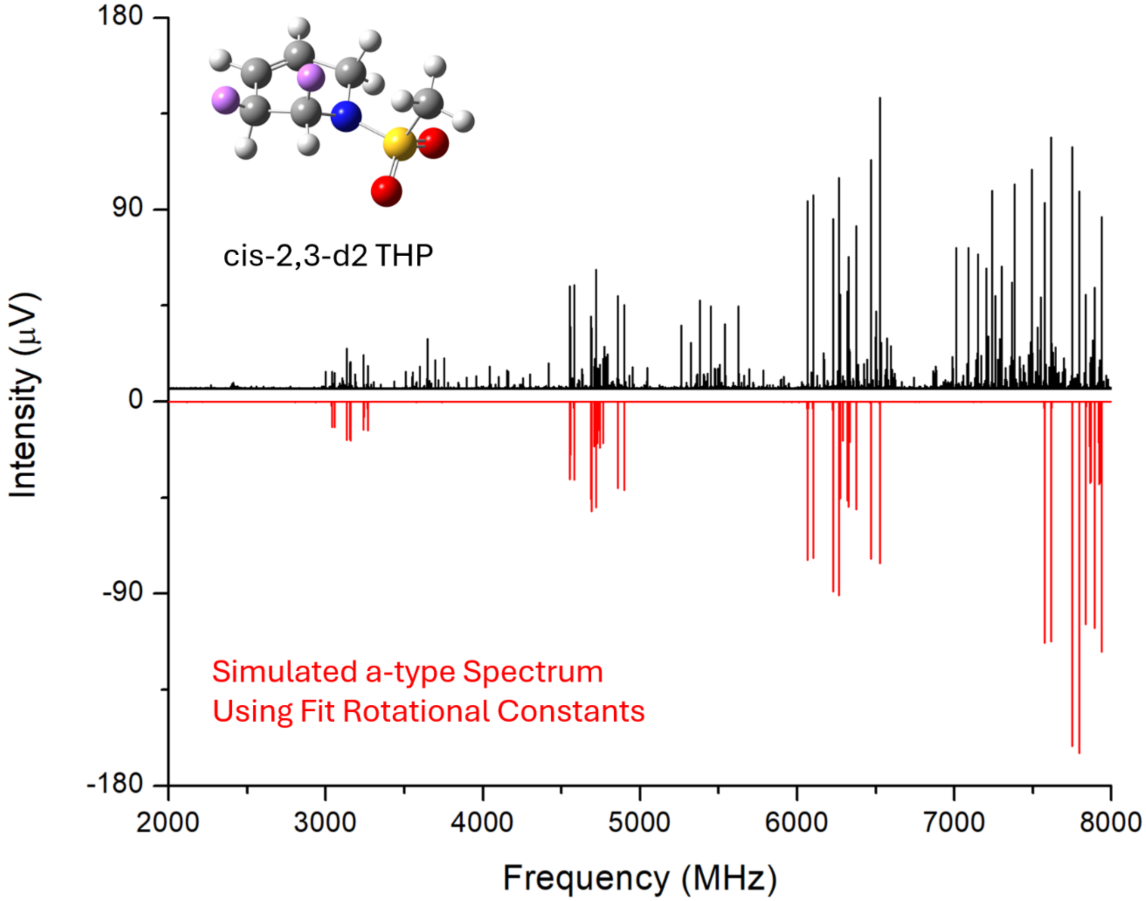

Supplementary Figure 129: The experimental broadband MRR spectrum of ***Rel-(2S,3S)-d$_2$*-14** in the 2-8 GHz frequency range is shown in black. The naming convention indicates the carbon atoms with deuterium substitutions (Fig. 128B). The structure of this isotopic variant is shown in the inset. The red spectrum is a simulation using the fit rotational constant for the two ring-pucker isomers of THP.

Isotopic impurities in the ***Rel-(2S,3S)-d$_2$*-14** sample are identified by assigning the spectral patterns for their rotational spectra. This process begins with a prediction of the spectrum for each isotopic variant under consideration. In all cases, the full set of isotopomers for the under-deuterated version of the analyte (N-1 deuterium substitutions) and over-deuterated version (N+1 deuterium substitutions) are included in the analysis. The set of misdeuteration isomers – the isotopomers of the target isotopologue – are also included. The identification of an isotopic impurity requires finding the spectral pattern that is characteristic for the impurity. Identification of a spectrum has 7 or more assigned transitions that fit within the expected experimental accuracy (a root-mean-squared frequency error of about 10 kHz or less).

For the ***Rel-(2S,3S)-d$_2$*-14** sample, the first step is verifying that dominant spectrum is consistent with predicted spectrum that uses scaled rotational constants from the theoretical equilibrium geometry. The identification of the 4_{04}-3_{03} rotational transition for the two ring pucker isomers is shown in Fig. S130. In this figure, the experimental spectrum is shown in black. The predicted spectrum using scaled theoretical rotational constants is shown in gray. The final fit spectrum is shown in red. There are three things to note: 1) The predicted spectra have good accuracy, and the experimental transition frequency is typically within 1 MHz of the prediction. 2) The hyperfine

structure from the ^{14}N nucleus is evident in the simulation and experiment. However, splitting from the hyperfine structure of the deuterium nucleus (I=1, like ^{14}N) is not observed for the a-type transitions in any of the spectra analyzed in this work. Although the hyperfine structure reduces the measurement sensitivity by fragmenting the transition strength, it is useful for identifying the experimental matches to the predicted rotational transitions through the distinctive line shape profile. 3) In cases where ring pucker isomers are expected, there is additional confidence in the spectroscopic identification because there must be a second matching spectral pattern with equal spectral intensity (within the 10-20% intensity fluctuations characteristic of the spectrometer).

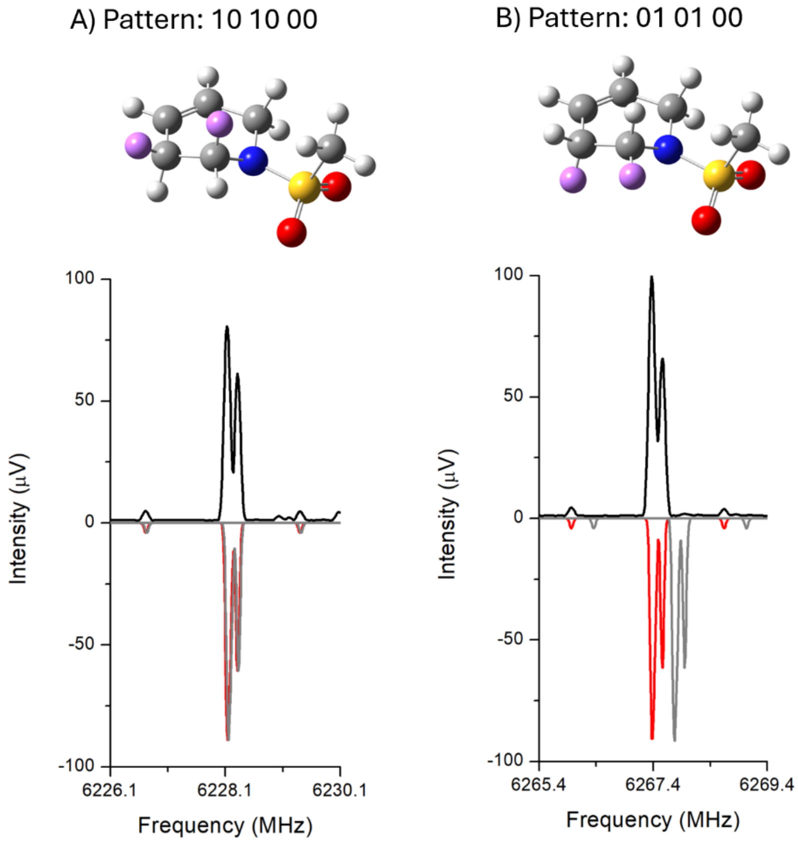

Supplementary Figure 130: The method for identifying a predicted deuterated THP species is illustrated. The spectrum in a 4 MHz window centered on the experimental 4_{04}-3_{03} transition frequency of a single deuterated THP molecule is shown in black. The spectrum simulation using the predicted rotational constants (Supplementary Table 6), with ^{14}N nuclear quadrupole fine structure is shown in gray. The spectrum simulation from the fit rotational constants is shown in red. For deuterated species with one or more isotopically chiral centers, two ring-pucker isomers are expected with equal intensity. The structures of these isomers for ***Rel-(2S,3S)-d*₂-14** is shown. The isotope pattern, used to identify the different isotopic variants, is given at the top and uses the H/D-atom labeling convention of Fig. 128A. The sample is 6-JDD-133 (see the composition results in Section E).

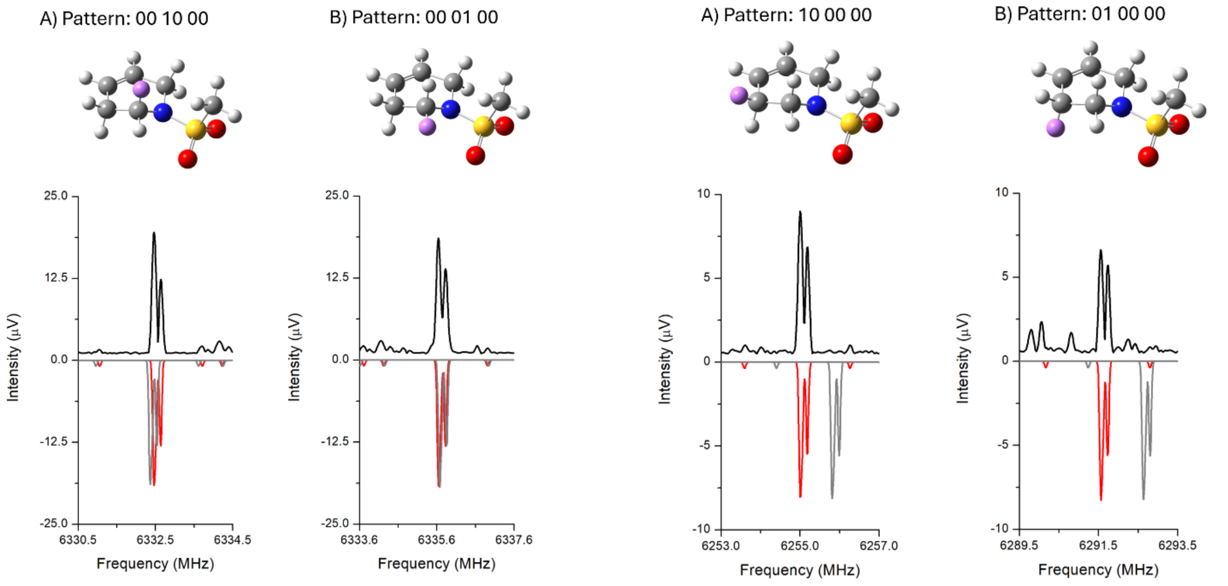

Supplementary Figure 131: The identification of the two isotopic impurities that are under deuterated is illustrated. See Fig. S130 for a description of the spectra.

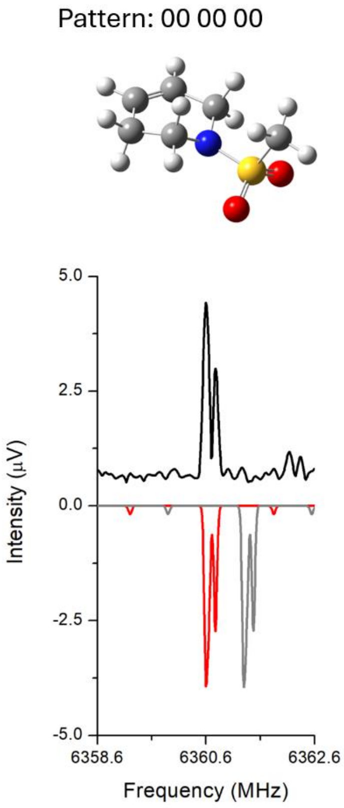

Supplementary Figure 132: The identification of THP-d_0 in the cis-2,3-d_2 sample is shown.

The spectrum identification of two relatively high abundance isotopic impurities is shown in Fig. S131. Both of these impurities have ring pucker isomers.

***d*₀-14** is also observed as an impurity in the sample – possibly as carry over from the initial measurement of this sample. In this case, there is only a single ring pucker isomer (Fig. S132). In terms of composition analysis, the total intensity of the ring pucker isomer transitions – or single transition in cases like ***d*₀-14** – is used to determine the species abundance.

The identification of a weaker isotopic impurity is shown in Fig. S133. This misdeuteration impurity makes up 1.7% of the sample. Both ring-pucker isomers are detected at 16:1 signal-to-noise ratio. For most samples, a 3:1 detection limit for species identification corresponds to a sample composition of about 0.3%.

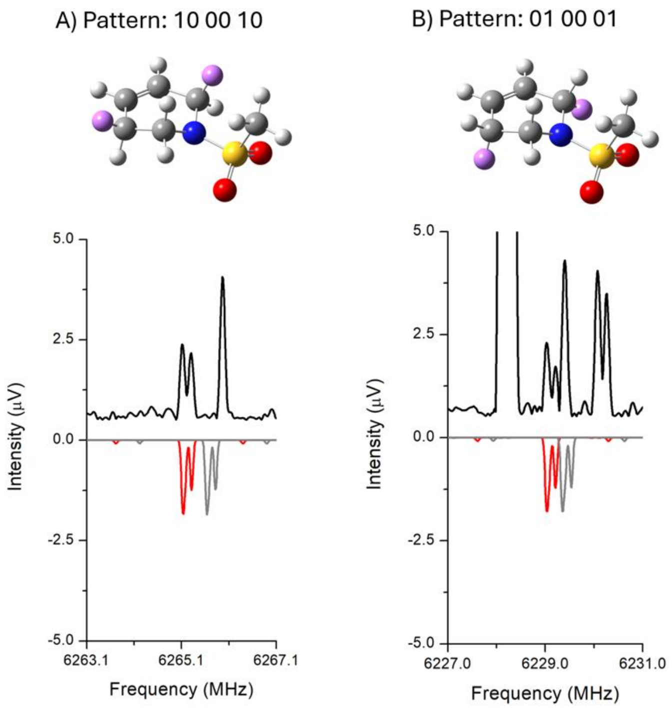

Supplementary Figure 133: The identification of a misdeuteration isotopomer of ***Rel-(2S,3S)-d*₂-14** is shown. This species is ***Rel-(2S,3S)-d*₂-14**.

A composite showing all identified species in a part of the full MRR spectrum is shown in Fig. S134.

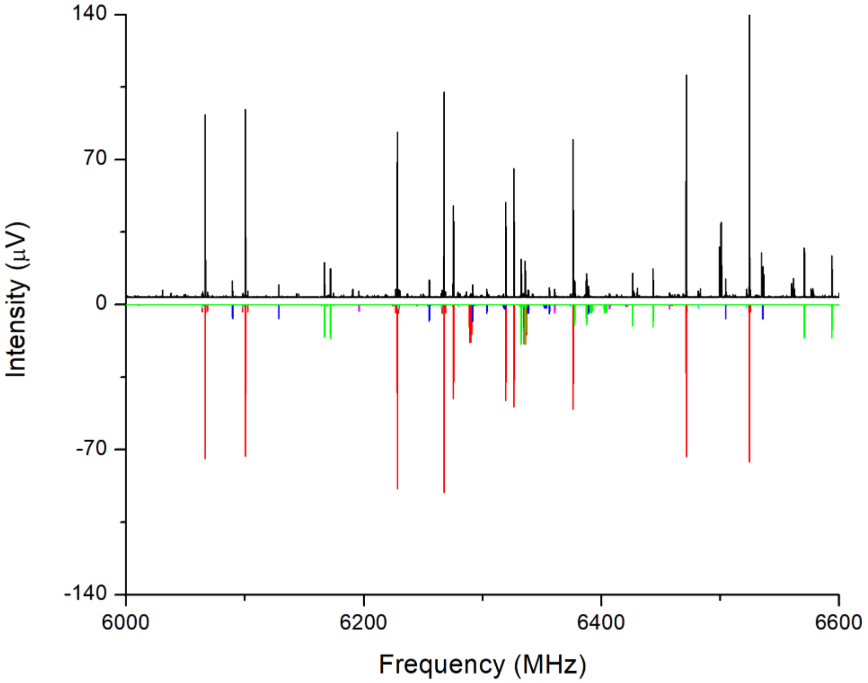

Supplementary Figure 134: A larger frequency range of the spectrum of ***Rel-(2S,3S)-d*₂-14** is shown. The simulated spectra for the species identified in Figs. S130-S133 are shown.

(D) Confidence in the Identification of Deuterated Analytes

The crucial issue in the identification of isotopic impurities is the confidence that the observed spectrum corresponds to the assigned structure. This confidence is assessed by using the root-mean-squared (rms) error in the predicted rotational constants:

$$rms(MHz) = \sqrt{\left(B_{exp} - B_{pred}\right)^2 + \left(C_{exp} - C_{pred}\right)^2} \qquad (1)$$

Note that this metric only uses the B and C rotational constants. This limitation comes from the fact that the a-type transitions are used in the spectroscopic analysis. These a-type spectra do not give a precise determination of the A rotational constant so this value is not used.

Tables S10-S13 in this section report the rotational rms errors for all 70 rotational spectra identified in this work. The deuteration pattern is defined using the structure shown in Fig. S128. The tables list all assigned spectra so that the ring pucker isomers of a species are shown as separate entries. The table gives the scaled prediction of the rotational constants from the theoretical equilibrium geometry and the experimental fit value. In several cases, where the species are only present in low abundance, the A rotational constant is fixed at the predicted value because the spectrum has low sensitivity to this parameter. This condition is denoted by listing the experimental A constant as “NF” – not fit.

The table reports the rms error to the closest matching predicted spectrum for all isotopic variants (the deuteration pattern for this best match is given in the first six columns of the table). The best rms match includes the predicted spectra for 144 different distinct chemical species (must with ring pucker isomers) listed in Tables S6-9. As the test for confident identification, the next best rms error to a predicted set of rotational constants is shown. In all cases, the rms error is a factor of three or more smaller for the best match.

Finally, there are natural abundance isotopic variants present from carbon (^{13}C), oxygen (^{18}O), and sulfur (^{33}S and ^{34}S). The natural abundance isotopic species from ^{15}N do not need to be considered because the transitions for this species would not have the hyperfine patterns that are observed in all assigned spectral transitions. The last column in the tables shows the rms error to the best matching natural abundance isotopic variant (C O S: carbon (C), oxygen (O), or sulfur (S)). Again, this rms error is significantly larger than for the assigned structure.

Supplementary Table 10: Isotopic Variants Identified in the Low Deuteration Samples.

Deuteration Pattern						A_{exp} (MHz)	B_{exp} (MHz)	C_{exp} (MHz)	A_{pred} (MHz)	B_{pred} (MHz)	C_{pred} (MHz)	RMS (MHz)	Next (MHz)	C O S (MHz)
0	0	0	0	0	0	2348.909	852.649	750.070	2348.742	852.800	750.121	0.160	4.376	7.202
0	0	1	0	0	0	2311.330	847.625	747.690	2310.609	847.684	747.638	0.079	2.622	2.867
0	0	0	0	0	1	2301.614	852.162	745.277	2301.985	852.205	745.269	0.043	0.398	2.519
1	0	0	0	0	0	2304.893	840.466	736.458	2304.953	840.609	736.534	0.162	0.586	1.036
0	1	0	0	0	0	2305.360	843.834	741.778	2305.428	844.022	741.881	0.214	2.318	0.742
0	0	0	1	0	0	2303.733	852.504	745.453	2303.743	852.521	745.448	0.018	0.351	2.847
0	0	0	0	1	0	2304.120	849.890	746.607	2303.319	850.003	746.585	0.115	2.435	3.287
1	0	1	0	0	0	2269.429	835.639	734.197	2268.957	835.688	734.174	0.054	0.679	0.766
0	1	0	1	0	0	2262.311	843.644	737.350	2262.539	843.696	737.396	0.069	0.295	1.048
1	0	0	0	1	0	2261.119	837.824	733.133	2260.536	837.927	733.141	0.104	0.565	0.331
0	1	0	0	0	1	2259.275	843.347	737.099	2259.857	843.429	737.147	0.095	0.458	1.428
1	0	1	0	1	0	2227.004	832.975	730.952	2225.930	832.983	730.865	0.087	0.664	1.067
0	1	0	1	0	1	2217.338	843.187	732.651	2218.083	843.129	732.651	0.058	2.348	2.142
1	0	1	0	0	1	2224.043	835.293	729.494	2224.092	835.242	729.419	0.091	0.315	0.920
0	1	0	1	1	0	2219.557	841.071	733.888	2219.172	841.086	733.865	0.028	0.340	1.405
0	1	1	0	0	1	2223.978	838.364	734.892	2224.115	838.350	734.845	0.049	0.356	0.730
1	0	0	1	1	0	2219.600	837.669	728.764	2219.132	837.637	728.720	0.055	0.379	1.977

Supplementary Table 11: Isotopic Variants Identified in the High Deuteration Samples.

Deuteration Pattern						A_{exp} (MHz)	B_{exp} (MHz)	C_{exp} (MHz)	A_{pred} (MHz)	B_{pred} (MHz)	C_{pred} (MHz)	RMS (MHz)	Next (MHz)	C O S (MHz)
1	0	0	1	1	0	2173.164	804.840	699.275	2172.900	804.826	699.305	0.033	0.318	2.496
0	1	1	0	0	1	2178.150	805.398	704.950	2178.854	805.400	704.962	0.012	0.335	3.046
1	0	1	0	1	0	2180.259	800.593	701.390	2179.393	800.619	701.377	0.029	2.105	1.402
0	1	0	1	0	1	2172.079	809.759	702.790	2173.060	809.719	702.846	0.069	0.802	3.053
1	0	1	0	0	1	2177.529	802.723	700.050	2177.794	802.689	700.040	0.035	0.286	0.332
0	1	0	1	1	0	2174.079	807.817	703.924	2173.967	807.844	703.973	0.056	0.305	2.328
1	0	1	1	1	1	2101.247	800.171	693.227	2101.139	799.994	693.127	0.203	2.116	0.771
1	1	0	1	1	1	2095.571	796.944	688.298	2096.101	796.892	688.326	0.059	2.266	1.634
1	1	1	0	1	1	2101.677	792.748	690.405	2101.877	792.729	690.393	0.023	0.281	0.332
1	1	1	1	0	1	2101.948	795.028	689.309	2102.634	794.917	689.310	0.111	2.107	0.498
0	1	1	1	1	1	2101.839	802.883	697.950	2101.800	802.744	697.871	0.160	0.706	1.122
1	1	1	1	1	0	2104.376	793.044	690.557	2104.000	792.987	690.552	0.057	0.356	0.150
1	1	1	1	1	1	2065.468	792.669	686.509	2065.588	792.535	686.459	0.143	3.592	0.246
1	0	1	1	0	1	2138.359	802.695	695.996	2138.809	802.545	695.945	0.159	1.840	0.928
0	1	1	1	1	0	2141.476	803.327	702.084	2140.902	803.272	702.051	0.064	0.390	1.790
1	1	0	1	1	0	2135.247	797.363	692.317	2135.316	797.389	692.391	0.078	0.241	0.471
1	1	1	0	0	1	2139.382	795.086	693.221	2139.903	795.092	693.264	0.043	0.372	0.739
0	1	0	1	0	0	NF	810.165	707.014	2214.858	810.235	707.120	0.127	0.222	6.206
1	0	1	0	0	0	NF	803.031	704.286	2219.933	803.097	704.327	0.077	1.499	3.151
0	1	0	0	1	0	NF	807.963	707.999	2214.654	808.118	708.094	0.182	0.778	6.183
1	0	0	0	0	1	NF	806.988	702.089	2212.098	807.038	702.168	0.094	0.382	0.438
0	0	0	1	1	0	NF	815.386	711.280	2214.165	815.384	711.282	0.003	0.382	12.815
0	0	1	0	0	1	NF	813.147	712.162	2219.381	813.116	712.122	0.050	0.295	12.140
1	0	1	1	1	0	NF	800.539	697.358	2140.492	800.443	697.307	0.108	0.414	0.480
0	1	1	1	0	1	NF	805.336	700.849	2139.632	805.217	700.826	0.122	0.799	1.092
1	1	1	1	0	0	NF	795.330	693.471	2142.790	795.312	693.517	0.050	0.316	0.721
1	1	1	0	1	0	NF	793.132	694.440	2141.177	793.200	694.473	0.076	2.286	0.987
1	1	0	1	0	1	NF	799.288	691.160	2134.287	799.259	691.240	0.085	2.090	0.290
0	1	1	0	1	1	NF	802.970	702.027	2139.981	802.950	701.983	0.048	0.303	2.142
1	0	0	1	1	1	NF	804.425	695.173	2132.719	804.330	695.152	0.098	2.033	2.625
1	0	1	0	1	1	NF	800.213	697.271	2139.100	800.154	697.206	0.088	0.233	0.756
0	1	0	1	1	1	NF	807.324	699.821	2134.022	807.264	699.823	0.060	0.703	1.877
1	1	0	0	0	1	NF	799.373	695.095	2173.016	799.468	695.223	0.159	0.460	0.969
1	1	0	0	1	0	NF	797.488	696.213	2173.933	797.643	696.337	0.198	2.214	0.323
0	1	1	0	1	0	NF	803.435	706.141	2180.022	803.500	706.149	0.065	0.739	4.543
1	0	0	1	0	1	NF	806.933	698.005	2171.561	806.864	698.036	0.076	1.848	3.650
1	0	1	1	0	0	NF	802.991	700.240	2179.949	802.939	700.238	0.052	0.362	0.659
0	1	1	1	0	0	NF	805.696	705.108	2180.519	805.683	705.127	0.023	0.330	3.232
0	0	1	1	0	1	NF	813.114	707.913	2178.273	812.968	707.832	0.167	0.515	8.801
0	0	1	1	1	0	NF	810.923	709.273	2179.869	810.839	709.187	0.120	0.453	8.511

Supplementary Table 12: Deuteration at Ethylene Position 1.

Deuteration Pattern						A_{exp} (MHz)	B_{exp} (MHz)	C_{exp} (MHz)	A_{pred} (MHz)	B_{pred} (MHz)	C_{pred} (MHz)	RMS (MHz)	Next (MHz)	C O S (MHz)
1	1	1	1	1	1	NF	813.368	701.876	2066.676	813.195	701.785	0.196	3.758	6.447
1	1	1	1	1	0	NF	813.788	706.134	2105.192	813.696	706.082	0.105	0.435	8.195
1	1	1	1	0	1	NF	815.878	704.833	2103.718	815.728	704.801	0.154	2.222	9.501
0	1	1	1	1	0	NF	824.809	718.336	2141.968	824.724	718.253	0.119	0.468	24.456
1	0	1	1	0	1	NF	824.102	711.917	2140.034	823.916	711.826	0.207	1.943	20.009
1	1	0	1	1	0	NF	818.400	708.024	2136.373	818.391	708.059	0.036	0.309	13.124
1	1	1	0	0	1	NF	815.956	708.937	2140.976	815.930	708.945	0.027	0.353	11.601
0	1	0	1	1	0	NF	829.608	720.314	2174.913	829.603	720.324	0.011	0.370	29.371
1	0	1	0	0	1	NF	824.152	716.169	2179.028	824.086	716.122	0.081	0.267	22.526

Supplementary Table 13: Deuteration at Ethylene Position 2.

Deuteration Pattern						A_{exp} (MHz)	B_{exp} (MHz)	C_{exp} (MHz)	A_{pred} (MHz)	B_{pred} (MHz)	C_{pred} (MHz)	RMS (MHz)	Next (MHz)	C O S (MHz)
1	1	1	1	1	1	NF	802.541	698.570	2107.193	802.428	698.491	0.138	0.727	1.081
1	1	1	1	1	0	NF	802.907	702.837	2147.890	802.877	702.795	0.052	0.400	2.386
1	1	1	1	0	1	NF	804.962	701.518	2146.444	804.867	701.496	0.097	0.738	0.425
0	1	1	1	1	0	NF	813.812	714.823	2184.205	813.779	714.755	0.076	0.402	14.761
1	0	1	1	0	1	NF	813.065	708.472	2182.728	812.931	708.394	0.155	0.648	9.174
1	1	0	1	1	0	NF	807.418	704.665	2180.007	807.472	704.708	0.069	0.243	2.823
1	1	1	0	0	1	NF	805.047	705.532	2184.623	805.079	705.550	0.037	0.444	3.615
0	1	0	1	1	0	NF	818.500	716.743	2218.078	818.559	716.756	0.060	0.256	19.004
1	0	1	0	0	1	NF	813.124	712.632	2222.651	813.113	712.596	0.038	0.341	12.524

(E) Sample Composition and Validation using NMR Spectroscopy

The sample composition is calculated using the intensities of the rotational transitions assigned to each isotopic variant. As will be discussed in more detail in a separate publication, the dipole moment variation between the different deuterated THP samples is negligible and the relative abundance of each species is proportional to the transition intensity. Chirped-pulse Fourier transform microwave spectrometers have intensity fluctuations of about 10-20%. Therefore, the analysis uses the total intensity from the 6 strongest transitions in the spectrum. Also, for species where there is a ring-pucker isomer, the abundance is proportional to the total intensity of both spectra combined – using a total of 12 measured transition intensities. In a small number of cases, a spectral overlap prevented an accurate measurement of the intensity of a single transition. In these cases, the intensity of the same transition in the corresponding ring-pucker isomer was used twice.

The sample composition is reported as the percent composition using all identified deuterated species in the sample. The composition analysis shows the set of deuterated variants of THP that were checked. In cases where the spectrum is not observed (if previously assigned) or not identified from the predicted rotational constants, the composition contribution is listed as 0%.

The accuracy of the MRR composition analysis can be checked using the expected ^{1}H NMR integrations as described previously.[10] The expected NMR integrations derived from the sample composition are reported and compared to the NMR integrations provided by the Harman group. These NMR spectra were measured on the same sample that was delivered for MRR analysis. Good agreement is observed providing validation of the MRR analysis.

The sample composition reports – with the NMR integration check at the end – are presented on the following pages.

Composition Analysis of *Rel-(2S)-d_1*-14 (5-JDD-199 Fraction B)

Supplementary Table 14: Compounds analyzed via MRR for ***Rel-(2S)-d_1*-14** sample (5-JDD-199 Fraction B).

	Ethylene1	Ethylene2	Pucker1	Pucker2		% Composition
Target	0	0	"001000"	"000100"		91.13278
--	--	--	--	--		--
Misdeuteration	0	0	"100000"	"010000"		0
--	0	0	"000010"	"000001"		5.3689
--	--	--	--	--		--
Underdeuteration	0	0	"000000"	--		3.49872
--	--	--	--	--		--
Overdeuteration	0	0	"010100"	"101000"		0
--	0	0	"000110"	"001001"		0
--	0	0	"010001"	"100010"		0
--	0	0	"000011"	--		0
--	0	0	"011000"	"100100"		0
--	0	0	"001100"	--		0
--	0	0	"010010"	"100001"		0
--	0	0	"000101"	"001010"		0
--	0	0	"110000"	--		0

Note: No carry over from previous THP samples was observed in a blank test prior to measurement.

Notation:
1) A “1” indicates that the position is deuterated, “0” indicates the position has a hydrogen.
2) For the ring pucker notation, the deuteration pattern on the three carbon atoms are given. Using the labeling from the NMR slides, the six values are:

Top C-3, Bottom C-3, Top C-2, Bottom C-2, Top C-6, Bottom C-6

NMR Carbon Atom Position:

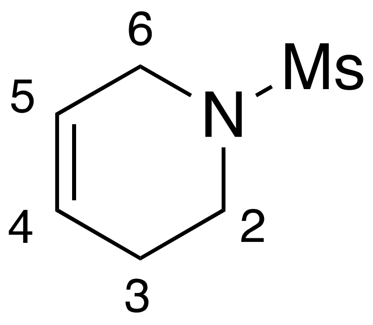

In this notation, the two ring pucker isomers for the target ***Rel-(2S)-d*****$_1$-14** (Sample 199) are:

"00 10 00"

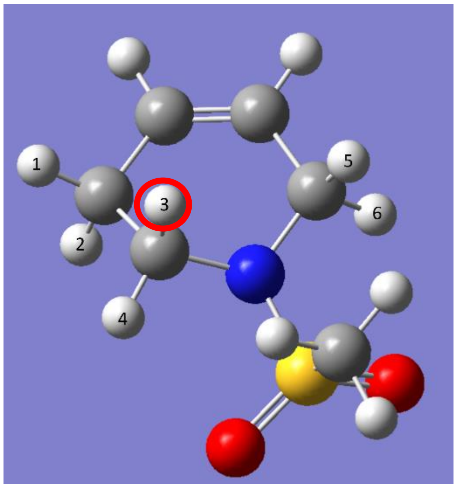

"00 01 00"

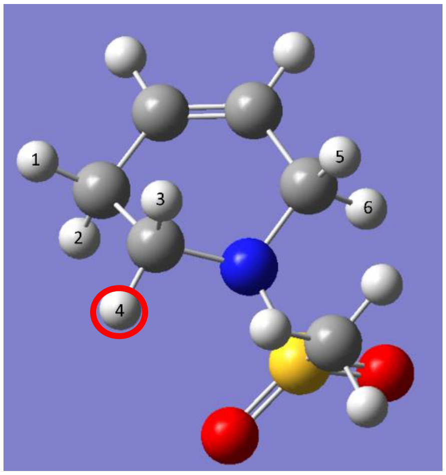

Using the MRR composition analysis, the ^{1}H NMR integrations are:

Carbon	ppm	MRR Integration	NMR Integration
C-3	2.25	2.00	2.08
C-2	3.33	1.09	1.05
C-6	3.73	1.95	2.04

Composition Analysis of *Rel-(3S)-d_1*-14 (5-JDD-121)

Supplementary Table 15: Compounds analyzed via MRR for ***Rel-(3S)-d_1*-14** sample (5-JDD-121).

	Ethylene1	Ethylene2	Pucker1	Pucker2		% Composition
Target	0	0	"100000"	"010000"		94.28181
--	--	--	--	--		--
Misdeuteration	0	0	"001000"	"000100"		0
--	0	0	"000010"	"000001"		1.04868
--	--	--	--	--		--
Underdeuteration	0	0	"000000"	--		4.02218
--	--	--	--	--		--
Overdeuteration	0	0	"010100"	"101000"		0
--	0	0	"000110"	"001001"		0
--	0	0	"010001"	"100010"		0.64697
--	0	0	"000011"	--		0
--	0	0	"011000"	"100100"		0
--	0	0	"001100"	--		0
--	0	0	"010010"	"100001"		0
--	0	0	"000101"	"001010"		0
--	0	0	"110000"	--		0

Note: This was the first low deuteration sample measured. The previous measurement was THP-d_0 and this is possibly present in the measurement as carryover. However, in this case, the spectrum was checked after cleaning of the sampling system with DCM (and replacing the gas lines). Following cleaning, the ***d_0*-14** signal was less than 1% of its previous value.

Notation:
1) A “1” indicates that the position is deuterated, “0” indicates the position has a hydrogen.
2) For the ring pucker notation, the deuteration pattern on the three carbon atoms are given. Using the labeling from the NMR slides, the six values are:

Top C-3, Bottom C-3, Top C-2, Bottom C-2, Top C-6, Bottom C-6

NMR Carbon Atom Position:

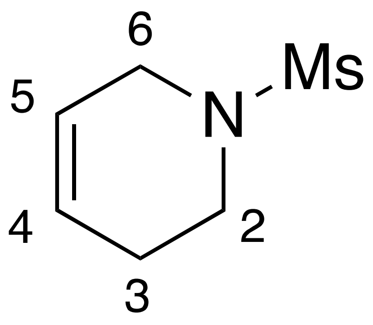

In this notation, the two ring pucker isomers for the target ***Rel-(3S)-d*$_1$-14** (Sample 121) are:

“10 00 00”

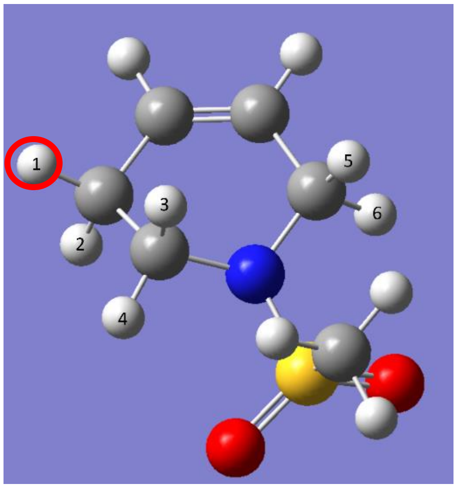

“01 00 00”

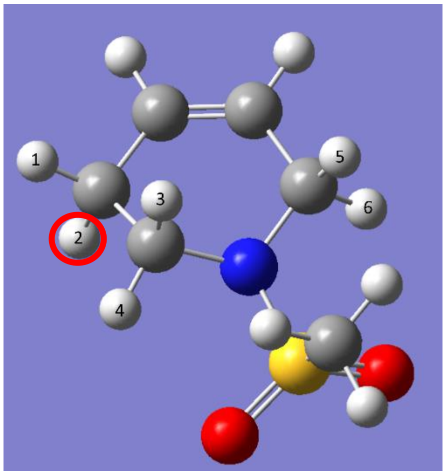

Using the MRR composition analysis, the ^{1}H NMR integrations are:

Carbon	ppm	MRR Integration	NMR Integration
C-3	2.25	1.05	1.05
C-2	3.33	2.00	2.41*
C-6	3.73	1.98	2.38*

*It is unclear why the NMR integrations are above the maximum of 2.0 in this measurement

Composition Analysis of ***Rel-(2S,3S)-d*$_2$-14** (5-JDD-205 Fraction C)

Supplementary Table 16: Compounds analyzed via MRR for ***Rel-(2S,3S)-d*$_2$-14** sample (5-JDD-205 Fraction C).

	Ethylene1	Ethylene2	Pucker1	Pucker2		% Composition
Target	0	0	"010100"	"101000"		75.99477
--	--	--	--	--		--
Underdeuteration	0	0	"100000"	"010000"		2.5378
--	0	0	"000010"	"000001"		0.25699
--	0	0	"001000"	"000100"		2.308
--	--	--	--	--		--
Double Underdeuteration	0	0	"000000"	--		0.20596
--	--	--	--	--		--
Misdeuteration	0	0	"000110"	"001001"		0
--	0	0	"010001"	"100010"		4.28825
--	0	0	"000011"	--		0
--	0	0	"011000"	"100100"		0
--	0	0	"001100"	--		0
--	0	0	"010010"	"100001"		0
--	0	0	"000101"	"001010"		0
--	0	0	"110000"	--		0
--	--	--	--	--		--
Overdeuteration	0	0	"011010"	"100101"		0
	0	0	"101100"	"011100"		0
	0	0	"001011"	"000111"		0
	0	0	"100110"	"011001"		0.45096
	0	0	"100011"	"010011"		0
	0	0	"110100"	"111000"		0
	0	0	"001110"	"001101"		0
	0	0	"101010"	"010101"		4.19975
	0	0	"110010"	"110001"		0
	0	0	"101001"	"010110"		9.75723

Note: No carry over from previous THP samples was observed in a blank test prior to measurement.

Notation:
1) A “1” indicates that the position is deuterated, “0” indicates the position has a hydrogen.
2) For the ring pucker notation, the deuteration pattern on the three carbon atoms are given. Using the labeling from the NMR slides, the six values are:

Top C-3, Bottom C-3, Top C-2, Bottom C-2, Top C-6, Bottom C-6

NMR Carbon Atom Position:

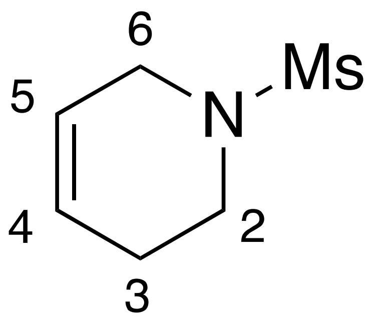

In this notation, the two ring pucker isomers for the target ***Rel-(2S,3S)-d*₂-14** (Sample 205) are:

"01 01 00"

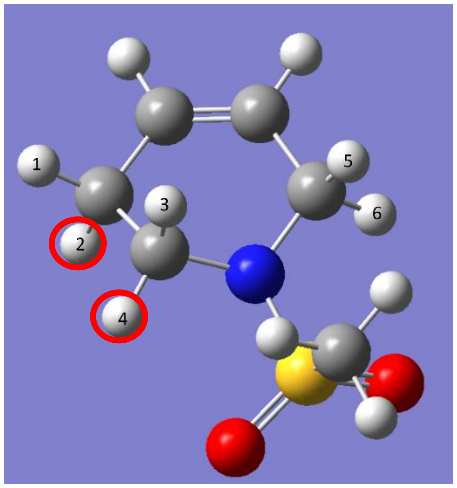

"10 10 00"

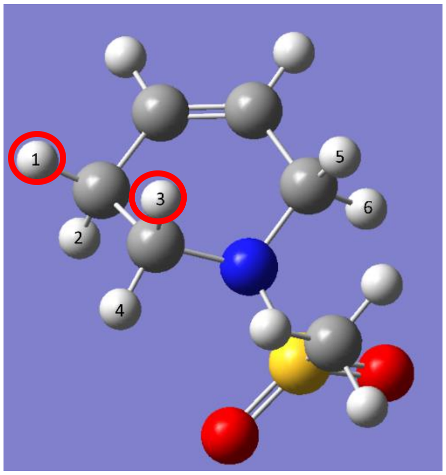

Using the MRR composition analysis, the ^{1}H NMR integrations are:

Carbon	ppm	MRR Integration	NMR Integration
C-3	2.25	1.03	1.04
C-2	3.33	1.07	1.11
C-6	3.73	1.81	1.87

Composition Analysis of *Rel-(2R,3R,6R)-d₅*-14 (6-JDD-133)

Supplementary Table 17: Compounds analyzed via MRR for ***Rel-(2R,3R,6R)-d_5*-14** sample (6-JDD-133).

	Ethylene1	Ethylene2	Pucker1	Pucker2		% Composition
Target	0	0	"010100"	"101000"		75.33638
--	--	--	--	--		--
Underdeuteration	0	0	"100000"	"010000"		6.23292
--	0	0	"000010"	"000001"		0.49655
--	0	0	"001000"	"000100"		14.37606
--	--	--	--	--		--
Double Underdeuteration	0	0	"000000"	--		1.4739
--	--	--	--	--		--
Misdeuteration	0	0	"000110"	"001001"		0
--	0	0	"010001"	"100010"		1.72588
--	0	0	"000011"	--		0
--	0	0	"011000"	"100100"		0
--	0	0	"001100"	--		0
--	0	0	"010010"	"100001"		0
--	0	0	"000101"	"001010"		0
--	0	0	"110000"	--		0
--	--	--	--	--		--
Overdeuteration	0	0	"011010"	"100101"		0
	0	0	"101100"	"011100"		0
	0	0	"001011"	"000111"		0
	0	0	"100110"	"011001"		0
	0	0	"100011"	"010011"		0
	0	0	"110100"	"111000"		0
	0	0	"001110"	"001101"		0
	0	0	"101010"	"010101"		0.35845
	0	0	"110010"	"110001"		0
	0	0	"101001"	"010110"		0

Note: No carry over from previous THP samples was observed in a blank test prior to measurement.

Notation:
1) A “1” indicates that the position is deuterated, “0” indicates the position has a hydrogen.
2) For the ring pucker notation, the deuteration pattern on the three carbon atoms are given. Using the labeling from the NMR slides, the six values are:

Top C-3, Bottom C-3, Top C-2, Bottom C-2, Top C-6, Bottom C-6

NMR Carbon Atom Position:

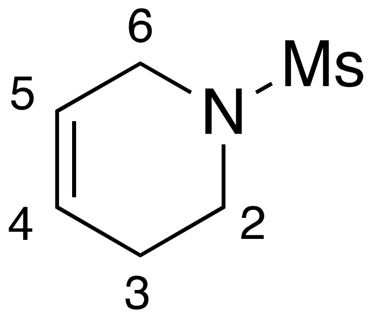

In this notation, the two ring pucker isomers for the target ***Rel-(2R,3R,6R)-d$_5$*-14** (Sample 133) are:

“01 01 00”

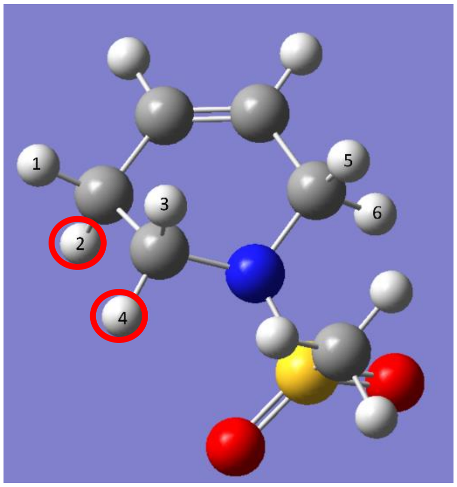

“10 10 00”

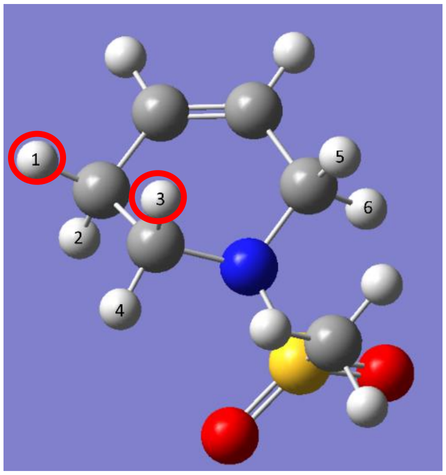

Using the MRR composition analysis, the ^{1}H NMR integrations are:

Carbon	ppm	MRR Integration	NMR Integration
C-3	2.25	1.16	1.17
C-2	3.33	1.10	1.08
C-6	3.73	1.97	2.01

Composition Analysis of ***Rel-(2R,3R,6R)-d₅*-14** (8-JDD-043)

Supplementary Table 18: Compounds analyzed via MRR for ***Rel-(2R,3R,6R)-d_5*-14** sample (8-JDD-043).

	Ethylene1	Ethylene2	Pucker1	Pucker2		% Composition
Target	1	1	"010110"	"101001"		82.36893
--	1	0	--	--		0.56483
--	0	1	--	--		0.66856
--	--	--	--	--		--
Overdeuteration	1	1	"110110"	"111001"		1.18325
--	--	--	"011110"	"101101"		0
--	1	1	"101110"	"011101"		0
--	1	1	"001111"	--		0
--	1	1	"010111"	"101011"		0.70238
--	1	1	"100111"	"011011"		0
unknown	1	1	"110011"	--		--
--	1	1	"111010"	"110101"		0
--	1	1	"111100"	--		0
--	--	--	--	--		--
Misdeuteration	1	1	"011010"	"100101"		0
--	1	1	"101100"	"011100"		0
--	1	1	"001011"	"000111"		--
--	1	1	"100110"	"011001"		3.68651
--	1	1	"100011"	"010011"		0
--	1	1	"110100"	"111000"		--
--	1	1	"001110"	"001101"		0
--	1	1	"101010"	"010101"		7.95808
--	1	1	"110010"	"110001"		0
--	--	--	--	--		--
Underdeuteration	1	1	"010100"	"101000"		0.76672
	1	1	"000110"	"001001"		0.52173
	1	1	"010001"	"100010"		0
	1	1	"000011"	--		0
	1	1	"011000"	"100100"		0
	1	1	"001100"	--		0
	1	1	"010010"	"100001"		1.57918
	1	1	"000101"	"001010"		0
	1	1	"110000"			0

The ***Rel-(2R,6R)-d_6-14*** species is detected at about 1%. This result could be carry over from a previous measurement of this target sample.

Notation:
1) A "1" indicates that the position is deuterated, "0" indicates the position has a hydrogen.
2) For the ring pucker notation, the deuteration pattern on the three carbon atoms are given. Using the labeling from the NMR slides, the six values are:

Top C-3, Bottom C-3, Top C-2, Bottom C-2, Top C-6, Bottom C-6

NMR Carbon Atom Position:

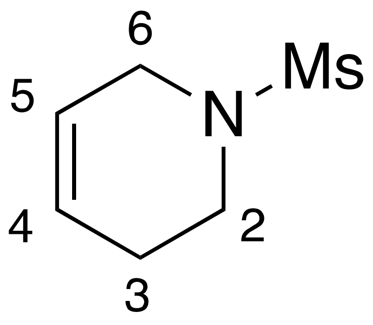

In this notation, the two ring pucker isomers for the target ***Rel-(2R,3R,6R)-d$_5$*-14** (Sample 043) are:

"01 01 10"

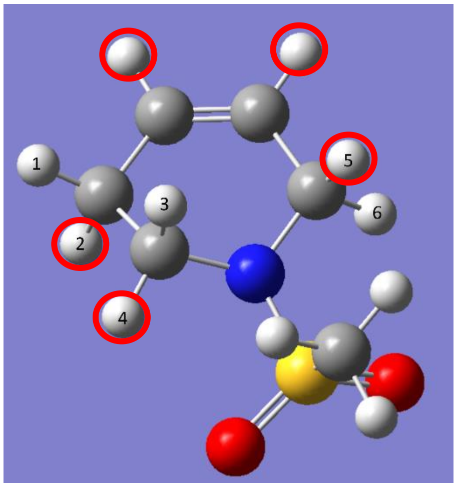

"10 10 01"

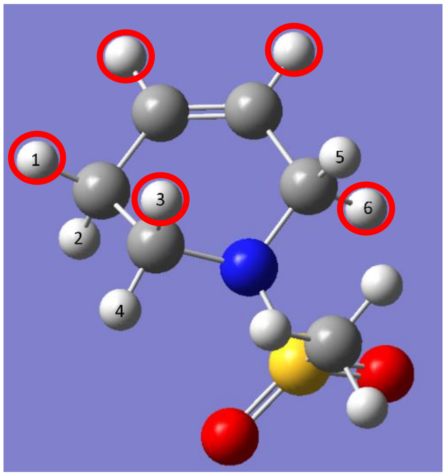

Using the MRR composition analysis, the ^{1}H NMR integrations are:

Carbon	ppm	MRR Integration	NMR Integration
C-3	2.25	0.99	1.02
C-2	3.33	1.02	1.03
C-6	3.73	1.00	1.00

Composition Analysis of *Rel-(3R,6R)-d_6*-14 (8-JDD-033)

Supplementary Table 19: Compounds analyzed via MRR for ***Rel-(3R,6R)-d_6*-14** sample (8-JDD-033).

	Ethylene1	Ethylene2	Pucker1	Pucker2		% Composition
Target	1	1	"011110"	"101101"		72.5079
--	1	0	--	--		0.64604
--	0	1	--	--		0.69107
--	--	--	--	--		--
Overdeuteration	1	1	"101111"	"011111"		0.25917
--	1	1	"110111"	"111011"		0
--	1	1	"111101"	"111110"		0
--	--	--	--	--		--
Misdeuteration	1	1	"110110"	"111001"		0
--	1	1	"101110"	"011101"		13.39437
--	1	1	"001111"	--		0
--	1	1	"010111"	"101011"		1.39912
--	1	1	"100111"	"011011"		2.67769
	1	1	"110011"	--		0
--	1	1	"111010"	"110101"		0
--	1	1	"111100"	--		0
--	--	--	--	--		--
Underdeuteration	1	1	"011010"	"100101"		4.37071
--	1	1	"101100"	"011100"		1.18501
--	1	1	"001011"	"000111"		0
	1	1	"100110"	"011001"		0.88461
--	1	1	"100011"	"010011"		0
--	1	1	"110100"	"111000"		0
--	1	1	"001110"	"001101"		0.4723
	1	1	"101010"	"010101"		0.36354
	1	1	"110010"	"110001"		0
	1	1	"101001"	"010110"		1.14843

No carryover from the ***Rel-(6R)-d_7*-14** (Sample 035) is detected. The ***Rel-(6R)-d_7*-14** sample was the only other highly deuterated sample run before this measurement.

Notation:
1) A "1" indicates that the position is deuterated, "0" indicates the position has a hydrogen.
2) For the ring pucker notation, the deuteration pattern on the three carbon atoms are given. Using the labeling from the NMR slides, the six values are:

Top C-3, Bottom C-3, Top C-2, Bottom C-2, Top C-6, Bottom C-6

NMR Carbon Atom Position:

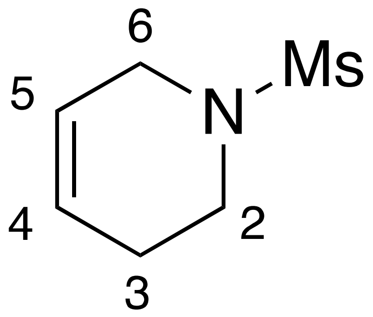

In this notation, the two ring pucker isomers for the target ***Rel-(3R,6R)-d*$_6$-14** (Sample 033) are:

"01 11 10"

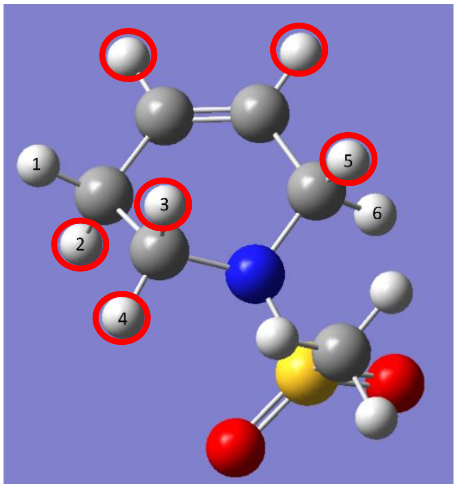

"10 11 01"

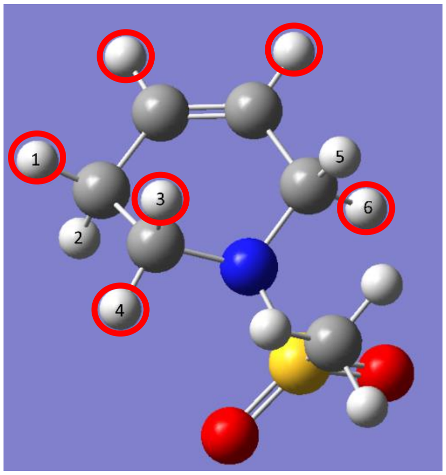

Using the MRR composition analysis, the ^{1}H NMR integrations are:

Carbon	ppm	MRR Integration	NMR Integration
C-3	2.25	1.01	0.92
C-2	3.33	0.11	Not determined
C-6	3.73	0.97	0.97

Composition Analysis of ***Rel-(2R,6R)-d_6-14*** (8-JDD-045)

Supplementary Table 20: Compounds analyzed via MRR for ***Rel-(2R,6R)-d_6*-14** sample (8-JDD-045).

	Ethylene1	Ethylene2	Pucker1	Pucker2		% Composition
Target	1	1	"110110"	"111001"		85.26603
--	1	0	--	--		0.62536
--	0	1	--	--		0.56724
--	--	--	--	--		--
Overdeuteration	1	1	"101111"	"011111"		--
--	1	1	"110111"	"111011"		0.76303
--	1	1	"111101"	"111110"		--
--	--	--	--	--		--
Misdeuteration	1	1	"011110"	"101101"		0.21762
--	1	1	"101110"	"011101"		--
--	1	1	"001111"	--		--
--	1	1	"010111"	"101011"		--
--	1	1	"100111"	"011011"		--
	1	1	"110011"	--		--
--	1	1	"111010"	"110101"		7.8728
--	1	1	"111100"	--		--
--	--	--	--	--		--
Underdeuteration	1	1	"011010"	"100101"		--
--	1	1	"101100"	"011100"		--
--	1	1	"001011"	"000111"		--
	1	1	"100110"	"011001"		0.49518
--	1	1	"100011"	"010011"		--
--	1	1	"110100"	"111000"		--
--	1	1	"001110"	"001101"		--
	1	1	"101010"	"010101"		--
	1	1	"110010"	"110001"		1.80218
	1	1	"101001"	"010110"		2.39045

The misdeuteration species in red is the ***Rel-(3R,6R)-d_6*-14** species and was measurement just before the ***Rel-(2R,6R)-d_6*-14**. This small impurity (0.22%) could come from measurement carry over.

Notation:
1) A "1" indicates that the position is deuterated, "0" indicates the position has a hydrogen.
2) For the ring pucker notation, the deuteration pattern on the three carbon atoms are given. Using the labeling from the NMR slides, the six values are:

Top C-3, Bottom C-3, Top C-2, Bottom C-2, Top C-6, Bottom C-6

NMR Carbon Atom Position:

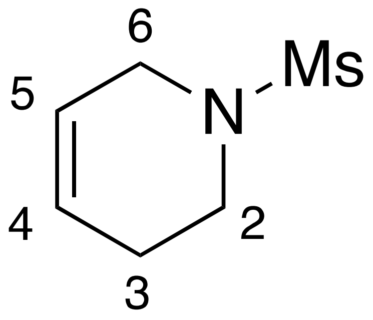

In this notation, the two ring pucker isomers for the target ***Rel-(2R,6R)-d*$_6$-14** (Sample 045) are:

"11 01 10"

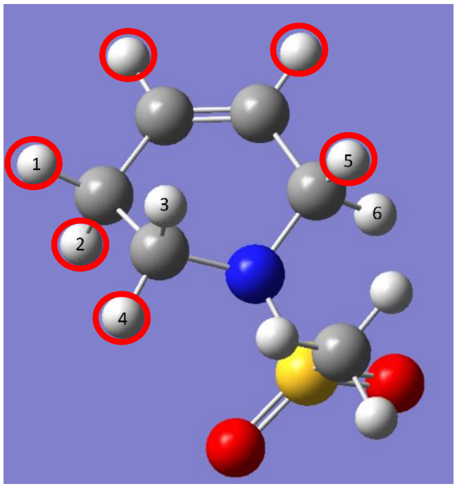

"11 10 01"

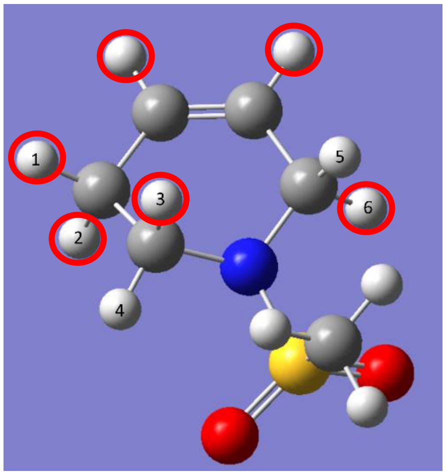

Using the MRR composition analysis, the ^{1}H NMR integrations are:

Carbon	ppm	MRR Integration	NMR Integration
C-3	2.25	0.03	Not determined
C-2	3.33	1.02	1.01
C-6	3.73	0.99	1.00

Composition Analysis of ***Rel-(3R)-d_7*-14** (8-JDD-101)

Supplementary Table 21: Compounds analyzed via MRR for ***Rel-(3R)-d_7*-14** sample (8-JDD-101).

	Ethylene1	Ethylene2	Pucker1	Pucker2		% Composition
Target	1	1	"011111"	"101111"	--	60.84316
--	1	0	--	--	--	0
--	0	1	--	--	--	0
--	--	--	--	--	--	--
Overdeuteration	1	1	"111111"	--	--	10.57101
--	--	--	--	--	--	--
Misdeuteration	1	1	"111101"	"111110'	--	4.39154
--	1	1	"110111"	"111011"	--	0.78412
--	--	--	--	--	--	--
Underdeuteration	1	1	"110110"	"111001"	--	2.34486
--	--	--	"011110"	"101101"	--	9.77839
--	1	1	"101110"	"011101"	--	7.2568
--	1	1	"001111"	--	--	0.55369
--	1	1	"010111"	"101011"	--	1.39952
--	1	1	"100111"	"011011"	--	2.07669
--	1	1	"110011"	--	--	0
--	1	1	"111010"	"110101"	--	0
--	1	1	"111100"	--	--	0

Note: There is a large overdeuteration (***d_8*-14**) impurity identified. THP ***d_8*-14** was the sample that was measured prior to the ***Rel-(3R)-d_7*-14** sample so carry over of the signal is possible. However, in all other measurements of the highly deuterated samples, the carryover was 1% or less.

Notation:
1) A “1” indicates that the position is deuterated, “0” indicates the position has a hydrogen.
2) For the ring pucker notation, the deuteration pattern on the three carbon atoms are given. Using the labeling from the NMR slides, the six values are:

Top C-3, Bottom C-3, Top C-2, Bottom C-2, Top C-6, Bottom C-6

NMR Carbon Atom Position:

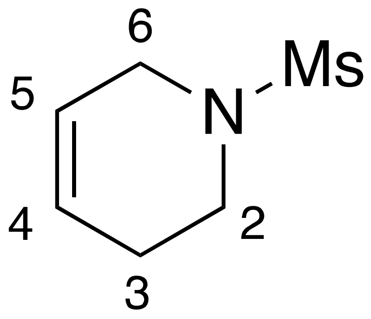

In this notation, the two ring pucker isomers for the target ***Rel-(3R)-d*$_7$-14** (Sample 101) are:

"01 11 11"

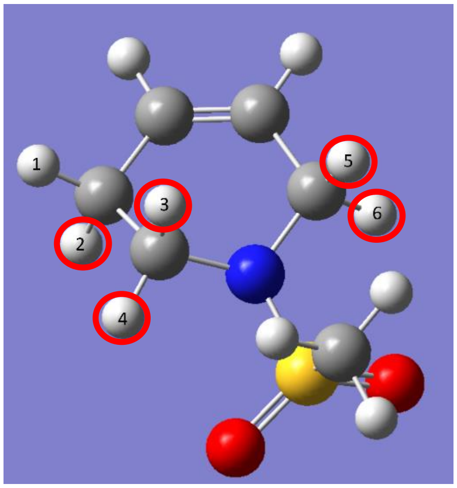

"10 11 11"

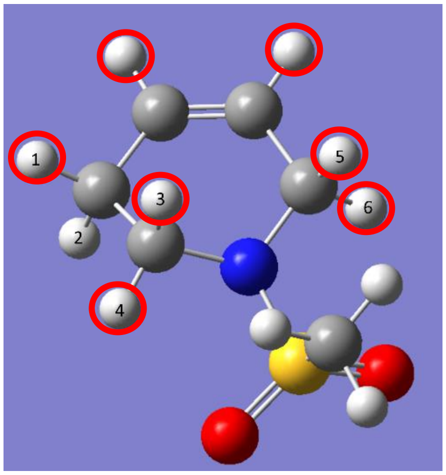

Using the MRR composition analysis, the ^{1}H NMR integrations are:

Carbon	ppm	MRR Integration	NMR Integration
C-3	2.25	0.82	0.9
C-2	3.33	0.07	0.07
C-6	3.73	0.24	0.20

Composition Analysis of d_8-14 (8-JDD-103)

Supplementary Table 22: Compounds analyzed via MRR for ***d*$_8$-14** sample (8-JDD-103).

	Ethylene1	Ethylene2	Pucker1	Pucker2		% Composition
Target	1	1	"111111"	"111111"		72.09338
--	1	0	--	--		0.62024
--	0	1	--	--		0.51022
--	--	--	--	--		--
Underdeuteration	1	1	"101111"	"011111"		2.34364
--	1	1	"110111"	"111011"		4.3249
--	1	1	"111101"	"111110"		18.78328
--	--	--	--	--		--
--	--	--	--	--		--
Double Under	1	1	"110110"	"111001"		0.85035
--	1	--	"011110"	"101101"		0
--	1	1	"101110"	"011101"		0
--	1	1	"001111"	--		0
--	1	1	"010111"	"101011"		0
--	1	1	"100111"	"011011"		0
--	1	1	"110011"	--		0
--	1	1	"111010"	"110101"		0
--	1	1	"111100"	--		0.47426

This analysis was extended to include the THP-d_6 species since one of the underdeuteration impurities was large.

Notation:
1) A "1" indicates that the position is deuterated, "0" indicates the position has a hydrogen.
2) For the ring pucker notation, the deuteration pattern on the three carbon atoms are given. Using the labeling from the NMR slides, the six values are:

Top C-3, Bottom C-3, Top C-2, Bottom C-2, Top C-6, Bottom C-6

NMR Carbon Atom Position:

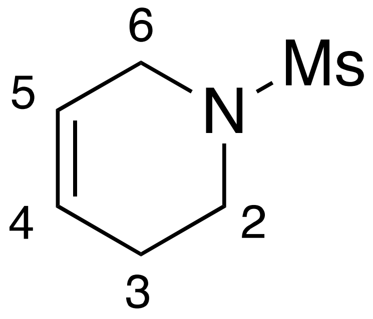

In this notation, the target ***d*₈-14** (Sample 103) is:
"11 11 11"

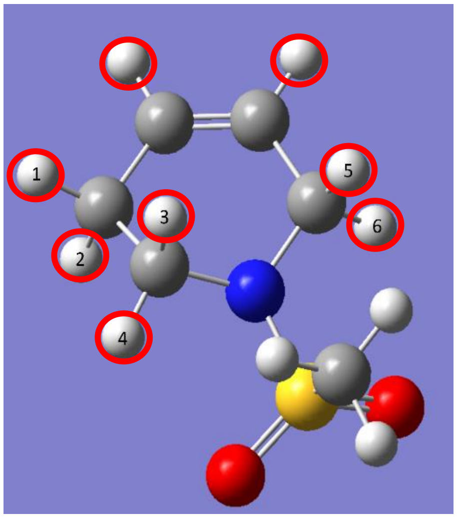

Using the MRR composition analysis, the ^{1}H NMR integrations are:

Carbon	ppm	MRR Integration	NMR Integration
C-3	2.25	0.02	Not determined
C-2	3.33	0.05	Not determined
C-6	3.73	0.21	0.17

MOLECULAR ROTATIONAL RESONANCE SPECTROSCOPY RESULTS

After subjecting each sample of **14** to MRR, analysis of each spectrum was then thoroughly conducted and observed peaks were matched to computational simulated peaks, which were in excellent agreement. The composition of each sample was conclusively determined with a detection limit of 0.2% of the overall sample for each impurity (Supplementary Table 22). Based on these results, several common impurity pathways were proposed to account for each of these impurities (Supplementary Figure 135). A sample-by-sample breakdown of species sought, detected, mechanistically explained follows.

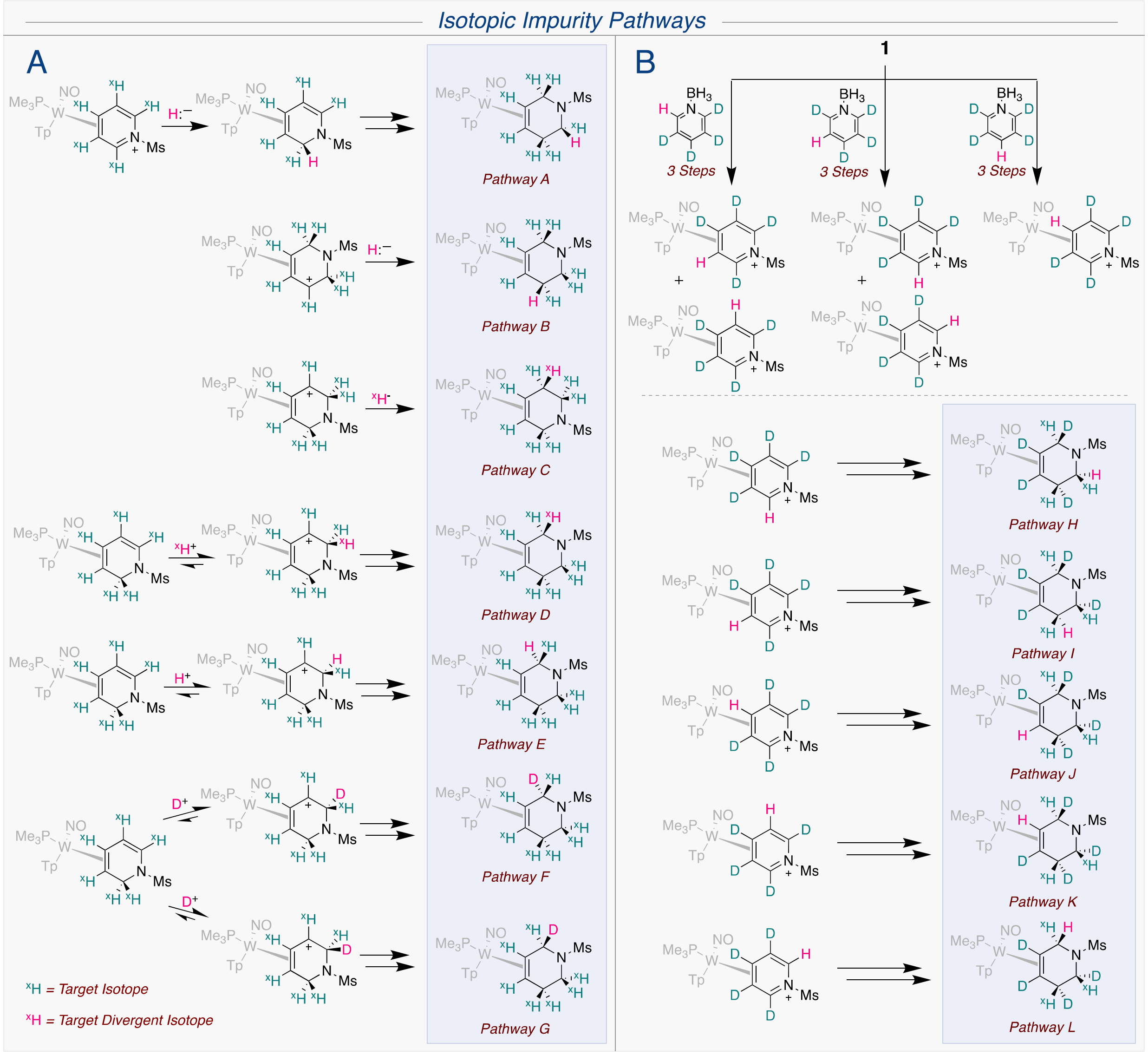

Supplementary Figure 135: (A) Various impurity pathways observed across samples. (B) Impurities generated stemming from the coordination of various d_4-pyridine borane species to [W].

Supplementary Table 22: Compounds detected via MRR for each deuterated analogue of **14**. The detection limit is ≥0.2% of the overall sample composition.

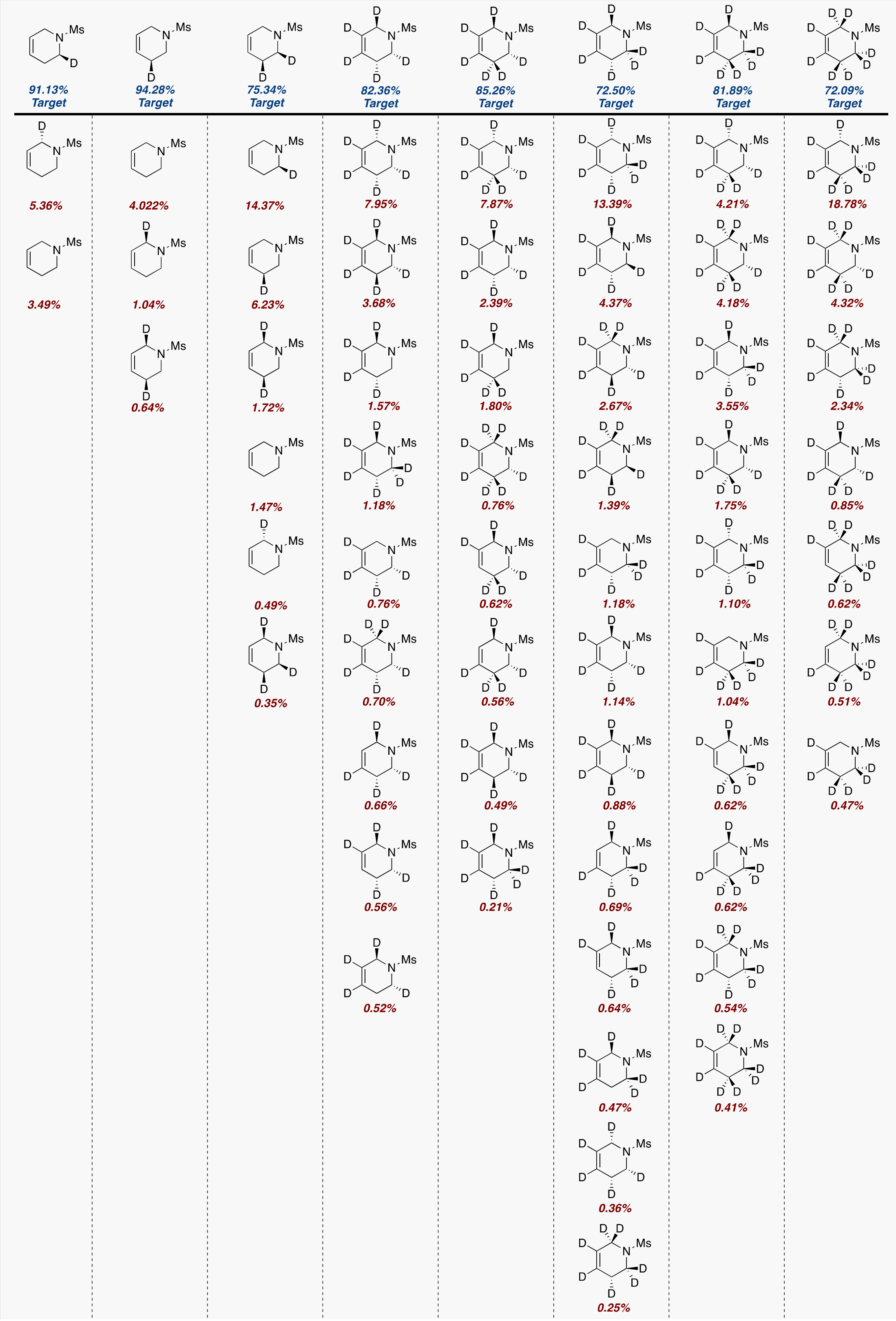

Sample: *Rel-(2S)-d₁*-14

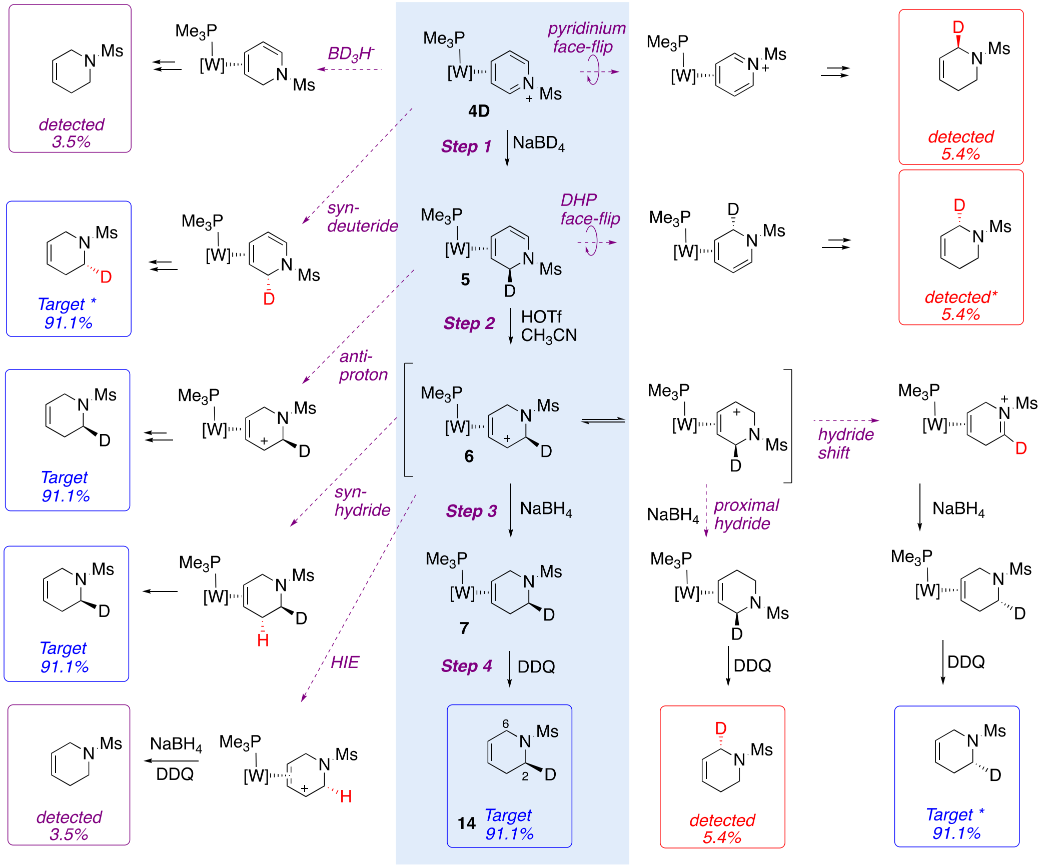

Supplementary Figure 122: Proposed impurity tree for ***Rel-(2S)-d₁*-14** based on the MRR results.

Sample: *Rel-(3S)-d*$_1$-14

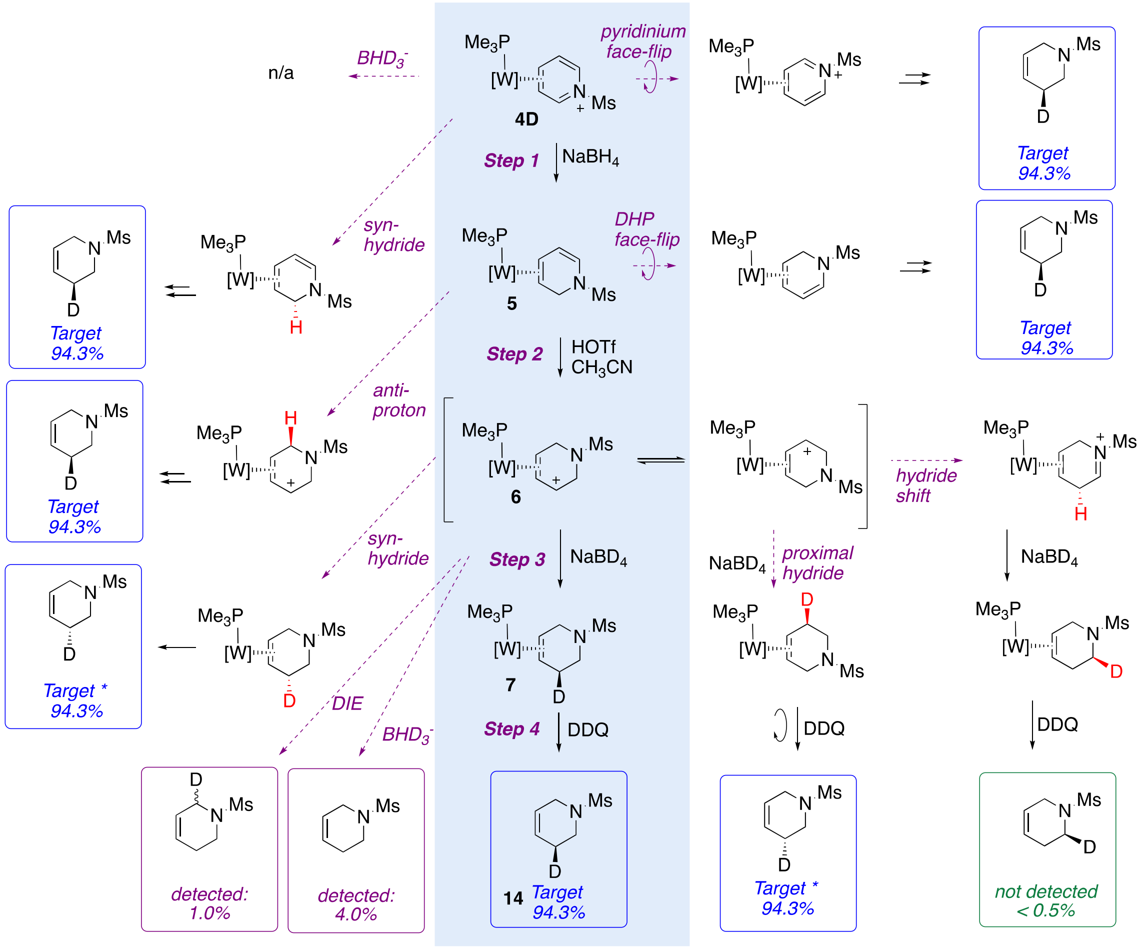

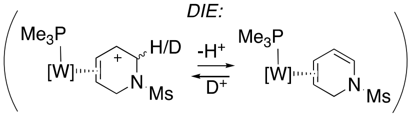

Supplementary Figure 123: Proposed impurity tree for ***Rel-(3S)-d*$_1$-14** based on the MRR results.

Sample: *Rel-(2S,3S)-d₂-14*

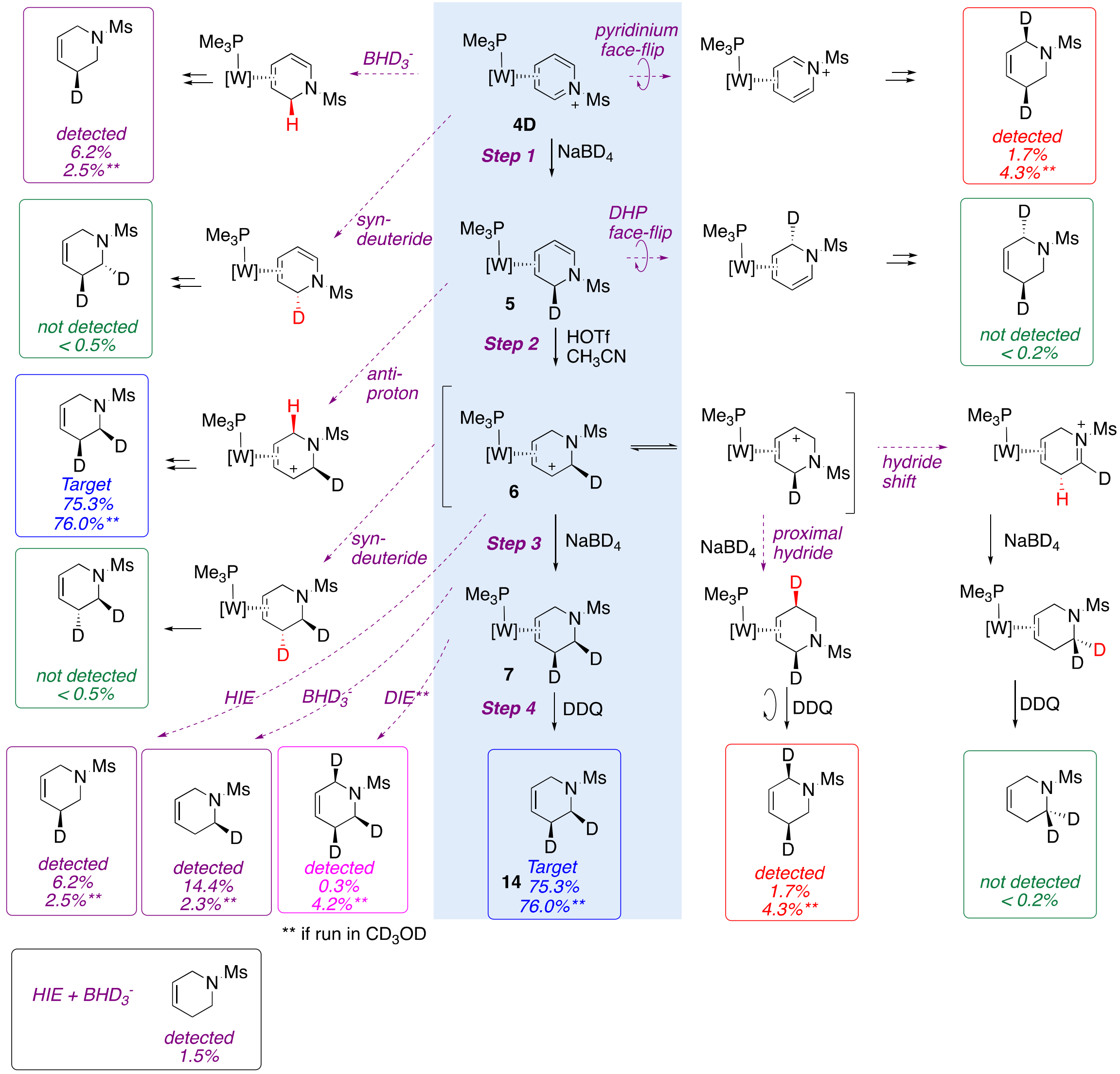

Supplementary Figure 124: Proposed impurity tree for ***Rel-(2S,3S)-d₂-14*** based on the MRR results.

Sample: *Rel-(2R,3R,6R)-d₅-14*

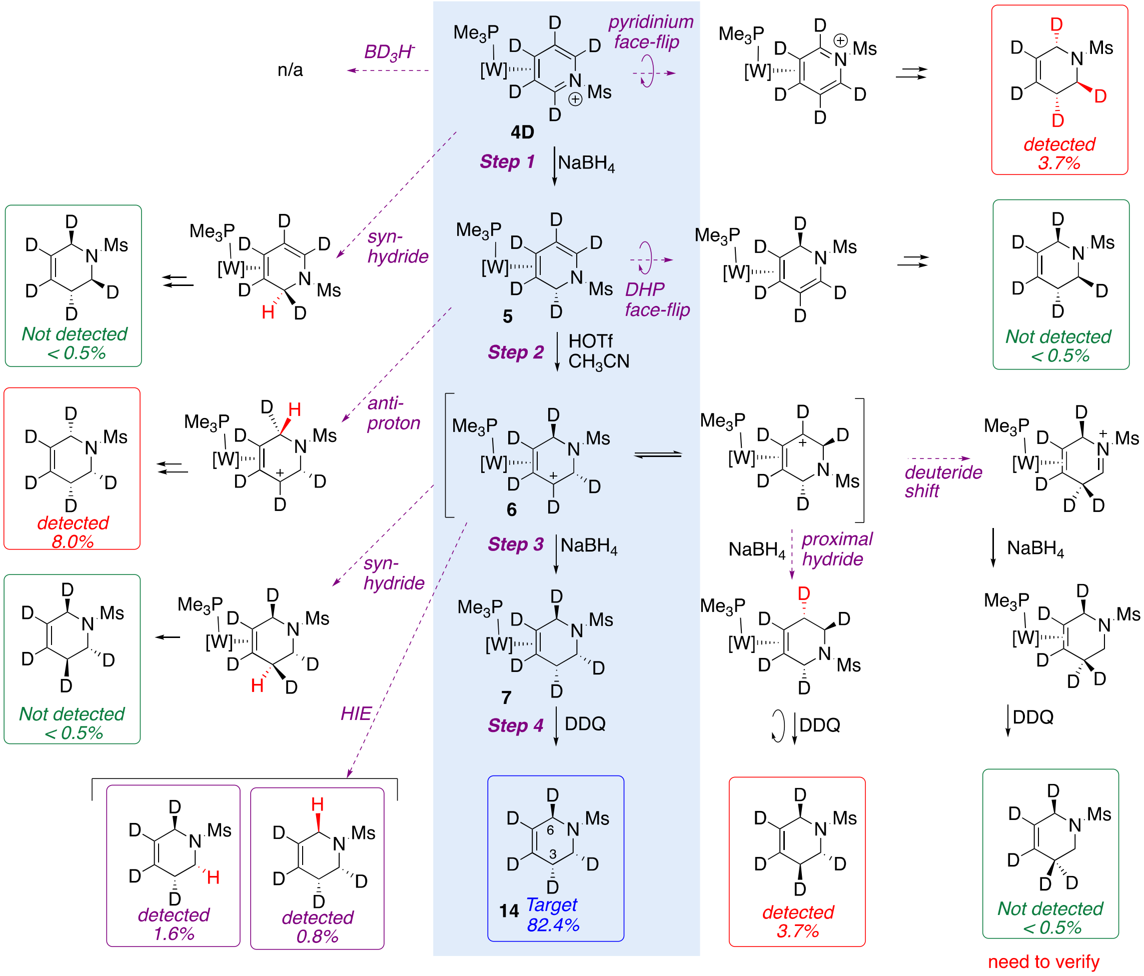

Supplementary Figure 125: Proposed impurity tree for ***Rel-(2R,3R,6R)-d₅*-14** based on the MRR results.

Sample: *Rel-(3R,6R)-d₆-14*

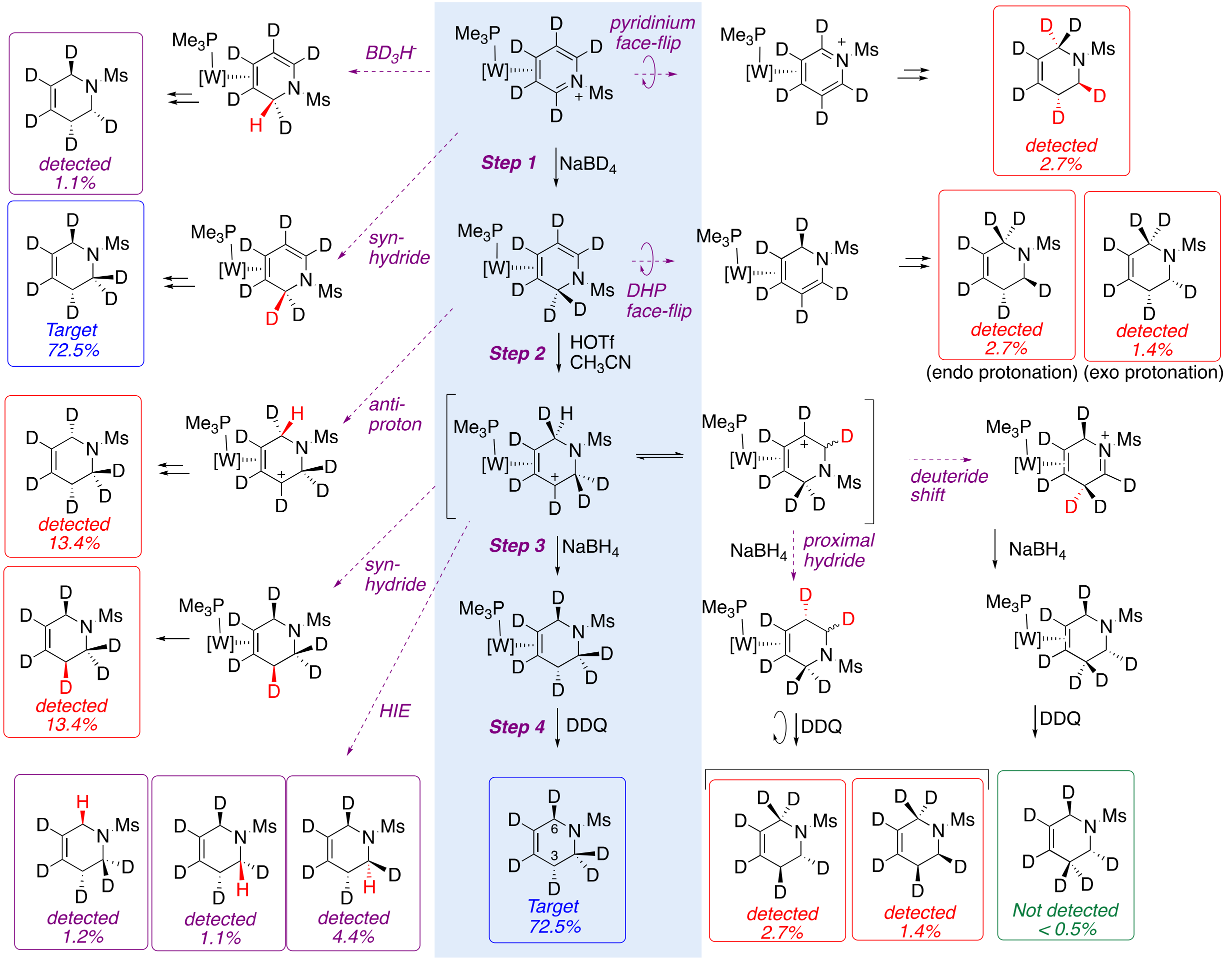

Supplementary Figure 126: Proposed impurity tree for ***Rel-(3R,6R)-d_6-14*** based on the MRR results.

Sample: ***Rel-(2R,6R)-d$_6$-14***

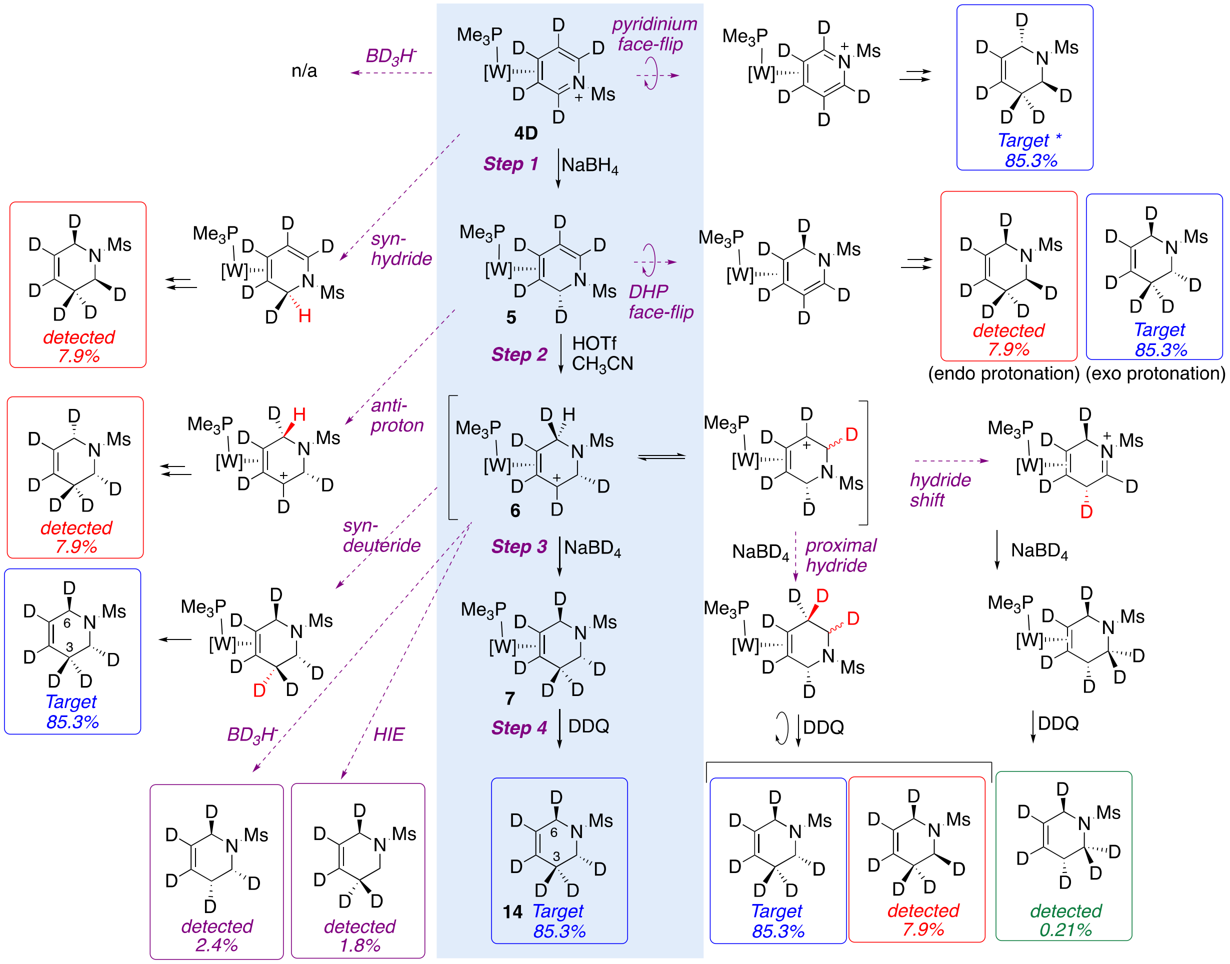

Supplementary Figure 127: Proposed impurity tree for ***Rel-(2R,6R)-d$_6$-14*** based on the MRR results.

Sample: *Rel-(6R)-d₇-14*

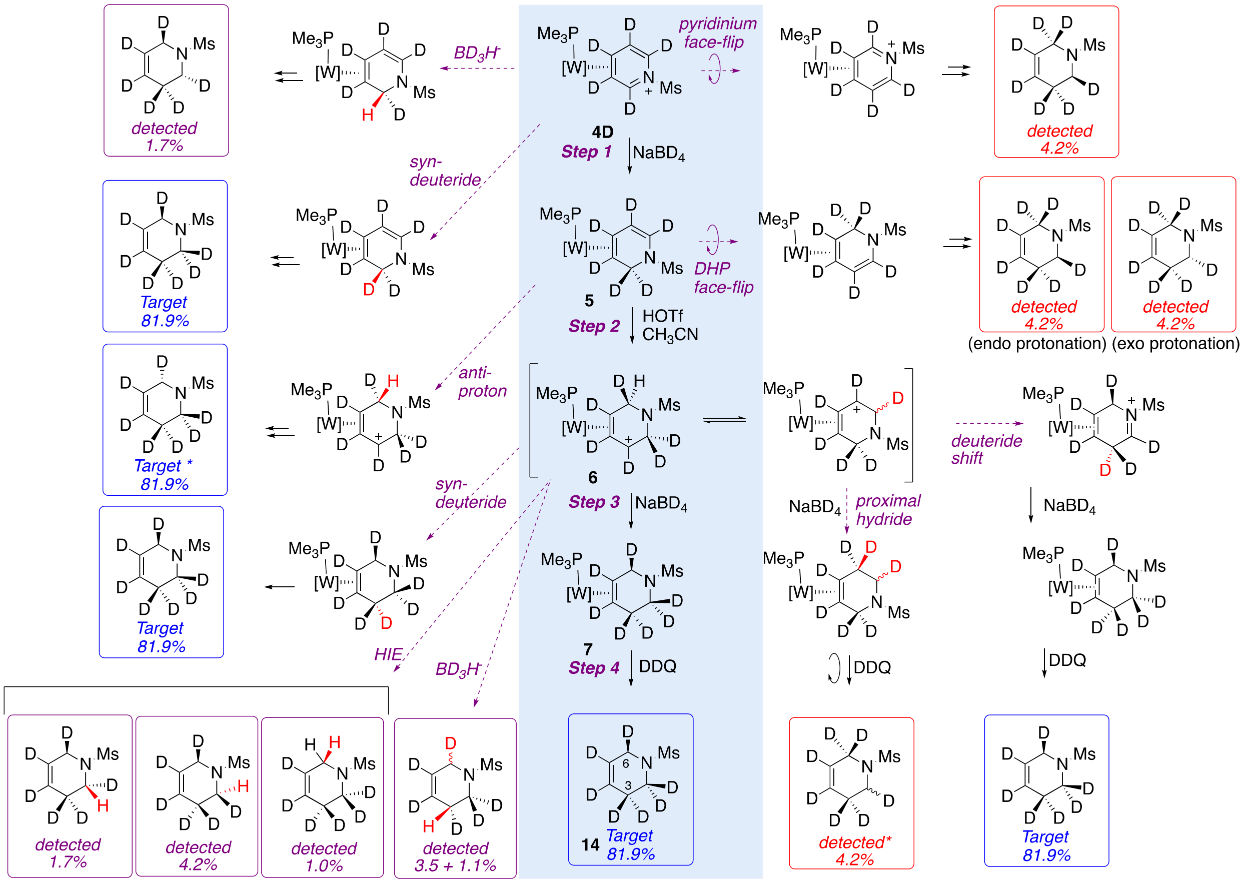

Supplementary Figure 128: Proposed impurity tree for ***Rel-(6R)-d₇-14*** based on the MRR results.

Sample: *Rel-(3R)-d$_7$*-14

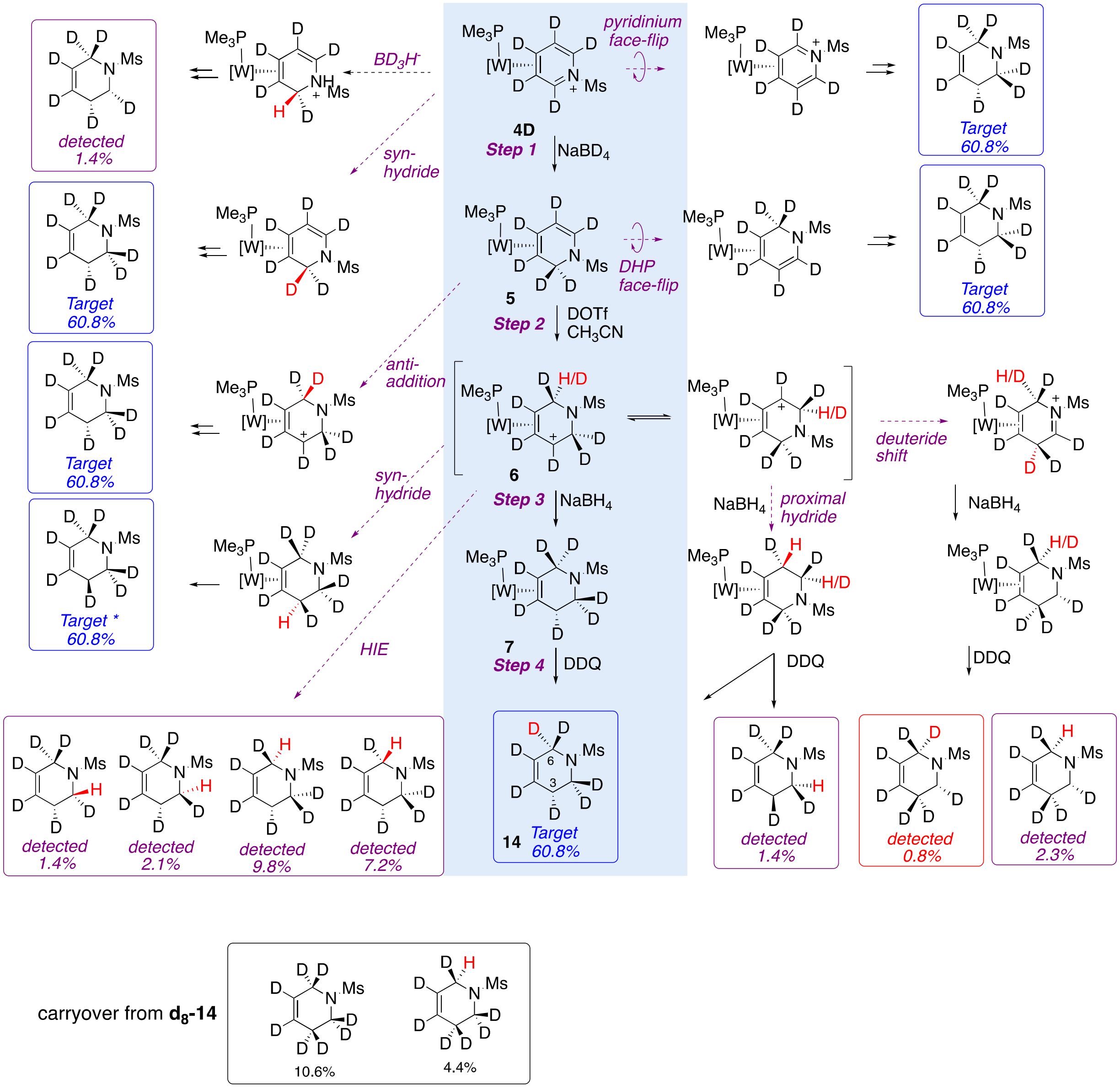

Supplementary Figure 129: Proposed impurity tree for ***Rel-(3R)-d$_7$*-14** based on the MRR results.

Sample: d_8-14

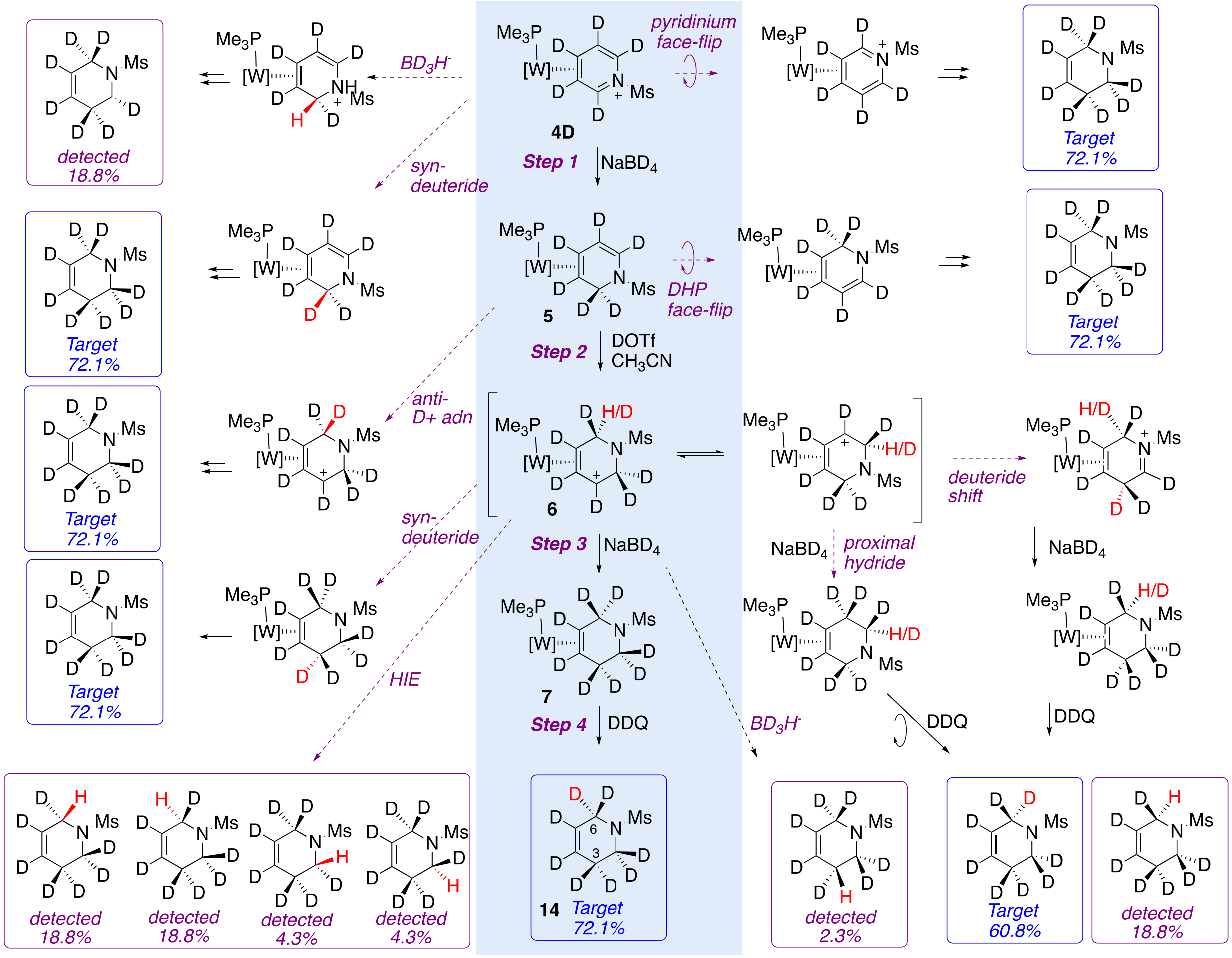

Supplementary Figure 130: Proposed impurity tree for ***d*₈-14** based on the MRR results.

Supplementary References:

(1) P. V. Ramachandran, A. S. Kulkarni, Y. Zhao, J. Mei, Amine-boranes bearing borane-incompatible functionalities: application to selective amine protection and surface functionalization. *Chem. Commun. (Camb.)* **52**, 11885-11888 (2016).

(2) D. P. Harrison, K. D. Welch, A. C. Nielander, M. Sabat, W. H. Myers, W. D. Harman, Efficient synthesis of an η^2-pyridine complex and a preliminary investigation of the bound heterocycle's reactivity. *J. Am. Chem. Soc.* **130**, 16844-16845 (2008).

(3) D. A. Delafuente, G. W. Kosturko, P. M. Graham, W. H. Harman, W. H. Myers, Y. Surendranath, R. C. Klet, K. D. Welch, C. O. Trindle, M. Sabat, W. D. Harman, Isomerization dynamics and control of the η^2/N equilibrium for pyridine complexes. *J. Am. Chem. Soc.* **129**, 406-416 (2007).

(4) J. D. Dabbs, M. N. Ericson, J. H. Wilde, R. F. Lombardo, E. C. Ashcraft, D. A. Dickie, W. D. Harman, The Tungsten-Promoted Synthesis of Piperidyl-Modified *erythro*-Methylphenidate Derivatives. *ACS Cent. Sci.* **9**, 1775-1783 (2023).

(5) Neill, J. L.; Evangelisti, L.; Pate, B. H. Analysis of isomeric mixtures by molecular rotational resonance spectroscopy. *Analytical Science Advances* **2023**, *4* (5-6), 204-219. DOI: https://doi.org/10.1002/ansa.202300021.

(6) Gordy, W.; Cook, R. L. Asymmetric Top Molecules, in *Techniques of Chemistry, Microwave Molecular Spectra*, Wiley (Knovel), 1984; pp 227-296.

(7) Gordy, W.; Cook, R. L. The Distortable Rotor, in *Techniques of Chemistry, Microwave Molecular Spectra*, 1984; pp 297-390.

(8) Grimme, S.; Steinmetz, M. Effects of London dispersion correction in density functional theory on the structures of organic molecules in the gas phase. *Phys Chem Chem Phys* **2013**, *15* (38), 16031-16042. DOI: 10.1039/c3cp52293h.

(9) *Gaussian 16 Rev. C.01*; Wallingford, CT, 2016.

(10) Vang, Z. P.; Reyes, A.; Sonstrom, R. E.; Holdren, M. S.; Sloane, S. E.; Alansari, I. Y.; Neill, J. L.; Pate, B. H.; Clark, J. R. Copper-Catalyzed Transfer Hydrodeuteration of Aryl Alkenes with Quantitative Isotopomer Purity Analysis by Molecular Rotational Resonance Spectroscopy. *Journal of the American Chemical Society* **2021**, *143* (20), 7707-7718. DOI: 10.1021/jacs.1c00884.

(11) Sloane, S. E.; Vang, Z. P.; Nelson, G.; Qi, L.; Sonstrom, R. E.; Alansari, I. Y.; Behlow, K. T.; Pate, B. H.; Neufeldt, S. R.; Clark, J. R. Precision Deuteration Using Cu-Catalyzed Transfer Hydrodeuteration to Access Small Molecules Deuterated at the Benzylic Position. *JACS Au* **2023**, *3* (6), 1583-1589. DOI: 10.1021/jacsau.3c00053.

(12) Brown, G. G.; Dian, B. C.; Douglass, K. O.; Geyer, S. M.; Shipman, S. T.; Pate, B. H. A broadband Fourier transform microwave spectrometer based on chirped pulse excitation. *Rev Sci Instrum* **2008**, *79* (5), 053103. DOI: 10.1063/1.2919120 READCUBE.

(13) Pérez, C.; Lobsiger, S.; Seifert, N. A.; Zaleski, D. P.; Temelso, B.; Shields, G. C.; Kisiel, Z.; Pate, B. H. Broadband Fourier transform rotational spectroscopy for structure determination: The water heptamer. *Chemical Physics Letters* **2013**, *571*, 1-15. DOI: 10.1016/j.cplett.2013.04.014.

(14) Suenram, R. D.; Grabow, J. U.; Zuban, A.; Leonov, I. A portable, pulsed-molecular-beam, Fourier-transform microwave spectrometer designed for chemical analysis. *Review of Scientific Instruments* **1999**, *70* (4), 2127-2135. DOI: 10.1063/1.1149725.
